# Over 20,000 precolonial earthworks in the Southwest Amazonia

**DOI:** 10.1038/s41586-026-10835-7

**Published:** 2026-07-29

**Authors:** Martti Pärssinen, Risto Kalliola, Alceu Ranzi, Eetu Puttonen, Rhuan Carlos Lopes, Pirjo Kristiina Virtanen, Francisco Apurinã, Kalle Ruokolainen, Juha Hyyppä, Antero Kukko, Mariana Campos, Fabio de Novaes, Markku Oinonen, Antonia D. Barbosa, Sanna Saunaluoma, Evandro Ferreira

**Affiliations:** 1 Instituto Iberoamericano de Finlandia, Madrid, Spain; 2 Indigenous Studies, Faculty of Humanities, University of Helsinki, Helsinki, Finland; 3 Department of Geography and Geology, University of Turku, Turku, Finland; 4 Laboratório de Pesquisas Paleontológicas, Universidade Federal do Acre, Rio Branco, Brazil; 5 Finnish Geospatial Research Institute, Espoo, Finland; 6 Instituto de Filosofia e Ciências Humanas, Universidade Federal do Pará, Belém, Brazil; 7 Programa Associado de Pós-Graduação em Antropologia, Universidade Federal do Ceará e Universidade da Integração Internacional da Lusofonia Afro-Brasileira, Redenção, Brazil; 8 Department of Biology, University of Turku, Turku, Finland; 9 Department of Biology, Aarhus University, Aarhus, Denmark; 10 Department of Built Environment, Aalto University, Espoo, Finland; 11 RuralTech, Brasília DF, Brazil; 12 Laboratory of Chronology, Finnish Museum of Natural History, University of Helsinki, Helsinki, Finland; 13 Instituto do Patrimônio Histórico e Artístico Nacional, Rio Branco, Brazil; 14 Degree Program in Digital Culture, Landscape and Cultural Heritage, University of Turku, Turku, Finland; 15 Herbário da Universidade Federal do Acre, Rio Branco, Brazil

**Keywords:** Archaeology, Environmental impact, Geography, Forestry, Palaeoclimate

## Abstract

Numerous geometric earthworks have been found in southwestern Amazonia, evidence of a monument-building precolonial civilization^1–3^. This knowledge has contributed to the discussion about the deep history and the precolonial urbanism of Amazonia, its sociocultural contexts and its environmental legacy^2,4–8^. Estimates of the geographical extent and population size of the forest civilization have remained weak, because the detection of earthworks has been almost exclusively limited to deforested areas where vegetation does not completely hide the structures. Here we used canopy-penetrating LiDAR data over 4,430 km of flight to define the geographical distribution and quantity of earthworks in this region better. On the basis of both existing data and our LiDAR documentation, we show that this cultural area alone contains as many earthworks as previously estimated for the whole of Amazonia. We also propose that the total human population of the area in 100–300 ad was 1.25–3 million people. If similar results are found elsewhere in Amazonia, the total precolonial population density and its effect on the current Amazonian soil and forest structure, biodiversity and even some modelling of the global climate should be re-evaluated.

## Abstract (web summary)

Using canopy-penetrating LiDAR data, the northern part of southwestern Amazonia appears to contain as many earthworks as previously estimated for the whole of Amazonia.

## Main

**Fig. 1:**
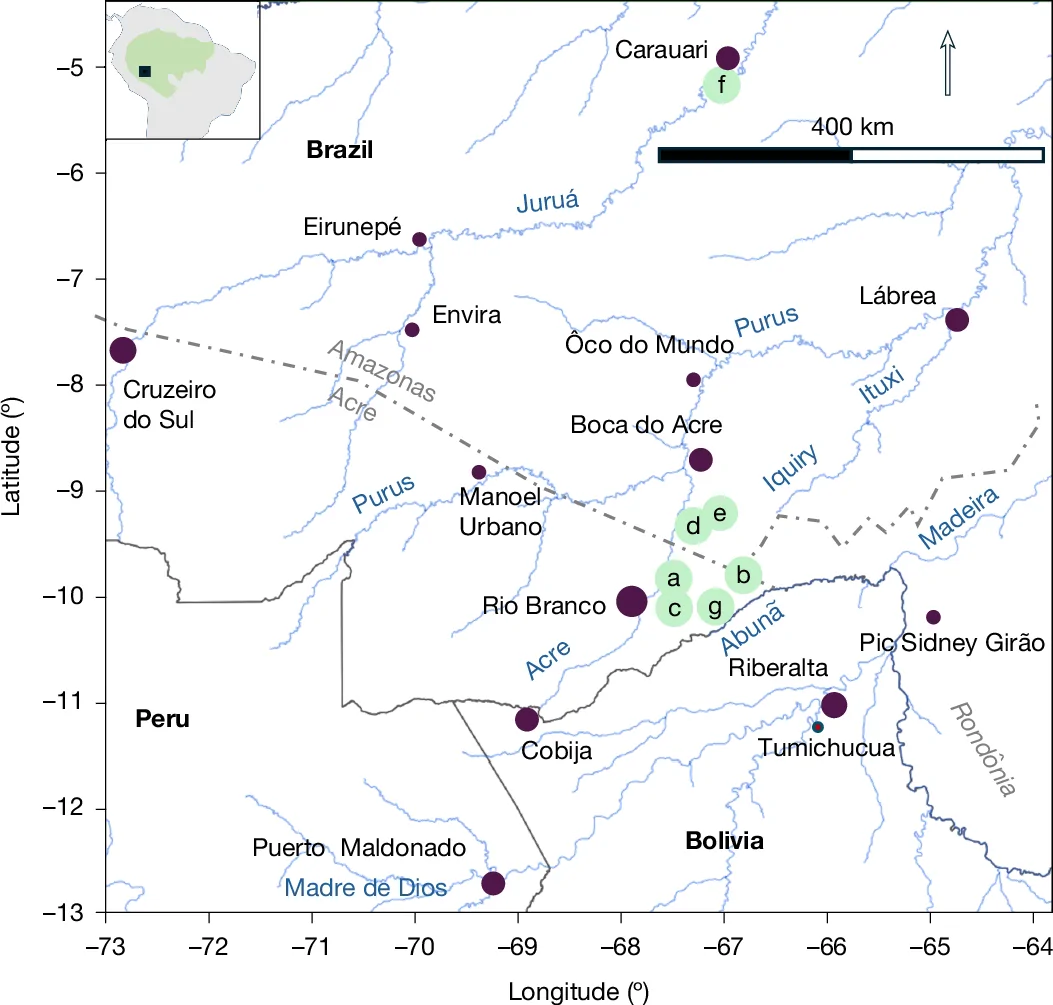
Map of the study area. The green points for a–f indicate the locations of images provided in Fig. 2; point g shows the location in Extended Data Fig. 4a. Data of rivers are from ref. ^50^.

The discovery of hundreds of geometrically patterned ditched earthworks, most notably in Brazilian Acre but also in the surrounding states (Fig. 1), has altered previous thinking about human occupation and effect on tropical forests in South America.

These so-called geoglyph-type earthworks prove that not only the floodplains but also the interfluvial hinterland (terra firme)—covering more than 90% of Amazonia—supported relatively dense human populations in some places with significant effects on forest structure^1–3,6,9,10^. At the same time, the widely accepted estimate of the precolonial population of Greater Amazonia (approximately 6.7 million square kilometres)^11^ has increased from about 1.5–2 million^12^ to 5–10 million^5^, and the estimated regional population in the Amazonian southern rim has been established as 0.5–1 million through the presence of 1,000–1,500 earthworks^7^. Furthermore, the discovery of relatively densely populated regions in Amazonia has led to discussions about several types of South American urbanism^4,13–15^, a trend that has been reinforced by recent research using airborne light detection and ranging (LiDAR) data acquired by airborne laser scanning (ALS). The technique has been applied in Llanos de Mojos, Bolivia^16^ and the Upano Valley, Ecuador^17^, and it has provided important insights into the structure of local cultures. However, the LiDAR surveys have not yet covered any larger forest areas with justified estimations of their population sizes.

Our previous systematic studies of high-resolution satellite imagery and aerial photographs have revealed nearly 1,300 earthworks^3^ in an area of approximately 134,000 km^2^ in the northern part of Southwest Amazonia. Most of the earthworks are associated with the civilization that built ditched geometric enclosures (geoglyphs), forming a multicultural complex society designated here as the Aquiry^2,3,18^ (Supplementary Note 1a). The communities of this civilization shared a common sociocosmology, as reflected in the monumental earthworks featuring geometric patterns, which began to be constructed over 2,500 years ago^3,9,10^. Furthermore, radiocarbon analyses indicate that some earthworks in the area are much later mound villages and roundish embanked enclosures without ditches (post-ad 1000–1200)^3,10,19^.

**Extended Data Fig. 1:**
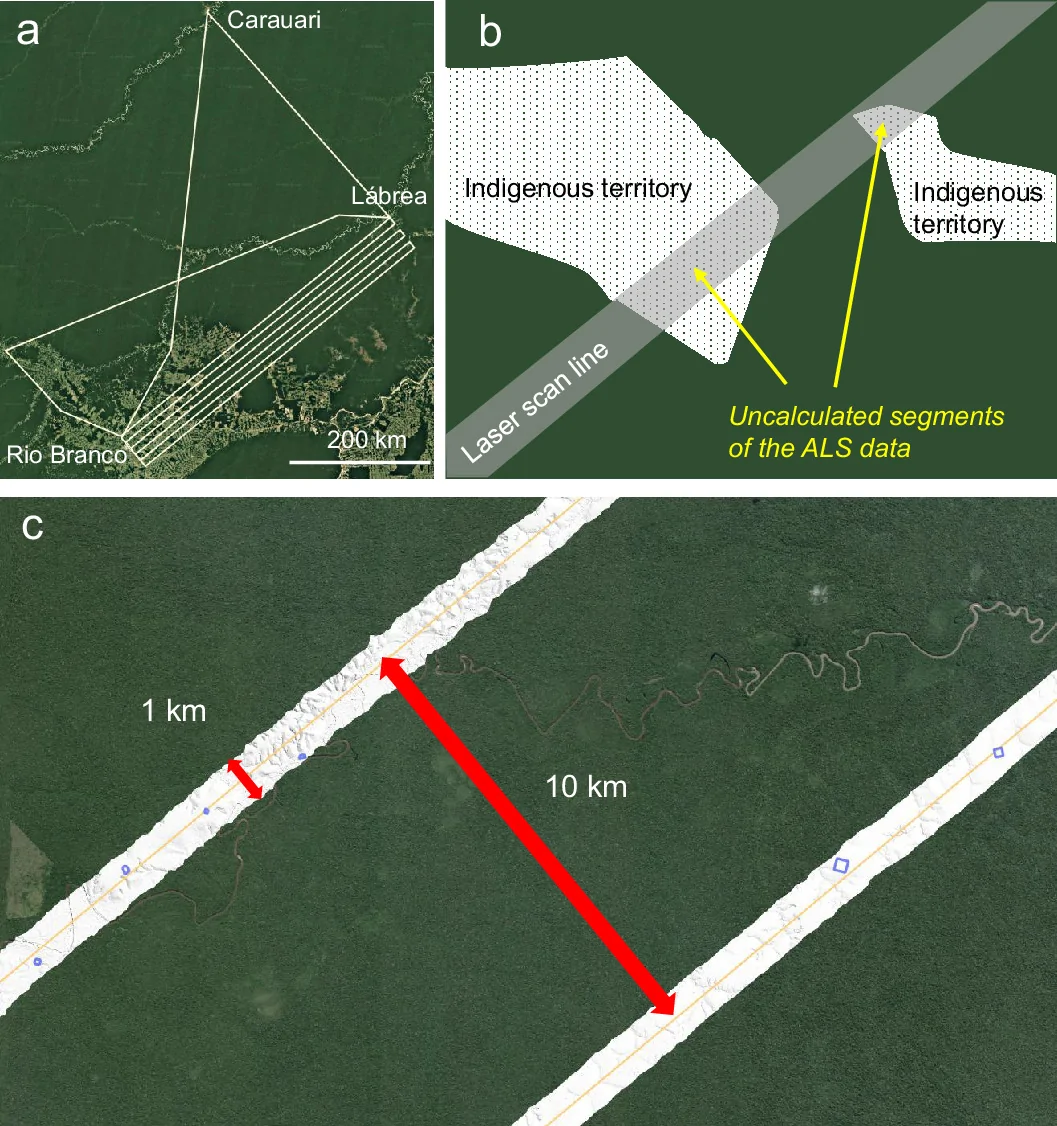
Lines for ALS. **a**. Routes of Lidar flights in the study area. **b**. Flight lines and Indigenous territories (white raster). **c**. Example of two parallel 1 km wide LiDAR data lines at a 10 km distance. The blue markings represent earthworks. Google Earth images in panels **a**,**b** are from Google Landsat/Copernicus. Google Earth images in panel **c** are from Airbus Landsat/Copernicus.

In 2024, we conducted ALS surveys over 4,430 km, covering 4,497 km^2^, in the Brazilian states of Acre and Amazonas to better delimit the geographical extension of this society. Our new LiDAR data, obtained from 10 ALS lines (Extended Data Fig. 1), document 432 earthworks, of which only 36 were previously known. By combining our previous results with the new LiDAR data, we are now able to determine the approximate geographical extension of the Aquiry civilization more accurately than before. In addition, we are now able to estimate the total number of earthworks and distinguish the Aquiry structures from later earthworks. This allows us to tentatively estimate the size of the population around 100–300 ad, when the civilization appears to have peaked.

## Earthworks and precolonial urbanity

**Fig. 2:**
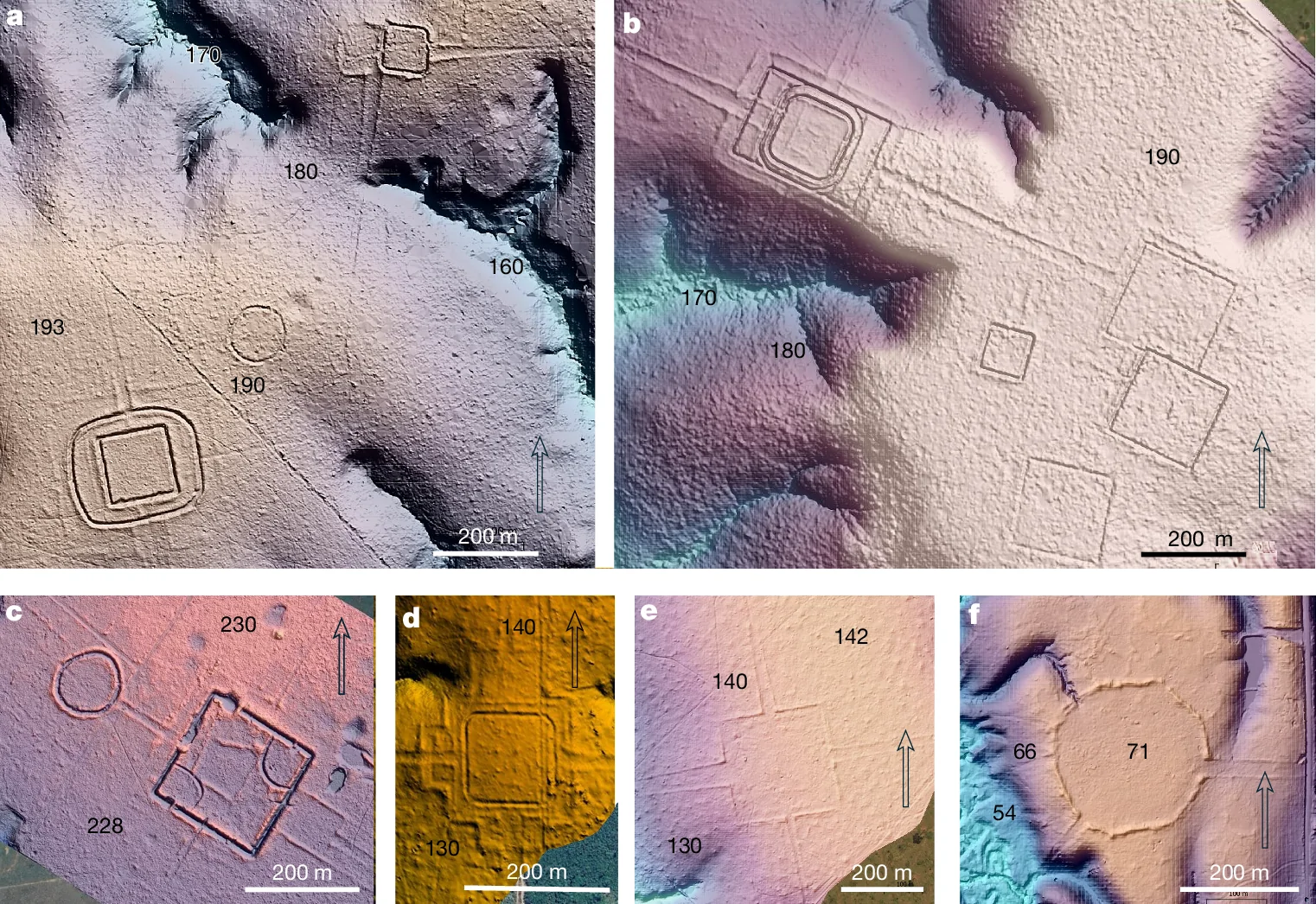
Diverse earthwork types with their topographical contexts. **a**, Piloto. **b**, D. Schaan. **c**, Fazenda Atlântica. **d**, amag34/Lid24_05_43a. **e**, Lid24_05_44a. **f**, Lid24_09_1b. Hillshading and colours are applied on digital terrain models; numbers in italics indicate relative elevation (metres above sea level) derived from those models.

**Extended Data Fig. 2:**
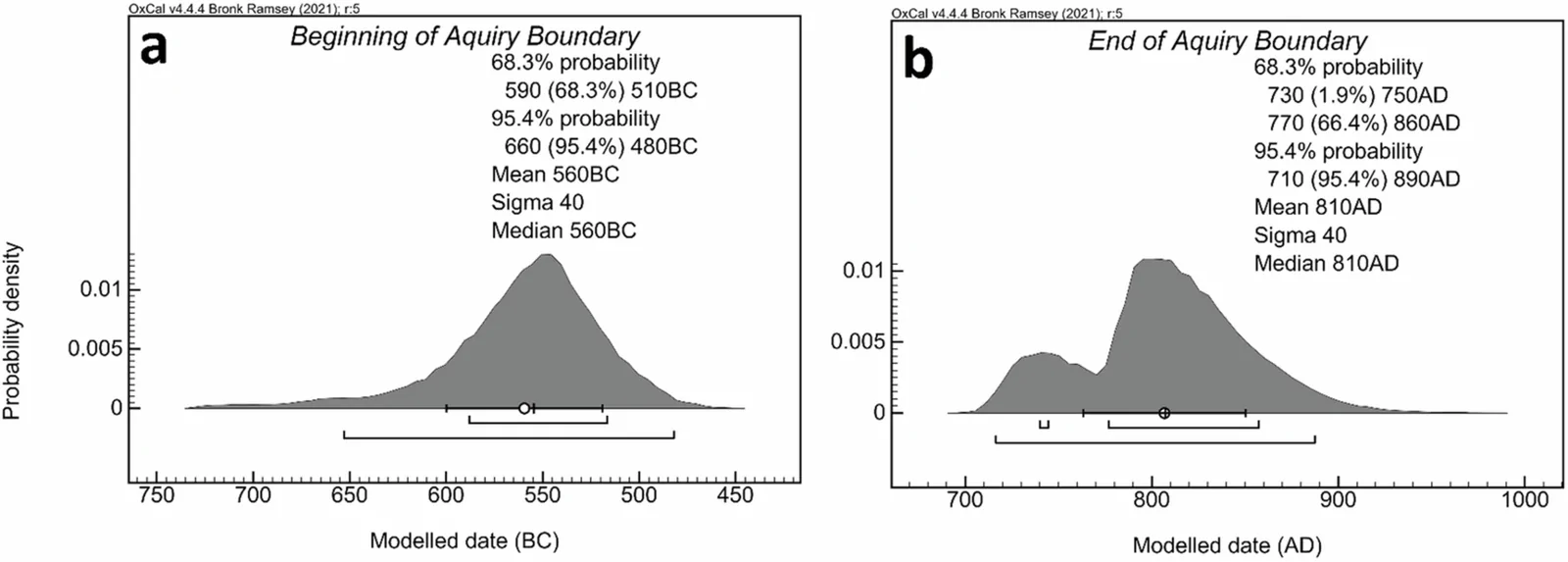
Modelled images of the Aquiry phase. **a**. beginning of the Aquiry phase**. b**. ending of the Aquiry phase.

**Extended Data Fig. 3:**
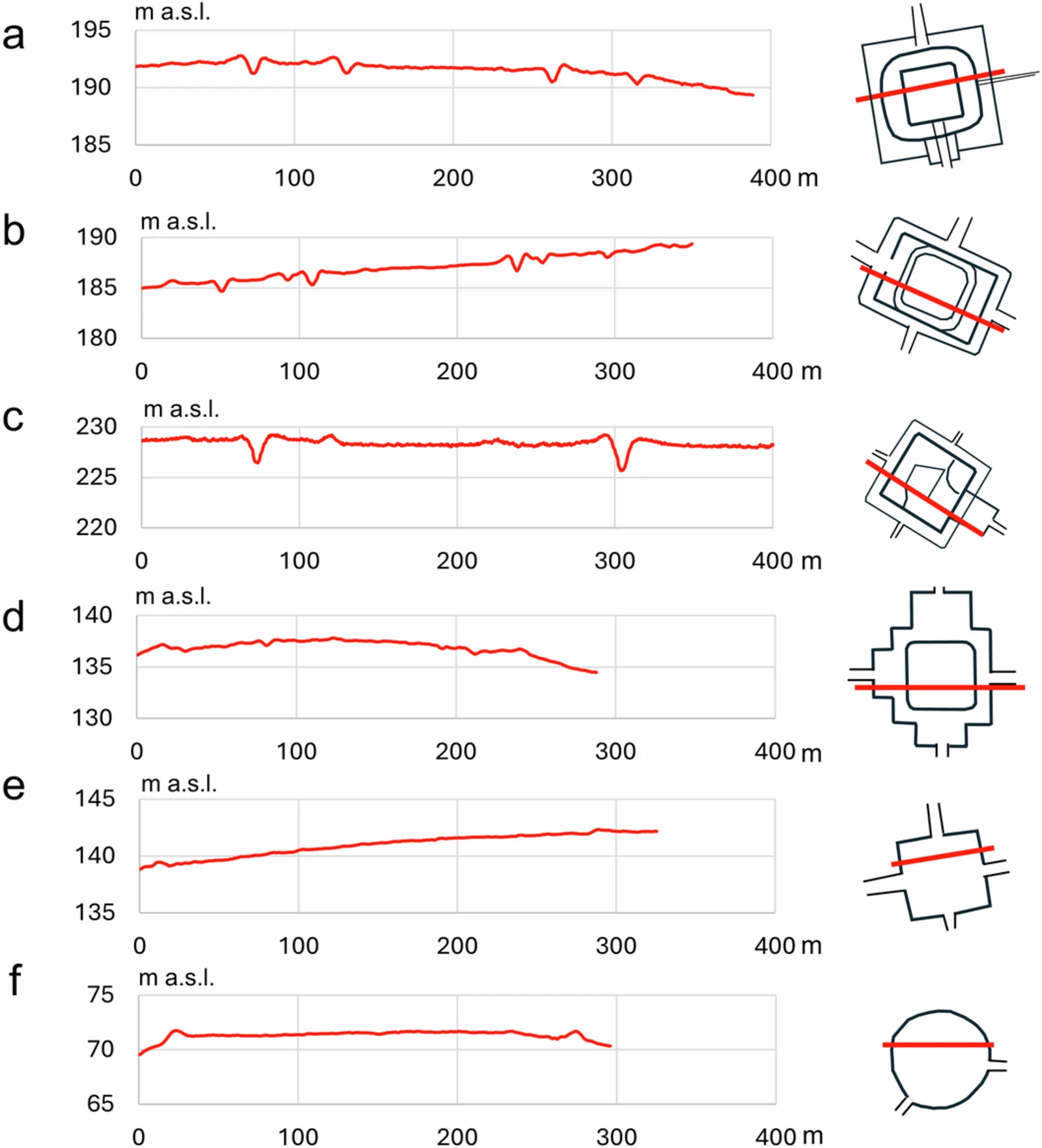
Topographic cross-sections of the Digital Terrain Models illustrated in Fig. 2a–f. The positioning of each cross-section is indicated by red lines on the maps drawn, with each line running from left to right.

**Extended Data Fig. 4:**
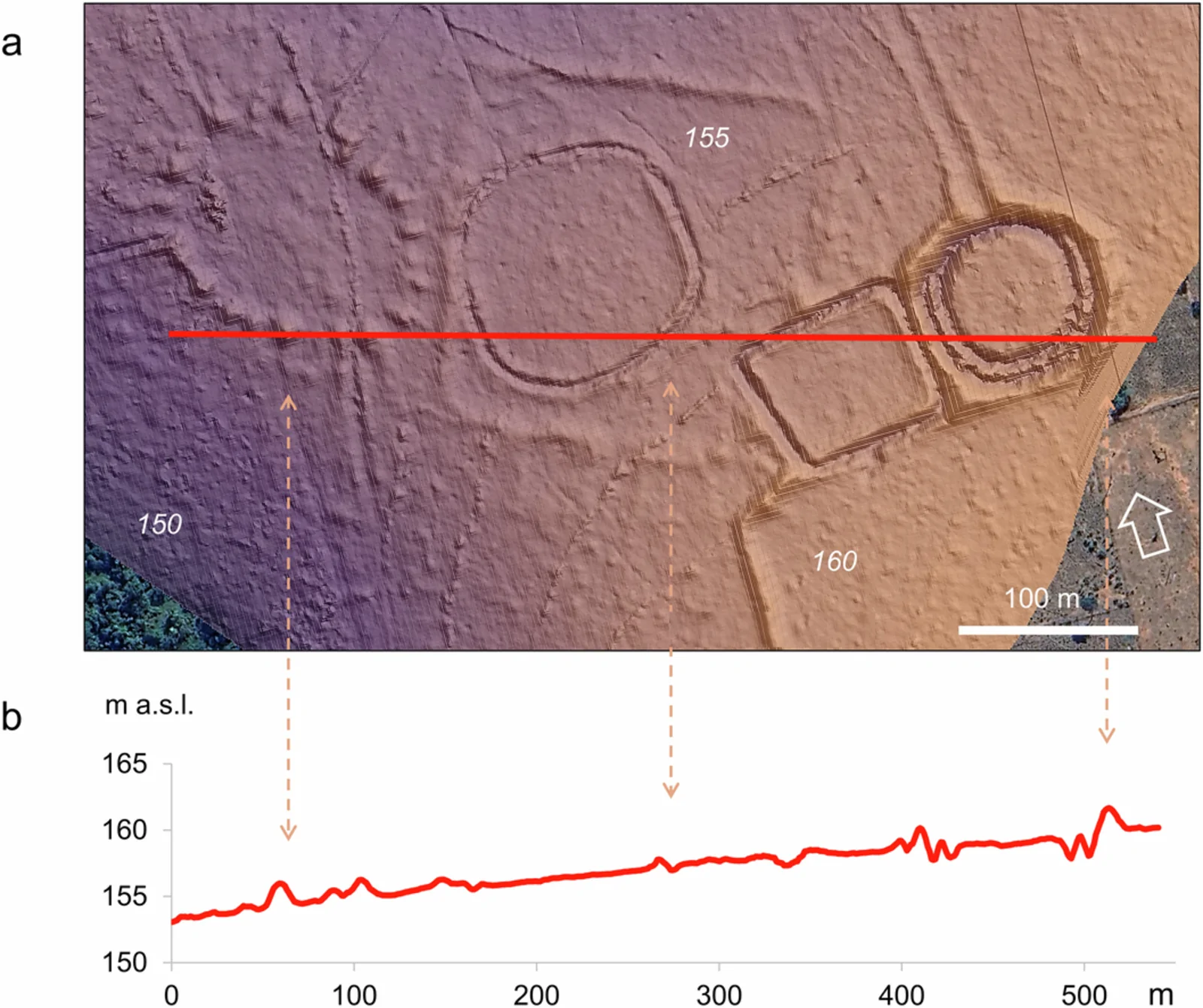
A group of earthworks on the Digital Terrain Model imagery. **a**. The DTM image shows the site (2g) Lid24_01_203 including a later mound settlement on the left. Hillshading has been applied to improve the visibility of structures; elevation variations are represented by colour gradients, and numbers in italics indicate metres above sea level. **b**. Cross-section of the earlier transect, indicated by a red line, with pink arrows between panels (a) and (b) to aid positioning of structures in both figures.

The size of the geoglyphs, constructed between 600 bc and ad 850 according to Bayesian modelling (Extended Data Fig. 2 and Supplementary Note 1b) with some regional variations in their forms, tends to vary between 0.35 and 14 hectares (ha). Nevertheless, the largest known geoglyph is nearly 50 ha (refs. ^20,21^). The embankments of the geoglyphs are typically outside or sometimes on both sides of the ditch^2,3^, and the depth of the excavated 9–13-m wide ditches reaches up to 5 m below the surface^2,3^. Most of the geoglyphs are solitary structures, with or without roads, but it is also common for two or more geoglyphs and embanked enclosures to form bigger clusters, up to eight enclosures (Fig. 2a–e and Extended Data Figs. 3 and 4). Furthermore, our LiDAR data reveal that embanked enclosures without ditches are much more common than expected, due to the difficulty of detecting them with satellite imagery. Solitary quadrilateral and multilateral embankments have not been dated earlier, but their geometric forms are associated with geoglyph structures—contrary to later roundish embankments—that we consider them to be part of the architecture of Aquiry and therefore contemporary with the ditched enclosures. In fact, using LiDAR data, it is easy to see that even many previously known geoglyphs, such as Piloto (in Fazenda Cipoal), D. Schaan, Fazenda Atlântica and ‘amag34’, have embanked quadrilateral and multilateral outer structures not previously seen in aerial photos or satellite imagery (Fig. 2a–d). All these structures seem to have served as public, ceremonial and political centres for invited visitors, without permanent occupation or defensive structures^6,9,10,19^. This kind of precolonial urbanity has also been recognized in the Andean Inka State, based on temporary gatherings of diverse groups invited by the Inka authorities^22–24^.

Building such monumental works in the rainforest must have required a large workforce, division of labour and an understanding of mathematics and geometry. It is also clear that the construction and maintenance of such centres for around 1,500 years must have had administrative, sociocultural and cosmological motivations, including diverse forms of arts, dance, music and sport^18,24,25^.

## Geographical extension of the geoglyphs

It has been stated that the highest probability of finding earthworks hidden under the canopy exists in southwestern Amazonia, from western Rondônia state to the cities of Cruzeiro do Sul (western Acre) and Eirunepé (Amazonas) on the upper Juruá River^11^ (Fig. 1). We agree with the high concentration of earthworks from the uppermost Madeira Basin in Rondônia via eastern Acre up to the upper Purus River, as the presence of geoglyph-type earthworks is well documented in this area^3^. However, we had serious doubts about the continuity of eastern Acrean geoglyph-type ditched enclosures to western Acre, because online satellite imagery of open areas visible on both sides of the road from Rio Branco to Cruzeiro do Sul did not reveal any earthworks after Manoel Urbano was passed further west^3^. Furthermore, after the discovery of the first earthworks in Acre in 1977, a PRONAPABA project found several archaeological sites in the upper Juruá; only one of these had a small circular embankment (Eirunepé), and at two sites a small mound was found, but no ditched structures^26^. Thus, the question of the density of several types of earthworks in this interfluve between the upper Purus and the upper Juruá became an essential part of our investigation, to predict the limits of the Aquiry civilization and estimate its total population. This is why we designed our two ALS lines from the city of Lábrea to Carauari and from Carauari to the city of Boca do Acre, totalling approximately 800 km and passing between the interfluve of the Purus and the Juruá (Extended Data Fig. 1a).

**Fig. 3:**
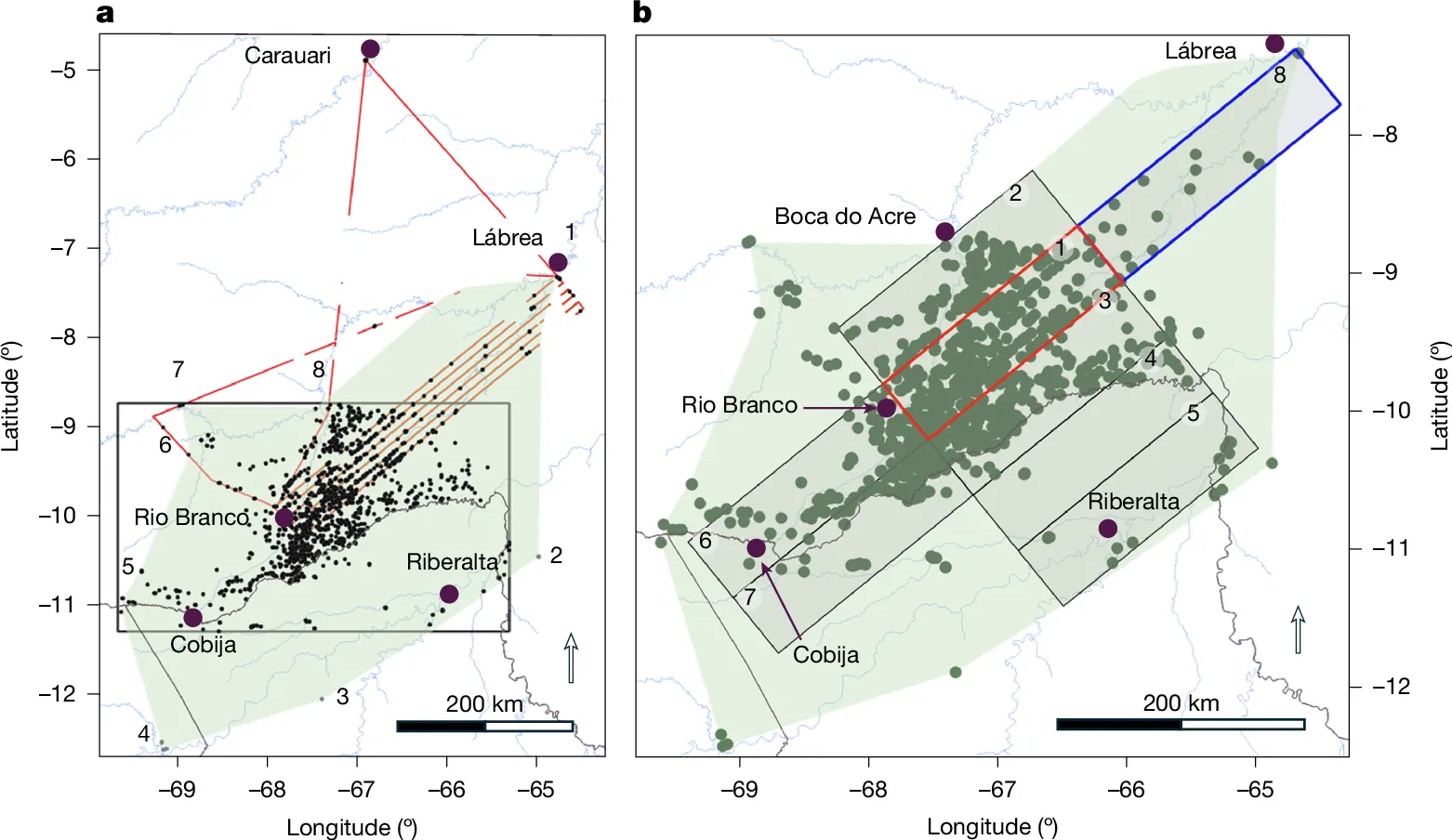
Area of the Aquiry. **a**, The area of geoglyphs and quadrilateral and multilateral embankments associated with the Aquiry are shown in green with their outermost edges: 1, Lid24_L6_223a; 2, Pic Sidney Girão; 3, MP15; 4, Puerto Maldonado; 5, maddo; 6, Lid24L10_32a; 7, Lid24_L8_18a; 8, puruq. The black points indicate all earthworks within the inventoried area (*n* = 1,649); the black rectangles indicate the areas surveyed by satellite imagery^3^; the red lines show ALS lines. **b**, Rectangles 1–8 indicate blocks with earthwork-number data: red outline (block 1), Rio Branco region; blue outline (block 8), low-density earthwork periphery. The green background is the same as that in **a**. The green dots (diameter of 10 km) indicate the Aquiry earthworks only (*n* = 1,413). Purple circles in **a** and **b** indicate the main present-day towns, to aid orientation. Data of rivers are from ref. ^50^.

No earthworks were detected by laser scanning in this upland area: either because the forest was too dense for the laser to produce an accurate terrain model, or because there were no earthworks on the flight lines. The latter option would lend support to the idea of ancient Amazonian buffer zones that were not permanently inhabited^27^. However, a large previously known circular earthwork with several smaller earthworks was confirmed on the banks of the Juruá River in Carauari (Fig. 2f). Similarly, no earthworks were detected in the approximately 200-km long ALS line passing through the interfluve north of the Purus, 40 km after Manoel Urbano to Ȏco do Mundo (Figs. 1 and 3a). After this, the line continued for approximately 300 km to the east, but only two geometrical ditched earthworks were observed on the northern side of the Purus River, not far from Lábrea. Because those are in contemporary Indigenous territories, we do not indicate their exact location.

To predict the full extension of the monumental ditched geoglyphs, we took into account that the fortified circular ditches in annually flooded Bolivian lowlands at Llanos de Mojos and Baures—as well as at the upper Xingu and the upper Tapajós in Brazil—are generally dated from between the thirteenth century and seventeenth century ad^28–30^, and therefore, they cannot be contemporary or directly related to the ceremonial Aquiry geoglyphs abandoned in the ninth century. Thus, we established the southern margins of the Aquiry geoglyphs between Pic Sidney Girão in Rondônia, Brazil, Tumichucua near the town of Riberalta in Bolivia, and Puerto Maldonado in Peru, based on where some nearby ditched earthworks are known and visible on satellite imagery (Fig. 3a,b).

The western and northern frontiers of the geoglyph-building society can now be established by the presence of ditched enclosures from Puerto Maldonado to the Purus River in Manoel Urbano and from there up to Lábrea in Amazonas state, following the presence of ditched enclosures near the Purus River (Fig. 3a). From Lábrea, the area seems to turn south back to Pic Sidney Girão in Rondônia. In summary, this geographical circumscription of the Aquiry civilization produced an area of about 183,000 km^2^.

The cultural relationship and dating of the remaining earthworks in Rondônia, located to the east and southeast of our established line, must be left as an open question due to the absence of archaeological studies of these recently mapped structures^31,32^.

## Number of earthworks in six ALS lines

Over the past two decades, the number of known earthworks in the study area has increased considerably, from the first published count of 60 earthworks^33^ to 200 earthworks^1^ and then 1,279 earthworks^3^. Earthworks are common around Rio Branco and surrounding areas to the north and southeast^3,34^. Although much of the Iquiry and Ituxi basins towards Lábrea in the state of Amazonas lie under the dense forest canopy, earthworks are potentially detectable by ALS. This led us to focus on this area using six parallel ALS lines, each approximately 445 km long, 1 km wide and 10 km apart, for a total of 2,380 km, as contemporary Indigenous territories below these lines are not included in our database.

We detected 347 structures of various earthwork categories, detailed in Extended Data Table 1a. Furthermore, it was calculated that 320 (92%) of these earthworks were located within 210 km of the start points in the Rio Branco region (block 1), whereas only 27 (8%) of the earthworks counted were detected at 235 km or less from the end points in Lábrea (block 8). Thus, the last 235 km can be described as an area with a low density of earthworks. Nevertheless, the forest around Lábrea is denser than that around Rio Branco^35^, and according to our sensitivity analysis, LiDAR penetration was about 10% lower there (Supplementary Note 1f). In general, of the area inspected west of the 66° W longitude (Fuso 19), we verified that 41.9% of the ground did not have sufficient point clouds to visualize ordinary earthworks. In Fuso 20, east of the 66° W longitude, this figure rises to 54.5%. However, geoglyphs covering more than 1 ha with deep ditches and high embankments were more visible. The total invisibility of even these features corresponds to 11.3% of the Fuso 19 area (including the Rio Branco region) and 17.8% of the Fuso 20 area (including the Lábrea region; Supplementary Note 1f).

## Factor five for satellite imagery

**Extended Data Fig. 5:**
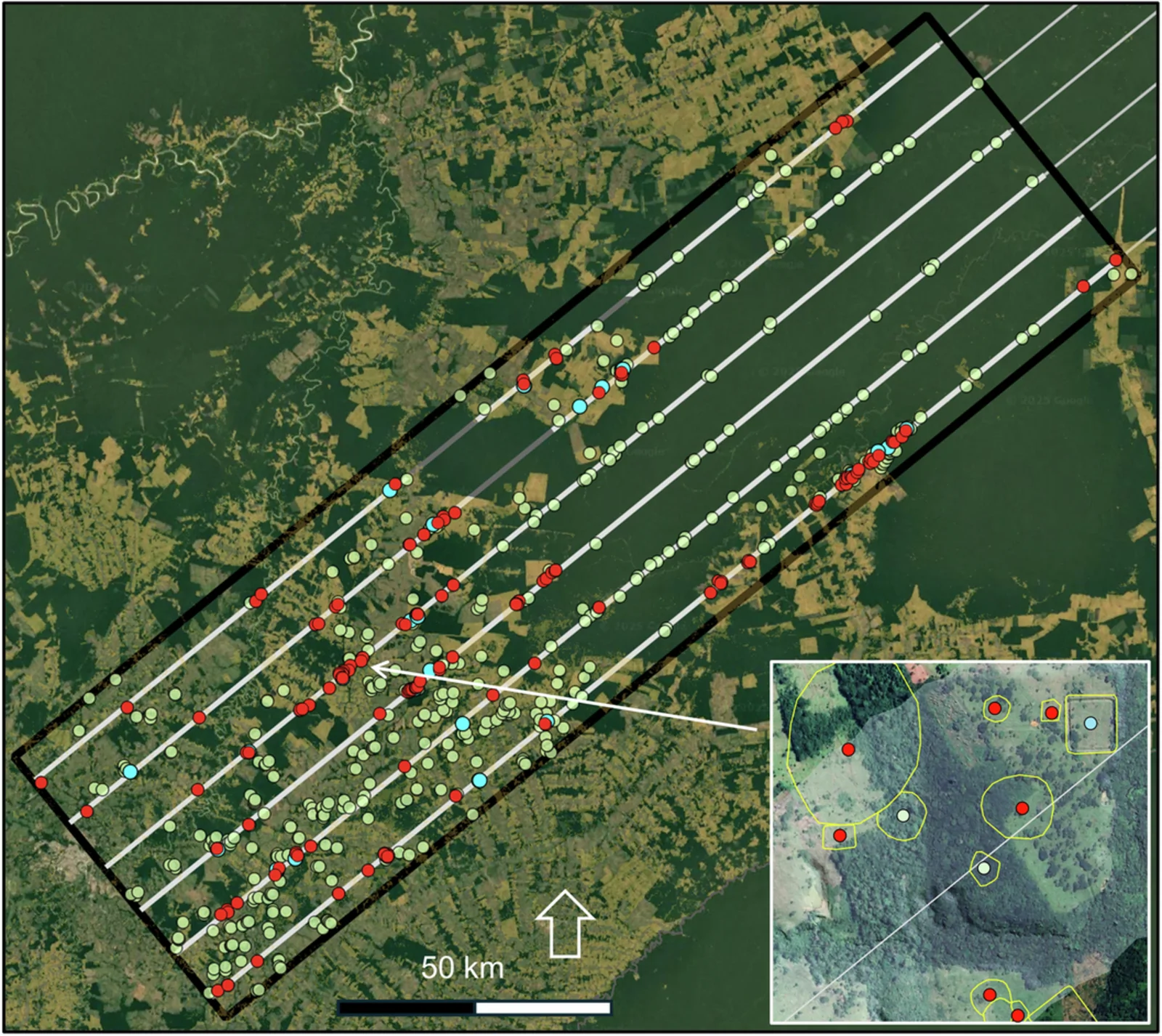
Earthwork detection using different data sources in Block 1. Blue points indicate structures in deforested areas with both satellite and Airborne Laser Scanning (ALS) coverage; red points represent structures detected only in ALS data in deforested areas. Light green points mark structures detected in only one dataset —ALS in forests or satellite imagery in deforested areas beyond ALS coverage. Due to the scale of this figure, there may be many earthworks under one point. Google Earth images are from Google Landsat/Copernicus and Maxar Technologies/Airbus.

The first important result of our analysis is that LiDAR scanning reveals five times more earthworks in the deforested areas of block 1 than we had previously found along the same lines using satellite imagery provided by Google Earth, Bing and ESRI^3^: we now found 156 earthworks where we had previously detected only 30 structures (Extended Data Fig. 5). The same factor is obtained when all ALS lines are considered. This means that the actual number of earthworks in the currently deforested areas of the region should be multiplied by five. In addition, using satellite imagery, we know that the currently almost untouched forest of northernmost Bolivia has only a few small deforested patches, but surprisingly many earthworks^3,21^. Thus, the relatively high-density area seems to extend as far as Tumichucua, the largest known geoglyph site situated near Riberalta. Also on the basis of satellite imagery, a relatively high-density area is located from the town of Rio Branco towards Boca do Acre, and finally, another relatively high-density area can be identified on the southwestern side of Rio Branco towards Cobija^34^ (Fig. 3a,b). If we now add six more sectors of a similar size to block 1 in these known relatively high-density areas between the towns of Boca do Acre, Riberalta and Cobija and multiply all previously detected structures of each block by satellite imagery (1,256 in total) by five, we arrive at 6,280 earthworks (Extended Data Table 2). Furthermore, if we assume that the forested areas of each block (77.5% in total) also contain monumental structures at a similar density to the deforested areas, we would have about 26,400 earthworks. Finally, if we assume that the rest of the study area (98,000 km^2^) belongs to a low-density periphery, possibly with 90% fewer earthworks, we would end up with 29,500 earthworks.

## Extrapolating the number of earthworks

Bearing in mind that the ALS captured 1-km-wide swaths 10 km apart, we can also extrapolate our LiDAR data as 10% of the block 1. Within this area, if we exclude 13 earthworks, more than 50% of whose areas lie outside the six LiDAR swaths, the total number of earthworks would be 3,070 (Extended Data Table 1c).

Hence, if we extrapolate the results of block 1, as an average, to all known relatively high-density areas of the Aquiry, this total area would cover 85,000 km^2^ and contain an estimated 21,490 earthworks (0.25 per square kilometre) in seven blocks (7 × 3,070). In addition, if we consider the rest of the area in question (98,000 km^2^) to be a low-density earthwork periphery like block 8 (0.02 per square kilometre; 8.41 × 270), we end up with an estimated total of 23,760 earthworks.

As a result, the overall difference between the extrapolation of LiDAR and satellite data is 23% among the seven core blocks, despite the variation in satellite imagery ranging from −52% (block 5) to +159.5% (block 7), compared with an average of 3,070 (Extended Data Table 2). However, because approximately 11.5–41.9% of the core area was determined to be impenetrable by laser, this suggests that the actual number could, indeed, potentially be near 30,000, as predicted by our extrapolation based on satellite-observed earthworks in each block.

In any case, even the most cautious estimation would be close to the figure recently predicted as the maximum for the whole of Amazonia^11^ and a factor of about 20 times higher than that predicted for the entire southern rim of Amazonia^7^. At the same time, our results would theoretically increase the total population estimate of the southern rim up to 6 million or more, based on the calculation of 500–600 people per earthwork derived from an ethnographically based linear regression model created for Lowland South America^7,36^. However, such an estimate would require a more detailed approach, as outlined below.

## Estimation of the population

The first estimate of the population in the geoglyph area in 2009 was calculated by the workforce required to remove the earth for the ditches and embankments in reasonable time and maintain those various generations^1^. At that time, the number of earthworks was estimated at 200, and it was concluded that a population of at least 300 people would be required for an average geoglyph-type structure. Hence, the original population estimate was 60,000 people^1^, with a maximum density of up to 1.5 people per square kilometre^34^. Instead of 200 structures, our new data radically predict approximately 24,000–30,000 earthworks, of which approximately two thirds belonged to the Aquiry period, as will be seen later.

**Extended Data Fig. 6:**
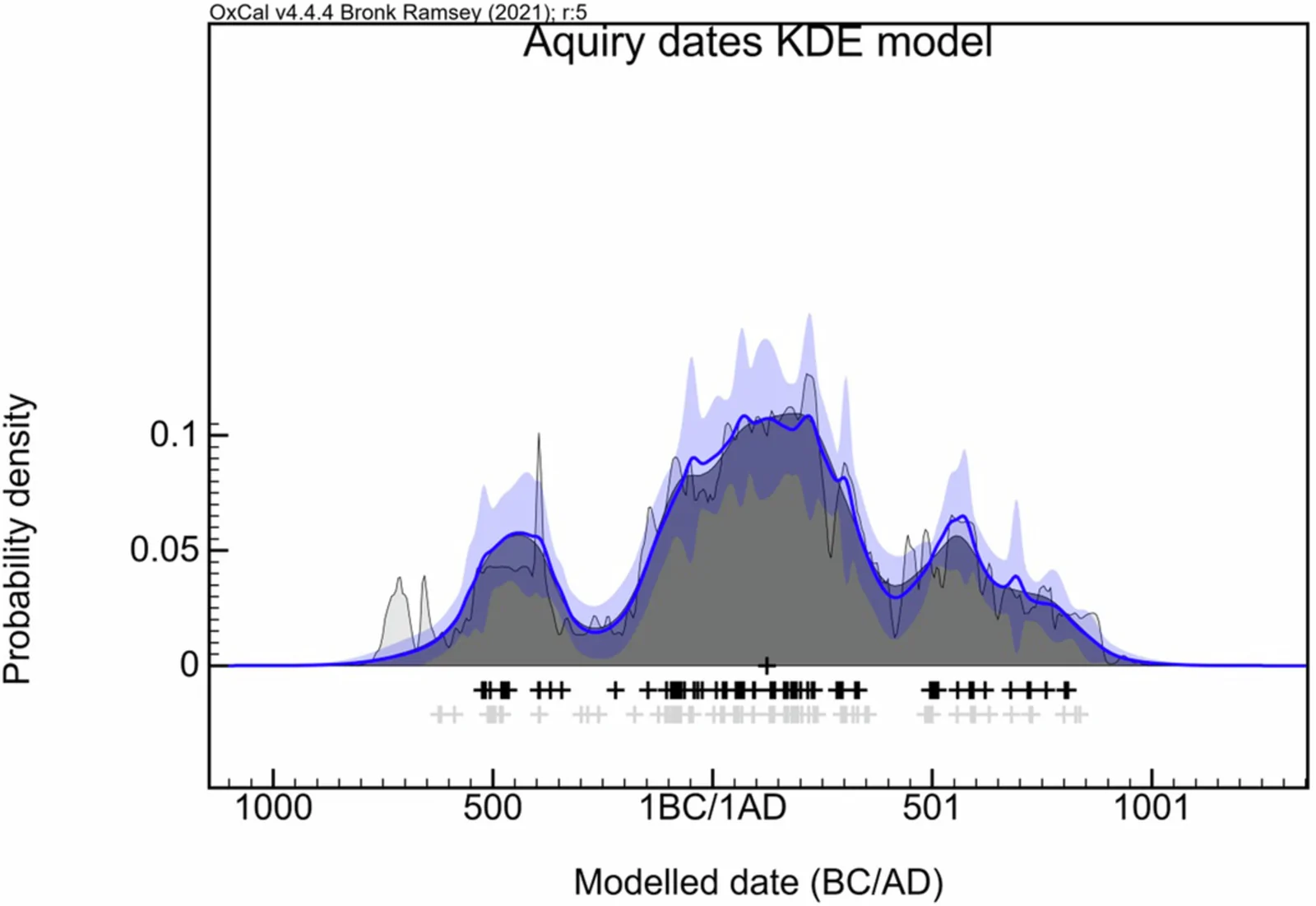
Kernel density estimates (KDE) on Aquiry geoglyph dates. Light grey crosses: median values of original calendar-year probability distributions (cpd); Black crosses: median values of posterior cpds; Light grey: sum distribution of the original dates; Dark grey: KDE model result; Blue line and light blue bands: mean ± 1sigma of the KDE distributions drawn from the modelling indicating the result significance.

**Extended Data Fig. 7:**
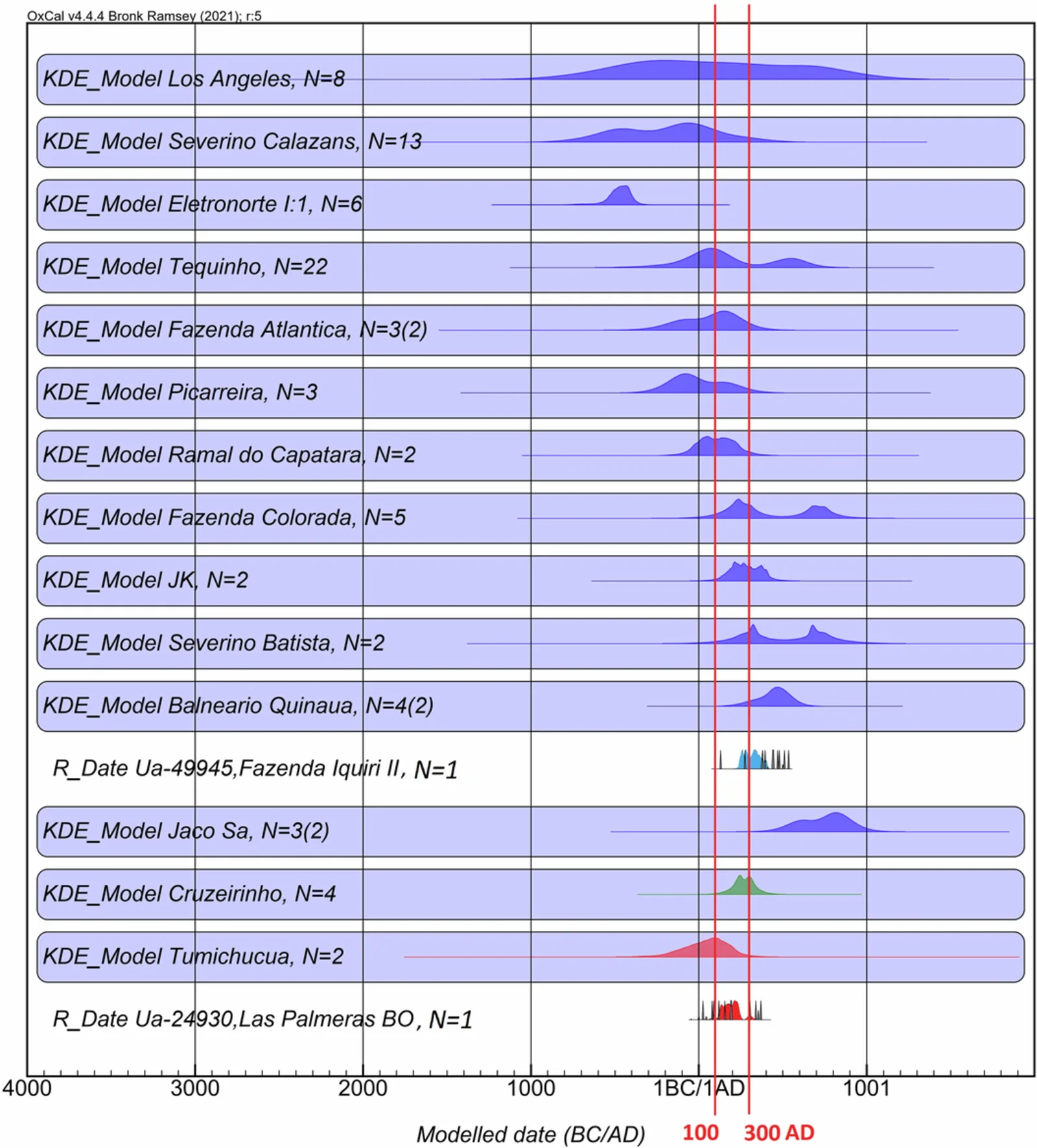
Overlapping KDE summaries of phase models combining radiocarbon dates of ditched geoglyphs associated with three subregions. Dates in Acre are blue, in Amazonas green and in Bolivia red. N shows the number of dates, and number of earthworks has been given in parentheses.

**Extended Data Fig. 8:**
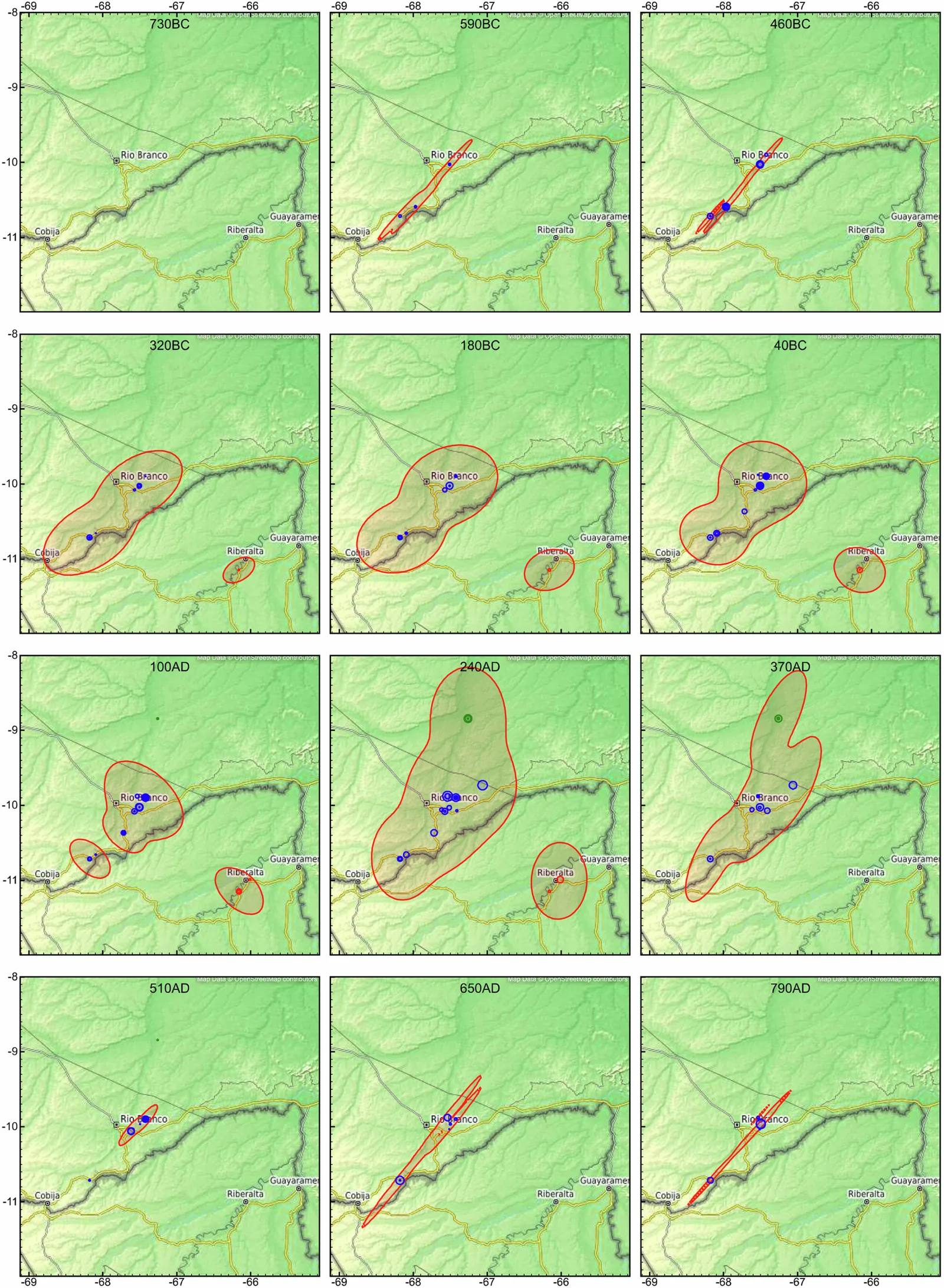
A series of maps showing the temporal and geographical development of dated Aquiry sites. Blue points refer to Acre, red to Bolivia and green to Amazonas sub-regions. The maps produced through OxCal 4.4.4 under the Open Database License (https://www.openstreetmap.org/copyright).

To calculate the population of the Aquiry, the first obvious question is regarding how many earthworks were in use at the same time. The densest overlap of calibrated dates occurs at around 100–300 ad, both chronologically and spatially (Extended Data Figs. 6–8). According to Kernel density estimates, 25% of all Aquiry earthworks were constructed during this period, with an average of 22 sites per year (Extended Data Fig. 6). However, when analysing the probability of the simultaneous use of all dated Aquiry earthworks, about 60% overlapping occurs approximately 100–300 ad in summarized Kernel density estimate phase modelling (Extended Data Fig. 7). Furthermore, when the average sizes of the earthworks were compared using satellite imagery, it appeared that the geoglyphs in Amazonas (block 2) and Bolivia (block 5) are, on average, 58% and 77% larger than those in Acre (block 1), respectively (Supplementary Note 1c).

Of the total of 334 earthworks inside our six parallel LiDAR lines, we have identified 120 ditched geoglyphs and 115 embanked quadrilateral or multilateral enclosures that all can be associated, as said, to the Aquiry civilization (Extended Data Table 1b). The average exterior size of all these earthworks is 2.98 ha (Supplementary Table 2). The rest of the earthworks are considered to be later post-1,000 ad structures. Of the Aquiry earthworks, 220 are in block 1, representing 10% of the block area. Thus, we can estimate that there are approximately 2,200 Aquiry earthworks in the entire block 1 (Extended Data Table 1c), and if we extrapolate this as an average to the other six relatively high-density blocks (2–7), we would have 15,400 ditched geoglyphs and quadrilateral and multilateral embankments in this area. Furthermore, if we consider that we recorded 15 geoglyphs and quadrilateral and multilateral embankments (0.01 per square kilometre) in the low-density block 8 and applied the same density to the rest of the Aquiry area (98,000 km^2^), we should add 1,262 earthworks to the previous figure, making a total of 16,660. If, as said, about 25–60% (4,165–10,000) of these earthworks were in use in 100–300 ad, and if an average earthwork required 300 people to build and maintain it, we arrive at a total population of about 1.25–3 million. In addition, we should bear in mind that our estimate, based on actual satellite discoveries in deforested areas, suggests a 23% higher number of earthworks. Furthermore, the forest canopy significantly hinders the detection of earthworks in block 1 (11.5–41.9%, mean 26.7%) and block 8 (17.8–54.5%, mean 36.2%) (where percentages indicate the proportion of earthworks that may remain undetected), even when they are larger in size, at least in block 2 (Amazonas) and block 5 (Bolivia). Therefore, this calculation method suggests that the actual number could be as high as 1.6–3.8 million.

## The Aquiry enclosures versus later villages

Another way to estimate the number of people in the precolonial Aquiry is to reason about how many people lived in an average mound village when these settlements appeared in the very same area in the early thirteenth century. These new village formations located near earlier ceremonial centres in the Aquiry core area (Extended Data Fig. 4a), and, according to our understanding, these people used similar domestic ceramics and had similar subsistence strategies, including maize cultivation^37^. As there are no signs of radical population replacement, these villages may offer the best indication of the size of the dispersed communities that chose to live together. However, direct comparison of the spatial size of settlements and geoglyphs is not recommended because of differences in their functions^36^.

Here we used the data collected by Iriarte et al.^19^ from roundish and rectangular mound villages in the Aquiry core area, described in Supplementary Note 1d. The average size of these settlements is 1.28 ha with 11.75 elliptical mounds. In general, mounds are much bigger than 100 m^2^ and can be classified as multi-family maloca longhouses with approximately five or six families and 25 people at a minimum^36,38^ (Supplementary Note 1d). Hence, we arrived at 235 people per hectare and 300 people per average mound village. However, if we assume that the original houses of all mound villages were not multi-family maloca longhouses but homes for single families, we would arrive at approximately 60 people per village. Nevertheless, even if this last option were possible, it is extremely unlikely (Supplementary Note 1d). For some, even 300 people per community may sound modest for an urban-like centre, but it is worth remembering as an example that in fifteenth century Germany, there were only 3,000 cities and towns. The population of 2,800 of these towns, with their public market squares, churches and public buildings, varied between 100 and 1,000 people^39^, which is at the limit of our numbers in southwestern Amazonia.

In every case, when comparing the possible population size of the later villages with communities that maintained earlier ceremonial and political centres, rather than the size of the structures, there is a match with our estimation of approximately 300 people per average earthwork and approximately 1.25–3 million people or more at the peak of the Aquiry. Only if the very unlikely estimation that each approximately 25-m long house foundation (mound) belonged to a single family were true would the population be about 250,000–600,000 people without the invisibility effect of laser penetration, which would raise the population, in this case, to approximately 315,000–760,000.

Finally, even though archaeological evidence testifies about important cultural changes after geoglyphs were abandoned, we should not underestimate the phenomenon of long duration either. For example, in Southwest Amazonia, many Indigenous nations held ceremonies for various purposes with 200–400 people, such as the kyynyry encounters of the Apurinã nation who live in the centre of the earthwork area. Depending on the purpose, an open space or several open spaces of 1–2 ha, as well as ceremonial roads, are created for the distinct phases of the ceremony^24^. Our Apurinã collaborators are aware of the past use of these ceremonial earthworks and how many people each geoglyph was built for, which is in line with the estimation presented here.

## Discussion and conclusion

Today, we know that soil and nutritional conditions in the Amazonian forest are highly heterogeneous. In Acre, several local interlocutors have confirmed that the soil is fertile and suitable for maize cultivation, for example. This has currently been confirmed by a base cation concentration map, which shows that the soil in the Aquiry area is among the most nutrient rich in the entire terra firme forest of Amazonia^40^. Furthermore, the correspondence between semi-domesticated and domesticated trees and the presence of Amazonian earthworks has been demonstrated^8,11^. Brazil nut trees were among them, and our study suggests that its current western extension to the Purus River^41–43^ corresponds to the western limit of the geometric ditched enclosures. The macrofossils of the Brazil nut and the fruits of diverse palms, such as the bacaba (*Oenacarpus mapora*), peach palm (*Bactris gasipaes* var. *gasipaes* and var. *chichagui*), uricuri (*Attalea phalerata*) and some *Astrocaryum* species have been detected in excavations as important sources of protein for the local population, and those tree species are still present around earthworks^8^. The variety of crops cultivated in the Aquiry area is also impressive, as detailed in Spanish accounts from the sixteenth and early seventeenth centuries^8^. In addition, phytolith studies have revealed the ancient presence of maize (*Zea mays*) and squash (*Cucurbita* sp.) in the geoglyph sites^37^. Furthermore, manioc, sweet potato, chilli pepper, beans and peanuts were also probably among the most common cultigens^37^. Therefore, poor soil and carrying capacity do not appear to be major limiting factors for population density in this area, as has been suggested for Amazonia as a whole^12^. On the contrary, the members of the geoglyph-building communities were active stewards of the land, water and forest gardens, and the construction and management of the sites were clearly linked to sociocosmology and social life entangled with more-than-human beings^24^.

To summarize, by using airborne LiDAR technology, it has been possible to triple the area of the Aquiry civilization from the earlier 60,000 km^2^ (ref. ^1^) to approximately 183,000 km^2^. Although the southern, western and northern boundaries of the Aquiry are well documented by the presence of geometric ditched enclosures, the question of its southeastern corner remains open due to the lack of archaeological research on earthworks in Rondônia. The presence of 347 total identified earthworks, with 334 of those earthworks used for extrapolation, in the systematically scanned area of 2,380 km^2^ is much higher than expected. According to our estimation, the entire area would have approximately 24,000–30,000 earthworks, of which about two thirds belonged to the Aquiry.

Today, the state of Acre covers 164,000 km^2^ with a population of 0.83 million (5.1 people per square kilometre), of which about 3.5% are Indigenous^44^. Although different methods and datasets may yield somewhat different conclusions, our results strongly suggest that between ad 100 and 300 the population of the Aquiry was approximately 1.25–3 million (7.1–16.5 per square kilometre), based on LiDAR data, earlier satellite imagery, ethnographic information and archaeological excavations. Furthermore, population density in the 85,000 km^2^ core area may have reached 13.6–32.6 per square kilometre.

It seems that periodic burning of the same bamboo-rich patches over thousands of years^45,46^ maintained the fertility of the soil and helped to create the conditions for the emergence of complex societies in the first millennium bc, comparable with other great South American civilizations. Eventually, the density of earthwork openings in the forested landscape became such that the concept of forest urbanism^47^, or low-density urbanism^13,48^, can be adequately applied, despite the lack of consensus on the meanings attached to the term ‘urbanism’^49^. At the same time, different social and linguistic groups were involved in managing, planting and maintaining their subsistence systems as well as participating in hunting, fishing and gathering activities. However, unlike many other tropical civilizations^16^, it appears that large-scale water management projects with raised fields and causeways were not a major focus of their subsistence strategies in extensive interfluve environments, although the presence of water sources appears to be one of the key elements for the earthwork sites and their roads^3^. In any case, these ancient achievements constitute an important legacy that is still observable in the soil, and the composition and biodiversity of the contemporary tropical forest. This legacy should be fully considered in future studies of Amazonia, including climate modelling and sustainable land-use planning. Furthermore, if our findings are confirmed and comparable results are obtained in other regions of the wider Amazonian area, the total precolonial population density should also be re-evaluated.

## Methods

### LiDAR data

This study was based on an airborne LiDAR survey in the Brazilian states of Amazonas (AM) and Acre (AC), reaching the municipalities of Carauari (AM), Lábrea (AM), Pauini (AM), Boca do Acre (AM), Manuel Urbano (AC), Sena Madureira (AC), Bujari (AC), Porto Acre (AC), Rio Branco (AC) and Senador Guiomard (AC), carried out between 6 June 2024 and 1 July 2024. The study area consists mainly of dense tropical rainforest, followed by some pasture and agricultural land. The flight mission included 1-km wide strips covering a total area of 4,497 km^2^, as shown in the flight plan in Extended Data Fig. 1a. In our analysis, earthworks discovered in Indigenous territories are not marked on our maps or database.

A V-Line Airborne RIEGL LMS-Q780 II full waveform laser scanner with Applanix POSAV 610 IMU mounted on a Seneca V aircraft was used for the LiDAR scanning. The RIEGL LMS-Q780 II is a long-range time-of-flight laser scanning system that supports full waveform analysis and multiple time-around processing. These features are advantageous for aerial surveys in complex canopied environments, such as dense forests. They increase the number of ground returns and improve the quality of digital surface models and digital terrain models (DTMs). Earthwork detection was performed from the DTMs. The flight altitude was approximately 1,125 m above ground (variation 1,090–1,250 m), and the flight speed was 130 knots with a laser pulse operating frequency of 1,600 kHz. The average LiDAR point density was 31.2 points per square metre, with a minimum density of 15 points per square metre and a maximum density of 60 points per square metre. Full waveform laser data processing was performed using RiProcess software (RIEGL). The GNSS–IMU (global navigation satellite system–inertial measurement unit) data were integrated into the pre-processing, to generate a georeferenced laser point cloud (UTM (Universal Transverse Mercator) 19 S/ SIRGAS (Geocentric Reference System for the Americas) 2000 (EPSG (European Petroleum Survey Group) 31979) and UTM 20 S/ SIRGAS 2000 (EPSG 31980)). In addition, the georeferenced point clouds were classified according to the LiDAR return of ground and non-ground points, allowing the generation of DTMs and digital surface models. Point cloud classification was performed using TerraScan software (TerraSolid).

The DTMs were generated using LASTools functions (RapidLasso) within a Python-based batch-processing pipeline. This pipeline performed ground-point classification, noise removal and data interpolation to the target resolution of 50 cm, which proved sufficient for geoglyph detection. The generated DTMs were then converted to hillshaded GeoTIFFs and a combined virtual raster using the Geospatial Data Abstraction Library^51^ for further spatial analysis using QGIS and a visual atlas shaded by Global Mapper. In addition, in June 2024, we used two drones in some easily accessible earthwork sites to provide more accurate mapping of areas that were only partially under the forest. One drone used a Zenmuse L2 system and the other an AlphaAir 10 system. Both had integrated LiDAR and RGB sensors and produced high-precision images that were analysed using the same QGIS and Global Mapper software.

Finally, the accuracy of detecting earthworks using our airborne LiDAR point clouds was tested by estimating visually the required point density for reliable detection within the scanned area. This sensitivity analysis and detailed description of the methods used in the ALS data processing and modelling can be found in the Supplementary Note 1e,f.

### Radiocarbon dates

To understand the chronological aspects of earthwork building activities, we have presented 162 radiocarbon dates of 32 earthwork sites and their 36 structures. All the presented radiocarbon dates were obtained using accelerator mass spectrometry. However, dates published in previous papers have used different calibration programs and curves. Therefore, to ensure comparability of new dates with previous dates, all dates have been calibrated to two sigma (95.4%) probability using the OxCal v4.4.4 program^52^ and currently recommended^53^ mixed (50/50) SHCal20 curve for the Southern Hemisphere^54^ and IntCal20 curve for the Northern Hemisphere^55^. Also, Bayesian modelling and Kernel density estimate phase models have been used. For a full description of the radiocarbon chronology, see the Supplementary Note 1b.

### Estimation of the number of earthworks and total population

First, we defined the area of the Aquiry civilization by the presence of geoglyph-type ditched enclosures using a combination of satellite imagery, aerial photography, the results of previous fieldwork and new LiDAR data. Our estimate of the number of earthworks in the area is based on a comparative study of satellite imagery and LiDAR DTM data from a systematically scanned area of 2,380 km^2^ (blocks 1 and 8). Considering the earlier satellite discoveries^3^, as well as the relatively high-density core and low-density periphery, the results are extrapolated to the entire area. However, the tropical forest can sometimes be too dense for the laser to reach the ground. Therefore, the total visibility of earthworks is also calculated using sensitivity analysis (Supplementary Note 1f), and the results have been incorporated into the discussion.

The total population was first estimated by approximating the labour required to build and maintain all the earthworks that were actively used in around 200 ad. Our estimate also compared the ceremonial earthworks with later precolonial mound settlements and their population densities, as well as with ethnographic documentation of livelihoods and the space required for ceremonies among contemporary Arawak-speaking and Panoan-speaking Indigenous populations in the same region.

### Reporting summary

Further information on research design is available in the Nature Portfolio Reporting Summary linked to this article.

## Online content

Any methods, additional references, Nature Portfolio reporting summaries, source data, extended data, supplementary information, acknowledgements, peer review information; details of author contributions and competing interests; and statements of data and code availability are available at https://doi.org/10.1038/s41586-026-10835-7.

## Supplementary information

Supplementary Information This file contains supplementary notes, supplementary tables, supplementary figures and supplementary references

Reporting Summary

Peer Review File

## Figures and Tables

**Extended Data Table 1:**
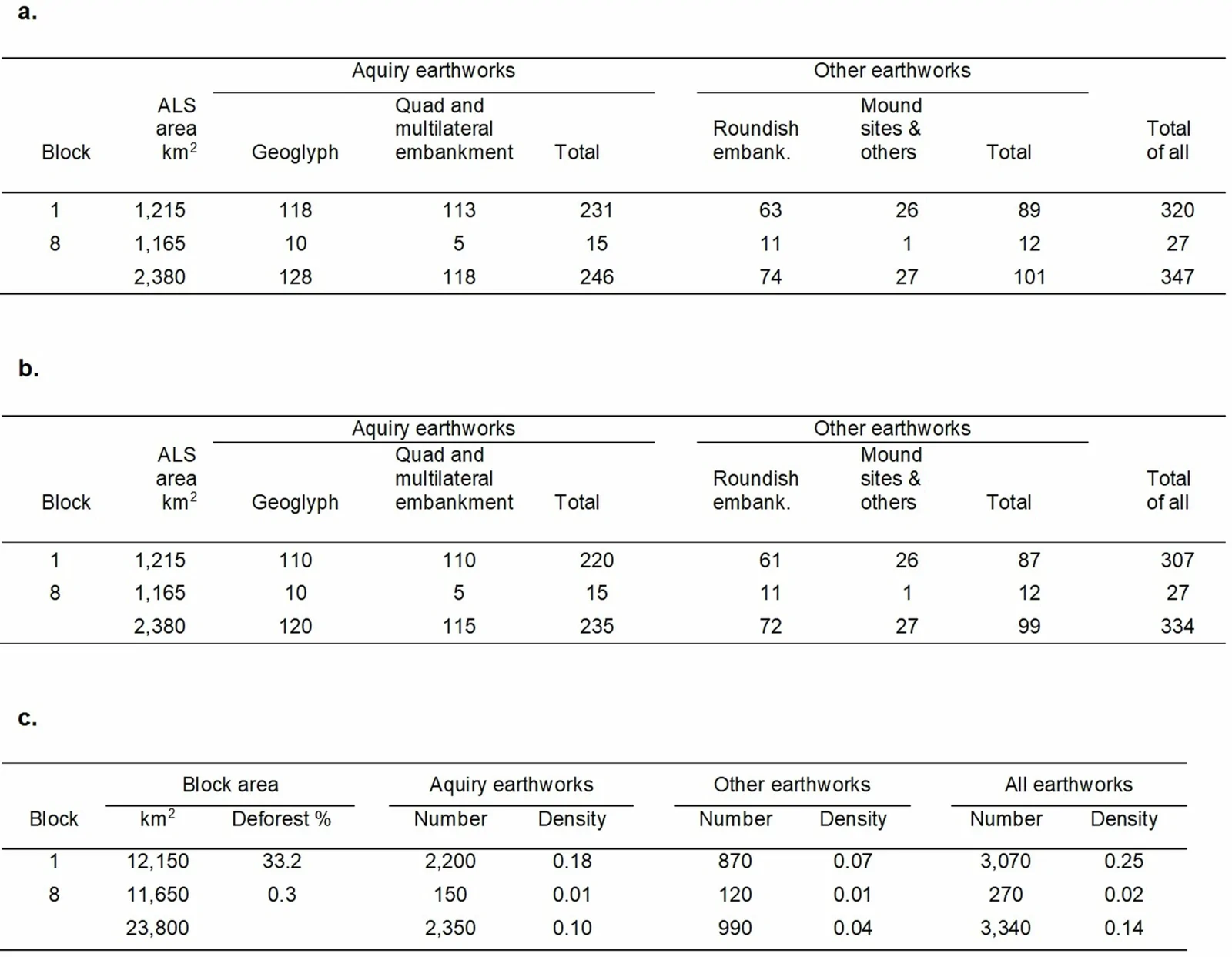
Area and number of earthworks in LiDAR data of the Blocks 1 and 8

**a**. Identified earthworks. **b**. Identified earthworks excluding structures that only partially (< 50%) intersect the LiDAR swath. **c**. Earthworks extrapolated to the entirety of Blocks 1 and 8 (×10). Indigenous territories are excluded, and only ceremonial and habitation sites are considered; wells, roads, causeways, and agricultural fields are omitted.

**Extended Data Table 2:**
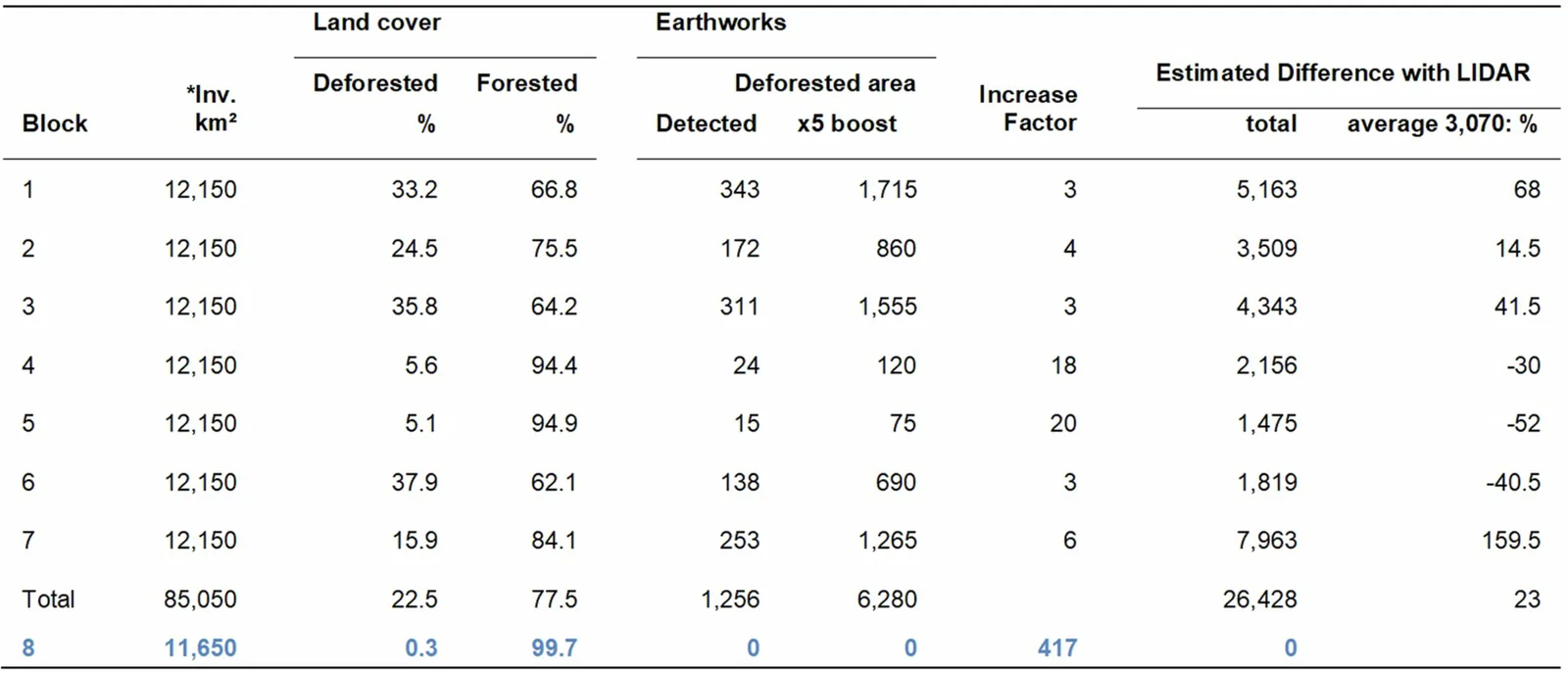
Blocks 1–8: Earthworks detected and extrapolated from satellite imagery

Inventoried area (*Inv. km²) of the blocks 1 and 8 excludes Indigenous territories overflown by LiDAR. The table shows the proportion of deforested and forested areas within each block^56^. The number of earthworks detected is based on a visual inspection of satellite imagery in the deforested landscape^3^. The x5 boost is due to the fivefold increase in the efficiency of LIDAR to detect earthworks in deforested landscapes. The Increase Factor assumes that both forested lands and deforested areas within the block contain equally dense earthwork settings. The last section shows the difference with an estimated average of 3,070, extrapolated from the LiDAR data. Relatively high density blocks of earthworks 1–7 are shown in black, and low-density block 8 is shown in blue.

## Data Availability

All relevant primary data have been provided with the article and its Supplementary Information. The complete datasets used to calibrate all radiocarbon dates are available in Supplementary Table 1a,b.

## Code availability

The original LiDAR raw data are restricted under Brazilian law. No custom code was developed. Details on the availability of background data used in the figures and tables are provided in the ‘Data’ section of the Reporting Summary.
