# Supplementary material for: Over 20,000 precolonial earthworks in the Southwest Amazonia
Source: Nature. 2026 Jul 29;657(8131):420–5. doi: 10.1038/s41586-026-10835-7 (PMC13558069; doi:10.1038/s41586-026-10835-7)
Supplement: Supplementary file 1 [file 41586_2026_10835_MOESM2_ESM.pdf]

Corresponding author(s): Martti Pärssinen

Last updated by author(s): May 31, 2026

Reporting Summary

Nature Portfolio wishes to improve the reproducibility of the work that we publish. This form provides structure for consistency and transparency in reporting. For further information on Nature Portfolio policies, see our Editorial Policies and the Editorial Policy Checklist.

Statistics

For all statistical analyses, confirm that the following items are present in the figure legend, table legend, main text, or Methods section.

n/a	Confirmed	
☐	☒	The exact sample size (*n*) for each experimental group/condition, given as a discrete number and unit of measurement
☐	☒	A statement on whether measurements were taken from distinct samples or whether the same sample was measured repeatedly
☒	☐	The statistical test(s) used AND whether they are one- or two-sided<br>*Only common tests should be described solely by name; describe more complex techniques in the Methods section.*
☒	☐	A description of all covariates tested
☐	☒	A description of any assumptions or corrections, such as tests of normality and adjustment for multiple comparisons
☐	☒	A full description of the statistical parameters including central tendency (e.g. means) or other basic estimates (e.g. regression coefficient) AND variation (e.g. standard deviation) or associated estimates of uncertainty (e.g. confidence intervals)
☒	☐	For null hypothesis testing, the test statistic (e.g. *F*, *t*, *r*) with confidence intervals, effect sizes, degrees of freedom and *P* value noted<br>*Give P values as exact values whenever suitable.*
☒	☐	For Bayesian analysis, information on the choice of priors and Markov chain Monte Carlo settings
☒	☐	For hierarchical and complex designs, identification of the appropriate level for tests and full reporting of outcomes
☒	☐	Estimates of effect sizes (e.g. Cohen's *d*, Pearson's *r*), indicating how they were calculated

Our web collection on statistics for biologists contains articles on many of the points above.

Software and code

Policy information about availability of computer code

Data collection	Applanix/PosPAC MMS software version 8.8
Data analysis	RiProcess software (RIEGEL) version 1.9; Terra Scan (Terra Solid Ltd) version 019; LASTools, including PLAST tools, (RapidLasso GmbH) version 241210; Python version 3.12.4 batch-processing pipeline; Global Mapper Portgs 22/Global Mapper_Portgs_lic Version 22; QGIS version 3.40.2 Bratislava.

For manuscripts utilizing custom algorithms or software that are central to the research but not yet described in published literature, software must be made available to editors and reviewers. We strongly encourage code deposition in a community repository (e.g. GitHub). See the Nature Portfolio guidelines for submitting code & software for further information.

Data

Policy information about availability of data

All manuscripts must include a data availability statement. This statement should provide the following information, where applicable:

- Accession codes, unique identifiers, or web links for publicly available datasets
- A description of any restrictions on data availability
- For clinical datasets or third party data, please ensure that the statement adheres to our policy

All relevant primary data are provided in the article, Extended Data, and Supplementary Information. The complete dataset used to calibrate radiocarbon dates is available in Supplementary Tables 1a–b, and the full dataset of earthworks identified by LiDAR is listed with coordinates in Supplementary Table 2. Background data:

The river network shown in Figures 1 and 3a–b of the study area is derived from the MERIT Hydro dataset, as described in Yamazaki, D., Ikeshima, D., Sosa, J., Bates, P. D., Allen, G. H., & Pavelsky, T. M. (2019). MERIT Hydro: a high-resolution global hydrography map based on the latest topography dataset. Water Resources Research, 55, 5053–5073. https://doi.org/10.1029/2019WR024873. Extended Data Figure 8 was produced using OxCal 4.4.4, based on base map data provided under the Open Database License (https://www.openstreetmap.org/copyright). Finally, the Indigenous Territories mentioned in the artide and Extended Data Tables 1a-b and 2 are derived from the Divisão de Processamento de Imagens of the Instituto Nacional de Pesquisas Espaciais (INPE): https://terrabrasilis.dpi .inpe.br/geonetwork/srv/eng/catalog.search#/metadata/9f2b8d5a-0a65-4441-b217-8b632c82ebd5. The same data on officially recognised Indigenous territories are also available at https://forest-gis.com/gis-resources/areas-indigenas-na-amazonia-legal-shapefile/, while the background imagery in Extended Data Figures 1a–c and 5 is sourced from Google Earth.

Research involving human participants, their data, or biological material

Policy information about studies with human participants or human data. See also policy information about sex, gender (identity/presentation), and sexual orientation and race, ethnicity and racism.

Reporting on sex and gender	N/A
Reporting on race, ethnicity, or other socially relevant groupings	N/A
Population characteristics	N/A
Recruitment	N/A
Ethics oversight	N/A

Note that full information on the approval of the study protocol must also be provided in the manuscript.

Field-specific reporting

Please select the one below that is the best fit for your research. If you are not sure, read the appropriate sections before making your selection.

☐ Life sciences ☒ Behavioural & social sciences ☐ Ecological, evolutionary & environmental sciences

For a reference copy of the document with all sections, see nature.com/documents/nr-reporting-summary-flat.pdf

Behavioural & social sciences study design

All studies must disclose on these points even when the disclosure is negative.

Study description	Quantitative spatial and size analysis of earthworks detected using LiDAR and satellite imagery, with qualitative categorisation and corresponding quantitative population estimates.
Research sample	Laser mapping of ten ALS lines totalling 4497 km² in Acre and Amazonas states of Brazil. To the best of our knowledge, this was the largest archaeological dataset in Amazonia systematically surveyed using LiDAR techniques in 2024, making it adequate for regional-scale analysis. For comparative purposes, we also used our research group's satellite imagery database, published as supplementary material in Kalliola et al., Acta Amazonica 54, e54hu22351 (2024).
Sampling strategy	The sample size was determined by competitively selecting airlines approved by the Brazilian Ministry of Defense and maximizing the available funding grant. In order to predict the limits of the Aquiry civilization, two ALS lines was designed from the city of Lábrea to Carauari and from Carauari to Boca do Acre, totalling c. 800 km. Additionally, a flight line from Boca do Acre to Rio Branco, and from there to Manoel Urbano and further Lábrea, totalling c. 850 km, was added. Furthermore, to estimate the average density of the earthworks and total population both in relative high-density core and low-density periphery areas, six parallel ALS lines were planned between Rio Branco and Lábrea, totalling more than 2750 km. The survey lines covered an area approximately ten times greater than that of earlier systematic LiDAR surveys conducted by Prümers et al. (Nature 606,325-358, 2022) in the Llanos de Mojos (Bolivia) and Rostain et al.(Science 383, 183-189, 2024) in the Upano Valley (Ecuador). Furthermore, our LiDAR data revealed 396 previously unknown earthworks, compared to 24 new earthworks that were announced by Peripatao et al. in Science 382, 103-109 (2023). Our sample size was maximised, and we expect that sample is representative enough to make a new estimation of the amount of earthworks and the total population during its peak in the established area of the Aquiry civilization. Clear saturation of earthworks was not observed; however, the boundaries between tentatively high- and low-density earthwork zones could be delineated using LiDAR survey lines and satellite imagery.
Data collection	Data were primarily collected using a RIEGEL LMS-Q780 II sensor mounted on a Seneca aircraft. The aircraft flew at an altitude of approximately 1,125 m above ground level at a speed of 130 knots, with a laser pulse repetition frequency of 1,600 kHz and an average LiDAR point density of 31.2 points/m². There were no prior hypotheses regarding the targets to be identified, as the study area consisted largely of dense, unlogged rainforest.
Timing	All flights for LiDAR data collection took place between 6. June and 1. July, 2024

Data exclusions	26 earthworks observed in Indigenous territories are excluded from the detailed database.
Non-participation	N/A (Participants were not involved in the study)
Randomization	N/A (Experimental groups were not involved in the study)

Reporting for specific materials, systems and methods

We require information from authors about some types of materials, experimental systems and methods used in many studies. Here, indicate whether each material, system or method listed is relevant to your study. If you are not sure if a list item applies to your research, read the appropriate section before selecting a response.

Materials & experimental systems

n/a	Involved in the study
☒	☐ Antibodies
☒	☐ Eukaryotic cell lines
☐	☒ Palaeontology and archaeology
☒	☐ Animals and other organisms
☒	☐ Clinical data
☒	☐ Dual use research of concern
☒	☐ Plants

Methods

n/a	Involved in the study
☒	☐ ChIP-seq
☒	☐ Flow cytometry
☒	☐ MRI-based neuroimaging

Palaeontology and Archaeology

Specimen provenance	Note: The PDF does not allow "N/A" to be selected for the two previous sections. Therefore, only Paleontology and Archaeology are marked (X).<br><br>Some of the radiocarbon (C^{14}) dates are newly obtained samples from the state of Acre. Permission for C^{14} sampling was granted by the Instituto do Patrimonio Historico e Artistico Nacional, Brazil (IPHAN, licence 01423000336/2017-11), and the dated samples are listed in Supplementary Tables 1a–b.
Specimen deposition	N/A
Dating methods	All radiocarbon dates were obtained using accelerator mass spectrometry (AMS). Radiocarbon ages were calibrated at the 95.4% confidence level using OxCal v4.4 and a mixed SHCal20 and IntCal20 calibration curve. Bayesian modelling was applied using the KDE_Model function in OxCal v4.4.4.

☒ Tick this box to confirm that the raw and calibrated dates are available in the paper or in Supplementary Information.

Ethics oversight	No specific ethical approval is required to conduct ALS flights or to collect C14 samples in Brazil.

Note that full information on the approval of the study protocol must also be provided in the manuscript.

Plants

Seed stocks	N/A
Novel plant genotypes	N/A
Authentication	N/A
