# Supplementary material for: Over 20,000 precolonial earthworks in the Southwest Amazonia
Source: Nature. 2026 Jul 29;657(8131):420–5. doi: 10.1038/s41586-026-10835-7 (PMC13558069; doi:10.1038/s41586-026-10835-7)
Supplement: Supplementary file 2 [file 41586_2026_10835_MOESM3_ESM.pdf]

nature portfolio

Peer Review File

Over 20,000 pre-colonial earthworks in the Southwest Amazonia

Corresponding Author: Professor Martti Parssinen

Any redactions in this file are there to maintain patient confidentiality, the confidentiality of unpublished data, or to remove third-party material.

This file contains all reviewer reports in order by version, followed by all author rebuttals in order by version.

Version 0:

Reviewer comments:

Referee #1

(Remarks to the Author)
This manuscript reports extensive archaeological structures in areas of the Amazon that until around a decade ago were thought to have supported only little human habitation. The manuscript argues convincingly that estimates of population sizes in the Amazon should be increased even more. While mostly based on remote sensing, some ground-truthing is presented (yet with limited detail). The LiDAR data were obtained not just by drone but also by plane, thus covering large areas.

I was asked to focus my review on the radiocarbon dating. However, I will touch on some additional aspects where necessary.

Although the manuscript reports many new earthworks, most of the additional results have already been reported elsewhere. The radiocarbon dates have been calibrated using the most recent SHCal20 calibration curve, but this isn't a novel result as it had already been presented by Kalliola et al. 2024. Indeed, extended Data Table 3 (which is a Figure, not a Table) seems to be mostly or entirely copied from Kalliola et al. 2024. Were all 162 dates (of which 14 newly reported) calibrated again and are they all presented in the Figure? It would be much better if the OxCal model (both code and output) were shown rather than just black/blue summary bars.

Could stratigraphies / maps be presented for Eletronorte I and Piçarreira, including positions of C14 dates? What pretreatment was used for the charcoal and macrofossils of the newly reported radiocarbon dates?

Please use a consistent time-scale throughout - the Supplementary note mixes "calBC" and "calADs" with BP and "years ago".

The references to radiocarbon dating in the Supplementary note should point to 54-55, not 55-56.

I fear that the supposed cosmological/astronomical significance of the structures is a case of over-interpretation (lines 57, 95, 96, 324). Certainly no consistent orientation of the features can be seen. The shapes of some of the structures are certainly interesting, but any links with cosmology/astronomy are tenuous at best and are not backed up by the three supporting citations. Kalliola et al. 2024's comments are speculative: "The reasons for these design choices remain unclear. These deviations may stem from challenges in accurate measurement during implementation, but they could also represent unknown manifestations of the builders' cosmology", with no further argumentation or evidence presented for any cosmological purpose. Schaan et al. 2012 nor Saunaluoma 2018 mention anything regarding shared cosmology or astronomy, but rather interpret the open areas as likely used for social activities such as parties.

Could the different extrapolations of earthwork numbers as well as of population sizes be presented with uncertainty ranges? E.g., using A_{obs}=2380 km2, A_{tot}=98000 km2, and n=6280, and taking SD as $\sqrt{n*A_{tot}/A_{obs}}$, the SD would become 508.5.

The potential ecological implications, as mentioned in the Discussion, are interesting (though have been mentioned before in previous publications by the authors and others). Although impacts on climate are mentioned (line 22, 363), this needs more explanation. How would a human-managed Amazon forest affect local, regional and/or world climate?

Referee #2

(Remarks to the Author)
ACR comments on the manuscript by Parssinen et al.
Professor Parssinen, the lead researcher on this archaeological project in the southwest Amazon of Brazil, has led a significant amount of research on the prehistoric civilization that created the geometric mounds and ditched structures dubbed "Geoglyphs", in addition to extensive research on the archaeology and ethnohistory of the Andean foothills of Amazonia. For several decades, he and his colleagues have selectively excavated at numerous Geoglyph sites, dated many of the sites, collected archaeo- and ethnobotanical data from them, analyzed and compared their pottery styles, analyzed the shapes and possible symbolism of the mounds, and researched the available ground-based topographic and remote sensing data. The findings they've published are generally consistent with those of other researchers on the culture.

The early theoretical background cited for this research is the theory espoused by mid- to-late 20th century archaeologists at the Smithsonial that the interfluvial tropical forest of Amazonia was such a hostile and resource-poor habitat that could not have been densely or permanently occupied by prehistoric people. They concluded, therefore, that the numerous mounds already known there since the nineteenth century must have been purely ceremonial mounds without significant or permanent residential populations.
The archaeological takeaway of the Parssinen et al. manuscript is that a large section of the interfluves in Acre and Amazonas states in southwest Amazonia are demonstrated by a combination of their new Lidar surveys with earlier air photos, ground surveys, and excavations to have been unexpectedly densely settled for several hundred years between about the first and fourth centuries AD cal. The prehistoric culture turns out to have been a regional civilization with a settlement pattern of moderate-size, permanently occupied towns accompanied by monumental, symbolic earth mounds that were periodically visited for public ceremonials. The artificial earth mounds have been dubbed Geoglyphs for their varied geometric shapes, which most researchers think had astronomical orientations. The prehistoric culture associated with them achieved a population density much higher than that of the region today under modern intensive open-field agriculture and ranching, by developing a subsistence system that combined agroforestry and gardening, which maintained forest cover. Thus, a relatively high density of population was maintained even without the labor-intensive raised fields known mainly from floodplain areas. The occupation was a kind of so-called "low density urbanism" consisting of clusters of residences somewhat dispersed from each other, with cultivated forest and fields around them. The consensus holds that the Geoglyphs themselves were used only for periodic public visits for meetings and ceremonials, not for permanent, residential occupation. It also appears that this civilization managed to maintain mostly non-violent interactions within its region, perhaps by uniting under a common mythology, as expressed by the symbolism of in their styles of monuments and ceremonial pottery.

In this manuscript, the researchers convey details of their extensive new Lidar survey of 4,430 kms and its results. They compare the mounds identified by the new survey with

the distribution of mounds already known from their and others' earlier research, creating a summary map of all the mounds detected in the area, for a total of >20,000, where only 200 were originally detected. In a comparison of different mapping methods, they show that their use of Lidar increased the detection of earthworks even in deforested areas by at least a factor of five. They therefore multiply the numbers of mounds already detected in areas outside the current survey by that factor and extend that density to as-yet unmapped forested areas where mounds are already known to exist. They also add to the total the expected number of mounds of the low density mound areas they mapped, those areas where prior research showed lesser mound density. They then reduce the number of roughly contemporary mounds to 17,430, using the known radiocarbon dates, pottery styles, and forms of the earthworks, eliminating ones judged to belong to earlier or later periods.

The researchers make several different estimates of the size of possible populations that built the mounds. First, using the numbers of people estimated to have done the labor needed to build up the mounds - calculated earlier from the earth volumes/areas of the mounds, they propose a minimum total of 3,140,000 people for the area during the time of the civilization there. They then make a different calculation, for purposes of comparison. They make an estimate of the average population of a prehistoric settlement in the study area, using the average area of several later mound villages.
They then multiply this average village population by the number of their new mounds' total, to estimate the possible total number of people living in the study area at the time of the civilization. This calculation gives a population density of about 75 people per hectare, using a multiple of the average number of people in nuclear family houses, which, in Amazonia, is considered to be five. In yet another calculation for comparison, they reason that, if the area of an average mound was roughly equal to the area of its accompanying settlements, the associated population would have been about 215 people, giving a somewhat smaller total population of 2,250,000 million people when multiplied by the newly detected mound totals. They also calculate another possible population size by multiplying the average of 100 people per hectare of medieval Europe, for a higher total of 3,150,000 million. That total is closer to the initial total based on the labor needed to build the mounds. Regardless of the data on which these calculations were based, all the possible populations from them are much higher than previously supposed, basically because of the greatly increased numbers of mounds detected in their Lidar survey.

Comments on publishability and some suggestions:
The new mound maps by the researchers' Lidar surveys and the extrapolations based on them make possible an important new finding about prehistoric human settlement in Amazonia. Not only archaeologists but researchers and planners from many different disciplines will be interested in such findings. The finding that this interfluvial region was densely occupied in a dispersed settlement pattern both helps explain why the current forest over much of Amazonia shows distinctive features of human management, as Ter Steege and colleagues (2013) have earlier concluded.
Furthermore, the fact that humans managed to maintain forest cover by intensive

agroforestry and gardening during this dense occupation is an important result of obvious utility for informing future land-use to maintain of forest cover. Forest cover is essential in the rainfall cycle, as Salati and colleagues (1979) have shown earlier, and it prevents erosion of the valuable organic topsoil resulting from the decomposition of plant litter on the forest floor. To me, those three elements of the research makes the manuscript very publishable by Nature.
However, I had several suggestions to make the paper clearer. My suggestions can be read by both the editor and the researchers.
Areal definitions. The manuscript leaves the term "Aquiry" for the study area rather mysterious, just referring to two citatations, one of whose title indicates that it is in the Upper Purus River. In the interest of easing comprehension of the paper for readers without experience in the Brazilian Amazon, I think it would be good to define the term briefly when first mentioned in the text.
Furthermore, the paragraph on its geographical extension mentions variation mound distributions in areas with different rainfall patterns and amount of human-associated tree species in forests, but it lacks a final sentence indicating what is the significance of those variations for the research described in the manuscript? Only later on, in the next paragraph, do the writers say that their location of Lidar transects was designed to evaluate the extent of the Aquiry in particular. To me, paragraphs should end with a sentence that suggests what it has concluded and indicates how that leads to the next paragraph.

Population estimates based on area of later prehistoric villages: The reference to use of the areas of 20 mound villages published by Iriarte et al. 2020 does not make clear how the number of houses were based on the areas of the villages. The authors should make that more clear. Furthermore, the text wasn't clear on why the 100 per hectare population density of medieval Europe is relevant? It would be OK to cite that figure as a comparison but not to base the population per hectare in the Aquiry.
House size and family size. I also felt that the authors' decision to reconstruct a nuclear-family size for the ancient dwellings in the study area might not be realistic for Amazonia, where a majority of residences of living indigenous people are multifamily houses, ranging from 30 to 100 individuals, on average. In some cases missionaries have persuaded some communities to change to single-family houses, as with the Shipibo in the vicinity of Pucallpa in Peru, though the people still group the houses into multi-family compounds. Although not many social anthropologists give information about settlement size and resident population, I cite some of the literature I found in Roosevelt 1980: 196-221.

In the southern Amazon from the ethnohistorically-known contact period and until today, villages also had multifamily houses (Posey 1994: 271-286; Wust 1994:315-342). (These two reports show that the size of the southern Amazon villages in terms of number of multifamily houses shrank significantly as indigenous populations diminished

during the conquest, but in this area they continued to use villages with several multifamily houses.) In the Northwest Amazon, an area much poorer agriculturally than all these other areas including the Aquiry, discrete villages usually are made up of a single house, but it is still a large, multifamily house, nonetheless (Koch-Grunberg 1909- 1910). Similarly, in a single-maloca Wai Wai village the resident population was 26 (Yde 1965).
Having myself geophysically mapped and test-excavated two mound villages on Marajo and reviewed a large number of earth mounds there of a period AD 500-900, immediately after time of the Geoglyph civilization, I think the houses associated with the Geoglyph mounds would more likely have been multifamily than nuclear. We based our evaluation of the size of Marajoara houses on the number of large ceramic hearth
structures clustered in each house and the wide extent of the floors around them (Bevan and Roosevelt 2003; Roosevelt 1991: 333-342; Roosevelt 2007: 451-454). Much later and larger prehistoric sites like Santarem in the Lower Amazon mainstream do have small house mounds more suitable for nuclear families.

Perhaps calculating population using multifamily houses rather than nuclear-family ones would not make too much difference in the eventual regional population estimates, but, without any reason given in the article for choosing nuclear family houses as opposed malocas, why not just do the calculation on the basis of multifamily houses, as well?

Above, I've made references to my and others' published works to make my points about house size and population, but I am not suggesting that the authors need to add these particular references to their paper. But I do think that they need to at least insert a sentence explaining why they chose to estimate based on an assumption of nuclear family living, as opposed to the multi-family house so comon in Amazonia. And also I feel that they they need to cite some sources where the living population of such houses can be compared to the area of the settlement.

Bevan B. and A. C. Roosevelt. 2003. Geoarchaeological exploration of Guajara, A prehistoric earth mound in Brazil. Geoarchaeology 18(3): 287-331.
Koch-Grunberg, T. 1909-1910. Zwei Yahr unter den Indianern: Reisen in Nordwest- Brasilien 1903–1905. 2 vols. Ernst Wasmuth, Berlin.
Posey, D. A. 1994 Environmental and social implications of pre- and postcontact situations on Brazilian Indians: The Kayapo and a new Amazonian synthesis. In In Amazonian Indians from Prehistory to the Present: Anthropological

Perspectives, ed A.
C. Roosevelt. Tucson: U. Arizona Press. Pp. 271-286.
Roosevelt, A. C. 1980. Parmana: Prehistoric Maize and Manioc Subsistence along the Amazon and Orinoco. Studies in Archaeology. New York: Academic Press.

Roosevelt, A. C. 1991. Moundbuilders of the Amazon: Geophysical Archaeology on Marajo Island, Brazil. Studies in Archaeology. San Diego: Academic Press.

Salati, E., et al. 1979. Recycling of water in Amazonia: An isotopic study. Water Resources Research 15(5): 1250-1258.

Ter Steege, H. et al. 2013. Hyperdominance in Amazonian tree flora. Science 342, 1243092
Wust, I. 1994 The eastern Bororo from an archaeological perspective. In Amazonian Indians from Prehistory to the Present: Anthropological Perspectives, ed A. C. Roosevelt. Tucson: U. Arizona Press. Pp. 315-342.
Yde, J. 1965 Material Culture of the Wai Wai. Ethnografisk Roekke 10. Copenhagen: Nationalmuseets Skrifter.

Prehistoric land use. The authors cite Lombardo et al. 2025 in a sentence about different types of urbanism in Amazonia. However, that article is more about subsistence than urbanism and its take on subsistence is inconsistent with what the authors of the manuscript say about the subsistence system of the Geoglyph civilization. Lombardo et al. 2025 maintain that there was maize monoculture in the mound sites in the floodplains of the Bolivian Amazon, on the basis of finding more phytoliths and pollen from maize than from trees in the mounds there. But since grasses produce pollen and phytoliths to sediment by orders of magnitude more in quanitity than do most tropical trees, the evidence in that article is not conclusive at all for maize monoculture. In comparison, the archaeo- and ethnobotanical research Parssinnen et al. reference for the Aquiry clearly shows strong reliance on numerous arboreal food crops, like peach palm and Brazil nut, and other dicots, like manioc, not only on maize. Perhaps the authors could clarify what they mean by citing this publication.

Modern landuse
In mentioning how modern agriculture and ranching land use in the area nowadays supports many fewer people than the estimated prehistoric population in question, the manuscript could clarify in a footnote that most of the agribusiness is managed for export and not for support of local populations.

Suggestions for future research. It hampers definitive estimation of residential population size and density of settlement for this Amazonian culture that it's not yet possible to actually map prehistoric towns themselves, as opposed to the mounds, which were not occupied on a residential basis. Since this research team and many others have found that anthropic black soils are associated with the occupation by the mound civilization and that anthropic forest that grows on such soils often has
characteristic structure, composition, and texture, in the future the researchers might try to use multispectral remote sensing imaging to map that type of anthropic vegetation.
Doing that analysis, along with selective ground-truthing with test excavations to determine the culture of black soil areas with that vegetation pattern, would allow better assessmtne of the extent and number of living sites occupied at the time.

Typographic errors. In reference note 12, Ubelager should be Ubelaker. For the reference to Iriarte et al. 2020, the note number is 19, not 20 as given in the text. On p. 23 of the manuscript on line 3 of the Acknowledgements, the word "administered" would be a more common English usage than the word they use, which is "administrated".

Referee #3

(Remarks to the Author)
Pärssinen et al. present new data about the archaeological record in southwestern Amazonia, showing that the number of earthworks located in the Aquiry area is far larger than previously believed. They do this by a performing a lidar survey over a subset of the whole region, then extrapolating the findings from this subset to the entire region. They follow by quantifying the total amount of earthworks, calculating that between 2.25 and 3.15 million people lived in the Aquiry area in 200 AD. While the findings of numerous geoglyphs are notable, the authors overinterpret the data they have, and rely on a series of problematic and unqualified assumptions to arrive at their final population counts.
The new data coming from the lidar survey are undoubtably interesting and valuable to all archaeologists and (palaeo)ecologists working in Amazonia. However, this new dataset is only used to extrapolate the purported total number of earthworks, without providing any analysis regarding the type of earthwork. In other words, how many of them are mounds, enclosures, causeways or other types of features? How are they distributed over the landscape? What type of connections exists among different earthworks? How many cubic metres of earth were moved to build them? These are all questions that can and must be answered once they have the data to do so. They listed the new sites discovered in a table in supplementary information where there is a “Aquiry” column, indicating whether the earthwork meets the definition of Aquiry structure—but they don’t actually define what constitutes an “Aquiry” structure. In the same table, they have another column called “accuracy” with a binary code for “good” or “somewhat good”, without specifying what “somewhat good” means. This also begs the question of whether the authors have “bad” data, or simply don’t report it. They don’t enumerate how many sites are consistent with the “Aquiry” definition nor how many are “somewhat good”. Likewise, they don’t describe what are the implications of these categories; are the “non Aquiry” sites and/or the “somewhat good” sites excluded from the analysis? Basically, from a very interesting and novel dataset they only extract the number of sites to be used in the subsequent extrapolations, without saying if—and what type of—data filtering was applied.
The most important information that is lacking is the relative proportion of ceremonial versus habitational sites. These are fundamental data to later support population estimates. In fact, across the Americas, the earliest examples of monumental

earthwork constructions come from mobile peoples (e.g. Poverty Point; Ohio Woodland-period Hopewell) or populations at a very low population density (e.g. Olmec; Middle Usumacinta phenomenon; Norte Chico). At various points the authors speculate that their number of detected earthworks is an underestimate due to the possibility that the forest canopy was too dense and not enough ground points were obtained. This should not be speculation; places where this would be a problem can and must be identified from the raw lidar point cloud data.

By far, the most shocking number they present—which if true would warrant publication in Nature—is the population density they calculate for the area. Based on the numbers they put forward, the core Aquiry area would have had a population of 28 people/km2, which is almost 6 times the modern density population in the Acre and far higher than estimates for early Neolithic (0.6p/km2); Iron Age (1.2 – 2.3p/km2); or the Roman Period (14.4 p/km2) (Zimmermann et al. 2009, Human Biology). Given the implication of such a claim, it is extremely important that it is grounded on more-than-solid evidence. Unfortunately, the 2.25 and 3.15 million people claim is completely unsupported. The authors not only interpolate their mound density estimates to areas between their transects—which is appropriate—but they extrapolate these estimates to the entire region. This is grossly inappropriate because the boundaries of the region are only based on a few radiocarbon ages, and phenomena such as population densities are expected to be highly spatially autocorrelated. The authors blindly assume that forested areas of each block should have as many monumental structures as unforested areas; the reasoning for this is never made clear. At various points they argue that the present forest community composition is in large part predicated on the presence of ancient human habitation; thus, unless modern deforestation is truly random to this type of patterning, we cannot assume as such.

There are two major problems with their population density estimates. The first concerns how they arrive at this number. They make an extrapolation from another paper, also led by Pärssinen (reference 1). There, there is a 220 words paragraph, from which we copy here the most relevant portion:

“It is estimated that, in order to build one geoglyph of 200 m diameter, c . 8000 m3 of soil had to be removed. If one agrees that one man or woman could move 1 m3 per day, 8000 individuals per day or a workforce of 80 individuals for 100 days would be necessary to do the job. Considering that there is also a necessity for people to do domestic work and provide food and shelter for the workers, we could be talking about a population of at least 300. It is clear that more people could do the same job in less time, and that fewer people could do it in more time. Even though, considering a population of 300 people per geoglyph, it would be possible to envision a regional population of 60 000 people, which indicates a fairly dense population living in a terra firme area.”

Here, not a single reference is provided for such an estimate, but, more importantly, 60 000 is the number of people needed to build the 200 sites they identified in 100 days! But the geoglyphs were built during a period of hundreds of years. Therefore, the estimated population is orders of magnitude lower than what they claim to be. Even assuming that the speculation of 300 people per geoglyphs is correct, in order to estimate the population density, they need to estimate how many geoglyphs were built per year. The simplest way to do it is assuming constant population density and dividing the number of geoglyphs by the number of years during which the Aquiry culture existed. The plot with radiocarbon ages they present in ED Table 3—underlying the argument that the geoglyphs were constructed and/or in use from 100/150 to 350/400 250 AD—fails to include any confidence intervals. There is no explanation of how the uncertainty derived from the radiocarbon measurements and the calibration is taken into account. Based on partially-overlapping probability distributions for dates from fifteen sites, the authors claim 60% overlap in occupation. Overlap probabilities must be calculated with a Bayesian approach. Just because the probability distributions for dates from two sites overlap does not imply that the occupations were coeval, rather that there is the possibility that they are contemporaneous. Likewise, given that they argue that the scale of habitation is unprecedented, the number of dated sites is wholly inadequate to extrapolate at such a scale. For what we know, the geoglyphs could have been built, as they state in line 76, between 750/700 BC and AD 900/950—i.e during a period of 1600 years. If we take their number of 300 people needed to build a geoglyph in 100 days to be valid, then, in 1600 years, 513 people alone (not 3 million!!) are sufficient to build all the 20 000 geoglyphs they claim to have found!!!

The second major problem with this estimate of three million persons is that they don’t discuss at all the huge implications in terms of food procurement and environmental carrying capacity. In the discussion, the authors claim that identifying cultivated crops in the archaeological record sidesteps any carrying capacity estimates. It does not; it only shows what may have been consumed but not what the maximum cultivable quantities were. Even if we consider the lower end of their population density estimates, 12.5 people/km2 (or 0.125 people/ha), they would have had to cultivate the whole area—and in a pretty intensive way. Nowadays, an indigenous family needs at least 20 hectares for slush and burn agriculture plus a far larger area for hunting and fishing, wood harvesting, construction materials, medicinal plants and other quotidian necessities. If the entire landscape were under cultivation, we would expect to see evidence of agricultural features everywhere. Instead, not a single productive system has been identified by the lidar survey—or at least none that they report. Previous phytoliths analysis conducted in and around geoglyphs show that the immediate surroundings of the geoglyphs were forested and that people practiced agroforestry (Watling et al, 2017 PNSA), not intensive open field agriculture. Pärssinen et al. should explain how agroforestry could have supported 3 million people for 300 years in an interfluvial area. There is no way this could have happened.

Finally, with due respect to the authors, the manuscript would benefit from extensive editing by a native English speaker. The population estimates are fundamentally flawed. Even if corrected, the findings would still depend on questionable extrapolations rather than direct measurements. The lidar findings—while interesting—are not noteworthy enough for publication in Nature given the plethora of lidar studies published recently in this and comparable journals.

Referee #4

(Remarks to the Author)
I co-reviewed this manuscript with one of the reviewers who provided the listed reports.

Referee #5

(Remarks to the Author)
The key topic of this paper is that large populations were associated with the geoglyph sites in Acre and the surrounding states in Amazonia.The authors have engaged in a very substantial regional survey and have identified a phenomenon in the distribution of the geoglyphs which is significant because it does not actually fit within the conventional categories of urban and civilisation. This is an important phenomenon on a huge scale whose significance is submerged by old conventions which are assumed to be global when they are actually varied regionally differentiated labels with many differing meanings. That condition of plural meanings eg urban can be mobile which has been pointed out for at least five years. And large structure building by mobile communities from coastal Peru to Eastern Australia is well known as well as among the Inuit.

While the populations of the region were surely largely than has been assumed it is now not new to make such population claims - as the paper notes. What is novel is the actual spatial configurations and the area over which the consistent pattern extends.

The problematic data and methodology issue is that the estimates of population need far more attention.

There is a serious issue that purported large civilisations and big populations have been proposed before (in the 1930s and 40s) - such as the Azanian civilisation in East Africa - on the basis of massive amounts of earth-moving. In that case, several thousand square kilometres of mountain terracing on a spectacular scale was presumed to indicate large numbers of people and high levels of organisation. But the human capacity to move soil and rock is prodigious and the logistics of the human ability to do so is well-known eg see Erasmus for Mesoamerica and see Acabado on the Ifugao terraces in the Phillipines. Large populations and hierarchical states cannot be assumed.

The authors point out early on that the geoglyphs were used and made by mobile communities which were not resident in the enclosures.Then later talk of sedentary scattered communities but with no information about occupation within the enclosures or an empirical demonstration that the users were either mobile or sedentary. Assumptions about urbanism seem to have intruded. They also show in the figure of the group of geoglyphs some geoglyphs had been overlaid by others and that some geoglyphs are juxtaposed which could mean either that they are complementary or one supercedes the other. These two basic pieces of information mean that the number of geoglyphs being built at any one time and in use at any one time (two different circumstances) is not readily apparent. The brief remark that about 60% were in use at one time - over a few hundred years - does not resolve that issue. What is needed is a map of the distribution of the C14 dated sites. Are they clustered in one area or widely distributed? Do the dates come from one site size or are distributed across the size range. That requires the actual site size distributions by local area and in aggregate not just an average and a large value. This then needs to be integrated with the data on the number of superpositions and juxtapositions to provide an estimate of potential contemporaneity of use.

The density of occurrence of the geoglyphs also needs to be specified within the likely dating ranges. At a rough estimate about 70-80 geoglyphs might have been made per year at the peak time in the denser area - distributed across 85,000 sq km. They might have all been in one place but if they were not this is sparse activity. These issues need to be investigated as a basis for population estimates. Statistical analysis and specification of uncertainties is needed.

For information on "earthmoving" the authors should look to analyses in Mesoamerica and in North America (eg the Hopewell sites).

For the population estimates, extrapolating from residential settlements centuries later is problematic. They are residential which the geoglyphs have not been shown to be in this paper. And using those later estimates introduces a retrodictive fallacy.

In relation to densities the association to European urban densities is problematic because urban settlements globally operate across a large spectrum of densities. Conventional compact urbanism is not even a description of urbanism is the 20th and 21st centuries. And the Classic Maya and the Angkorian urban settlements operated at low-densities. Greater Angkor, the most extensive city of the agrarian world with 750,000 to 900,000 people had an overall density under 10 p/ha. There is no standard urban density.

In conclusion, the paper does not rigorously make the case for the claimed large populations. However, the authors possess a superb set of spatial data. The use of appropriate logistical analyses and rigorous spatial and temporal modelling is required to provide a probability assessment of the regional pattern which the authors are seeking to analyse.

Analytic procedures have been recommended above. The authors need to provide rigorous references for population estimates which will substantially include referencing key studies of logistics and labour populations.

The paper is clearly written but the presentation of the proposed population estimates is hard to follow because is spread over several parts of the paper and needs to be consolidated so that the components of the case can be readily accessible.

Roland Fletcher

Referee #6

(Remarks to the Author)
I co-reviewed this manuscript with one of the reviewers who provided the listed reports.

Version 1:

Reviewer comments:

Referee #1

(Remarks to the Author)
The Bayesian modelling presented in this revised manuscript is a useful approach to summarizing the radiocarbon-based estimate of the beginning and end of the Aquiry phase, as well as identifying periods of activity within the Aquiry Phase and for individual sites (KDE models; Extended Data Fig. 5). Several of the sites also contain evidence for previous and subsequent archaeological activity.

The usage of a mixed NH-SH calibration curve makes sense.

I'm not so convinced that the KDE activity timings could really be translated into space (Supplementary Fig. 3). Perhaps this Figure could be left out without detriment to the arguments made.

I agree that there is no need to provide much detail on the pretreatment of the charcoal samples, however please do cite a charcoal pretreatment paper (e.g., one of Bird et al. 2014, Gillespie 1997, Olsson & Kilicci 1964, or Wood 2015).

Otherwise I'm happy with the responses to my comments.

line 312, "1,31-3,14" should be "1.31-3.14".

References

Bird et al. 2014. The efficiency of charcoal decontamination for radiocarbon dating by three pre-treatments – ABOX, ABA and hypy. Quaternary Geochronology 22, 25-32. doi:10.1016/j.quageo.2014.02.003

Gillespie R. 1997. Burnt and Unburnt Carbon: Dating Charcoal and Burnt Bone from the Willandra Lakes, Australia. Radiocarbon 39, 239-250. doi:10.1017/S0033822200053236

Olsson IU, Kilicci S. Uppsala natural radiocarbon measurements IV. Radiocarbon 6, 291-307. doi:10.1017/S0033822200010754

Wood, R., 2015. From revolution to convention: the past, present and future of radiocarbon dating. Journal of Archaeological Science 56, 61-72. doi:10.1016/j.jas.2015.02.019

Referee #2

(Remarks to the Author)
REVIEWER #2's NOTES ON THE REVISED PARSSINEN My characterizations of the key results, significance, and originality of the manuscript are as I wrote them in my original review. Briefly, the results are highly significant and robust, as they are based on a large, new database as well as all relevant earlier results by the authors and by others.The methodologies used for gathering the mapping data and the analyses employed are valid and well described. I found the authors' characterization of the uncertainties excellent(and unusually honest). I base my evaluation of the research on my many decades of research in Amazonianarchaeology, ethohistory, and ethnology as well as in tropical ecology.

The authors' answers to the reviewers' questions about radiocarbon dates and the added supplementary information about them and other details seem satisfactory and,in fact, improve the manuscript. Minor errors, such as typographic errors, have been corrected. The authors' term, Aquiry, for the Geoglyph area as now defined, has been clarified, as a reviewer requested. A few sections about which reviewers had questioned the organization of the writing have now had to be removed to limit the length of the paper. Those are all satisfactory outcomes, for me.

The issue raised about "astronomical orientation"has been satisfied by editing the terms used in the article. I would like to mention, however, in support of the authors' interpretation of geometric images as elements of a shared cosmology, that such images used as symbols for astronomical bodies considered by current Indigenous Amazonians as important figures in myths of creation, are present in Amazonian rock art as early as c. 13,000 calendar years ago, in Monte Alegre, Para, Brazil.

The authors are correct to mention, in response to the question by reviewer #3 about the possible effect of a well-forested prehistoric Geoglyph area, that Salati and his colleagues and others have shown that at least 75% of the rain in Amazonian

forests derives from moisture stored and respired by the forest itself, not by moisture coming in from oceans, contrary to many researchers' expectations. If the Geoglyph makers followed the widely demonstrated current Indigenous Amazonian practice of maintaining heavy forest cover through use of agroforestry techniques, then rainfall would have been much more regular and consistent than it is in now deforested or "savanna" parts of Amazonia. Thus, the lesson of the Geophyphs, as presented by the authors, is one very relevant to the current and future of Amazonia as a habitat.

I agree with the authors that reviewer #3's questions are in some cases based on an inaccurate grasp of the original manuscript and also on speculations that, although interesting, seem beyond the subject of the manuscript and in any case that would not enhance its relevance for Nature. In any case,the authors make several useful clarifications to address most of reviewer #3's more relevant comments and questions. I feel the need to mention that reviewer #3 seems to be influenced by some early assumptions about Amazonia necessarily having a limited carrying capacity because it is a tropical forest. Many of we archaeologists who work in Amazonia have done conclusive empirical research of various kinds that shows that the environmental limitation theory of Amazon prehistory has not been upheld. Furthermore, that reviewer seems acquainted only with Indigenous slash and burn cultivation, whereas numerous Indigenous peoples as well as acculturated Amazonian people (the majority of whose DNA is Indigenous, showing that despite no longer speaking Indigenous languages, they are long-term inhabitants of Amazonia) get most of their calories from tree crops, whether domesticated, cultivated, or wild. As recent researchers, whom the authors refer to in their response, show in various ways, permanent cultivation and harvesting of trees and shade-tolerant annuals and perennials, whether in terra firma or floodplains, was the main subsistence activity, supplemented by fishing and hunting. This type of landuse has definitely been shown for sitesin the Geoglyph region. Furthermore, reliance on forest utilization for a major part of subsistence would not leave any particular type of landform such as what the reviewer seems to expect. As an archaeologist an environmentalanthropologist, I do not find the population estimates of the authors "shocking" but, as I mention in my review, they seem consistent with those I made based on the sites I have surveyed and excavated in other parts of Greater Amazonia, some of which I mention in my review. I should also mention that my review of studies of pollen results carried out for both prehistoric and modern periods show no indication of wide-areal deforestation in prehistory. The percentages of grass that modern deforested or "savanna" areas have do not occur in prehistory in the places that have been studied (E.g., Roosevelt 2021 and Absy 1979).

As for reviewer #5, who questions how population estimates can be made when the sites mapped are primarily ceremonial, one can point to the pattern of ceremonial meetings among living Amazonian societies, such as the female initiation rites among the Shipibo of the upper Amazon in Peru orthe funerals of the Kayapo and neighboring groups in the middle Xingu. Both groups were very populous during the prehistoric period, as Myers shows for the Shipibo and their neighbors and as Posey has shown for the Kayapo. For these events, as I know from attending some of these ceremonials, people are invited to come from many residential communities in the surrounding area to central places, where they spend several weeks dancing, feasting, wrestling, trading, etc. Thus ceremonial constructions like the Geoglyphs would represent meeting places for a number of residential villages. Thus, making population estimates is a valid exercise, and the authors have made different estimates based on different possible community and family house sizes. The reviewer praises the results of the authors' Lidar mapping program and the statistical analysis,and the authors have satisfactorily addressed his/her suggestions, I think.

Signed

A. C. Roosevelt

Referee #3

(Remarks to the Author)
This manuscript presents two main results: (1) the quantification of the number of geoglyphs, and (2) the implications of this quantification in terms of estimated population density. While the first part represents “hard data,” and our previous comments have been partially addressed through clarifications and improved analyses, the second part (see the paragraph entitled Estimation of the population in the revised manuscript) remains purely speculative and unsupported.
Their population estimation relies entirely on their own publication (Pärssinen et al., 2009), where they assert—without any supporting evidence—that building a geoglyph with a diameter of 200 m would require 80 individuals working for 100 days. This provides a metric linking the number of earthworks constructed to the amount of labor over time. However, in response to our main comment, the authors now claim that “it is not important how many geoglyphs were built per year.” Moreover, in the manuscript they state: “To calculate the population of the Aquiry, the first obvious question is: how many earthworks were in use at the same time?” (line 252, revised manuscript) and “if an average earthwork required 300 people to build and maintain it, we arrive at a total population of about 1.31–3.14 million” (line 276, revised manuscript).
I strongly disagree with conflating the number of people allegedly required to build the earthworks with those needed to maintain them or with those who might have used them simultaneously. These are three fundamentally different types of activities. This confusion between metrics is repeated both in their responses to us and to Referee 5 (who raised the same concern), where they write that “the focus of this article is not on the number of earthworks constructed per year, but rather on the peak period during which the largest number of earthworks were used simultaneously.” Here again, they shift from discussing the number of people needed for construction (the figure provided in their own paper) to discussing “simultaneous use” and “maintenance.” Estimates of the number of people associated with each of these activities should be based on distinct and independent lines of evidence. Yet there is absolutely no supporting evidence for how they estimate the number of people who would have simultaneously used or maintained these geoglyphs.
In our opinion, Pärssinen et al. (2009) cannot be considered a valid reference for estimating past population densities, as it lacks any robust supporting argument; and even if the estimation in Pärssinen et al. (2009) were reliable, the metric used there concerns construction time per unit of labour. Therefore, it is essential that the authors consider, based on radiocarbon

ages and their chronological model, how many geoglyphs were constructed per year.
When asked to “provide rigorous references for population estimates which will substantially include referencing key studies of logistics and labour populations,” the authors reply that this will be “a focus of a future study.”
To conclude, I believe that the claim of extremely high population density made in this manuscript is unsupported. Without such evidence, the mere quantification of the number of earthworks is not sufficient to warrant publication in Nature.

Referee #4

(Remarks to the Author)
I co-reviewed thisB→<↑sub> manuscript with one of the reviewers who provided the listed reports.

Referee #5

(Remarks to the Author)
As this is a review of a revised manuscript we will proceed directly to overall and then specific comments. A core issue in the manuscript is the play on terms such as civilisation, urban and dense.

The issue with civilisation is that it is being used in two different concurrent ways because it is an inherently vague, poorly defined term. If it were replaced by "cultural tradition" throughout the early part of the paper the implicit "quality" and "magnitude" inherent to the term "civilisation" would be absent. And the jump at the end of the paper to civilisation referring to massive, wealth state organised societies with large settlements (eg Greater Angkor) several orders of magnitude larger than the earthen enclosures would then be glaringly apparent. The authors would need to explain why comparability is claimed across several orders of magnitude to societies which are literate, had major state administrations and built very substantial structures.

Urban is used because it is term which possesses cachet and has rapidly gained a vague and plastic identity as an academic category. The authors have many companions worldwide in this practice of overextending the term urban.

And dense is readily used by the authors when it is not an appropriate descriptor. A region of 183,000 sq km with an average density between 0.07 persons per ha and 0.2 persons per ha, at maximum, is not densely occupied. Extensive dense rural provinces would have overall densities in the 2-5 p/ha range.

Comparison to other societies produces further issues - the Neolithic enclosures and megalithic monuments of Europe are considered to have had public, ceremonial and political functions in relation to visits by mobile regional populations. The monuments display mathematical and geometric competence and involved cosmological understandings. They required organised labour. Are they, on the analogy proposed by the authors therefore urban? -a term which specialists who work on Stonehenge reject. And has repeatedly been pointed out large structures do not require large amounts of labour. The population estimates made on the basis of maintenance costs are problematic because the evidence is not provided that earthen embankments stabilised by vegetation need substantial maintenace.

A specific set of data which is required in this paper is the size distribution of the earthen enclosures. Does it have a characteristic form of many small features, some medium sized features circa 10-14 ha perhaps, a a few rare larger ones at circa 50 ha? Readers need to know, because reference to average sizes is problematic when a massive skew to a few large cases is occurring.

The occurrence of the earthen enclosures over time also needs to be reconsidered. Even with a claimed peak period of only 200 years (note - in a 1500 year tradition) in the 85,000 sq km denser area 22,400 enclosures generates 4 per sq km ie 1 on average in a 25 ha area ie "half" an enclosure in a century at peak activity. This surely is not substantial or rapid construction. Is the total population actually an aggregate of all the people who have lived in a region over a span of time rather than a maximum population present at any one time.

Note that a Britannica reference - 39 - is not sufficient nor is the use of Curet 1998 as a source for population estimates that is ove 25 years old.

Kirrily White has also added some additional specific comments

Rebuttal Document

p. 25 – are they suggesting that state hierarchy was required to construct the ceremonial etc. earthworks in Acre?

p. 26 – addressing of mobility seems adequate, there is insufficient information available to date appearance of domestic flora in the area, or to understand the dating and structure of mounds which have not been previously identified as later mound villages.

p. 27 – they have provided more information on the radiocarbon dates, locations, and areas – see file 535106_0_supp_4929181_t4ll10, (supplementary data) lines 45-174. Dated sites are limited but are distributed over a largish area of the Acre earthworks and a few sites in Bolivia and Amazonas. There is tabulated information in the supplementary data.

p. 28 – they have addressed the density of occurrence of geoglyphs in the dating ranges. Most of the dates are for Acre. There is more information about this in the supplementary material. There is a series of maps in the Supplementary material on p. 4 which shows distribution of dated sites by blocks of time. There are also some sites with multiple dates showing phases of use. See also lines 117-174 on pp. 6-7 in the supplementary material. I think that without identification of occupation areas, they have to go on the size of the earthworks or maybe conjoined earthworks, so that the sizes are all small.

p. 29 – see note below for supplementary data on population size, they don't have much to go on, and some discussion of population seems to be needed.

Supplementary Data

pp. 2-3, lines 47-74. I am concerned that they are not interpreting the KDE data appropriately by using percentages, would like to discuss it with you, but might need to consult a mathematician.

pp. 7-8, lines 175-206 discussion of population size. Importantly, they are talking about very small villages and I am aware from Roosevelt (1991. Moundbuilders of the Amazon: Geophysical Archaeology on Marajo Island, Brazil. Academic Press, San Diego, p.342.) that multifamily longhouses could have 35-60 occupants. In her thesis (pp.171-72), Denise Schaan uses this estimate to propose 12-60 people per building for Camutins mound complex on Marajo Island – the range accounts for the different sizes of houses and mounds.

The way the population estimates are presented in this paper for the geometric earthworks does not seem to be outside the norm for literature in the Amazon regions

even though there is not yet occupation evidence, besides perhaps size of earthworks, to base them on and all they can do is use what researchers have said about other regions.

pp. 8-14 is discussion of lidar methodology. I can't really assess this, but explanations and data appear to be thorough.

pp. 20-28 is a list of site sizes measured from the satellite and lidar imagery.

Revised Paper

p. 2, lines 40-46 – dense populations? Again it's problematic – but it is frequently used in the literature without clarification of what dense means and the discussion in Amazon is on types of urbanism, so it isn't an outlier in terms of literature from other regions.

Lines 51-52 – again the issue of justification of population estimates. Does the provision of the areas of earthworks help? They have no occupation data and are using comparison with other regions, the size of mounds used at a later time period and ethnographic analogy – is it sufficient?

p.4, line 97 – Comparison with Inka, it is comparing apples and oranges.

p.5 lines 110-11 – it's not wrong to say that researchers do use these criteria, just wrong that they do use it – all kinds of hunter gatherers built earthworks and practiced arts etc…

Most of the paper is description of the data and discussion of different scenarios of interpreting population sizes. It might be helped by a discussion of population mobility, but they are not claiming large cities, just piling on the discussion of urbanism elsewhere in the Amazon literature. I am confused about whether or not they are trying to claim the Aquiry are a state as well as a civilization…

Referee #6

(Remarks to the Author)
I co-reviewed this manuscript with one of the reviewers who provided the listed reports.

Version 3:

Reviewer comments:

Referee #2

(Remarks to the Author)
My comment notes on Parssinen et al. re-resubmission February 2026

Overall, the research presented, illustrated, and interpreted in the current manuscript is important new data on the character, location, and chronology of the ancient culture of the Geoglyph defined by its ceremonial earth constructions. The data is, as

the authors state, an important contribution to understanding how Amazonians managed to occupy terra firma terrain of the basin while also maintaining the forest cover for over 12,000 years. Their long-term research on the Aquiry culture has also been important in showing another example of the diversity of regional cultures that Amazonians developed over the milennia and the long-term continuity of human occupation in the area.

The research has potential applications nowadays, as the authors suggest. Right now, Amazonia is in a perilous situation. Deforestation has sped up until recently, and even now, agribusiness uses that require deforestation - intensive cattle ranching for meat and clear cultivation of soybeans - have become important in the export economies of Brazil and its neighbors. Despite the current economic importance of beef and soy for export, production of tree fruits for export would be much more sustainable and lucrative economically, but would require a shift in agronomic approach and a willingness to produce fruit of various species, like Moriche/Buriti, peach palm, and Caryocar, species with nutritious, sweet or savory fruits as yet undeveloped for the export trade. (Acai took off like wildfire, but other fruits have even more potential, and there are legions of candidates.) By the building of prosperous local businesses consistent with forest preservation and regeneration, rural poverty, soil erosion, and drought could be alleviated, while also increasing the GDP. In addition, engaging both indigenous people and their rural neighbors in developing entrepreneurship and taking part in conservation planning for managing forests and developing aboriculture. Thus, Amazonian countries can take advantage of badly needed cultural expertise that for the most part has not been utilized, despite the strong causal association of still-existing tropical forests with the presence of indigenous/rural populations.

The authors have been responsive to most of the reviewers' criticisms, and I don't see any compelling additional criticisms, in the current reviews, relevant to their imaging and measurement methods and consequent conclusions about the numbers of prehistoric earthworks and the implications. So, their findings from that research don't seem controversial in essence, and they are novel, showing that many more mounds were built and more densely placed during the particular prehistoric period than was expected. That even the terra firma of Amazonia incurred dense human occupations during the last few thousand years of prehistory does fit with much of the recent archaeological data on earthworks and habitation mounds elsewhere in Amazonia and it also fits with the ethnohistoric evidence for existence of larger population sizes in contact-period settlements and ceremonial centers than there are in current indigenous settlements.

Reviewer 1 seems to be mollified on the whole by the responses from the authors and their revisions to the manuscript. But, contrary to this revewier, I do think the chronological illustration should stay in, because it does show that constructions that were mapped were made in a well-defined cultural period. Thus Parssinen et al.'s mound count totals clearly relate to the extent of that particular culture at that time period, and are not a hodge-podge assemblage of earth constructions from a wide range of occupations. In fact, the authors are careful to not exaggerate the number of Geoglyph mounds or areas that have them (lines 114-134).

Revewer 3, though has now doubled down on their criticisms, mainly on the issue of population estimates. His/her two main contentions seem rather unfair. The claim that the article authors rely on only one source for their estimates isn't really accurate, as the authors contest, considering their many publications dealing with different types of evidence for reconstructing population and their discussion of various alternative methods to do so, in the manuscript to Nature.

As for reviewer 3's criticism that the authors use too many different ways to assess what the earthworks might represent in settlement population sizes and densities, that criticism seems to conflict with the first criticism. In any case, to me, it's useful to compare different ways of estimating population for a prehistoric culture. Amazonian cultures, the shape and size of their settlements, and their propensity or not of building earthworks make them extraordinarily varied. Thus, no one choice of ancient, historic, or modern culture could necessarily be the perfect model for any ancient one. But Iriarte's maps and estimates of later mounds and settlements in the area, combined with the ethnohistoric and ethnographic testimonies he references, do, as the authors point out, make clear that by then houses were malocas, which are extended family houses, not nuclear ones. Malocas, as I mention in my reviewer comments on earlier versions of the manuscript, are the most common type of family residence documented in Amazonia before intensive missionization. (MIssionaries did not like extended family living, especially not when the family was matrilineal.) And, as the authors show, the different ways of reconstructing population for the Aquiry tend to produce comparable results. In any case, the authors give a wide range of population in their estimates, not a single estimate. (By the way, no nuclear family house I've observed in Amazonia was as long as 25 meters; that size would be an extended family house, a maloca.)

Reviewer 3's questions about the chronology of the building and use of the Geoplyph earthworks seem unrealistically persnickety, given the authors' very detailed discussion, analysis, and illustration of the radiocarbon dating and the mapping project. Furthermore, I found the current manuscript's descriptions of methodology in the supplements very clear and detailed.

Reviewer 5. This reviewer's comments/questions about political organization are interesting in that the issue of the range of types of societal organization in tropical rainforest areas has been debated over the years. At first, anthropologists assume that the "hostile" tropical forest prohibited the development of any complex societies there. For that reason, Meggers et al. assumed that any complex societies found there, like Marajoara, had to be brief, unsuccessful intrusions. (There is no doubt about the Amazonian origin of that culture and its long life-span, contrary to Meggers. The initial dates for Marajoara in eastern Amazonia are about 1000 years older than those of its descendants in the Andean foothills of Amazonia.)

Anthropologsts had argued that societies with "high" art and monumental architecture must have been centralized and stratified, á la Julian Steward. Different researchers have referred to such complex societies as either chiefdoms or states. But some Amazonian large-scale moundbuilders with highly complex cultures do not show clear evidence of stratification or

political centralization. For example, the Marajoara culture does not present evidence of social stratification or centralization, although Meggers, who wrongly thought it was an Andean intrusion, sometimes used the term state for the culture. But all the sites of the culture studied so far have two main kinds of material culture: highly decorated, sometimes very large vessels in the context of burials and special food service with special foods and drinks in feasting areas, and much less-decorated pottery usually in smaller sizes, in the context of ordinary food and drink consumption at household stoves and in black soil garbage. (I present information about these distributions in Roosevelt 1991, and the whole issue of the implications of monumentality in South America is discussed in Early New World Monumentality, edited by R. L. Burger and R. M. Rosenswig. Gainesville: University of Florida Press. 2012.)

Denise Schaan in her dissertation (2004) hypothesized that the Marajoara society was a chiefdom. But since chiefdoms are considered to have two levels of strata: elites and commoners, and presumed centralized control, the differential material culture patterns within the sites seems, instead, to represent houshold daily life on the one hand and special ceremonial events on the other. And almost all Marajoara sites reported so far are mounds topped with multiple malocas. And, although there are large clusters of as many as 20 to 40 mounds as well as trios or singles, the character of their material culture and its distribution are not different in ways that would suggest that the large mound clusters were in control of the smaller mound sites or had special material culture that singles or small mound clusters did not have.

This reviewer 5 also takes issue with the authors' use of the term "civilization" for the prehistoric Anquiry culture, and suggests that they should use the term cultural tradition instead. But nowadays in Amazonian studies most researchers use the term civilization for cultures that have significant cultural complexity in some form, whether monumental constructions, whether they are ceremonial, residential, or agricultural, archaeoastronomical and/or monumental rock art, monumental sculptures and paintings, elaborate crafts and fine art, large, organized settlements with specialized centers, an extensive geographic sphere, or some combination of these characteristics. Thus, the elaborate, monumental, carefully planned, standardized, and geometrically precise earthwork constructions of the widespread Aquiry Geoglyph culture would qualify it to be called a civilization.

This reviewer also objected to the authors' listing the literate Khmer civilization as one of the world's great civilizations in the same breath as the Aquiry culture. But none of the ancient complex cultures of South America considered civilizations were literate, and in general anthropologists don't separate the early literate civilizations from the non-literate ones as different organizational classes of society. Thus, the Huari and Inca empires are considered civilizations, as are the much more modest Chavin, Moche, and Chimu cultures. Societies considered civilizations by anthropologists vary greatly in their characteristics. Many were highly urbanized, but some important imperial ones, like the Inca, were not, and settlement patterns changed through time. As John Rowe pointed out early in the history of anthropological research in South America, ceremonial centers in the Andes can exist separately from residential settlements, and ancient complex cultures there vary in whether people live in such centers or if the centers are empty when not used for ceremonies, with the resident populations are dispersed around them. And, as the authors say in their rebuttal, they mention the Khmer society only to emphasize its contrast with the Aquiry in regard to "hydraulic civilization".

In any case, in Greater Amazonia several expansive mound-building cultures developed that are uncontroversially classified by anthropologists as highly complex societies and often referred to as civilizations: the Marajoara culture, the Santarem culture, the Upano society, the Llanos de Mojos mound culture, and the Geoglyph culture. All but Santarem were long-lasting regional cultures, and the Marajoara and Santarem cultures were also "Horizon Styles", called thus because their art styles and settlement patterns rapidly spread through much of Amazonia from their regions of origin. The settlement patterns and earthwork constructions, food choices, agricultural methods, and hunting and fishing methods vary a lot from culture to culture, but all had earthworks, diverse, highly decorated, elaborate, and abundant art and craft production. And all are considered "urban" in settlement pattern either because they had a large nucleated ceremonial-residential center with dispersed towns and villages, like Santarem (at least 4 square km in area, or because they had non-residential ceremonial centers with dispersed residential settlements like the Geoglyph cultures. Or in the case of Marajoara, because they had large and sometimes highly clustered multi-maloca mound residential-ceremonial settlements as well as numerous multi-maloca residential-ceremonia settlements on small mounds.

Reviewer 5's comments about the Stonehenge monument are interesting and a bit clueless. The reviewer says that researchers on Stonehenge would not call it an urban society, and that's no surprise. As the authors point out, this monument is teeny-tiny compared to most Geoglyphs, and the number of known ceremonial sites like it in an area comparable to the Aquiry are very few. Furthermore, the culture of the people who built different stages of the monument is not well defined. No regional prehistoric culture has been defined for the monument in the ways that the cultures of the Amazonian complex cultures mentioned above have been defined: residential forms and their distribution, monument forms and distribution, the particular material culture, geographic extent of the material culture, patterning of material within residential settlements and ceremonial places, burtial customs, etc. It would indeed be premature to decide the issue of the settlement pattern and sociopolitical organization of that poorly-defined culture. Therefore, I don't see its relevance to the article under review.

Reviewer 5 also takes issue with the authors' use of Curet's estimates of the populations of Antillean archaeological sites as out of date. But the authors refer to numerous other archaeologists' estimates over the years for possible populations of tropical lowland archaeological sites in comparison with relevant ethnographic and gephysical evidence, including mine from the 1980s, 1990s, and 2000s, and especially Iriarte's recent results from the Geoglyph region at a later time than the Aquiry culture. I've always liked Curet's analyses because he used geophysical survey to augment surface findings and excavation data at sites, as we have in the Santarem area and on Marajo (E.G., Roosevelt 2007). The reviewer's comment therefore seems captious to me.

K. White's review. In regard to the idea of the Inca being apples to Amazonia's oranges, and therefore not an appropriate culture to compare with them, she may want to think back to John Rowe's long-ago foundational articles on Inca settlement patterns and how they, despite indeed being an empire, did not have the large dense urban settlements expected of an "urban civilization" (Rowe 1963: 18-19): "The Incas were not city builders...Large cities are not as generally characteristic of Peruvian settlement as small urban settlements." He thought that in many Andean periods, ceremonial centers without permanent population offered the needed services. And Richie Burger would readily admit that the Andean Chavin culture had many fewer occupation sites than the Marajoara, Santarem, or Upano cultures. Furthermore, though in the article Marrti Parssinen was modest about his research on the Inca, merely listing one of his works in the references, he is an established, well-published specialist on Inca ethnohistory and on the historical and archaeological record of the Inca expansion in Amazonia.

(To defend K. White's comment about the existence of complex cultures and monumental buildings among prehistoric hunter-gatherers, I would point to the populous Japanese Jomon culture as a prime example of one. But I don't think that the authors need to digress in that direction.)

By the way, in her review, K. White mentions Schaan's estimates of numbers of mound builders for a mound at Camutins, which numbers she notes were based on estimates I made based on my geophysical surveys and excavations at Teso dos Bichos (Roosevelt 1991). But it's important to note that the Os Camutins has c. 40 residential mounds total overall, and the main Camutins mound itself is almost kilometer long before it was looted. So, that mound would have had not just 12-60 people but hundreds, and the multimound site it is part of would have had thousands in the 10 square kilometers in which the mounds are distributed. So, a really dense occupation.

In any case, the authors do not claim, nor has anyone that I know about seriously made the argument, that the Geoglyph cultures constituted a state, though some researchers may consider it a chiefdom. However, without being able to map settlements, but only the ceremonial constructions, all population estimates have to be indirect, and without systematic research within residential sites, their organization can't realy be evaluated. When settlements of the culture can eventually be found, mapped, and excavated, the issue can be looked at more directly. (Some of our very preliminary analysis of GPS ground-truthed Landsat thematic images from the Santarem area suggests that black soil garbage habitation deposits have enough effect on forest vegetation on them that they may be perceptible remotely.) Furthermore, some Ge-speaking round-village indigenous societies relatively near the Geoglyph region do have terms like "noble" and "commoner", according to the ethnographic studies of Nimuendaju and several later researchers who did their dissertations under David Mayberry-Lewis at Harvard. And Heckenberger and Peterson's mapping of roads in the Upper Xingu in late prehistory implied some degree of centralization in ceremonial activity. But in all those studies, it does not seem that the societies had the types of stratified and centralized sociopoliitical organization that most anthropologists would call states.

Referee #3

(Remarks to the Author)
Pärssinen et al. have provided a lengthy rebuttal to my comment from the previous round of revisions. While it addresses many secondary aspects, it does not engage with the central issue. I will therefore try to be clearer here.

They state that 300 people can build a geoglyph of 8,000 cubic meters in 100 days. They also write: “We clearly state that this 'independent' chronological activity model yields a figure of 4,360 earthworks per 200 years, equating to an average of 22 geoglyphs constructed per year between 100 and 300 AD. According to our various estimates, this would require an average community of 300 people, multiplied by 4,360, equating to 1.3 million people.”

This final sentence is where the main disagreement arises. If 300 people build one geoglyph in 100 days, they can build 3.65 geoglyphs per year, or 730 geoglyphs in 200 years. If 4,360 geoglyphs were built in that period, the implied population would be 300 × (4,360 / 730) = 1,792 people, not 1.3 million.

This number could be increased based on other lines of evidence, such as habitation sites, labor required for maintenance, demographic modeling, and estimates of carrying capacity. However, even accounting for all of these factors, it is not possible to reach a population estimate of 1.3 million.

They cite Hirst et al. (2025). In that paper, Hirst et al. estimate the average population of the Casarabe Culture, based on the energetics of constructing all known earthworks, to be between 9,000 and 60,000 inhabitants, accounting for the movement of 50 million cubic meters of earth over 1,000 years. In contrast, the Aquiry earthworks involved the movement of 8,000 × 4,360 = 35 million cubic meters in 200 years. Taking the upper limit of the Hirst et al. estimate, the maximum population of the Aquiry would be 60,000 × 5 × (35 / 50) = 210,000, which is still far below 1.3 million.

If we instead apply Pärssinen et al.’s calculation to the Casarabe culture, the resulting population would be 50,000,000 /

8,000 × 300 = 1,875,000 people within an area of 5,000 km², corresponding to a population density of 375 people per km². This is between 20 and 200 times higher than the estimates reported by Hirst et al.

Given the above, I reiterate the concerns already expressed in my previous revisions.

Referee #5

(Remarks to the Author)

Logical inconsistency in the use of terms

The current logical issue in archaeology is that there is no agreed or correct definition of either civilisation or urbanism. As is emphasised in Fletcher (RJF) 2020 in the Journal of Urban Archaeology there are numerous regional definitions of urbanism and no group of regional specialists will concede to any other NOR is there an agreed global definition. Nor does RJF argue for one. RJF argues for the designation of a universal “frame of reference” which would be quantifiable to allow global cross-comparison and has proposed such a frame of reference on which White (KW) has been working.

Our specific emphasis is that currently there is a serious logical problem created by regional specialists claiming their own definitions AND concurrently wishing to refer to usage elsewhere to claim a global legitimacy derived from the cachet associated with the terms “urban”. The same applies to “civilisation” – there is no agreed definition or usage of the term. The French refer to Palaeolithic cultures as “civilisation” but that is not the conventional English usage. The term civilisation has also rather gone out of fashion in scholarly commentary and is primarily used only as a standard label eg Roman of Chinese civilisation. We do not argue that there is a proper definition of civilisation because there isn’t one.

The usage in the paper of a regionally specific term and then claiming association to world-wide examples which purports reference to a global term that actually has no agreed definition is logically inconsistent.

General definition claim

Sentences 110-111 are problematic and illustrate the issue. Among the list of words only “administration” is a term among Western criteria used to distinguish towns from villages. Universality has been invoked but all the other labels “sociocultural and cosmological motivations. Including diverse forms of art, dance, music and sport” are characteristics of immensely varied societies including the Australian Aborigines (The First Nations of Australia), the North American Plains Indians and numerous other societies. If all such societies are to be labelled as possessing towns then the significance of the category disappears. We are not advocating for a specific meaning of the label town, only emphasising that the effort to use a generality ascribed to another regional case from elsewhere to validate a particular regional designation - which the authors advocate - while concurrently claiming the uniqueness of their regional designation is not logical. To illustrate further – do the bora rings or the huge, hectare-sized eel traps build by First Nations Australians, or the inukshuk stone trap-lines several kilometres long constructed by the Inuit, or the construction of the massive earthen cursus structures near Stonehenge display the presence of “administration”? Or has “administration” been stretched to mean human collective action, without any trace of the management accoutrements familiarly associated with administration? The paragraph is problematic.

Population estimate issues

In reference to the population estimates the procedures remain problematic. First, the reference to the later mound sites is irrelevant to the calculation derived from the reasonable logistical estimate of the number of people who would be needed to construct a geoglyph. Secondly, the issue remains that no actual demonstration has been provided of the contiguity of the use of the sites except over many decades and that removes the one-to-one singular connection between a builder population and one site. As the authors have shown elsewhere in their work the charcoal in the sites relates to episodic burning of the local vegetation. The sites are traps for that material. The Bayesian analyses do indeed show the increasing and then the decreasing amount of that burning over time. The ceramics in the sites do indeed show that people are periodically passing through the sites. But neither shows that the population at any one time equalled the total population required to provide different labour populations to build each site that occur within a C14 error range of about 30 years. Because, if one group of people is constructing a new site every few years - say every 6 years - then the population required to construct a set of sites within 30 years is 1/5 of the estimate presumed from claiming that the labour required is unique to each site and is cumulative and contemporaneous.

What is assumed but not empirically demonstrated is that each site remains continuously in use all through the relevant period and that the ceramic debris represents the continual rather than the episodic presence of people. If, by contrast, this is the signature of a seasonal and annual throughput population that is episodically using many sites over a given period of time then the total population was much smaller than the claimed aggregate in the paper. The issue is not how many sites are contemporaneous, ie exist, across a given period of time but the degree to which they are each being used in a given time span, eg a year, and how many sites are being used periodically, either by one group of builders or by episodic aggregations of otherwise very small groups of people to build one. It is a fallacious assumption inherent to property-owning societies that everyone who builds a structure then stays nearby thereafter and is associated with a particular structure. Numerous societies world-wide have not done so.

Depending on the degree to which groups are moving through multiple sites and the frequency with which they are building new sites, which they then use episodically over decades, the population estimates for any one period of time would differ substantially and would be much lower than estimates correlating a sum total, cumulative numbers of all builders for all sites within a ballpark period such as 30 years. The paper needs to incorporate how large those differences in estimates of total populations might be, because radically different estimates of food consumption and firing impact on vegetation among others, then follow.

General analytic issue

Finally, we wish to emphasise that the recent data on constructed sites coming out of several different regions of the eastern lowlands of South America is profoundly important, including the data on the sites in this paper. The new information is consistent with the characteristics of numerous “anomalous “ sites worldwide in very diverse environments, which demonstrate that conventional terms such as “urban” do not adequately encapsulate the diversity and different trajectories of

what are now many hundreds of sites. In addition, the story of the low-density settlement pattern phenomenon over the previous seven millennia is of serious significance for our efforts to understand the prospects of our present day, burgeoning, low-density, conventionally labelled industrial urban landscapes.
Just as recognising a previously unrecognised class of lifeforms is considered to be a profound intellectual contribution so likewise with these cultural phenomena which are being shoehorned into centuries old categories that no longer have a global, coherent definition. As we and our colleagues have pointed out in 2024 in Frontiers in Human Dynamics, there have been at least two earlier, different operational scales of low-density settlements each scale represented by numerous sites. The sites in both of these different spatial scales ceased to operate and neither scale of site developed into an order of magnitude larger form. The implications for our industrial low-density giant urban settlements are ominous. Note that our industrial low-density cities do not derive from agrarian low-density cities like Angkor, they only derive from smaller compact industrial cities. And the Classic Maya - Angkor scale low density settlements did not derive from sites resembling the oppida, the Trypillia sites and the recently reported Amazonian sites.
If differing types of settlement can all be blandly designated as a generalised category of low-density urbanism, then not recognising profound differences across differing scales of settlement magnitude has major implications is a worrying problem of scholarship.

Referee #6

(Remarks to the Author)
I co-reviewed this manuscript with one of the reviewers who provided the listed reports.

Version 4:

Reviewer comments:

Referee #2

(Remarks to the Author)
Reviewer 3 objects to the authors' overall population estimate for the Aquiry on the basis that multiplying the people needed to build a mound multiplied by the estimated number of earthworks does not give an estimate that large. However, total estimated population has to factor in both the estimated local mound totals during the peak time period of the occupation and the overall total of mounds found in the region according to the authors' new Lidar survey. In addition, the number of people required to build a mound is not the total of the community that they were part of, which would include as many children.

Furthermore, reviewer 3 finds fault with Parssinen et al. because their population estimate is higher than that of Hirst et al. (2025) based on Casarabe in the Llanos de Mojos. But Hirst et al.'s estimate is not based counts of habitations in the archaeological habitation mounds, since no one mapped houses and their hearths by ground-penetrating geophysical surveys and stratified-random test excavations at Casarabe as has been done for Marajoara mounds (Bevan and Roosevelt 2003; Roosevelt 1991, 2007). Thus, the site-population estimates for Casarabe don't actually count people directly and should not be used to rebuke Parssinen et al. for their estimates, also based on mound counts. And as Parssinen et al. point out in their response to the reviewer, the reviewer's comparison treats differently the counts of different types of mounds in the two regions.

In addition, Hirst et al. base their population estimate in part on the notion that the Amazon has a limited carrying capacity. Do some researchers really still assume there is a set carrying capacity for a habitat? I thought we were beyond that, given that Amazonia has shoved in everyone's faces the fact that at various times it supported occupations of dense, sedentary populations for long periods without evidence of significant deforestation. Most of us now understand that different technologies of production permit different carrying capacities and that different regions have different sediments, which affect their ability to support intensive production. In the Llanos, overgrazed Hispanic cattle pastures have replaced prehistoric earthwork field systems built for intensive agriculture in relatively poor soils. But the Aquiry has a range of soils considered to have good agricultural potential.

Moving on, reviewer 5 finds fault with Parssinen et al.'s use of the terms urban and civilization for the prehistoric Aquiry society. I've discussed at length in my earlier comments the many types of large, dense prehistoric occupations in Amazonia that have various types of permanent settlement. And anyone who has worked on a range of sites of complex cultures world-wide knows that the term urban is applied to settlements just as varied as the Amazonian ones I described (Graeber and Wengrow 2021).

But this reviewer's statements about the term civilization are patently inaccurate and makes one wonder how this reviewer could be so ignorant of recent theoretical discussions about civilization in archaeology and anthropology, as their statement quoted here, makes clear.

The reviewer claims "... there is no agreed definition or usage of the term. The French refer to Palaeolithic cultures as “civilisation” but that is not the conventional English usage. The term civilisation has also rather gone out of fashion in scholarly commentary and is primarily used only as a standard label eg Roman of Chinese civilisation. We do not argue that there is a proper definition of civilisation because there isn’t one. The usage in the paper of a regionally specific term and then claiming association to world-wide examples which purports reference to a global term that actually has no agreed definition is logically inconsistent."

In their statement about the issue of whether to call the Aquiry society a culture or a civilization, reviewer 5's statement is not accurate. It is not the case that Parssinen et al. are taking the initiative to push a "regional" culture as a civilization onto "world-wide" examples of civilizations. Researchers on world civilizations and cultures absolutely have used the term abundantly and recently, in addition to Amazonian researchers (such as Roosevelt 2024; Roosevelt and Guida Navarro 2021). The most recent, prominent, and persuasive discussion that reviewer 5 has managed to miss is in Graeber and Wengrow's 2021 book, The Dawn of Everything: A New History of Humanity. New York: Farrar, Strauss and Giroux. In it they extend the term civilization to the many prehistoric societies that have dense, often urban populations, civil rather than monarchical governments, complex religious systems, calendrics, intensive cultivation systems, large style horizons, widespread commercial and kinship networks, and more, as the Amazonian ones do. This book has had wide influence on researchers of these kinds of societies. How can a reviewer for Nature be so isolated from intellectual developments in anthropology as to be unaware of it?

This reviewer also takes issue with the use of the term "low-density urbanism" for Amazonian sites, referring to their co-authored article in Frontiers in Human Dynamics. They worry that low-density, as opposed to high-density urbanism, isn't sustainable because it is more vulnerable to climate change than compact urbanism. I'm not a big fan of the term "low density urbanism", but, as the manuscript authors point out, most archaeologists in Amazonia are using the term for the large, indubitably permanent mound settlements, like Casarabe and the Marajoara mounds, as well as for black-Indian-soil "garden cities" like Santarem Center and the many large black soil sites in its hinterland.

In most of the densely settled upland regions of Amazonia in prehistory, people used earth for construction, and they did not deforest but created house gardens and encouraged or planted trees for food and materials. Their refuse collected in deep deposits around settlements, affording sufficient enriched soil for permanent cultivation even on terra firma. Thus, abundant tropical rainfall (which is dependent on the recycling of moisture by forest vegetation, according to the measurements of Salati and colleagues and others) and moderate ambient temperatures are maintained. In contrast, in most modern, compact urban areas in the tropics, deforestation is advanced, increasing ambient temperatures and reducing rainfall by more than half, and inviting forest fires. Also, paving may be extensive, preventing infiltration of the remaining rains and increasing temperatures even further.

Reviewer 5, however, also doubts that in Amazonia settlement was permanent. They suggest that habitation sites might have been lived at episodically, reducing the possible estimated population for the occupation of the Aquiry mounds. However, the excavations by numerous researchers at a wide range Amazonian sites from c. 500 BC to c. 1500 AD shows that residence was indeed was often permanent. One has only to look at the stratigraphic profiles published for Amazonian and Orinoco mound sites to see that there is rarely a gap showing abandonment between house floors in occupation layers. The widespread and deep build-up of black-Indian-soils is further evidence of permanent settlement.

I don't see any evidence in the Amazonian pollen record for the episodic widespread forest burning they claim had gone on. Aside from clearing vegetation for the base of mounds, Amazonians then and today use fallen dead-wood for firewood; they don't cut down trees for hearth-fires. Charcoal is abundant in nearly all household refuse that has been excavated. It's the product of normal burning hearth-use, the occasional burning down of a house, and the discard of normal household food-preparation and cooking refuse. And if the reviewer doubts that black-Indian-soils are a product of permanent residence, they can go watch them forming even today in many permanent settlements in the Amazon.

I read their Frontiers article the reviewer cites and found it problematic in both methodology and causal reasoning. They don't define clearly what they mean by low or high density, though by reading their text one can see that what they mean is low- or high-density cities, not low- or high density regional settlement patterns. And they claim illogically that a small population is necessarily dispersed, thus low-density, while a large local population is necessarily densely settled. Of course, as Graeber and Wengrow detail, there were densely settled cities both small and large. But even though they criticize Parssinen et al. for not having habitation counts, Fletcher et al. don't employ habitation counts as a measure of density, either. They even use water tanks instead of habitations for their density map of Ankor, and give no evidence on the density of that city's residences, and whether they had low-density in terms of having gardens, fields, and forest between them, as Classic Mayan cities like Copan had and that Amazonian cities and their suburbs, like Santarem, had, or dense settlements as the urban core of Zimbabwe had.

Plus, their evidence that low-density agrarian settlements don't survive into modern cities, unlike ancient high-density ones is really flawed. San Salvador was a low-density urban city center for the historic Kongo "kingdom" (ruled by a king-queen pair, not by a king alone), and it survived and became a large modern city in Angola. Kumase, the capital of the Asante society, was a large, low density city, the Garden City of West Africa", yet it has evolved and expanded into a major population and trading center in Ghana today. Also, the Santarem ancient city center, a large low-density settlement with extensive suburbs, was never abandoned, either, but has evolved into a large modern city. Though it has a paved urban core of apartment buildings and town-houses, most of the residential population has years and gardens and outbuildings.

Furthermore, city states like the Sumerian ones were compact, like Mesopotamian and Anatolian Neolithic settlements before them, but they were not longer-lived than low-density hill-cities of the time. And the ancient Mesopotamian societies, like the Indus valley ones, salted in their fields with excessive irrigation and failed agriculturally, not because of vulnerability to climate change. So much for "the rugged endurance" of ancient high-density cities. And the Great Zimbabwe city center was a high-density settlement, not a low density one, contrary to the authors, yet it has no modern industrial city that evolved from it, of compact or dispersed settlement patterns. The causal arguments in Fletcher at al. thus are hardly definitive. Vulnerability of an ancient or historic society to climate change is only one possible reason for abandonment. In the cases

that they review, I see a range of other causes in evidence, such as re-orientation of commerce after technological change or change in trading partnerships or conquest by an outside society with different labor and production systems.

If Fletcher et al. are worried about climate change, then the low-density tropical forest indigenous settlement patterns of the Amazon are ideal models for the future, not ones to avoid. They preserve rainfall levels, moderate temperatures, and prevent soil erosion. Their anthropic forests are packed with species that have much value for food and materials but that have not yet been widely commercialized globally. On the successful model of Acai, such nutritious fruits as Caryocar and Moriche are just waiting to be developed as freezable purees to be sources of income for local populations and delectation by avid, first-world urban consumer populations.

References cited

Bevan, B. W. and A.C. Roosevelt
2003 Geoarchaeological exploration of Guajara, A prehistoric earth mound in Brazil. Geoarchaeology 18(3): 287-331.

Graeber, D. and D. Wengrow
2021 The Dawn of Everything: A New History of Humanity. New York: Farrar, Strauss and Giroux

Roosevelt, A.C.
1991 Mound-builders of the Amazon: Geophysical Archaeology on Marajo Island, Brazil. Studies in Archaeology. San Diego: Academic Press.

Roosevelt, A.C.
2007 Geophysical archaeology in the lower Amazon: A research strategy. In Remote Sensing in Archaeology, edited by Farouk El Baz and James R. Wiseman. New York: Springer. Pp. 435-467.

Referee #3

(Remarks to the Author)
Pärssinen et al. performed two LIDAR-based prospections. The first one, based on a 700-km-long flight, aimed at defining the extent of the Aquiry area. The second one consisted of flying over the Aquiry area along six parallel lines, 445 km long, 1 km wide, and 10 km apart.
By combining the results of the first LIDAR survey with the already known archaeology of the area, they estimate the Aquiry culture to cover 183,000 km². They describe the Aquiry as a "multicultural, complex society" made up of "different social and linguistic groups" that shared a common socio-cosmology and architectural style. I think this needs some elaboration. The main argument for saying that the Aquiry shared a common socio-cosmology is the construction of highly specific geometric architecture, which is basically what they call architectural style. I don’t really see that there is anything else than the shape of the earthworks in defining who the Aquiry people were. They exclude the Baures region because the ages are more recent than those from the Aquiry. However, when comparing the Aquiry to the people who built later mound villages in the same core area around the early 13th century, the authors note that there are no signs of radical population replacement because the people living in these later villages "used similar domestic ceramics" (no reference provided by the way) and shared similar subsistence strategies. So, why excluding Baures on the base of the ages? Is it because of the ceramic style? Also, when the only criterium is the architectural style of the earthworks, in what sense are Aquiry earthworks different from all the other similar earthworks found in the Amazon (Peripato, et al., 2023)? I would expect 1) a clear definition of the architectural features that set the Aquiry apart from other earthwork styles and 2) show that everything considered Aquiry fits the definition. Otherwise, in my opinion, the definition of what constitute the Aquiry is arbitrary.

Combining the data from the second group of flights (covering 2,380 km²) with the already known archaeology, they estimated the number and density of earthworks for the whole 183,000 km² area.
The first step of this estimation is based on multiplying all previously detected earthworks by five, because the LIDAR detected five times more earthworks than were detected in deforested areas by looking at optical satellite imagery. This is where my first concern arises. It is possible, and I would expect it, that the undetected earthworks in satellite imagery are smaller and/or of a different type than those detected only by LIDAR. This being the case, they cannot just multiply all the earthworks by five. For example, a rectangular geoglyph with a 4-meter-deep ditch is likely to be identified in both datasets, while a shallow canal or small road could have gone undetected on optical images. Therefore, while the number of earthworks is five times greater, the amount of habitational or ceremonial sites is not. And, most importantly, the amount of work needed for earthwork construction is not five times larger.
The second step consists of estimating the number of earthworks in between the 10-km-spaced LIDAR scans. They basically count how many earthworks are detected in a 1-km swath and multiply them by 10 to account for the whole area. This is where my second big concern arises. The average earthwork is 200 meters in diameter, meaning that a lot of earthworks are cut by the 1-km swath. See a clear example of this in their ED Figure 5, where a large circular ring which is only partially within the scan is counted (red point) as being within the swath. When they multiply the number of earthworks detected in one swath by 10, they are basically counting all the earthworks which are partially outside the swath twice. This results in a further overestimation of the number of habitational or ceremonial sites.

The third step consists of extrapolating what has been estimated in the second step to what they consider to be the high-density areas of the Aquiry, which cover 85,000 km². They do this by assuming that 1) the density of earthworks in forested areas is the same as in deforested areas. In supplementary table 2, they take the number of earthworks visually detected in optical satellite images, then they multiply this number by 5 (I already discussed why I don’t agree with this approach) and

then they multiply this number for the ration between forested over deforested area. First thing I don't understand is why they do this for sectors 1 and 8, for which they have the actual lidar scan. The have a measurement (3200 earthworks detected by lidar in Block 1), but they don't use it. They use the lidar data only to come up with a 5X factor that they apply to all the deforested areas, concluding that in Block 1 there are 5163 earthworks rather than the 3200 actually detected by LIDAR. The second thing is that they assume that the density of earthworks in forested areas is the same as in the deforested. But, it can be argued that deforestation follows the existence of roads and modern roads often follow the same path of ancient roads. In fact, McMichael et al. (2017) have shown that modern accessibility to areas of the Amazon correlates with the number of archaeological sites. They have the actual LIDAR for two blocks to test if this is the case or not. Their sensitivity analysis demonstrated that regions with denser forest cover experienced about a 10% drop in LiDAR penetration to the ground compared to more open regions, 10% less points shouldn't preclude the detection of large earthworks. However, they also state that "11.5% - 41.9% of the core area was determined to be impenetrable by laser". This second statement is in sharp contrasts with the previous one. Overall, I feel that they are not being clear with what happened with the LIDAR data in the forested area. These data should be shown, they should say how many earthworks they found in the forested areas of block 1 and 8. Despite claiming that their data come from LIDAR survey, in reality, LIDAR survey is only used (wrongly) to infer the factor 5. All the data used to come up with number of earthworks and population are based on already known number of earthworks in the deforested areas, multiplied by 5 and then extended to the whole area.

The overestimations of these three steps are cumulative, because each step makes a further overestimation of the previous overestimation, making their final number of 24,700 earthworks in the Aquiry region, and their claim that "this cultural area alone contains as many earthworks as previously estimated for the whole of Amazonia," unsupported.

In the second part of their manuscript, Pärssinen et al. estimate the population density of the Aquiry culture. They do this in two ways: through energetics (by estimating the amount of work needed to build all the earthworks they estimated to be in the Aquiry area) and ethnographically, by estimating how many people could have lived in what they consider to be habitational mounds.
The first type of estimation is just an arithmetic scaling of what the same authors estimated to be the population needed to build each geoglyph. This work they cite (Pärssinen, et al., 2009) is based on several simplistic assumptions, starting with: "If one agrees that one man or woman could move 1 m^3 per day". For example, they do not consider the huge differences in moving 1 m^3 when building a causeway vs. excavating a 4-meter-deep ditch or building a mound. However, they cite Hirst et al. (Hirst, et al., 2025). Here, Hirst et al. have done the exact same thing, estimating population density for the Casarabe culture by looking at the energetics of earthwork construction. But despite Pärssinen et al. knowing this paper, they don't engage with it. It is very apparent that Hirst et al.'s methodology is far more sophisticated and reliable than what Pärssinen et al. have done in their 2009 paper. I think they should use the methodology in Hirst et al. for two very important reasons. 1) Hirst et al. account for the energy and time involved in not only digging soil, but also transporting it, so that the number of people needed to build 1 m^3 of a mound is larger than the amount needed to build 1 m^3 of a causeway, for example. 2) Hirst et al. account for the timespan over which earthworks have been built. It is not the same to assume that the same number of earthworks has been built over 50 years as over 500 years; the first case would imply 10 times the population density of the latter. Pärssinen et al. don't engage with this temporal aspect at all. They estimate that about 25% to 60% of the total estimated Aquiry earthworks were in simultaneous use during a 200 year long window coming up with a figure of 4,360 to 10,460 earthworks that were co-existing/used during that 200-year peak. But use or co-existence doesn't imply any similarity with the amount of work needed for construction. They do state that the Aquiry built an average of 22 sites per year, but then they calculate the total population simply by multiplying a total of 4,360 to 10,460 earthworks (which represent earthworks used) by their estimate of 300 people (which is the number of people they said are needed for construction), concluding a population of about 1.31–3.14 million, in fact equating the energetics of construction with those of use and/or maintainace. In this way, they add another big overestimation to the previous overestimations.

They also estimated the population size by using ethnographic analogues in their paragraph titled "The Aquiry enclosures vs. later mound villages". Here, the average village had 11.75 multi-family mounds; each mound housed a minimum of 25 people; 11.75 multiplied by 25 yields approximately 294 (or roughly 300) people per village. But, in ED table 1 they say that the Aquiry earthworks are "Geoglyphs" and "Quad and multilateral embankment" and "mound sites" are classified under "other", meaning not Aquiry. How did they estimate how many habitational sites where Aquiry? At 300 people per village, we need between 4,360 and 10,466 Villages to come up with a figure comparable to the one based on construction energetics. But, 4360 to 10,466 is the total number of earthworks, not the number of villages, which is likely far smaller (they explicitly state that Aquiry earthworks were ceremonial, non-residential centres). Basically, I couldn't understand what calculations led them to say: "In every case, when comparing the possible population size of the later villages with communities that maintained earlier ceremonial and political centres, rather than the size of the structures, there is a match with our estimation of c. 300 people per average earthwork and c. 1.31–3.14 million people or more at the peak of the Aquiry." In my opinion this latest statement is also unsupported.

Peripato, V., et al., 2023. More than 10,000 pre-Columbian earthworks are still hidden throughout Amazonia, Science 382, 103-109.

Pärssinen, M., Schaan, D., Ranzi, A., 2009. Pre-Columbian geometric earthworks in the upper Purús: a complex society in western Amazonia, Antiquity 83, 1084-1095.

Hirst, J., Singarayer, J.S., Lombardo, U., Mayle, F., 2025. Constraining the population size estimates of the pre-Columbian Casarabe Culture of Amazonian Bolivia, PLoS ONE 20, e0325104.

Referee #5

(Remarks to the Author)
Thankyou for the work on the paper to bring the focus on to the population estimates. While I consider those estimate to be too high because each mobile group could built and use several features per year without overload given all we know about the readiness with which people can carry out construction logistics, I do not proposed to critique that issue at this time. That can wait for other occasions of debate and discussion.

There is, however, a statement in lines 288-91 which is problematic because it continues to claim that a comparison to other 'urban' settlements is meaningful by a reference to German settlements which are labelled 'urban' and have populations in the 100-1000 range. As mentioned before a region may use the label 'urban' that way but others may not so the case could only be made for the validity of a generalisation across continents, timespans and disparate cultures if it could be shown that this was a universal correlation of the label and specific population sizes. As is well known there are numerous different definitions, designations of urbanism and specifications for it.

The phrase in lines 360-61 also needs some change of terms. There may be many different concepts in use. The core issue is that there is no agreement about the many meanings attaches to the label 'urban'. That is point being made in the paper which is being referred to.

I wish the authors well with the publication of this paper. I consider that the new understanding of the diversity of settlement forms from research in Amazonia is profoundly important. I do not consider that its importance is conveyed or enhanced by forcing those diverse, remarkable phenomena into the old category of "urban. That may be awkward, but our job as researchers is to find, recognise and report what is not familiar, rather than seek to normalise it.

Open Access This Peer Review File is licensed under a Creative Commons Attribution 4.0 International License, which permits use, sharing, adaptation, distribution and reproduction in any medium or format, as long as you give appropriate credit to the original author(s) and the source, provide a link to the Creative Commons license, and indicate if changes were made.
In cases where reviewers are anonymous, credit should be given to 'Anonymous Referee' and the source.
The images or other third party material in this Peer Review File are included in the article's Creative Commons license, unless indicated otherwise in a credit line to the material. If material is not included in the article's Creative Commons license and your intended use is not permitted by statutory regulation or exceeds the permitted use, you will need to obtain permission directly from the copyright holder.
To view a copy of this license, visit https://creativecommons.org/licenses/by/4.0/

Point-by-point discussion, Nature manuscript 2025-04-08530

We would like to thank our referees for taking the time to review our manuscript **"Over 20,000 pre-colonial earthworks in the Southwest Amazonia"**. They asked us to clarify various points and posed many important questions. We have now made comprehensive modifications to the text, bearing in mind the suggestions provided by the referees. Due to the limited space, details of many uncertainties have also been explained and clarified in the Supplementary Information. Particular attention has been given to how the discovery of a surprisingly high density of ditched earthworks affects population estimations in our study area. We are thankful to the referees for their feedback, which has helped us improve the revised manuscript.

Below is our point-by-point response to the reviewers' comments and concerns. The referees' comments are in red and our answers are in black.

Referee #1

Although the manuscript reports many new earthworks, most of the additional results have already been reported elsewhere. The radiocarbon dates have been calibrated using the most recent SHCal20 calibration curve, but this isn't a novel result as it had already been presented by Kalliola et al. 2024. Indeed, extended Data Table 3 (which is a Figure, not a Table) seems to be mostly or entirely copied from Kalliola et al. 2024.

Most of the dating data originates from excavations carried out by our research team over the past 20 years. We are not presenting them as new findings, but rather to demonstrate that most geoglyphs were used within a specific period. These dates were not copied from the figure in Kalliola et al. (2024), which is based on the mean of each date calculated using a simple mathematical formula. Furthermore, in the figure in *Acta Amazonica*, only the horizontal bars labelled 'oldest' and 'most recent' were considered, with no allowance made for possible breaks. In our figure/table, however, each two-sigma date has been considered in its entirety alongside all breaks between dates. The mathematical mean has not been used, because the date may in fact be closer to the oldest or youngest date on the calibration curve. The only aspect of the presentation that follows Kalliola et al. (2024) is the appearance; similarly, the figures in the OxCal

models follow a typical OxCal appearance. Both have commonly been used in the archaeological literature. Following this introduction, we changed the presentation to the modelled OxCal version, as was preferred. Furthermore, we have now presented all dates using a mixed calibration curve to avoid suggestions that we could have copied the work of others.

Were all 162 dates (of which 14 newly reported) calibrated again and are they all presented in the Figure?

Not all 162 dates were presented in Extended Table/Figure 3. Only 96 dates relevant to ditched geoglyphs and roundish enclosures were included (as indicated in the text). Pre-geoglyph and mound settlement dates are not presented in the Figure. Those are only listed in Supplementary Table 1, because they are not relevant for the population estimation of the Aquiry. In the updated version of Extended Figure 5, we only presented a modelled summary of the dates of the ditched earthworks. The remaining dates can be found in the Supplementary Information.

It would be much better if the OxCal model (both code and output) were shown rather than just black/blue summary bars.

Detailed OxCal calibration data for all 162 samples, including laboratory code, pit, depth, material, B.P. date and calibrated date with two sigma, are presented in Supplementary Tables. The output is now clarified in a new figure using a Kernel Density Estimate (KDE) and Bayesian modelling, created for OxCal 4.4.4.

Could stratigraphies / maps be presented for Eletronorte I and Piçarreira, including positions of C14 dates?

Yes, we could, but we have been advised not to use too many pages for detail. We have provided information on the depth of each sample in the excavated pits (see Supplementary Tables 1a–e). Furthermore, some of us are preparing a separate article on the results of the excavations in Eletronorte I and Piçarreira, which has not yet been published. When dealing with specific archaeological sites, some of us have presented figures in other articles showing stratigraphy alongside ^{14}C dates, cited in the

manuscript (see Pärssinen et al., 2020 and 2021). However, most articles with published ^{14}C dates do not include this kind of data; they normally only provide a map indicating each dated pit. It would be problematic to obtain this kind of information on sites excavated by others (e.g. Dias, 2006). In any case, we have now included maps of Eletronorte I:1 and Piçarreira in the supplementary material, showing the location of the test pits.

What pretreatment was used for the charcoal and macrofossils of the newly reported radiocarbon dates?

Pre-treatment of Eletronorte I and Piçarreira samples were the following:

1. Visible root-fibres are removed.

2. 1 % HCl is added (10 h, just below the boiling point) (carbonates are removed).

3. 1 % NaOH is added, (10 h, just below the boiling point). The soluble part is precipitated by addition of concentrated HCl. The precipitate, which mainly consists of humus material, is washed, dried, and referred to as fraction SOL. The insoluble fraction, referred to as INS, is mainly consisting of the original organic material and should therefore provide the most re-liable age. Influence of contaminants could be obtained from the SOL fraction.

Prior to the accelerator determination of the 14C-content, the washed and dried material, acidulated to pH 4, is combusted to CO2 which is graphitised using a Fe-catalyst reaction. In the present investigation, fraction INS has been dated.

We could add this information, but we are not sure whether it would be relevant in this kind of listing, given that the radiocarbon analysis was conducted by the same prestigious laboratory in Uppsala, Sweden as was most of the other C14 dating in Supplementary Table 1a–b. Previous articles do not explain the pretreatments of other samples either, and we tried to respect the recommended article space. If necessary, however, we could include it.

Please use a consistent time-scale throughout - the Supplementary note mixes "calBC" and "calADs" with BP and "years ago".

In our system, BP dates refer to years before 1950 AD. Calibrated years (2 sigma, 95.4%) are marked as 'calBC' and 'calAD' to distinguish them from calendar years BC and AD. This system is used in Oxcal Programs, for example, and is accepted by journals such as Antiquity (see Pärssinen et al., 2020). If this system poses a problem for Nature, however, we will follow the editors' recommendations. We have now removed the expression "years ago".

The references to radiocarbon dating in the Supplementary note should point to 54-55, not 55-56.

This, as well as a few other similar errors, have now been corrected.

I fear that the supposed cosmological/astronomical significance of the structures is a case of over-interpretation (lines 57, 95, 96, 324). Certainly no consistent orientation of the features can be seen. The shapes of some of the structures are certainly interesting, but any links with cosmology/astronomy are tenuous at best and are not backed up by the three supporting citations. Kalliola et al. 2024's comments are speculative: "The reasons for these design choices remain unclear. These deviations may stem from challenges in accurate measurement during implementation, but they could also represent unknown manifestations of the builders' cosmology", with no further argumentation or evidence presented for any cosmological purpose. Schaan et al. 2012 nor Saunaluoma 2018 mention anything regarding shared cosmology or astronomy, but rather interpret the open areas as likely used for social activities such as parties.

There seems to be a misunderstanding of the concept of cosmology here. While in some disciplines, cosmology is related to astronomy, in the human and social sciences it refers not only to the sun, moon, planets and stars as mystical and spiritual entities, but also to ideas about the nature of the universe and the place and relations of humans with other beings in the world. In other words, it concerns perceptions of the world's origin, structure, and governing principles. In Indigenous studies, for example, the term 'cosmology' is preferred to 'worldview' (one of our authors belongs to the Apurinã nation). When Indigenous peoples share the idea that diverse beings are related by geometric appearances (Virtanen and Saunaluoma, 2017; see also Schaan et al., 2012) and construct geometric earthworks over large areas for their ceremonial centres, they

are indeed sharing a common cosmology. One of our researchers has extensive evidence on this matter. To avoid confusion, we now use the term 'socio-cosmology', as it considers the more-than-human social dimensions. References to astronomy in line 95 concern sun and moon symbols found in ceramics and geoglyph designs (Pärssinen, 2021; Kalliola et al., 2024). Also, the preference for using cardinal directions when orienting geoglyphs is statistically demonstrated in an article currently under review (Kalliola et al., 2025, Latin American Antiquity). We could add this reference as a submitted manuscript, but as it is not an important topic in this article, we removed the word astronomy from line 95 so that our explanation would take up too much space.

The potential ecological implications, as mentioned in the Discussion, are interesting (though have been mentioned before in previous publications by the authors and others).

Yes, as we and others have previously mentioned, the discovery of hundreds of earthworks in western Amazonia has significant ecological implications. However, this is the first time we have been able to define the main area of the geoglyphs by combining our earlier fieldwork with satellite studies and the results of the new LiDAR campaign, which allows us to estimate more accurately the scale and extent of the earthworks. The volume of earthworks appears to be greater in the 183,000 km^2 study area than the current estimate for the entire 6.7 million km^2 of Amazonia (Peripato et al., 2023). As our area represents only around 2.7% of the total Amazonian area, this finding is indeed interesting.

Although impacts on climate are mentioned (line 22, 363), this needs more explanation. How would a human-managed Amazon forest affect local, regional and/or world climate?

As the referee #2 says “the fact that humans managed to maintain forest cover by intensive agroforestry and gardening during this dense occupation is an important result of obvious utility for informing future land-use to maintain forest cover. Forest cover is essential in the rainfall cycle, as Salati and colleagues (1979) have shown earlier.” In our study, we refer specifically to global climate studies and models which used to suggest that the Western Amazonian tropical forest was pristine and that the presence of

charcoal indicated former dry periods. However, if Western Amazonia was densely populated thousands of years ago, and if people used fire while maintaining the main forest cover, then these climate models should be modified accordingly. We have therefore added a clarified reference to climate studies and future land-use planning in the text.

Referee #2

Areal definitions. The manuscript leaves the term "Aquiry" for the study area rather mysterious, just referring to two citatations, one of whose title indicates that it is in the Upper Purus River. In the interest of easing comprehension of the paper for readers without experience in the Brazilian Amazon, I think it would be good to define the term briefly when first mentioned in the text.

This suggestion has been considered, and the term Aquiry has now been explained in the new Supplementary Note 1a.

Furthermore, the paragraph on its geographical extension mentions variation mound distributions in areas with different rainfall patterns and amount of human-associated tree species in forests, but it lacks a final sentence indicating what is the significance of those variations for the research described in the manuscript? Only later on, in the next paragraph, do the writers say that their location of Lidar transects was designed to evaluate the extent of the Aquiry in particular. To me, paragraphs should end with a sentence that suggests what it has concluded and indicates how that leads to the next paragraph.

We incorporated this sentence into the text, but we ultimately had to remove the entire section from the manuscript due to restricted space.

Population estimates based on area of later prehistoric villages: The reference to use of the areas of 20 mound villages published by Iriarte et al. 2020 does not make clear how the number of houses were based on the areas of the villages. The authors should make that more clear.

We have now clarified this accordingly.

Furthermore, the text wasn't clear on why the 100 per hectare population density of medieval Europe is relevant? It would be OK to cite that figure as a comparison but not to base the population per hectare in the Aquiry.

This was intended as a comparison, but as it is not relevant, we removed it.

In the Northwest Amazon, an area much poorer agriculturally than all these other areas including the Aquiry, discrete villages usually are made up of a single house, but it is still a large, multifamily house, nonetheless (Koch-Grunberg 1909- 1910). Similarly, in a single-maloca Wai Wai village the resident population was 26 (Yde 1965).
Having myself geophysically mapped and test-excavated two mound villages on Marajo and reviewed a large number of earth mounds there of a period AD 500-900, immediately after time of the Geoglyph civilization, I think the houses associated with the Geoglyph mounds would more likely have been multifamily than nuclear… Perhaps calculating population using multifamily houses rather than nuclear-family ones would not make too much difference in the eventual regional population estimates, but, without any reason given in the article for choosing nuclear family houses as opposed malocas, why not just do the calculation on the basis of multifamily houses, as well?

This is a good and important point. There is no reason to use the lower estimate systematically, as we did earlier when we merely mentioned that multi-family malocas would also have been an option. Having now verified archaeological and historical records, we are convinced that the house foundations in mound villages do indeed refer to multi-family units.

Prehistoric land use. The authors cite Lombardo et al. 2025 in a sentence about different types of urbanism in Amazonia. However, that article is more about subsistence than urbanism and its take on subsistence is inconsistent with what the authors of the manuscript say about the subsistence system of the Geoglyph civilization… Perhaps the authors could clarify what they mean by citing this publication.

We are aware that Lombardo et al. (2025) present a different kind of subsistence practice among the Casarabe culture compared to the Aquiry. However, they also classify their case as a form of urbanism. Therefore, we refer to it as a relatively recent example of *different* or *several types of urbanism* in the Amazon region (compared to the traditional type and so-called forest urbanism).

In mentioning how modern agriculture and ranching land use in the area nowadays supports many fewer people than the estimated prehistoric population in question, the manuscript could clarify in a footnote that most of the agribusiness is managed for export and not for support of local populations.

OK.

Suggestions for future research dealing with presence of anthropic black soils.

This suggestion is welcome. Unfortunately, we have not yet found any Amazonian Dark Earth around the excavated earthworks in our research area. Only ash and tiny pieces of charcoal have been found. Dark Earth is present in the Juruá basin (Diaz, 1977–1980), but as far as we know, it does not belong to the Aquiry sphere.

Typographic errors. In reference note 12, Ubelager should be Ubelaker. For the reference to Iriarte et al. 2020, the note number is 19, not 20 as given in the text. On p. 23 of the manuscript on line 3 of the Acknowledgements, the word "administered" would be a more common English usage than the word they use, which is "administrated".

These errors have now been corrected.

Referee #3

The new data coming from the lidar survey are undoubtably interesting and valuable to all archaeologists and (palaeo)ecologists working in Amazonia. However, this new

dataset is only used to extrapolate the purported total number of earthworks, without providing any analysis regarding the type of earthwork.

This is not true. We are analysing both aspects and answering the questions regarding the total number of earthworks (line 221: 24,700) and the number of earthworks at the Aquiry (line 266: 17,430).

In other words, how many of them are mounds, enclosures, causeways, or other types of features?

No causeways were detected in our study area. Roads are only mentioned, not counted, in connection with the following types of structure: (1) ditched and embanked enclosures (geoglyphs); (2) quadrilateral and multilateral embankments; (3) roundish embankments; and (4) mound villages/settlements. These are the four types of earthwork structure relevant to this article. Some of us have a separate article on the roads in the referee process. As we note in the manuscript, ditched and embanked enclosures (geoglyphs) and quad and multilateral embankments are related and belong to the Aquiry civilization.

How are they distributed over the landscape?

Figure 3a shows the distribution of all the known earthworks. Figure 3b shows the distribution of the Aquiry earthworks only, indicated by green dots. Given the limited number of allowed figures, it is not relevant to have a specific map for mound settlements. Furthermore, Iriarte et al. (2020: Fig. 1) have published a map showing their distribution, which we have added a reference to.

What type of connections exists among different earthworks?

As stated in the manuscript, mound villages and roundish embanked enclosures are later than the Aquiry earthworks. Please refer to lines 59-60: "…radiocarbon analyses indicate that some earthworks in the area are much later mound villages and roundish embanked enclosures (post AD 1000/1200) [3,10,19]." This point is mentioned again, for the case, in lines 236-237.

How many cubic metres of earth were moved to build them?

In 2009, we estimated that around 8,000 m³ of soil had to be removed from a geoglyph measuring 200m diameter (Pärssinen et al., 2009). This was a modest estimation, considering that, of the geoglyphs we have excavated so far, the original bottom of the ditch was often 4–5 metres below the surface. However, the shape of the ditch also affects the calculations. In a few cases, for example, the last two metres have been quite narrow. LiDAR and satellite images do not resolve these issues. Therefore, we are reluctant to speculate further, especially since we know that some of our colleagues are planning to publish an article on this topic. Additionally, we think that more excavations would be needed to make reasonable estimates. In other words, we do not currently have enough data to do so well enough.

They listed the new sites discovered in a table in supplementary information where there is a “Aquiry” column, indicating whether the earthwork meets the definition of Aquiry structure—but they don’t actually define what constitutes an “Aquiry” structure.

Again, this is not true. For example, lines 254–256 state: “Of the total of 347 earthworks in our six parallel LiDAR lines, we have 128 ditched geoglyphs and 118 embanked quad or multilateral enclosures that all can be associated, as said, to the Aquiry civilization (Extended data Table 1a.).”

In the same table, they have another column called “accuracy” with a binary code for “good” or “somewhat good”, without specifying what “somewhat good” means. This also begs the question of whether the authors have “bad” data, or simply don’t report it.

We have explained the table as follows: “Aquiry indicates whether the earthwork meets the definition of an Aquiry structure: yes (y), no (n), and accuracy indicates the clarity of outline recognition in size measurements, classified as good (g) or somewhat good (s).” To clarify the “fuzzy” concepts of “good” and “somewhat good”, we have now added a reference to Kalliola et al. (2024), in which these concepts are explained and applied. The clarity of outline is "good" when the size is seen and measured exactly. When part of the earthworks could not be seen clearly (for example, due to thick

vegetation or because part of the earthwork was outside the LiDAR image), the entire size was estimated according to the form of the earthwork (quadrangular, circular, oval, etc.) with “somewhat good” accuracy. If the form could not be seen well enough due to thick vegetation or another reason, it was not registered at all.

They don’t enumerate how many sites are consistent with the “Aquiry” definition nor how many are “somewhat good”.

Again, this is not true. All Aquiry structures are marked as 'yes' (y) in Supplementary Table 2. Also, the accuracy of their size in hectares is classified as 'good' (g) or 'somewhat good' (s). The classification of Aquiry earthworks according to relevant blocks 1 and 8 are listed in Extended Data Table 1a. These and the other Aquiry earthworks are also shown in Figure 3a.

Likewise, they don’t describe what are the implications of these categories; are the “non Aquiry” sites and/or the “somewhat good” sites excluded from the analysis?

The difference between Aquiry and non-Aquiry sites is essential for our analysis of the population, as explained in the text. To reiterate: Aquiry sites are:
(1) ditched and embanked enclosures (geoglyphs); (2) quad and multilateral embankments. Non-Aquiry sites are (1.) roundish embankments and (2.) mound settlements. Sites with 'good' and 'somewhat good' size accuracy have been included in the analysis, as can be seen by reading the text. We firmly believe that this fuzzy concept is fully adequate to describe the level of certainty agreed upon by at least two professionals.

Basically, from a very interesting and novel dataset they only extract the number of sites to be used in the subsequent extrapolations, without saying if—and what type of—data filtering was applied.

As mentioned, this is not true. Data filtering has been carried out and is explained in the text.

The most important information that is lacking is the relative proportion of ceremonial versus habitational sites.

Unfortunately, as explained in the text and as referee 3 must be aware, no settlements dating back to the time of the geoglyphs have yet been found. Therefore, this question cannot be studied. In archaeology, it is common for ceremonial or funeral sites to be identifiable from one period but not another, and vice versa for settlement sites. In our area, all the known mound villages date from a later period. The Aquiry settlements are likely to have been spread out around the ceremonial sites, as among the Maya (Isendahl and Smith, 2013), as they have not been found in low-density areas or outside the Aquiry area either. Mounds can be seen in the vicinity of geoglyphs in many satellite and LiDAR images without an indicative plaza, both individually and in groups. However, we cannot determine whether these mounds are natural or man-made, due to a lack of permission to investigate these potential sites. However, given that Indigenous oral tradition and corresponding ceremonies in the Amazon seem to have deep roots and have endured for a long time, we have a good idea about how many people were required for the most important ceremonies in each ceremonial terrain. This information is included in the text. Furthermore, although Aquiry settlements are unknown and, in general, comparisons between settlement and ceremonial sizes are not recommended (Curet 1998), we can compare the average population size of communities that maintained ceremonial sites with those of communities that established mound villages. This has now been done.

At various points the authors speculate that their number of detected earthworks is an underestimate due to the possibility that the forest canopy was too dense and not enough ground points were obtained. This should not be speculation; places where this would be a problem can and must be identified from the raw lidar point cloud data.

Given our understanding of how LiDAR technology works, it is clear that variations in forest canopy density affect the ability to detect earthworks. Problems can occur everywhere: when some trees are flowering, laser penetration towards the ground is lower, whereas when some trees are dropping their leaves, penetration is generally better. However, massive leaf dropping may also affect laser penetration at ground level, etc. Similarly, the structure of earthworks affects visibility: geoglyphs with deep

ditches and high embankments are easier to detect than eroded embanked enclosures. Furthermore, as we wrote in the manuscript, earlier studies indicate that the canopy tends to be thicker in Labrea than in Rio Branco (MMA, Ministry of the Environment, 2006). While it would be possible in theory to check the penetration of the approximately 140 billion point clouds obtained metre per metre, this enormous task would not change the amount of earthwork that has already been calculated as certain. At best, it would indicate areas of low penetration in June 2024, but it would not reveal whether additional earthworks were present or not. Nevertheless, we have now conducted a preliminary analysis by dividing our ALS area into two parts, Fuso 19 and Fuso 20, and taking various ALS point cloud tiles from both halves for analysis. After over two weeks of computing and modelling, it appeared that the impenetrable terrain area was between 11.3% and 41.9% in Fuso 19 (west of the UTM 66W longitude), and between 17.8% and 54.5% in Fuso 20 (east of the UTM 66W longitude). These results have now been added to the Supplementary Note, and they are considered in the main text.

By far, the most shocking number they present—which if true would warrant publication in Nature—is the population density they calculate for the area. Based on the numbers they put forward, the core Aquiry area would have had a population of 28 people/km2, which is almost 6 times the modern density population in the Acre.

This is an odd comparison. As far as we know, all textbooks on the methodology of comparison demand that the units of comparison should be comparable. In fact, we mentioned that at its highest point, the Aquiry area had a population density of 12.5–17 people/km^2 (2.5–3.5 times higher than the current population density of the entire state of Acre, to demonstrate its potential). Furthermore, as referee number 2 says, most agribusinesses today are managed for export rather than to support local populations. However, referee 3 begins to compare the core area of the Aquiry with the entire area of Acre, highlighting a significant difference between the two. The methodological problem with this statement is that the core area and the whole area are not comparable units. Using the same flawed logic, one could compare the population density of the capital city, Rio Branco, to that of the core area of the Aquiry.

and [population] far higher than estimates for early Neolithic (0.6p/km2); Iron Age (1.2 – 2.3p/km2); or the Roman Period (14.4 p/km2) (Zimmermann et al. 2009, Human Biology).

Here, the same unconventional logic continues. For example, the referee presents a population of 14.4 p/km² for the Roman Period without saying that it refers to all European provinces (2.5 million km²), and it is in fact the same as we have estimated for the whole Aquiry (183,000 km²). According to Scheidel (2007: table 3.1), in Roman Italy (310,000 km²) the population density was 26-29/km² or even 39-42/ km².

The authors not only interpolate their mound density estimates to areas between their transects—which is appropriate—but they extrapolate these estimates to the entire region. This is grossly inappropriate because the boundaries of the region are only based on a few radiocarbon ages, and phenomena such as population densities are expected to be highly spatially autocorrelated.

We were able to date the use of different types of earthworks accurately by analysing 162 radiocarbon samples. Furthermore, boundaries were drawn using earthwork types from around 1,700 examples, not just the 162 dates. In addition, we are now refining the dates by dividing the samples into groups based on their geographical origin, specifically the Amazonas, Acre, and Bolivian regions. We are then using Bayesian methods to analyse these samples. As previously mentioned, most of the geoglyphs were used between 100 and 300 AD. In terms of regional distribution, all sites in the Amazon and Bolivian regions date back to this period. In the case of Acre, 62% of sites (8 out of 13) date back to this period. Although the proportion is even higher in Amazonas and Bolivia, in accordance with the precautionary principle, we have used a threshold of 60% as the maximum. Even the KDE activity modelling, which does not calculate continuing activities from earlier periods (confirmed by radiocarbon dates), indicates that 61% of geoglyphs were constructed between 250 BC and 400 AD. This has now been added to the Supplementary material. In general, the definition of high-density areas is based on a 20-year review of new forest clearings, nearly 1,300 real archaeological sites found by satellite imagery, and 400 sites found by our current LiDAR data. These are not blind estimates. Furthermore, applying proportionality to the

difference in detection accuracy between LiDAR models and satellite images (factor 5) directly to confirmed high-density areas produces the same result.

The authors blindly assume that forested areas of each block should have as many monumental structures as unforested areas; the reasoning for this is never made clear.

As most blocks have more forested than unforested areas, we do not expect to find the same number of earthworks in both areas. In fact, we expected the amount of forestation in an area to have no effect on the general estimate of the entire block. Why should it affect the estimate when large empty areas, as far as we have seen, only exist outside the core area? The core area block scanned by LiDAR includes both forested and unforested areas, as well as conglomerates and small empty spaces. Our earlier studies of satellite images and field confirmations have shown that the same is true in the extrapolated areas, and this has been considered. We consider that statistical area coverage of 10% of the LiDAR Block 1 is sufficient to extrapolate the results to six similar areas within the Aquiry sphere. These six blocks include 913 previously identified earthworks. Although the total LiDAR sample coverage is only around 1.5%, this is still statistically significant when compared with the 0.08% coverage of the entire Amazon region in the Peripato et al. (2023) LiDAR data, particularly given that the quantity of earthworks detected in each block via satellite imagery has been considered in our analysis.

At various points they argue that the present forest community composition is in large part predicated on the presence of ancient human habitation; thus, unless modern deforestation is truly random to this type of patterning, we cannot assume as such.

Many things have affected the deforestation processes, but basically the direction in which it has been continuing during the last 50 years has been quite random. Nevertheless, the core area in general has clearly been a target of farmers and ranchers in Brazil for its fertility. Furthermore, the Brazilian government's economic projects since the late 1960s have included road construction that has affected the distribution of contemporary non-Indigenous populations in the area. Large-scale cattle ranching, on the other hand, has its own logic regarding the selection of areas for deforestation that does not necessarily follow the logic of productive areas for planting. Contemporary

soil fertilization techniques and current environmental legislation, as well as political and economic pressures, also affect the deforestation pattern in these areas. Therefore, current patterns have distinct logics, including from a socio-cosmological perspective when compared to those of Indigenous populations. On the other hand, the ethnographic research shows how Southwestern Amazonian forests have largely been stewarded and managed by the ancestral populations. This is also evident in stories, in addition to forest management and land use practices. Phytolith and botanical studies have also shown that the modern forest composition in the geoglyph area is a result of pre-colonial impact. For example, Watling et al. (2018:135) summarise: "Along with the earthworks themselves, these forests, where they remain, are part of the landscape legacies left by the geoglyph builders."

There are two major problems with their population density estimates. The first concerns how they arrive at this number. They make an extrapolation from another paper, also led by Pärssinen (reference 1). There, there is a 220 words paragraph, from which we copy here the most relevant portion:

"It is estimated that, in order to build one geoglyph of 200 m diameter, c . 8000 m3 of soil had to be removed. If one agrees that one man or woman could move 1 m3 per day, 8000 individuals per day or a workforce of 80 individuals for 100 days would be necessary to do the job. Considering that there is also a necessity for people to do domestic work and provide food and shelter for the workers, we could be talking about a population of at least 300. It is clear that more people could do the same job in less time, and that fewer people could do it in more time. Even though, considering a population of 300 people per geoglyph, it would be possible to envision a regional population of 60 000 people, which indicates a fairly dense population living in a terra firme area."

Here, not a single reference is provided for such an estimate, but, more importantly, 60 000 is the number of people needed to build the 200 sites they identified in 100 days! But the geoglyphs were built during a period of hundreds of years. Therefore, the estimated population is orders of magnitude lower than what they claim to be. Even assuming that the speculation of 300 people per geoglyphs is correct, in order to estimate the population density, they need to estimate how many geoglyphs were built per year. The simplest way to do it is assuming constant population density and dividing

the number of geoglyphs by the number of years during which the Aquiry culture existed.

These are statements by the reviewer are inaccurate. In our manuscript, we refer specifically to the recent population estimation for the Southern Rim region of the Amazon (De Souza et al., 2018). Based on the presence of 1,000–1,500 earthworks, they estimated the population to be 0.5–1 million (lines 43–45). After estimating that the total number of earthworks in our area, which forms only part of the Southern Rim, is 20 times higher, these numbers would lead to an unacceptable population size (lines 225-227). This is one of the main points. We then briefly refer to the article by Pärssinen et al. (2009), which covers the same topics cited by referee 3 in relation to the estimation of 200 earthworks and a total population of 60,000 (lines 230–234). At that time, we were aware of only one radiocarbon date, c. AD 1283, and the calculation was performed on the basis that the 200 earthworks were constructed and maintained within a short timeframe. Therefore, the statement that "not a single reference is provided for such an estimate" is incorrect. Two hundred earthworks, each maintained by around 300 people, would make for a total population of 60,000.

The referee asks us estimate how many geoglyphs were built per year to estimate the population density adding: "The simplest way to do it is assuming constant population density and dividing the number of geoglyphs by the number of years during which the Aquiry culture existed." For us, it would be a serious error to use our limited space for such speculation. First, it is not important how many geoglyphs were built per year thousands of years ago. As the referee 3 is also referring to the European towns in the connection to population density, he/she or the authors he/she cites do not give numbers either how many towns there had been built per year. As we note on lines 293-295, in the 15th Germany there were only 3,000 towns and cities, but now we must add that we have not seen in demographic calculations any estimation how many of them were built per year when occupied in that particular century. Simply, it is not relevant for our argument and what we are showing. Once built, they were maintained for hundreds of years as ceremonial centres in the Aquiry area or as places of worship and trade in those German towns.

Secondly, it is more important to know how many places of encounter/earthworks were used at the same time. In Germany, it was expected that all 3,000 towns and cities would be in use simultaneously in the 15th century. However, it would be erroneous to assume that the same population density would have been maintained in Germany or the Aquiry civilization for around 1,500/1,600 years. As we have demonstrated in our manuscript, up to c. 60% of the dated earthworks were in use around AD 200 in the Aquery sphere, and many fewer were in use in 500 BC or AD 700. Stable population density is not a reliable indicator over such a long period of time. This is why we are using our 162 radiocarbon dates — a relatively good and normal amount for this kind of article — to specify when population density was at its highest.

To avoid these kinds of comments, we have now made important revisions to the manuscript to clarify our position on these topics.

The plot with radiocarbon ages they present in ED Table 3—underlying the argument that the geoglyphs were constructed and/or in use from 100/150 to 350/400 250 AD—fails to include any confidence intervals. There is no explanation of how the uncertainty derived from the radiocarbon measurements and the calibration is taken into account. Based on partially-overlapping probability distributions for dates from fifteen sites, the authors claim 60% overlap in occupation. Overlap probabilities must be calculated with a Bayesian approach. Just because the probability distributions for dates from two sites overlap does not imply that the occupations were coeval, rather that there is the possibility that they are contemporaneous.

What is said in this paragraph is slightly different to what was said earlier by referee 3, but it is easier to understand. In our manuscript, we have taken empty intervals into account and present the reader with the 2-sigma (95.4%) confidence intervals in the Supplementary Information. Nevertheless, we agree that it would be useful to incorporate Bayesian modelling into the analysis. Therefore, we have now added a Bayesian approach and KDM modelling with probability densities to the manuscript.

Likewise, given that they argue that the scale of habitation is unprecedented, the number of dated sites is wholly inadequate to extrapolate at such a scale.

What is adequate and what is inadequate is relational. We have collected dates during more than 20 years, and normally we have been authorized by IPHAN [Brazil], INAR [Bolivia] and landowners to excavate only a few sites, with permissions valid for one to two years. By combining the results of those excavations with the results of our colleagues, we have 32 sites, 36 earthworks, and 162 radiocarbon dates. Before our LiDAR flights, 1,279 structures and some 850 sites were known. Thus, our 32 sites represent c. 3.5% of those known sites in the extended area from the Brazilian states of Acre, Amazonas, and Northern Bolivia. We think it is statistically adequate for preliminary estimation – and at least, it is the best possible.

It could take another 20 years to double the number of dated sites, which seems like an unreasonable request. Furthermore, it should be noted that the master sequence of Andean chronology, created by John H. Rowe in 1963 and still in use today, was developed over 60 years ago using far fewer radiocarbon dates. Alternatively, if we compare the partially contemporary Chavín civilisation in Peru with the Aquiry, we can note that its chronology was based on a long-term analysis of five sites and 44 dates by Burger (1981) and was later redefined by Rick et al. (2009) using the results of the long-standing Stanford Project and others. They accumulated 75 dates from Chavín de Huántar and 71 from six other sites – a total of 146 dates, 45 years after Rowe. At least we have many more dated sites than these, even though referee 3 considers our number "wholly inadequate."

For what we know, the geoglyphs could have been built, as they state in line 76, between 750/700 BC and AD 900/950—i.e during a period of 1600 years. If we take their number of 300 people needed to build a geoglyph in 100 days to be valid, then, in 1600 years, 513 people alone (not 3 million!!) are sufficient to build all the 20 000 geoglyphs they claim to have found!!!

The referee seems to recommend his/her own calculation how many geoglyphs were built per year in the 183,000 km² area (omitting all the results of the C14 analysis except the starting and ending dates). As we noted earlier, for us, it is relevant to estimate how many of those earthworks were in use simultaneously in AD 100 - 300. Instead, the idea that a group of 513 people moving around would have built all those earthworks in the Amazonas, Acre, and Bolivian regions over 1,600 years, is not an adequate way to base

a population estimate. Revisions to the manuscript have now been made to avoid confusions and “recommendations” of these kinds.

The second major problem with this estimate of three million persons is that they don’t discuss at all the huge implications in terms of food procurement and environmental carrying capacity. In the discussion, the authors claim that identifying cultivated crops in the archaeological record sidesteps any carrying capacity estimates. It does not; it only shows what may have been consumed but not what the maximum cultivable quantities were.

In fact, we are referring to some previous generalised carrying capacity estimates, which are currently being criticised by numerous research results. Our article presents the results of one of the largest systematic archaeological airborne laser scanning studies conducted in Amazonia to date. A full analysis of the carrying capacity is simply not possible due to lack of space. With corresponding references, we only mention that agroforestry was maintained for the semi-domesticated Brazil nut and many semi-domesticated and domesticated palms, and maize and squash were also cultivated. Watling et al. (2015) also mention the following: “Given that the earthwork builders were engaged in agriculture, it is highly likely that other domesticates not identified in the phytolith record were also being grown and consumed. Potential accompaniments [for maize and squash] include manioc, sweet potato, chilli pepper, beans, peanuts and cotton.” The presence of dust and tiny pieces of charcoal –without so called Amazonian Dark Earth (ADE) - in our dated test pits made outside Severino Calazans earthwork indicates that these openings were used for agriculture. These openings sometimes occupied areas of up to 3–5 km² (Pärssinen and Ranzi, 2020), but no systematic studies have yet been conducted. Watling et al. (2017:1872) also cast doubt on earlier theories regarding the importance of the presence of ADE, questioning “whether quantification of forest burning should play such a central role in determining areas of high versus low populations.”

Furthermore, a new study of soil fertility in the Amazon region, which we have now included in our manuscript, confirms that samples from the geoglyph area in Brazil are among the most fertile in the entire Amazonian *terra firme* (Zuquim et al., 2023). This came as no surprise, as all the farmers we consulted confirmed that the soil in geoglyph

areas is highly fertile and ideal for growing maize, which also explains the recent boom in the conversion of hundreds of thousands of hectares of deforested land for industrial-scale maize cultivation in the ancient Aquiry region. The variety of crops cultivated in the Aquiry area is also impressive, as detailed in the 16th and early 17th century Spanish accounts (see the summary in Pärssinen et al., 2021: 313–314). This indicates a long and impressive history of mixed agricultural and agroforestry practices. Thus, it is clear that the area, containing both *terra firma* and fluvial *varzea* zones, has the potential to support a population up to three million or even more.

Even if we consider the lower end of their population density estimates, 12.5 people/km2 (or 0.125 people/ha), they would have had to cultivate the whole area—and in a pretty intensive way. Nowadays, an indigenous family needs at least 20 hectares for slush and burn agriculture plus a far larger area for hunting and fishing, wood harvesting, construction materials, medicinal plants and other quotidian necessities. If the entire landscape were under cultivation, we would expect to see evidence of agricultural features everywhere. Instead, not a single productive system has been identified by the lidar survey—or at least none that they report. Previous phytoliths analysis conducted in and around geoglyphs show that the immediate surroundings of the geoglyphs were forested and that people practiced agroforestry (Watling et al, 2017 PNSA), not intensive open field agriculture. Pärssinen et al. should explain how agroforestry could have supported 3 million people for 300 years in an interfluvial area. There is no way this could have happened.

The numbers from extensive ethnographic work related to Amazonian Indigenous peoples' swidden sizes are completely different from the views of the reviewer. Furthermore, the immediate surroundings of the geoglyphs were open for several hundred metres to several kilometres (Pärssinen and Ranzi, 2020). Furthermore, as Watling et al. (2017) present, large-scale slash-and-burn cultivation is mainly a post-conquest practice (Arroyo-Kalin, 2012). Instead, they argue that another kind of agroforestry and agriculture was practised in the pre-colonial geoglyph period.

If the forest was indeed full of domesticated and semi-domesticated trees, as well as other species in forest gardens and almond groves, why would they burn it? Moreover, it is a well-known fact that no evidence of massive burning or Dark Earth formation has

been found in the Aquiry sphere. It is also important to note that an Indigenous scholar is part of our research group, and one author has collaborated with Indigenous peoples in the area for over 20 years. They are aware that a current Apurinã family in the geoglyph area requires between one and three hectares (depending on family size and other factors) to survive for one year, rather than 20 hectares. According to colonial sources on Peru, the typical "chacra" field of a family was similar in size at around 1 hectare, while the largest ones were around 3 hectares. Thus, 60 families of 300 people could be supported by 60 hectares of agricultural land. They do not need 1,200 hectares for that.

Given the 27–28-year life cycle of bamboo in the area (McMichael et al., 2014; Virtanen et al., 2022), it is possible that the fields were put into use by taking advantage of the typical mass death of bamboo, whereby trees and leaves are burned during the dry season to fertilise the fields. Using traditional methods, manioc produces an average yield of 5–7 tonnes, but when grown alongside corn the total yield can be 1.5–3 tonnes higher. For instance, when fertilised with human manure, fish heads, and other food waste, the yield can reach 10 tonnes per hectare. This would be sufficient for one family on 0.30 hectares, especially given that the nearby forest is full of cultivated and semi-cultivated palm fruits and palm kernels, which are nutritious, as are the Brazil nuts from the orchards. However, the area is often left to rest after three years of use and then re-cultivated after six years (if the field has not been fertilised in the meantime), in which case close to one hectare is required in total. After three complete cycles on three plots, the bamboo dies off again, making it easy to increase cultivated area or change the area under cultivation. In our revised versions, we also refer to our ethnographic material that, as said, does not support the reviewer´s view of 20 hectares needed for an Indigenous family.

Finally, it is important to mention that the expansion of manioc and peach palm farming is said to be associated with the expansion of polychrome ceramics (Neves, 2008). Polychrome ceramics first appeared in the Aquiry area some years before the Common Era, coinciding with the peak of geoglyph ceremonies (see Pärssinen, 2021). The presence of a root like manioc is difficult to find in archaeological records, but the presence of both domesticated and wild peach palm nuts, along with many other species, is confirmed in the Tequinho geoglyph site (Pärssinen et al. 2021). Also,

Sancho Pizarro, among many others, saw manioc and maize fields, among 'other roots and fruits', on his expedition to *tierra firme* at the turn of the years 1560 and 1561, south of the Amazon River (Vázquez de Espinosa, 1629: 287).

If the entire landscape were under cultivation, we would expect to see evidence of agricultural features everywhere. Instead, not a single productive system has been identified by the lidar survey—or at least none that they report. Previous phytoliths analysis conducted in and around geoglyphs show that the immediate surroundings of the geoglyphs were forested and that people practiced agroforestry (Watling et al, 2017 PNSA), not intensive open field agriculture. Pärssinen et al. should explain how agroforestry could have supported 3 million people for 300 years in an interfluvial area. There is no way this could have happened.

One should not expect to easily see agricultural fields from the air in Amazonia, except in the areas that are flooded annually and where raised fields, canals and causeways have been constructed, making them visible. In fact, even many embanked earthworks were only visible to us in LiDAR images after extensive processing. Some great river floodplain sections, such as those of the Purus and the Madre de Dios, were part of the Aquiry sphere, but agricultural earthworks have not been located even there. So far in our area, the land use practices during various millennia have only been demonstrated through archaeological excavations and soil studies in some sites (Watling et al., 2015, 2017; Pärssinen et al., 2020, 2021). Nevertheless, these studies firmly indicate that intensive open-field agriculture was practised alongside intensive agroforestry and gardening with various domesticated and semi-domesticated species. There is no doubt that the Aquiry people were capable of this. It is time to abandon previous theory that the poverty of Amazonian soil is an important limiting factor everywhere. We must remember that less than 25 years ago it was considered impossible to find any evidence of a complex society in the *terra firme* of Amazonia. Even Betty Meggers, one of the main supporters of the limiting factor theory, was totally surprised when the discovery of various geoglyphs in Acre was published in English in 2003 (personal communication with MP and AR in 2003). Our understanding of Amazonia is evolving.

The population estimates are fundamentally flawed. Even if corrected, the findings would still depend on questionable extrapolations rather than direct measurements. The

lidar findings—while interesting—are not noteworthy enough for publication in Nature given the plethora of lidar studies published recently in this and comparable journals.

Almost all population estimates of past societies are subject to uncertainty, because direct measurement is impossible. Therefore, direct measurement cannot be the starting point for such studies. Nevertheless, as Denevan (2014) once observed, "the size, distribution and demise of the native population are key issues in understanding pre-colonial cultural development and its environmental impact on the Amazon region." Discussions about the Amazonian population have been ongoing for a long time without direct measurement. Following the discovery of a society that built geoglyphs in the southern rim of the Amazon, the number of geoglyphs and other earthworks in the region has been discussed more widely (e.g. Peripato et al., 2023), and this has been taken into account when estimating population densities in Amazonia (Denevan, 2014; De Souza et al., 2018). Our LiDAR survey and actual measurement on 10% of Block 1 have already raised 35% our earlier estimated amounts of earthworks in the Aquiry area. Furthermore, comparing the visibility of earthworks in LiDAR images with a combination of different satellite images shows that the human eye missed approximately 500% (a factor of 5) of earthworks in open areas that can be detected in LiDAR images. This discovery alone will quintuple the amount calculated for the open core area, even though only 22.5% of the core has been deforested.

We haven't seen any signs that the earthwork distribution in those still forested areas (77.5% of the core in blocks 1-7) would be completely different from the mosaic of the deforested areas seen in satellite images. Therefore, we have good reasons to estimate more than 20,000 earthworks for the whole area. This radical conclusion was obtained from our LiDAR survey, which was originally planned in 2018 - 2019 and carried out in 2024. This may be shocking to some, but this discovery will obviously affect future population size estimations in the Amazon – even if better ways of estimating population density are applied in future. In any case, our LiDAR survey, as well as other important recently published surveys, demonstrates how radically LiDAR technology is changing our understanding, especially in forested areas. It would be extremely unfortunate if these kinds of LiDAR survey results were not published, simply because of the large number of LiDAR studies recently published in this and comparable journals.

Fortunately, referee 3 also writes, "By far the most shocking number they present — which, if true, would warrant publication in Nature — is the population density they calculate for the area." As mentioned, our population estimates are a consequence of the unexpected number of earthworks detected during the survey. Firstly, they radically changed earlier estimates of the number of earthworks in the entire Amazon region (published in Science), and secondly, they radically changed population density estimates based on the number of earthworks, especially in the Southern Rim of the Amazon region (published in Nature). Relatively much higher estimates of the relationship between earthworks and population size have previously been presented. Our estimation is more modest than some of these – only the number of earthworks was surprisingly high. This is why we have amended the text to clarify what the results are and how they will affect recent estimations of the earthworks and population density in Amazonia.

Referee #5

There is a serious issue that purported large civilisations and big populations have been proposed before (in the 1930s and 40s) - such as the Azanian civilisation in East Africa - on the basis of massive amounts of earth-moving. In that case, several thousand square kilometres of mountain terracing on a spectacular scale was presumed to indicate large numbers of people and high levels of organisation. But the human capacity to move soil and rock is prodigious and the logistics of the human ability to do so is well-known eg see Erasmus for Mesoamerica and see Acabado on the Ifugao terraces in the Phillipines. Large populations and hierarchical states cannot be assumed.

Yes, we are aware of these cases and agree with the referee. Nevertheless, when making a comparison, it is important to bear in mind that the enormous terraced constructions in these areas were realised by the local people over long periods of time to meet their subsistence needs. Their function was not the same, and these were not built all at once, unlike the individual Acrean geoglyphs, which were constructed for ceremonial, political, and social purposes. These ancient terraced projects demonstrate that no state hierarchy was needed to construct them. If they are dated accurately, they can also be

used to calculate carrying capacity. In Amazonia, comparison could be made, for example, with the carrying capacity of raised fields. Unfortunately, these are lacking in our area.

The authors point out early on that the geoglyphs were used and made by mobile communities which were not resident in the enclosures.Then later talk of sedentary scattered communities but with no information about occupation within the enclosures or an empirical demonstration that the users were either mobile or sedentary.

Yes, the geoglyphs appear to be ceremonial centres with no evidence of permanent settlements. This suggests a dispersed surrounding population (Kalliola et al., 2024: 10/21), somewhat like those found in Maya centres (Isendahl & Smith, 2013: 135). Mobility between sites appears to have been common, with similar geometric principles shared across a wide area. As we explained to referee 3: “In our area, all the known mound villages date from a later period. The Aquiry settlements are likely to have been spread out around the ceremonial sites, as among the Maya (Isendahl and Smith 2013), as they have not been found in low-density areas or outside the Aquiry area either. Mounds can be seen in the vicinity of geoglyphs in many satellite and LiDAR images without an indicative plaza, both individually and in groups. However, we cannot determine whether these mounds are natural or man-made, due to a lack of permission to investigate these potential sites.”

There is also the fact that the visitors of geoglyphs ate at least maize and squash, and almost certainly manioc, sweet potatoes, and some other agricultural products. There were also fruits (and palm hearts) from domesticated peach palm and many other semi-domesticated forest tree species, such as Brazilian nuts, leading us to think that the people were attached permanently, or at least semi-permanently, to their geoglyph sites and maintained mixed agriculture and agroforestry around them.

They also show in the figure of the group of geoglyphs some geoglyphs had been overlaid by others and that some geoglyphs are juxtaposed which could mean either that they are complementary or one supercedes the other. These two basic pieces of information mean that the number of geoglyphs being built at any one time and in use at any one time (two different circumstances) is not readily apparent. The brief remark that

about 60% were in use at one time - over a few hundred years - does not resolve that issue. What is needed is a map of the distribution of the C14 dated sites. Are they clustered in one area or widely distributed?

These are good points. While most sites have only one earthwork, some of the more interesting sites have two or three earthworks near each other, often connected by a road, which probably indicates that they were used at the same time (although one or two of these may be somewhat older). Our radiocarbon dates suggest up to a 60% probability match that earthworks were used contemporaneously in these sites at the beginning of the Common Era. In our dated sites (n = 32) and in the sites seen in the LiDAR images (n = 406), it is rare (less than 5%) for more than three ditched structures to be patterned near each other by connecting roads or to be constructed closely together. Superposition also occurs, but this is an even rarer occurrence (making up less than 2% of our LiDAR data). This can sometimes indicate renovation (e.g. Cruzeirinho) or the later use of an earlier structure (e.g., when a later mound settlement was established on the same site, as at Fazenda Colorada). Finally, of the sites included in this LiDAR sample, two have three to five structures established around an embankment. One or two later mound settlements may also be located within or outside the same conglomerate (see Extended Data Figures 3a and 3b). This relatively rare type only exists in the vicinity of Rio Branco. Unfortunately, we do not have any radiocarbon dates for this type of earthwork conglomerate. Only one date exists for a later mound settlement inside Dois Circulos: Estrutura IV (Iriarte et al., 2021). Further details on these cases can now be found in the Supplementary information section.

The samples have always been divided between three regions according to the states in question: Amazonas in the north and Acre in the centre (both in Brazil), and Bolivia in the south. However, this division has now been refined more clearly. We have also produced new OxCal maps showing the widespread distribution of dated sites in these three states between 250 BC and AD 400.

Do the dates come from one site size or are distributed across the size range. That requires the actual site size distributions by local area and in aggregate not just an average and a large value. This then needs to be integrated with the data on the number

of superpositions and juxtapositions to provide an estimate of potential contemporaneity of use.

We have now compiled information on the sizes of the dated earthworks collected by us and our colleagues. The radiocarbon dates obtained from the geoglyphs range from 0.35 to 49 hectares in interior size. Further details can now be found in the Supplementary information.

The density of occurrence of the geoglyphs also needs to be specified within the likely dating ranges.

The likely dating ranges are now specified. Furthermore, to better highlight the most probable years of the peak in our Oxcal calibration, both chronologically and regionally, we have now divided the area more clearly into three regions for which we have radiocarbon dates. The Acre dates come from the *terra firme* area between the Acre and Abunã rivers, to the east and south of the capital, Rio Branco. The dates from Amazonas come from the south-east of Boca do Acre, near the main channel of the Purus River. The Bolivian dates come from an area near the confluence of the Madre de Dios and Beni rivers, to the east and south of Riberalta. The distance between Boca do Acre and Riberalta is approximately 300 km as the crow flies. Furthermore, we have used a Bayesian boundary model for each region and summarised the radiocarbon data using a Kernel Density Estimate (KDE) with a KDE_Model created for OxCal 4.4.4. Changes in population growth or decline processes are a topic for another article.

At a rough estimate about 70-80 geoglyphs might have been made per year at the peak time in the denser area - distributed across 85,000 sq km. They might have all been in one place but if they were not this is sparse activity. These issues need to be investigated as a basis for population estimates. Statistical analysis and specification of uncertainties is needed.

We are now using Kernel density estimation to identify the period during which most of the earthworks were constructed, including uncertainties. However, the focus of this article is not on the number of earthworks constructed per year, but rather on the peak period during which the largest number of earthworks were used simultaneously,

bearing in mind that some geoglyphs were in use for as long as 1,000 years. To achieve this, we also used summarised KDE phase modelling to estimate the periods of highest chronological and spatial overlap. To be clear, in this article, we are not studying population growth or decline processes in the same way as Klassen et al. (2021) did in the medieval Greater Angkor region. This topic will be covered in a future article.

For information on "earthmoving" the authors should look to analyses in Mesoamerica and in North America (eg the Hopewell sites).

Yes, we are aware of those studies. In fact, we have been exchanging information with the researchers for 20 years. We will try to include one or two references.

For the population estimates, extrapolating from residential settlements centuries later is problematic. They are residential which the geoglyphs have not been shown to be in this paper. And using those later estimates introduces a retrodictive fallacy.

Yes, we are aware of this problem. On the other hand, although cultural traditions change throughout human history, certain cultural and social structures, practices, and belief systems tend to endure over long periods of time, which our colleagues in the French Annales School have demonstrated. This is why it is better to find explanations and data for comparison from the very same area than from Mesoamerica or North America, for example.

Considering the ceremonial versus residential issue, we agree that the size of those different sites is not comparable due their different functions. Nevertheless, comparing the population size of the later villages, not their spatial size, with that of the dispersed communities around each geoglyph who constructed each earthwork, may give us an approximation of the population of an average ceremonial site. Furthermore, the tradition of organising annual ceremonies for one's community and invited guests from outside is still very much alive among various Indigenous Amazonian peoples living in the ancient region of the Aquiry. This tradition has a long history. These communities still consider geoglyphs to be sacred sites for ceremonies. Our aim is to present this more clearly.

In relation to densities the association to European urban densities is problematic because urban settlements globally operate across a large spectrum of densities. Conventional compact urbanism is not even a description of urbanism is the 20th and 21st centuries. And the Classic Maya and the Angkorian urban settlements operated at low-densities. Greater Angkor, the most extensive city of the agrarian world with 750,000 to 900,000 people had an overall density under 10 p/ha. There is no standard urban density.

We agree that there is no such thing as a standard urban density. In fact, European urban cities were not intended to be a basis for comparison, but rather to help the reader to understand more about Western tradition by showing that medieval towns were not large either. Furthermore, European medieval towns were quite autonomous compact units, where agricultural fields and forest resources were located outside the walls, as was the case with ditched and embanked geoglyphs. As far as we know, this was not the case with Greater Angkor. In any case, we have now modified and clarified this issue.

In conclusion, the paper does not rigorously make the case for the claimed large populations. However, the authors possess a superb set of spatial data. The use of appropriate logistical analyses and rigorous spatial and temporal modelling is required to provide a probability assessment of the regional pattern which the authors are seeking to analyse.

This has now been done, as much as the data reasonably allows.

Analytic procedures have been recommended above. The authors need to provide rigorous references for population estimates which will substantially include referencing key studies of logistics and labour populations.

This will be a focus of a future study. There is simply no space to elaborate on this issue in this manuscript.

The paper is clearly written but the presentation of the proposed population estimates is hard to follow because is spread over several parts of the paper and needs to be consolidated so that the components of the case can be readily accessible.

We have now added more subheadings to help readers follow the presentation's overall logic.

References

Arroyo-Kalin, M. Slash-burn-and-churn: Landscape history and crop cultivation in pre-Columbian Amazonia. *Quat. Int.* **243**, 4-18 (2012).

Burger, R.L. The Radiocarbon Evidence for the Temporal Priority of Chavin de Huantar *Amer. Antiq.* **46**, 592-602 (1981).

Curet, L.A. New formulae for estimating prehistoric populations for lowland South America and the Caribbean. *Antiquity* **72**, 359-375 (1998).

Denevan, W. M. Estimating Amazonian Indian Numbers in 1492**.** *J. Lat. Am. Geogr*. **13,** 207-221 (2014).

De Souza, J.G. *et al*. Pre-Columbian earth-builders settled along the entire southern rim of the Amazon. *Nature Communications* **9**, 1125 (2018).

Dias, O. Relatórios de pesquisas nos estados do Acre e Amazonas, PRONAPABA. Manuscripts in the files of IPHAN, Brazil (1977-1980).

Dias, O. As Estruturas Arqueológicas de Terra no Estado do Acre - Amazônia Ocidental, Brasil. Um caso de resiliência? in *Estudos Contemporâneos de Arqueologia* (eds. Dias, O, Carvalho, E. & Zimmermann, M.) 59-168 (Palmas: Unitins, IAB, 2006).

Iriarte, J. *et al*. Geometry by design: contribution of Lidar to the understanding of settlement patterns of the mound villages in SW Amazonia. *J. Computer Appl. Archaeol.* **3**, 151-169 (2020).

Iriarte, J., de Souza, J.G., Robinson, M.E., Barbosa, A.D. & da Silva, F. Refining the Chronology and Occupation Dynamics of the Mound Villages of South-eastern Acre, Brazil. *Amazônica, Revista de Antropologia* **13**, 153-176 (2021).

Isendahl, C. & Smith, M.E. Sustainable agrarian urbanism: The low-density cities of the Mayas and Aztecs. *Cities* **31**, 132-143 (2013).

Kalliola, R., Pärssinen, M., Ranzi, A., Seppä, I. & Barbosa, A.D. Geographies of ancient geometric earthworks and their builders in Western Amazonia. *Acta Amazonica* **54**, e54hu22351 (2024).

Kalliola, R., Pärssinen, M., Ranzi, A. & Barbosa, A.D. Ancient Amazonian Earthwork Roads: Unveiling Ceremonial, Livelihood, and Networking Significances. *Submitted manuscript.*

Klassen, S. *et al.* Diachronic modeling of the population within the medieval Greater Angkor Region settlement complex. *Sci. Adv.* **7**, eabf8441 (2021).

Lombardo, U. *et al.* Maize monoculture supported pre-Columbian urbanism in southwestern Amazonia. *Nature*, online 29.1.2025. https://doi.org/10.1038/s41586-024-08473-y3

Neves, E.G. Ecology, Ceramic Chronology and Distribution. Long-Term History and Political Change in the Amazonian Floodplain, in *Handbook of South American Archaeology* (eds. Silvermann, H., & Isbell, W.) 359-379 (New York: Springer, 2008).

McMichael, C.H., Palace, M.W. & Golightly, M. Bamboo-dominated forests and pre-Columbian earthwork formations in south-western Amazonia. *J. Biogeogr.* **41**, 1733-1745 (2014).

MMA Ministry of the Environment. *Vegetation Coverage Maps of Brazilian Biomes: Amazon Biome* (Brasília: MMA Ministry of the Environment, 2006).

Pärssinen, M. Tequinho Geoglyph Site and Early Polychrome Horizon 300 BC – AD 300/500 in the Brazilian State of Acre, *Amazônica: Revista de Antropologia* **13**, 177-220 (2021).

Pärssinen, M. & Ranzi, A. Mobilidade cerimonial e a emergência do poder político com as primeiras estradas conhecidas do oeste amazônico (2000 A.P.), in *(I)Mobilidades na Pré-história: Pessoas, recursos, objetos, sítios e territórios,* (eds. Vilaça, R. & Simas de Aguiar, R.) 307-349 (Coimbra: Coimbra University Press, 2020).

Pärssinen, M., Schaan, D. & Ranzi, A. Pre-Columbian geometric earthworks in the upper Purús: a complex society in western Amazonia. *Antiquity* **83**, 1084-1095 (2009).

Pärssinen, M, Balée, W., Ranzi, A. & Barbosa, A.D. The geoglyph sites of Acre, Brazil: 10 000-year-old land-use practices and climate change in Amazonia. *Antiquity **94**,* 1538-1556 (2020).

Pärssinen, M., Ferreira, E., Virtanen, P.K. & Ranzi, A. Domestication in motion: Macrofossils of pre-colonial Brazilian nuts, palms and other Amazonian planted tree species found in the Upper Purus, *Environ. Archaeol.* **26**, 309-322 (2021).

Peripato, V. *et al*. More than 10,000 pre-Columbian earthworks are still hidden throughout Amazonia. *Science* **382**, 103-109 (2023).

Rick, J.W. *et al.* La cronología de Chavín de Huántar y sus implicancias para el Periodo Formativo. *Boletín de Arqueología PUCP* **13**, 87-132 (2009).

Rowe, J.H. Urban Settlements in Ancient Peru. *Ñawpa Pacha* **1,** 1-27 (1963).

Salati, E., Dall´Olio, A., Matsui, E. & Gat, J.R. Recycling of water in the Amazon Basin: An isotopic study. *Water Resour. Res.* **15**, 1250-1258 (1979).

Schaan, D. *et al*. New radiometric dates for precolumbian (2000–700 BP) earthworks in western Amazonia, Brazil. *J. Field Archaeol.* **37**, 132-142 (2012).

Scheidel, W. Demography, in The Cambridge Economic History of the Greco-Roman World (eds. Scheidel, W., Morris, I. & Saller R.P.) 38-86 (Cambridge: Cambridge University Press, 2008).

Vázquez de Espinosa, A. Compendio y descripción de las Indias Occidentales. *Biblioteca de Autores Españoles* **231** (Ediciones Atlas, [1629] 1969).

Virtanen, P.K. & Saunaluoma, S. Visualization and movement as configurations of human–nonhuman engagements: precolonial geometric earthwork landscapes of the Upper Purus, Brazil. *Am. Anthropol.* **119**, 614-630 (2017).

Virtanen, P.K., Apurinã, F., Ruokolainen, K. & Manchineri, L. The Role of *Guadua* Bamboo in Land Management and Indigenous Perspectives on Bamboo Ecosystem in Southwestern Amazonia. *Hum. Ecol.* **50**, 1077-1088 (2022). https://doi.org/10.1007/s10745-022-00376-8

Watling, J., Saunaluoma, S., Pärssinen, M. & Schaan, D.P. Subsistence practices among earthwork builders: Phytolith evidence from archaeological sites in the southwest Amazonian interfluves. *J. Archaeol. Sci. Rep.* **4**, 541-551 (2015).

Watling, J. *et al.* Impact of pre-Columbian “geoglyph” builders on Amazonian forests. *PNAS* **114,** 1868-1873 (2017).

Watling, J., Mayle, F.E. & Schaan, D. Historical Ecology, human niche construction and landscape in pre-Columbian Amazonia: A case study of the geoglyph builders of Acre, Brazil. *J. of Anthropol. Archaeol.* **50**, 128-139 (2018).

Zimmermann, A., Hilpert, J. & Wendt K.P. Estimations of Population Density for Selected Periods Between the Neolithic and AD 1800. *Hum. Biol.* **81**, 357-380 (2009).

Zuquim, G. *et al.* Introducing a map of soil base cation concentration, an ecologically relevant GIS-layer for Amazonian forest. *Geoderma Regional* **33**, e00645 (2023). http://doi.org/10.1016/j.geodrs.2023.e00645

Point-by-point discussion, 29.12.2025 (manuscript 2025-04-08530A)

We would like to thank our referees for taking the time to review our manuscript **"Over 20,000 pre-colonial earthworks in the Southwest Amazonia."** Below is our point-by-point response to the reviewers' new comments and concerns. The referees' comments are in red and our answers are in black.

Referee #1

The Bayesian modelling presented in this revised manuscript is a useful approach to summarizing the radiocarbon-based estimate of the beginning and end of the Aquiry phase, as well as identifying periods of activity within the Aquiry Phase and for individual sites (KDE models; Extended Data Fig. 5). Several of the sites also contain evidence for previous and subsequent archaeological activity.

We are pleased that the referee #1 understands our methods and considers our Bayesian modelling and KDE models adequate to identify periods of activity within the Aquiry phase in general, as well as for individual sites in particular. This approach provides valid radiocarbon-based evidence for estimating both the period of time when the Aquiry geoglyphs were most actively constructed and the proportion of the geoglyphs that were in use. These estimates were essential in our attempt to provide a justified figure for the number of people living in the area during the peak period.

The usage of a mixed NH-SH calibration curve makes sense.

We agree. Although recalibrating every sample took a lot of time, the result was the most accurate possible.

I'm not so convinced that the KDE activity timings could really be translated into space (Supplementary Fig. 3). Perhaps this Figure could be left out without detriment to the arguments made.

Supplementary Figure 3 has now been included in the manuscript because Referee #5 required us to incorporate it into the chronological and spatial analyses, writing: "*What is needed is a map showing the distribution of the C14-dated sites*."

I agree that there is no need to provide much detail on the pretreatment of the charcoal samples, however please do cite a charcoal pretreatment paper (e.g., one of Bird et al. 2014, Gillespie 1997, Olsson & Kilicci 1964, or Wood 2015).

We thank Referee #1 for the observation, and the suggested citation is now incorporated into the revised manuscript.

Otherwise I'm happy with the responses to my comments.

line 312, "1,31-3,14" should be "1.31-3.14".

We thank Referee #1 for the constructive comment.

Referee #2

REVIEWER #2's NOTES ON THE REVISED PARSSINEN My characterizations of the key results, significance, and originality of the manuscript are as I wrote them in my original review. Briefly, the results are highly significant and robust, as they are based on a large, new database as well as all relevant earlier results by the authors and by others. The methodologies used for gathering the mapping data and the analyses employed are valid and well described. I found the authors' characterization of the uncertainties excellent (and unusually honest). I base my evaluation of the research on my many decades of research in Amazonian archaeology, ethnohistory, and ethnology as well as in tropical ecology.

The authors' answers to the reviewers' questions about radiocarbon dates and the added supplementary information about them and other details seem satisfactory and, in fact, improve the manuscript. Minor errors, such as typographic errors, have been corrected. The authors' term, Aquiry, for the Geoglyph area as now defined, has been clarified, as a reviewer requested. A few sections about which reviewers had questioned the organization of the writing have now had to be removed to limit the length of the paper. Those are all satisfactory outcomes, for me.

Considering that the referee #2 appears to be probably the best-known living specialist of Amazonian archaeology and anthropological history, well-known for her knowledge of Amazonian research traditions and contemporary research, we are glad to read her satisfaction with our revised manuscript and the final results.

The issue raised about "astronomical orientation" has been satisfied by editing the terms used in the article. I would like to mention, however, in support of the authors' interpretation of geometric images as elements of a shared cosmology, that such images used as symbols for astronomical bodies considered by current Indigenous Amazonians as important figures in myths of creation, are present in Amazonian rock art as early as c. 13,000 calendar years ago, in Monte Alegre, Para, Brazil.

Yes, it was somewhat disappointing that we could not expand on this topic due to the limited space. Nevertheless, the article in which some of us present statistical data on the 'astronomical orientation' of Amazonian geometric earthworks has now been accepted for publication. In the future, we are also willing to study more about links for astronomical bodies in ceramics, body paintings, rock art, etc., as well as in Indigenous oral traditions.

The authors are correct to mention, in response to the question by reviewer #3 about the possible effect of a well-forested prehistoric Geoglyph area, that Salati and his colleagues and others have shown that at least 75% of the rain in Amazonian forests derives from moisture stored and respired by the forest itself, not by moisture coming in from oceans, contrary to many researchers' expectations. If the Geoglyph makers followed the widely demonstrated current Indigenous

Amazonian practice of maintaining heavy forest cover through use of agroforestry techniques, then rainfall would have been much more regular and consistent than it is in now deforested or "savanna" parts of Amazonia. Thus, the lesson of the Geophyphs, as presented by the authors, is one very relevant to the current and future of Amazonia as a habitat.

Yes, indeed. We are impressed by the reviewer's understanding of our research findings on the relatively high density of ceremonial earthworks and the sustainability of the livelihood strategies of past Indigenous communities. These findings must certainly be taken into account when planning future land and forest use, as well as climate research.

I agree with the authors that reviewer #3's questions are in some cases based on an inaccurate grasp of the original manuscript and also on speculations that, although interesting, seem beyond the subject of the manuscript and in any case that would not enhance its relevance for Nature. In any case, the authors make several useful clarifications to address most of reviewer #3's more relevant comments and questions. I feel the need to mention that reviewer #3 seems to be influenced by some early assumptions about Amazonia necessarily having a limited carrying capacity because it is a tropical forest. Many of we archaeologists who work in Amazonia have done conclusive empirical research of various kinds that shows that the environmental limitation theory of Amazon prehistory has not been upheld. Furthermore, that reviewer seems acquainted only with Indigenous slash and burn cultivation, whereas numerous Indigenous peoples as well as acculturated Amazonian people (the majority of whose DNA is Indigenous, showing that despite no longer speaking Indigenous languages, they are long-term inhabitants of Amazonia) get most of their calories from tree crops, whether domesticated, cultivated, or wild. As recent researchers, whom the authors refer to in their response, show in various ways, permanent cultivation and harvesting of trees and shade-tolerant annuals and perennials, whether in terra firma or floodplains, was the main subsistence activity, supplemented by fishing and hunting. This type of land use has definitely been shown for sites in the Geoglyph region. Furthermore, reliance on forest utilization for a major part of subsistence would not leave any

particular type of landform such as what the reviewer seems to expect. As an archaeologist an environmental anthropologist, I do not find the population estimates of the authors "shocking" but, as I mention in my review, they seem consistent with those I made based on the sites I have surveyed and excavated in other parts of Greater Amazonia, some of which I mention in my review. I should also mention that my review of studies of pollen results carried out for both prehistoric and modern periods show no indication of wide-areal deforestation in prehistory. The percentages of grass that modern deforested or "savanna" areas have do not occur in prehistory in the places that have been studied (E.g., Roosevelt 2021 and Absy 1979).

All of these issues are highly important and provide valuable additional information for our discussions with Referee #3. Our own research results from diverse disciplinary backgrounds in the Aquiry area fully support these latest findings.

As for reviewer #5, who questions how population estimates can be made when the sites mapped are primarily ceremonial, one can point to the pattern of ceremonial meetings among living Amazonian societies, such as the female initiation rites among the Shipibo of the upper Amazon in Peru or the funerals of the Kayapo and neighboring groups in the middle Xingu. Both groups were very populous during the prehistoric period, as Myers shows for the Shipibo and their neighbors and as Posey has shown for the Kayapo. For these events, as I know from attending some of these ceremonials, people are invited to come from many residential communities in the surrounding area to central places, where they spend several weeks dancing, feasting, wrestling, trading, etc. Thus ceremonial constructions like the Geoglyphs would represent meeting places for a number of residential villages. Thus, making population estimates is a valid exercise, and the authors have made different estimates based on different possible community and family house sizes. The reviewer praises the results of the authors' Lidar mapping program and the statistical analysis, and the authors have satisfactorily addressed his/her suggestions, I think.

Signed

A. C. Roosevelt

As Referee #2 has internationally recognised in-depth knowledge of population estimation methods in the Amazonian context, as well as of Amazonian societies' deep past until the present time, we are grateful for these positive comments.

Referee #3

This manuscript presents two main results: (1) the quantification of the number of geoglyphs, and (2) the implications of this quantification in terms of estimated population density. While the first part represents "hard data," and our previous comments have been partially addressed through clarifications and improved analyses, the second part (see the paragraph entitled Estimation of the population in the revised manuscript) remains purely speculative and unsupported.

It seems the referee #3 considers his previous doubts to have been clarified in the first part of our manuscript, which presents the results of our LiDAR survey in terms of the number and size of earthworks. However, Referee #3 still objects to the latter part of the manuscript, regarding it as purely speculative. In our opinion, the referee´s comments on this matter are based on significant misunderstandings and misreading of the Amazonian context, as well as some irrelevant comparisons and statements, which we would like to address.

Their population estimation relies entirely on their own publication (Pärssinen et al., 2009), where they assert—without any supporting evidence—that building a geoglyph with a diameter of 200 m would require 80 individuals working for 100 days. This provides a metric linking the number of earthworks constructed to the amount of labor over time.

We appreciate the reviewer's point, but Pärssinen et al. (2009) clearly stated that their estimation was based on field-measured dimensions of a geoglyph, implying that 8,000 m^3 of soil was moved and that 1 m^3 of soil can be moved per day by one person. The estimate in Pärssinen, Schaan & Ranzi (2009) was based on the fact that, in any construction work, one person can dig approximately 1–2 m^3 per day with an iron spade

and hoe. The calculation presented was also based on late Denise Schaan's practical experiments, which were conducted at various excavation sites over several years and in different months. People in Schaan's experiments used iron hoes and spades instead of wooden tools. Therefore, we believe that our estimation of the number of people required to create ditched enclosures at these sites must be considered an absolute minimum. In addition, we would like to emphasise that our aim was to estimate the size of an average community that built and maintained a geoglyph, not just the number of people that was directly involved in the construction work. In a thriving community that could have both built and continuously maintained a geoglyph, there must have been adults performing daily domestic tasks in agroforestry, gardens, and cultivated fields as well as by fishing, hunting and gathering with those who prepared food for the workers. Additionally, one must consider that a community necessarily comprises elders, young people, children, breastfeeding mothers, and others who cannot contribute as much as healthy adults do. As such, the result was roughly an average of 300 people per geoglyph. Finally, to support the idea that our 2009 estimate was a minimum figure, we cited a 2018 estimate published in *Nature* which suggested that 500–600 inhabitants were required for each earthwork in the Southern Rim of Amazonia (De Souza *et al.* 2018). This second estimation was based on ethnographic evidence and a linear regression model.

As a response to reviewer's criticism that we had based our population estimate entirely on one paper and one line of logic, we would like to point out that we wrote the following on lines 284–286: "*Another [third] way to estimate the number of people in the pre-colonial Aquiry is to consider how many people lived in a typical mound village when these settlements first appeared in the same area in the early 13th century*", and we summarised the results of this comparison on lines 286–316. A more detailed presentation of this comparison can be found in Supplementary Information, lines 175–206. Between these two different archaeological phases, there are no signs of radical population replacement, but rather a clear continuity (see also Iriarte *et al*. 2021). The number of individual mounds (representing multi-family *maloca* dwellings) surrounding a plaza can be easily calculated using satellite imagery. These villages may offer the best indication of the size of the dispersed communities that chose to live together somewhat later in the core area of the ancient Aquiry. Therefore, by comparing population estimates for the mound phase with those for the earlier geoglyph phase, we

concluded that population density during the mound period was approximately 235 people per hectare. However, the average size of the mound villages was 300 people per earthwork, and these occupied a smaller spatial area than the geoglyphs. This provides independent evidence regarding the approximate size of the communities that constructed and maintained the geoglyphs.

A fourth piece of evidence supporting our argument and conclusion is the amount of space Indigenous communities currently require for traditional ceremonies in the same region, for which we have strong first-hand ethnographic evidence (lines 317–326). These ceremonies require an open space of one to two hectares (approximately 1.5 hectares for 300 people) and ceremonial roads. Hence, it can be supposed that the average 3-hectare geoglyph was planned for a maximum of 600 people. If around 300 of these people belonged to their own community, the remaining 300 were probably intended for visitors from other communities, as various historical and ethnographic sources suggest. Anna Roosevelt (Referee #2) also refers to the same point in the Ucayali region, Peru in her assessment. Thus, it is reasonable to assume that inviting more visitors than there were in one community would have posed a considerable threat in the event of a conflict. Finally, we presented a speculative and unlikely theory that if the mounds created by the large *maloca* dwellings were residences for individual families, the figure would be 60 people per earthwork. Thus, it is not adequate to claim that our estimation relies entirely on our own publication of the year 2009 without any supporting evidence.

However, in response to our main comment, the authors now claim that “it is not important how many geoglyphs were built per year.” Moreover, in the manuscript they state: “To calculate the population of the Aquiry, the first obvious question is: how many earthworks were in use at the same time?” (line 252, revised manuscript) and “if an average earthwork required 300 people to build and maintain it, we arrive at a total population of about 1.31–3.14 million” (line 276, revised manuscript). I strongly disagree with conflating the number of people allegedly required to build the earthworks with those needed to maintain them or with those who might have used them simultaneously. These are three fundamentally different types of

activities. This confusion between metrics is repeated both in their responses to us and to Referee 5 (who raised the same concern), where they write that "the focus of this article is not on the number of earthworks constructed per year, but rather on the peak period during which the largest number of earthworks were used simultaneously." Here again, they shift from discussing the number of people needed for construction (the figure provided in their own paper) to discussing "simultaneous use" and "maintenance." Estimates of the number of people associated with each of these activities should be based on distinct and independent lines of evidence. Yet there is absolutely no supporting evidence for how they estimate the number of people who would have simultaneously used or maintained these geoglyphs. I strongly disagree with conflating the number of people allegedly required to build the earthworks with those needed to maintain them or with those who might have used them simultaneously. These are three fundamentally different types of activities.

In our opinion, Pärssinen et al. (2009) cannot be considered a valid reference for estimating past population densities, as it lacks any robust supporting argument; and even if the estimation in Pärssinen et al. (2009) were reliable, the metric used there concerns construction time per unit of labour. Therefore, it is essential that the authors consider, based on radiocarbon ages and their chronological model, how many geoglyphs were constructed per year...

Of course, the construction, ongoing maintenance, and use of earthworks are all separate activities that the communities of the Aquiry have carried out, even if they are partly overlapping. For example, based on the estimates in the 2009 article, if all the working-age men (one per family) were among the 300 community members, then around 60 men and 20 women (one per three families) would have to dig. They would work a minimum of 100 full days. On average, one geoglyph could be dug in a year. However, according to the oral tradition (collected by PKV) of some Indigenous peoples in the area, the division of labour in local communities was such that some residents were responsible for burning and clearing the forest, while others were

responsible for digging. Applying this information to the 2009 estimate would increase the minimum required excavation time to 200 days, but it would not necessarily change the required community size — only the division of labour would change.

Secondly, it should be noted that maintaining land structures in the Amazonian rainforest requires repetitive, careful cleaning of continuously growing vegetation. It is still a crucial act to prepare and maintain ritual plazas in Amazonia by careful collective cleaning activities. We have robust ethnographic evidence for this in Acre and Amazonas, e.g. diverse people cleaning in rows (field notes by PKV). Rainforest clearings quickly become overgrown if leaves are not cleaned and shoots are not removed at the root. These cleaning operations were generally reported by Europeans who arrived in the 19th century (e.g. Mercier, [1894] 1981), and this practice continues in many places in Amazonia today. For instance, Lathrap (1962) described how ceremonial areas and streets in Peruvian Amazonia were continuously cleaned and kept free of vegetation. Furthermore, our archaeological excavations at over 30 sites in the Aquiry region also prove beyond a doubt that the interior of the geoglyphs was always cleaned after ceremonies. As the places were used temporally, the ceremonial potteries also moved with people. It is often difficult to find diagnostic pieces of pottery or charcoal large enough for dating purposes inside the structures. However, over centuries of use, ash, dust, and tiny pieces of charcoal and pottery normally accumulated on the ground to a depth of between 20 and 40 centimetres, sometimes up to 70 cm. Pottery has also accumulated at the bottom of the ditches. In some cases, more of the cleaned soil drifted into the moats at the end of the geoglyphs' use (field notes by MP and SS). Additionally, it has been found that fire and sandy clay were also used for cleaning in places where food and drink were served, and many dishes were broken (Pärssinen 2021a; Pärssinen *et al*. 2021, and field notes by MP).While the exact organisation of these events remains uncertain, it is evident that these activities, along with the controlled burning of vegetation, were undertaken by the communities themselves. It is important to note that there is no evidence of a centralised state or itinerant construction workers in the Amazon region. If the same workers had designed and dug several geoglyphs, their similarity would certainly attract attention. However, this is not the case. We can therefore assume that the communities themselves did the digging, while the design work required the mathematical skills and expertise of a few individuals.

Finally, according to historical and ethnographic evidence, most people from each community participated in the most important local ceremonies, and important people were also invited from other communities for some specific ceremonies. An independent metric approximation of these people is given on page 8. During the historical period, drinking and dancing activities were extremely popular among the Brazilian Purus and Bolivian Baures (Eder [1772] 1985; Labre 1872), and Eder ([1772] 1985, 85) even argued that the only function of local chiefs, *arama*, was to invite his subjects to drink with "largesse". Nevertheless, drinking was not the only purpose of the gathering. Eder ([1772] 1985, 293–299) also mentions wrestling, archery, running, eating hot corn cobs, and, most importantly, ball games.

In summary, although burning, digging, cleaning, maintaining, organising ceremonies, feasting, and other uses of geometrically patterned earthworks are all different activities, it is quite clear that the same community organised all these activities in each particular ceremonial centre over long periods of time. Thus, two questions arise: how many of these earthworks were constructed per year during the peak periods, and how many of them were in active, simultaneous, yearly use, maintained by their corresponding communities? Archaeological excavations and the chronological layers observed and dated during the last 20 years confirm beyond a doubt that these sites were used for several centuries, sometimes for over a thousand years. Reports of these excavations are filed with the Brazilian Instituto do Patrimônio Histórico e Artístico Nacional, and most of their scientific results can be found in the reference list of this manuscript. A list of all available radiocarbon dates, along with the latest and most accurate calibration corrections as supporting evidence, can be found in the Supplementary Information (Supplementary Tables 1a and 1b). In addition, we used the currently recommended Bayesian approach and modelling to produce a Kernel density estimate of all geoglyph dates (see Supplementary Information, lines 39–74). We also produced a series of maps showing the temporal and geographical development of dated Aquiry sites using OxCal 4.4.4 (see Supplementary Figure 3). Furthermore, we have presented and analysed the overlapping KDE summaries obtained through calibration and Bayesian modelling (Extended Data Figure 5 and lines 251–261), which show that most of the radiocarbon dates obtained from chronological levels associated with human activity inside geoglyphs (e.g. shards, burnt clay, charcoal and burnt macrofossils) are clearly contemporaneous between 100 and 300 AD. These overlapping summaries include the

earthworks built during the period in question, as well as those built between c. 600 BC and 100 AD that were still in active use. In sum, this modelling suggests that up to 10,460 earthworks in total were used at least some moments during the period 100 - 300 AD. This kind of modelling is probably the most common way in which archaeological contemporaneity is indicated in current journals such as *Radiocarbon*, and the other analyses presented before this serve as supporting evidence. Thus, the argument that 'there is absolutely no supporting evidence for how they estimate the number of people who would have simultaneously used or maintained these geoglyphs' is not true.

As said, when estimating the total population of the Aquiry, the most relevant question for us is when most of the earthworks were used over a short period of time. However, this does not mean that modelling techniques such as Kernel density estimation (KDE) are not useful for determining how many structures were constructed in a given period or year, even though these techniques do not take into account earthworks constructed before the calculated period that were still in use at that time.

Contrary to what the reviewer says, we did indeed estimate the number of geoglyphs constructed per year "*based on radiocarbon ages and their chronological model*". Between lines 252 and 255, we state that "*the densest overlap of calibration dates occurs at around 100–300 AD, both chronologically and spatially, corresponding to a 25% probability of the Aquiry earthworks being constructed, according to Kernel density estimates (see Supplementary Figure 2)*". We clearly state that this 'independent' chronological activity model yields a figure of 4,360 earthworks per 200 years, equating to an average of 22 geoglyphs constructed per year between 100 and 300 AD. According to our various estimates, this would require an average community of 300 people, multiplied by 4,360, equating to 1.3 million people. Taking the previously mentioned multi-family versus single-family dwellings and visibility issues into account, the absolute minimum required would be between 370,000 and 800,000 people, but this is extremely unlikely.

Furthermore, we would like to point out that the figure for an average community of 300 people includes the calculation of the average labour force required to construct a geoglyph, which is at least 80 people digging 1 m^3 per day. Thus, if all 60 adult men of each community of 300 people (accepting the nearly universal relation of 1 adult man per family of 5 individuals) and 20 women were needed for each construction for 100

days of the year, then the construction of 4,360 earthworks would have required 261,600 adult men in total. This corresponds (x5) to the same population of 1.3 million people. As we have argued, this is the minimum population size (for constructed 4,360 earthworks) required for the earthwork-building Aquiry society, provided the basic assumptions are correct. Furthermore, if all 80 earthmovers were local men, the average community population would have been near 400 people, and hence the minimum population would have been 1.7 million. Moreover, it is highly unlikely that communities five times smaller — in the case, if the later mound village dwellings used for comparison belonged to single families rather than general multifamily units — would have been able to construct 4,360 monumental earthworks over 200 years.

By using the variety of evidence presented above, particularly based on radiocarbon data, we argue that ca. 25–60% of all known geoglyphs were in simultaneous use over a period of 200 years from 100 to 300 AD. This means that, at any given moment over this period, there were ca. 4,360–10,460 geoglyphs in use. In other words, one must try to envision a human population that would be able to allocate enough labour to construct 4,360 geoglyphs and maintain these, along with thousands of other earthworks constructed before, in a sufficiently adequate condition for festivities, ceremonies, and other activities.

Finally, we are surprised by the opinion that one should look separately at the numbers of people required for building, maintenance, and use of the geoglyphs. Why speculate about these kinds of communal activities? In our view, the only reasonable assumption about the geographical pattern of population distribution of the Aquiry is that it basically consisted of thousands of communities, where each one was associated to a single or multiple geoglyphs that served as centres for festivities and ceremonies. If one accepts that, in most cases, a community with a single ceremonial centre was the operative unit in the study area, then it should be obvious what lines of evidence one can use to estimate the average population size of a community. A community must have been able to use, maintain, and perhaps build a geoglyph, unless it was just maintaining one that was built by the previous occupants of the community.

Accordingly, one should use all possible lines of evidence for estimating the population size that could continue these activities without jeopardising the sustainability of the community. This is what we have done in the manuscript, and we cannot understand

what the utility of a separate analysis of the time and people needed for different activities would be, other than approximating the possible number of workers needed to excavate ditches. Estimates of the number of people associated with each activity do not tell us much about the total population, as it is highly likely that the same adults participated in various communal activities (including opening areas for earthworks by fire, digging ditches and opening roads, cleaning roads and earthworks, constructing communal storage and other buildings, sowing and harvesting crops, preparing drinks and food for ceremonies, and participating in celebrations and ceremonies). In addition, women, children, and elderly people have played a role in many of these activities. For these reasons, we cannot calculate the population more accurately. As we have already noted, our Indigenous colleagues have agreed with our estimation of how much space a gathering of 200–400 people would need for their most important traditional ceremonies (lines 320–326). Thus, further analysis should be left until more information is available.

Referee #5

The issue with civilisation is that it is being used in two different concurrent ways because it is an inherently vague, poorly defined term. If it were replaced by "cultural tradition" throughout the early part of the paper the implicit "quality" and "magnitude" inherent to the term "civilisation" would be absent. And the jump at the end of the paper to civilisation referring to massive, wealth state organised societies with large settlements (eg Greater Angkor) several orders of magnitude larger than the earthen enclosures would then be glaringly apparent. The authors would need to explain why comparability is claimed across several orders of magnitude to societies which are literate, had major state administrations and built very substantial structures.

Here, Referee #5's statement is unfortunately outdated. There are more than two ways the term civilization can be used, and in fact, the way in which the referee defines civilization was rejected in South American archaeological and anthropological discourse more than 70 years ago.

Over the last 150 years, hundreds of different definitions for civilization have been proposed. Because it seems to us that the referee does not know the vast literature on civilizations in the South American context, we must summarize some of our earlier writings. In anthropological and archaeological discussions, the concept has a long

history and has been treated as an evolutionary process. For example, Edward B. Tylor (1871) stated that civilization signified "the most complex of human conditions." Somewhat later, Lewis Henry Morgan ([1877-1878] 1985) presented his more sophisticated evolutionary theory, how societies developed from savagery to barbarism and from barbarism to civilization (that included writing). This was based on Christian J. Thomsen (1848), and John Lubbock (1865) divisions of European pre-history, and a Roman poet Titus Lucretius Carus´s idea (ca. 55 BC: book V) that the development of humankind started from Stone Age to Copper/Bronze Age and developed from Copper/Bronze Age to the Iron Age of the Roman civilization. Thanks to Friedrich Engels (1884), the writings of Morgan became a part of Marxist theory of how societies developed during their journey toward communism.

At the beginning of 20th century, many other evolutionary theories appeared. Among the most influential were the theories of Spengler and Toynbee. Of these, the British historian Arnold Toynbee collected information of more than 20 independent world civilizations and compared their rise and fall in his 12 voluminous mayor work: *A Study of History* (1934-1961). From the Americas, Toynbee accepted four full entities to his list: Andean, Mexican, Yucatec, and Mayan civilizations. He concluded that civilizations rose mainly due to environmental and other challenges that were resolved by creative individuals and minorities forming elite leaders.

Also, it is important to note that North American anthropologist Julian Steward (1955) opposed Morgan´s original ideas. Adopting some ideas of Toynbee, Steward argued that instead of a unilineal and universal track, human cultures had multilinear development possibilities dependent on environmental, technological, and regional factors. Writing, iron technology, or state administration were not considered necessary conditions for civilization. When Steward edited the Handbook of South American Indians (1946-1950/1959), he saw the Andes as the focus of higher civilizations. While volume I of the Handbook, edited by Steward, was dedicated to marginal hunting and gathering tribes, volume II focused on Andean farming civilizations. Volume III dealt with horticultural, hunting and gathering tropical forest tribes, and the fourth volume detailed Circum-Caribbean tribes. The level of civilization is only specifically mentioned in the Andean context, when referring to Chavín, Moche (Mochica), Tiwanaku, Inca, and Columbian Muisca (Chibcha) cultures. Also, volume I is an exception among the marginal tribes: there, a Swiss writer, Alfred Métraux (1946, 209) mentions the

Diaguita culture in Chile and Argentina as a civilization due to the high quality of their geometrically painted polychrome pottery and highly developed social organization.

The Handbook of South American Indians formed a standard model for South American civilizations for a long time, which Indigenous nations themselves and many decolonial researchers have shown to be wrong in many publications. It is crucial to note that at the time when Handbook was edited, Amazonian archaeology was only developing, and Acrean geoglyphs, among others, were unknown. Therefore, it is understandable that Steward believed the tropical forest to be ecologically unsuitable for cultural development toward civilization. Their claim can only be understood if one knows well the current Amazonian research and genealogy of Amazonian archaeology. Amazonian societies were mainly seen as "primitive" egalitarian tribes living in small impermanent hamlets in a hostile environment and without any complex social organizations (Steward & Métraux 1948; Steward & Faron 1959). This idea was maintained until the end of the last century (Meggers 1992). Nevertheless, our understandings of past human development started to change radically around 30 years ago, as researchers started to realize the effects of European diseases on Indigenous populations (e.g. Denevan 1992, 205-234; Dobyns 1966; Roosevelt 1991) and anthropological research was conducted in the Lower and Central Amazon river, in the Xingú, as well as in the Bolivian Mojos and Baures regions (Erickson, 2000, 2006; Heckenberger *et al*. 2003, 2008; Neves & Petersen 2006; Roosevelt *et al*. 1991; Roosevelt 2013). There, highly developed, mainly multicultural civilisations were found that built monumental earthworks, including dwellings and/or ceremonial centres with roads/causeways and canals. These societies also shared religious views and iconographic trends, and they domesticated various edible plants. The earliest examples of Amazonian civilizations are fully comparable to the early civilisations of the Andes, as evidenced, for example, by the widespread Chavín religious cult, whose iconography and construction of monumental ceremonial centres is comparable in size to those of the Aquiry civilization. Chavín is also known to have been significantly influenced by Amazonian cultures. Consequently, many authoritative publications, such as *Science* and *Antiquity*, now use the term civilization to refer to Amazonia as well.

Today, civilization is used to speak about Western or Christian civilization, Chinese civilization, Islamic civilization, ancient Andean and Mesoamerican civilizations, and the like, referring general religious, ideological, and cultural features (churches,

mosques, pyramids, etc.) within greater multicultural geographical areas of complex societies (see also Spinney 2025). We may well add ancient Amazonian civilizations to this list as well, as proposed by Heckenberger et al. (2008), among others. In the case of the Aquiry, the main cultural features were their ceremonial centers, monumental geometrically patterned ditched earthworks (due absence of suitable stone) with wide and narrow roads, and likely some huge wooden buildings that have already disappeared. Finally, it is also important to note that the 19th century meaning of the term civilization is not the one that the Oxford and Cambridge English Dictionaries use today. For example, OED https://www.oed.com/dictionary/civilization_n?tab=meaning_and_use#9266268 gives seven entries for the noun civilization, of which the nearest of our field are the followings:

-The state or condition of being civilized; human cultural, social, and intellectual development when considered to be advanced and progressive in nature. Also extended use.

-With modifying word. The culture, society, and way of life of a particular country, región, epoch, or group. E.g., Islamic civilization, Neolithic civilization, Christian civilization, Egyptian civilization, etc.

According to the Cambridge online-dictionary the concept of civilization means:

(1.) Developed society, in general, and
(2.) human society with its well developed social organizations, or the culture and way of life of a society or country at a particular period in time, or
(3.) a highly developed culture, including its social organization, government, laws, and arts, or the culture of a social group or country at a particular time, in particular (see, https://dictionary.cambridge.org/dictionary/english/civilization)

These definitions are consistent with our understanding of how the term “civilization” should be used in English. However, if we were to revert to the old Eurocentric definition of civilization from the 19th-century Morgan–Engels school, which encompasses the notions of a requisite hierarchical state and Eurasian-style writing systems, we would face significant challenges and likely accusations of neo-colonialism. We do not want to adopt this Eurocentric definition of "civilization," and

we argue that most Great Andean multicultural complex societies, such as Chavín and Tiwanaku, are rightly called civilizations. The earlier Chavín civilization was structured around ceremonial centers such as Chavín de Huantar, the best-known center of Chavín civilization. It was about 7.5 ha of its size during ca. 1000 – 500 BC, and only after this older period, the size of the center duplicated to 15 ha, ca. 500 – 200 BC (Burger 1992, 161). Thus, it is not extremely big. The largest geoglyph in Tumichucua (Bolivia) covers an area of 50 hectares. When combined with other earthworks, the entire archaeological site encompasses an area of 120 hectares (Arnold & Prettol 1988; Saunaluoma 2010; Pärssinen, Kalliola & Ranzi 2024).

We interpret the referee's citation to 'Greater Angkor' as referring to the following passage from lines 377–381: "*However, unlike many other tropical civilizations in the world*[16,48]*, it appears that large-scale water management projects with raised fields and causeways were not a major focus of their subsistence strategies in extensive interfluve environments, although the presence of water appears to be one of the key elements for the earthwork sites and their roads*[3]."

Here, note 16 refers to Prümers et al.'s research in Llanos de Mojos, Bolivia, and note 48 refers to Lucero, Fletcher & Coningham's article "*From 'collapse' to urban diaspora*," in which the authors compare Angkor (c. 750,000 people) in Cambodia, Anuradhapura (c. 250,000 people) in Sri Lanka, and the Maya capital of Tikal (c.60,000 people?) in Guatemala. In these tropical regions, the management of different kinds of waterworks was crucial for the maintenance of administrative and socio-political systems, and after the breakdown the inhabitants largely abandoned the urban centres. Finally, "*former subjects continued farming, but many did so in different areas within smaller community networks and engaged in a different kind of urbanism on the periphery of the former states*" (Lucero *et al.* 2015).

The question is therefore merely a general statement that the Aquiry is different from many other tropical civilizations that had large-scale water management projects, including raised fields and causeways. Although some canals are known to exist in the Aquiry region (e.g. Tumichucua), raised fields and causeways are absent. We have discussed omitting this statement at the time, but it was kept in because Referee #5 had specifically asked us to note the comparison with Mesoamerica. As we could not fit in any new references, we left it in. However, if the referee is bothered by the fact that the

article compares Greater Angkor, which had a population of nearly one million, to Tikal, which was more than ten times smaller, then the entire reference can be omitted.

Urban is used because it is term which possesses cachet and has rapidly gained a vague and plastic identity as an academic category. The authors have many companions worldwide in this practice of overextending the term urban.

Since there is no such thing as standard urban density, it is sometimes difficult to assess when the term is being used too broadly. However, this issue has been addressed in previous articles (e.g. Pärssinen, 2021b; Virtanen, 2023), which show a different idea of Amazonian urbanity, and we refer to these articles and other supporting articles in our manuscript. In particular, we highlight how the latest research addresses this issue in relation to the Amazonia. Unfortunately, the reviewer appears to be unfamiliar with this discussion and has not read the referenced sources from the manuscript carefully enough.

For instance, Pärssinen (2021b) compared the Aquiry civilization with the Greek city-states, referencing the work of researchers at the University of Copenhagen in Denmark who catalogued 1,035 Greek poleis stretching from Spain and France to Libya and Asia Minor via current Greece. The Greek civilization lasted about 1250 years, from 750 BC to AD 500. Hansen (2006) estimates that some 500 poleis are still missing from the Danish inventory, but in general some 850 – 1000 city-states made up Greek civilization during the Archaic and Classical periods (c. 700 – 323 BC). Most Greek city-states were fortified by walls, and they had a plaza for markets and public assemblies. The superficies of smallest city-states had less than 8 ha and their entire defensible territory reached up to c. 10 km² while the most typical poleis had c. 27 ha behind walls and their defense circuit varied between 25 and 100 km². Only some 10% of city-states, including Athens, consisted of around 182 ha or more.

Today, we know of hundreds of the Aquiry sites that contain two, three, or more geoglyphs and cover more than 20 ha. It is also important to consider that it was calculated that the public space of medium size Greek city was c. one quarter of the fortified area, and the rest of the walled city was dedicated for housing and private use. Thus, it was estimated that some 6 – 7 ha was dedicated for ceremonial and social purposes in a typical Greek city. If we now compare this to the ceremonial centers of the Aquiry, the difference is not very big. The biggest known geoglyph has 50 ha in

Tumichucua that covers, as an archaeological site, 120 ha in total, and it is likely that more than a thousand of the Aquiry individual geoglyphs reach 6 – 7 ha. These facts, as well as many other common aspects, indicate that we may speak about civilization and some kind of urbanism, such as low density or forest urbanism, in the connection of the Aquiry.

Furthermore, we have keenly followed the discussion of different kinds of urbanism, especially in the Amazonian context and even in other areas. Among others, we have noted that Lucero, Fletcher & Coningham (2015) seemingly refer to Postclassic Maya culture as smaller communities "*engaged in a different kind of urbanism on the periphery of the former states*." In our area, we do not claim that villages that appeared after the collapse of the Aquiry would have shown any further marks of urbanism. In every case in our manuscript, we refer to articles by leading researchers that deal with pre-colonial Amazonian urbanity. The list of citations is as follows:

1. Roosevelt, A.C. The Amazon and the Anthropocene: 13,000 years of human influence in a tropical rainforest. *Anthropocene* **4**, 69-87 (2013).
2. Heckenberger, M.J., Russell, J.C., Fausto, C., Toney, J.R., Schmidt, M.J., Pereira, E., Franchetto, B. & Kuikuro, A. Pre-Columbian Urbanism, Anthropogenic Landscapes, and the Future of the Amazon. *Science* **321**, 1214-1217 (2008).
3. Neves, E.G. Was Agriculture a Key Productive Activity in Pre-Colonial Amazonia? The Stable Productive Basis for Social Equality in the Central Amazon, in *Human-Environment Interactions, Current and Future Directions* (eds. Brondízio, E.S. & Moran, E.F.) 371-388 (Dordrecht: Springer, 2013).
4. Lombardo, U., Hilbert, L., Bentley, M.K., Bronk Ramsey, C., Dudgeon, K., Gaitan-Roca, A., Iriarte, J., Mejía Ramón, A.G., Quezada, S., Raczka, M., Watling, J.G., Neves, E.G. & Mayle, F. Maize monoculture supported pre-Columbian urbanism in southwestern Amazonia. *Nature*, online 29.1.2025. https://doi.org/10.1038/s41586-024-08473-y3
5. Prümers, H., Betancourt, C.J., Iriarte, J., Robinson, M. & Schaich, M. Lidar reveals pre-Hispanic low-density urbanism in the Bolivian Amazon. *Nature* **606**: 325-358 (2022).
6. Rostain, S., Dorison, A., de Saulieu, G., Prümers, H., Le Pennec, J-L., Mejía Mejía, F., Freire, A-M., Pagán-Jiménez, J. R. & Descola, P. Two thousand years of garden urbanism in the Upper Amazon, *Science* **383**, 183-189 (2024).
7. Virtanen P.K. Urbanity in Ancient and Present-Day Southwestern Amazonia. Human-Environment Collectives, Cycles of Generosity, and Their Ruptures, in *Urban Imaginaries in Native Amazonia: Tales of Alterity, Power, and Defiance*

(eds. Santos-Granero, F. & Fabiano, E.) 205-232 (Tucson: The University of Arizona Press, 2023).

After comparing these (and many other) cases, we are convinced it is correct to speak about different kinds of tropical, low-density urbanism in all these contexts, including the Aquiry. Unfortunately, due the fact that some of us have analysed this topic already elsewhere, it does not make sense to give a new comprehensive analysis in this article. On the administrative aspects of geoglyph building society, please see also Virtanen & Saunaluoma (2015, 2017) and Pärssinen & Ranzi (2020). Finally, we would like to cite forthcoming comments of Kirrily White: ”*dense populations? Again it's problematic – but it is frequently used in the literature without clarification of what dense means and the discussion in Amazon is on types of urbanism, so it isn't an outlier in terms of literature from other regions*.”

And dense is readily used by the authors when it is not an appropriate descriptor. A region of 183,000 sq km with an average density between 0.07 persons per ha and 0.2 persons per ha, at maximum, is not densely occupied. Extensive dense rural provinces would have overall densities in the 2-5 p/ha range.

This comment is interesting. While Referee #3 has argued that it is impossible for the area to have been so dense that the population reached up to 3 million people, Referee #5 states that 3 million is not dense at all. If we understand correctly, the population of the Aquiry should have been between 36.5 and 91.5 million people (two to five people per hectare) before it could be considered dense. These amounts correspond to the general population estimates of all pre-colonial double continents of the Americas, and all of them should have lived in the Aquiry before we could call the area an "*extensive dense rural province*."

For clarity, we consider an area of 85,000 km² to be relatively dense, not 183,000 km². Secondly, the concept of density is relative, with fuzzy logic, and depends on what we are comparing it to. Referee #5 does not mention where he obtained his universal density values, but he may have his own Interaction-Communication model in mind (Fletcher, White & Penny, 2024), which was developed to study the internal residential density of settlements. We do not know. Nevertheless, we are not talking about the

internal density of settlements when we talk about the Aquiry population density, because the settlements themselves have yet to be discovered.

In every case, we prefer to use these concepts relationally according to the context in which they are employed, as most researchers currently do, until researchers have debated and agreed on the use of our referee's universal values of low, medium, and high population density. Meanwhile, we will ensure that this relationality is clearly expressed throughout our manuscript (e.g. relatively densely populated region).

Comparison to other societies produces further issues - the Neolithic enclosures and megalithic monuments of Europe are considered to have had public, ceremonial and political functions in relation to visits by mobile regional populations. The monuments display mathematical and geometric competence and involved cosmological understandings. They required organised labour. Are they, on the analogy proposed by the authors therefore urban? -a term which specialists who work on Stonehenge reject. And has repeatedly been pointed out large structures do not require large amounts of labour. The population estimates made on the basis of maintenance costs are problematic because the evidence is not provided that earthen embankments stabilised by vegetation need substantial maintenace.

Firstly, as mentioned earlier, it should be noted that maintaining structures in the Amazonian rainforest requires careful repetitive cleaning. It is still a crucial act to prepare and maintain ritual plazas in Amazonia by careful collective cleaning activities. Rainforest clearings quickly become overgrown if leaves are not cleaned and shoots are not removed at the root. We do not know about Stonehenge. However, as we have described earlier: "*These cleaning operations were generally reported by Europeans who arrived in the 19th century (e.g. Mercier [1894]1981), and this practice continues today in many places in Amazonia. For example, Lathrap (1962) described how ceremonial areas and streets in Peruvian Amazonia were continuously cleaned and kept free of vegetation. Furthermore, our archaeological excavations at over 30 sites in the Aquiry region prove beyond doubt that the interior of the geoglyphs has always been cleared before and after ceremonies. As the places were used temporally, the ceremonial potteries also moved with people. It is often difficult to find diagnostic*

pieces of pottery or charcoal large enough to date inside the structures. However, over centuries of use, ash, dust, and tiny pieces of charcoal and pottery have accumulated on the ground. Pottery has also accumulated at the bottom of the ditches, and in some cases, more of the cleaned soil has drifted into the moats at the end of the geoglyphs' use (field notes by MP and SS). Additionally, it has been found that fire and sandy clay were also used for cleaning in places where food and drink were served and many dishes were broken (Pärssinen 2021a; Pärssinen et al. 2021; and field notes by MP)."

To our knowledge, the size of the original circular ditch of the Stonehenge ceremonial centre is approximately 0.785 hectares. In comparison, the Tumichucua circular ditch covers an area of around 50 hectares and has a total area of around 120 hectares, making Stonehenge a relatively small site. The average size of the Aquiry sites is 3 hectares, and there are also hundreds of sites with earthwork concentrations covering an area of 20–40 hectares. However, it is unclear whether the Stonehenge area could potentially comprise thousands, or even up to 20,000, similar sites, or sites that are three times larger on average, within an area of approximately 85,000 km². This is a central question. If this can be proven, then we could consider whether we could also talk about Neolithic forest urbanism in Europe using an analogy.

A specific set of data which is required in this paper is the size distribution of the earthen enclosures. Does it have a characteristic form of many small features, some medium sized features circa 10-14 ha perhaps, a a few rare larger ones at circa 50 ha? Readers need to know, because reference to average sizes is problematic when a massive skew to a few large cases is occurring.

This is a bit odd, given that the sizes of all dated earthworks are explained in the text, and the sizes of the 406 earthworks discovered by our LiDAR project are listed in the Supplementary Table 2. As this article deals with the results of the LiDAR project, our focus is on this material. Looking at this list, it can be seen that the size of earthworks "tends to vary between 0.35 and 14 hectares" (lines 79–80). This means, as everyone can easily see, that there are not any significant jumps between the sizes that gives an average 3 ha. We also note that some known earthworks are large, covering up to 50 hectares, and clusters of various earthworks in the same site exist. As these issues have already been dealt with in previous studies, we have only provided references to some of those articles.

Furthermore, the coordinates of 1,279 earthworks, which were previously known mainly through satellite imagery, have been published as a supplement to *Acta Amazonica* (54/4, 2024):

https://doi.org/10.23729/31c411bf-5d94-4cfc-be06-006c16a5c309 (Dataset>You can open the new version via this link> Data> Files> Ancient earthworks)

We have used this data to calculate the average size of the Aquiry type earthworks in the Blocks 1, 2, and 5. Nevertheless, this data and its copyrights belong to another project coordinated by the University of Turku. The referee may request unpublished information on the size of the earthworks listed in the supplementary material of *Acta Amazonica* via the following email address: riskall@utu.fi.

The occurrence of the earthen enclosures over time also needs to be reconsidered. Even with a claimed peak period of only 200 years (note - in a 1500 year tradition) in the 85,000 sq km denser area 22,400 enclosures generates 4 per sq km ie 1 on average in a 25 ha area ie "half" an enclosure in a century at peak activity. This surely is not substantial or rapid construction. Is the total population actually an aggregate of all the people who have lived in a region over a span of time rather than a maximum population present at any one time.

What can be considered a "substantial amount of construction" is also a fuzzy and relative expression. If one expected that the entire population of North and South America (or between 36.5 and 91.5 million) had to have lived in this particular area before it could be considered substantial or dense, then constructions made by 1.3 million would be a low amount. However, we can return here to one of our main points that the Amazonas covers 6.5 million km², and it is predicted that there are between 10,272 and 23,648 undiscovered pre-Columbian structures in the area "*with 95% probability giving an average of 16,187 sites*" (Peripato et al. 2023). From this point, 22,400 enclosures in an area of 85,000 sq km could indeed be considered relatively substantial. This is because the cited area represents only 1.3% of the entire Amazonia (which is 76.5 times bigger than our 85,000 km²).

We used the OxCal 4.4.4 KDE Model function to estimate the relative intensity of human activity. This identified a local maximum of the Aquiry period in the calendar-year probability distribution between 250 BC and 400 AD (see Supplementary Fig. 2). This suggests that the construction of dated structures belonging to the Aquiry cultural

phase peaked during this period. Judging by the ratio of the KDE distribution sums during the peak and full periods, as well as the associated uncertainties in the KDE plot, the peak corresponds to 61% ± 20% of the full KDE distribution. Since the dates were derived directly from the structures, it can be estimated that approximately 60% of them were constructed during the peak period.

As explained in the manuscript, Supplementary Figure 2 illustrates the relative intensity of the entire Aquiry phase. However, it does not fully account for the fact that many earlier earthworks were still in use during the construction peak around 200 AD (see Extended Data Figure 5 and Supplementary Figure 3). The KDE model function estimates that 25% of construction activities date to the period 100–300 AD. This equates to 22 per year. Therefore, it is also important to examine how many earthworks were actually in use, and how many were cleaned and maintained, during this period according to 14C dates. Examining the overlap of the radiocarbon dates of all sites using KDE-modelled summaries (see Extended Data Figure 5) reveals that, around 200 ± 100 AD, approximately 60% of the dated centres were in use simultaneously, with a considerably high probability. All the radiocarbon dates relating to ceremonial activities in the Amazonas region (n = 4) and Bolivia (n = 3) fall within this period, as do around 60% of Acrean geoglyphs. Taking into account all uncertainties, we estimate that there is a 25–60% probability that all earthworks were in use simultaneously around 100–300 AD, with a strong preference towards the upper limit. It is estimated that during this period, approximately 4,360–10,460 Aquiry earthworks were in use within an area of 183,000 km², and around 4,043–9,702 earthworks were in use within an area of 85,000 km². This equates to 0.048–0.114 earthworks per sq km, which, when multiplied by an average population density of 300, equates to 14.4–34.2 people per sq km in the 85,000 sq km core area. This aligns with the concept of low-density urbanism presented in the Amazonian context. For example, after we submitted this manuscript for *Nature*, Hirst and others published an article where they wrote: "Our results indicate that the Casarabe Culture [in the Bolivian Mojos] likely grew to a maximum population of between 10,000 and 100,000 people within a 5020 km² quadrant of their former territory, representing a density of between 2 and 20 people km.$^{2-}$ These values…support previous interpretations that this culture practiced a form of low-density urbanism" (Hirst *et al.* 2025, 1/29).

As with most estimations of this kind, researchers specify the time period during which the population maximum is achieved at sites that were used during various centuries. For instance, De Souza et al. (2018) assert that the maximum extent of the earthworks was reached at some point between 1250 and 1500 AD in the Southern Rim. Similarly, Dyer (2000, 508) writes that, in later medieval Britain, there were likely 861 small towns, and that, in any one year between 1270 and 1540 AD, around 700 of these were functioning as urban communities, and so on. We would argue that the maximum was probably reached in the Aquiry area at some point between 100 and 300 AD.

Note that a Britannica reference - 39 - is not sufficient nor is the use of Curet 1998 as a source for population estimates that is ove 25 years old.

We cited *Encyclopaedia Britannica* because it summarised the size and number of German towns in the 15th century well, which we know to be true after reading dozens of articles on the topic. However, if *Nature* does not allow articles on population history published in the *Encyclopaedia Britannica* to be cited ("*German society, economy and culture in the 14th and 15th centuries: Urban Life*"), we can adjust our general comparison to focus on later medieval British towns, as discussed in the *Cambridge Urban History of Britain* (2000). This can be done with two references. However, we do not understand why we cannot mention Curet's (1998) article published in *Antiquity*, in which a linear regression model was created specifically for estimating prehistoric populations in Lowland South America. This is the same model used by De Souza et al. in their *Nature Communications* article in 2018, which is discussed in our manuscript (lines 43–45, 235–241). Furthermore, we agree with Curet that direct comparison of the spatial size of settlements and ceremonial centres is not recommended due to differences in their functions (lines 292–293).

Kirrily White has also added some additional specific comments:

p. 25 – are they suggesting that state hierarchy was required to construct the ceremonial etc. earthworks in Acre?

Page 25 ? We are not suggesting that in any place of the manuscript. On the contrary, in various earlier articles we have demonstrated that it is unlikely that any hierarchical

state would have been involved when Acrean earthworks were constructed. As ethnographical and ethnohistorical examples from Ceylon, Philippines, the Peruvian coast, or Bolivian Baures, for example, demonstrate that impressive earthworks can be built and maintained without demanding state organization (e.g. Pärssinen 2021b).

p. 26 – addressing of mobility seems adequate, there is insufficient information available to date appearance of domestic flora in the area, or to understand the dating and structure of mounds which have not been previously identified as later mound villages.

???

p. 27 – they have provided more information on the radiocarbon dates, locations, and areas – see file 535106_0_supp_4929181_t4ll10, (supplementary data) lines 45-174. Dated sites are limited but are distributed over a largish area of the Acre earthworks and a few sites in Bolivia and Amazonas. There is tabulated information in the supplementary data.

Yes

p. 28 – they have addressed the density of occurrence of geoglyphs in the dating ranges. Most of the dates are for Acre. There is more information about this in the supplementary material. There is a series of maps in the Supplementary material on p. 4 which shows distribution of dated sites by blocks of time. There are also some sites with multiple dates showing phases of use. See also lines 117-174 on pp. 6-7 in the supplementary material. I think that without identification of occupation areas, they have to go on the size of the earthworks or maybe conjoined earthworks, so that the sizes are all small.

OK. However, it should be recognised that 'small' is also a fuzzy and relative concept. In the Amazonian context, Acrean earthworks, as public ceremonial spaces of their communities, have comparable sizes to many other cultural areas considered to be

examples of low-density urbanism (e.g. Heckenberger *et al*. 2008; Prümers *et al*. 2022; Rostain *et al*. 2024; Lombardo *et al*. 2025).

As in this article, the size and density of Indigenous peoples' ceremonial centres are used to estimate population size. Similarly, the size and density of churches in medieval Christian cities have been used for this purpose in Europe. Sometimes it has been the only possibility, and other times those have been compared to other available historical or archaeological information (e.g. Gauthier *et.al.* 1986–2014; Buringh 2021). Furthermore, while the question of different forms of urbanism and settlement size in different areas and periods is interesting, it is not the main focus of this article. Here, we examine the size of the Aquiry area and the number of ceremonial centres, attempting to make a preliminary population estimate based on currently available information.

p. 29 – see note below for supplementary data on population size, they don't have much to go on, and some discussion of population seems to be needed.

What is the note the reviewer is referring to? Considerable discussion on population is provided especially in lines 242-326 of the main text, and in many parts of the Supplementary Information, especially in the lines 175-206.

Supplementary Data

pp. 2-3, lines 47-74. I am concerned that they are not interpreting the KDE data appropriately by using percentages, would like to discuss it with you, but might need to consult a mathematician.

A mathematician and specialist of a radiocarbon chronology is a member of our research group -- his laboratory has realised the KDE modelling, and he has also given percentages. Percentages were also confirmed by an archaeologist and some other specialists before being added to the manuscript.

pp. 7-8, lines 175-206 discussion of population size. Importantly, they are talking about very small villages and I am aware from Roosevelt (1991. Moundbuilders of the Amazon: Geophysical Archaeology on Marajo Island, Brazil. Academic Press, San

Diego, p.342.) that multifamily longhouses could have 35-60 occupants. In her thesis (pp.171-72), Denise Schaan uses this estimate to propose 12-60 people per building for Camutins mound complex on Marajo Island – the range accounts for the different sizes of houses and mounds.

As one can see, we have used a number situated near the lower limit (25) to avoid accusations of exaggeration.

The way the population estimates are presented in this paper for the geometric earthworks does not seem to be outside the norm for literature in the Amazon regions even though there is not yet occupation evidence, besides perhaps size of earthworks, to base them on and all they can do is use what researchers have said about other regions.

OK.

pp. 8-14 is discussion of lidar methodology. I can't really assess this, but explanations and data appear to be thorough.

OK.

pp. 20-28 is a list of site sizes measured from the satellite and lidar imagery.
OK.

Revised.

p. 2, lines 40-46 – dense populations? Again it's problematic – but it is frequently used in the literature without clarification of what dense means and the discussion in Amazon is on types of urbanism, so it isn't an outlier in terms of literature from other regions.

OK. This is an important point that we have emphasised. We will ensure that this relationality is clearly expressed throughout our manuscript, for example by using the term 'relatively densely populated region'.

Lines 51-52 – again the issue of justification of population estimates. Does the provision of the areas of earthworks help? They have no occupation data and are using comparison with other regions, the size of mounds used at a later time period and ethnographic analogy – is it sufficient?

Ethnographic analogy has been among the most used method in Amazonian population estimations, as well as the size and amount of the habitational mounds. As said, in Europe the size and amounts of cathedrals, churches and basilicas have also been used in population estimations as a line of research. These are our current possibilities, and we are convinced these allow us to give best possible estimation at the moment.

p.4, line 97 – Comparison with Inka, it is comparing apples and oranges.

What is wrong with referring to similar administrative principles in the Inca state? It seems that the referee knows little about the Inca State and has not read the text to which we are referring, which she should have done. We also advise reading comparative books like *Rethinking the Andes–Amazonia Divide. A cross-disciplinary exploration* (eds. Pearse, A.J., Beresford-Jones, D.G. & Heggarty, P., 2020) and *Interpretando Huellas. Arqueología, Etnohistoria y Etnografía de los Andes y sus Tierras Bajas* (ed. Muñoz, M.A., 2018). Firstly, comparisons between Andean and Amazonian cultures have revealed close ties spanning over 5,000 years. Secondly, the Incas occupied part of the former Aquiry territory. This area was part of Tawantinsuyu, in a quarter known as Antisuyu. It is a well-known fact that the Incas did not introduce radical changes to the local administrative principles in conquered territories. The nearest Inca fortification, Las Piedras (Pärssinen, 2015), is located less than 20 kilometres from the Aquiry site of Tumichucua, and some of us have participated in various international conferences where comparisons have been made between Andean and Amazonian cultural traditions and practices.

As our referee does not appear to have read the texts to which we are referring, we would just like to point out that when discussing different types of South American urbanism, it is important to remember that many provincial capitals of the Inca state were also used for feasting and religious ceremonies. Inca provincial capitals, such as Huánaco, Pumpu, Jauja and Paria, were centres where Inca governors, representing the

ruling Inca, invited local people — who lived in small villages and periodically performed work for the Inca state and church — to demonstrate the generosity of the ruling Inca and redistribute gifts stored in warehouses (Morris 1982, 162–166; Pärssinen 1992, 275). The only permanent inhabitants of these urban-like centres, which covered an area of around 100–200 hectares (Pärssinen *et al*. 2010), were a few hundred women and their guards who prepared food and beverages for the invited guests. When the Inca Empire collapsed in the 16th century, these centres were abandoned.

p.5 lines 110-11 – it's not wrong to say that researchers do use these criteria, just wrong that they do use it – all kinds of hunter gatherers built earthworks and practiced arts etc…

Of course, all human communities practised some form of art, and small mobile groups also built significant monuments. Here, we understand that the referee is criticising earlier researchers, such as V. Gordon Childe, who are discussed in lines 110–11. However, does the referee argue that all kind of hunter gatherers built thousands of geometrically planned monumental earthworks in forested areas with five meter deep, over ten meter wide, and up to 2.5 km long ditches together with hundreds of kilometres of wide roads? We would like to know where all kinds of hunter gatherers may have built thousands of similar monumental earthworks and roads.

Most of the paper is description of the data and discussion of different scenarios of interpreting population sizes. It might be helped by a discussion of population mobility, but they are not claiming large cities, just piling on the discussion of urbanism elsewhere in the Amazon literature. I am confused about whether or not they are trying to claim the Aquiry are a state as well as a civilization…

As said earlier, we are not claiming that the Aquiry was a state, but we agree with Heckenberger et al. (2008) and many others that in this Amazonian context we may speak about ancient civilization.

Cited references:

Arnold, D. & Prettol, K. Aboriginal earthworks near the mouth of the Beni, Bolivia, *J. Field Archaeol.* **15**, 457-465 (1988).

Burger, R. L. *Chavin and the Origins of Andean Civilization.* (London: Thames and Hudson, 1992).

Buringh, E. The Population of European Cities from 700 to 2000. *Res. Data J. Humanit. Soc. Sci*. **6**, 1-18 (2021).

Curet, L.A. New formulae for estimating prehistoric populations for lowland South America and the Caribbean. *Antiquity* **72**, 359-375 (1998).

De Souza, J.G. *et al*. Pre-Columbian earth-builders settled along the entire southern rim of the Amazon. *Nature Communications* **9**, 1125 (2018).

Denevan, W. M. The Pristine Myth: The Landscape of the Americas in 1492. *Annals of the Association of American Geographers* **82**, 369-385, 1992.

Dobyns, H. F. Estimating Aboriginal American Population: An Appraisal of Techniques with a New Hemispheric Estimate. *Current Anthropology* **7**, 395-416, 1966.

Dyer, C. Small towns 1270-1540, in The Cambridge Urban History of Britain (ed. Palliser, D.M.) 505-538 (Cambridge: Cambridge University Press, 2000)

Eder, F. J. *Breve descripción de las reducciones de Mojos ca. 1772* (ed. Barnadas, J.M.). (Cochabamba: Historia Boliviana,1985).

Engels, F. *Der Ursprung der Familie, des Privateigenthums und des Staates, im Anschluss an Lewis H. Morgan´s Forschungen*. (Hottingen-Zürich: Verlag der Schweizerischen Volksbuchhandlung , 1884).

Erickson, C. L. Lomas de ocupación en los Llanos de Moxos, in *Arqueología de las tierras bajas*, (eds. Durán Coirolo, A. & Bracco Boksar, R.) 207-226 (Montevideo, Uruguay: Comisión Nacional de Arqueología, 2000).

Erickson, C. L. The Domesticated Landscape of the Bolivian Amazon, in *Time and Complexity in Historical Ecology*, (ed. Balée, W. & Erickson, C.L.) 235-278 (New York: Columbia University Press, 2006).

Fletcher, R., White, K. & Penny, D. Risk and low-density dispersed urbanism. *Front. Hum. Dyn.* **6**:1347157 (2024).

Gauthier, N., Picard, J.C., Duval, N. *et al.* (eds) *Topographie Chrétienne des cités de la Gaule, des origines au milieu du VIIIe siècle*. 16 vols. (Paris: Impr. de Boccard, 1986-2014).

Hansen, M. H. *Polis: An Introduction to the Ancient Greek City-State*. (Oxford: Oxford University Press, 2006).

Heckenberger, M. J. *et al.* Amazonia 1492: Pristine Forest or Cultural Parkland? *Science* **301**: 1710-1713 (2003).

Heckenberger, M.J. *et al*. Pre-Columbian Urbanism, Anthropogenic Landscapes, and the Future of the Amazon. *Science* **321**, 1214-1217 (2008).

Hirst J., Singarayer J., Lombardo U. & Mayle F. Constraining the population size estimates of the pre-Columbian Casarabe Culture of Amazonian Bolivia. *PLoS One* **20**(5): e0325104 (2025). https://doi.org/10.1371/journal.pone.0325104

Iriarte, J., de Souza, J.G., Robinson, M.E., Barbosa, A.D. & da Silva, F. Refining the Chronology and Occupation Dynamics of the Mound Villages of South-eastern Acre, Brazil. *Amazônica, Revista de Antropologia* **13**, 153-176 (2021).

Labre, A. R. P., *Rio Purús: Noticia*. (Maranhão: Imp. M. F. V. Pires, 1872).

Lathrap, D.W., *Yarinacocha: Stratigraphic Excavations in the Peruvian Montana*. Doctoral dissertation, Harvard Graduate School of Arts and Sciences, 1962.

Lombardo, U. *et.al.* Maize monoculture supported pre-Columbian urbanism in southwestern Amazonia. *Nature*, online 29.1.2025. https://doi.org/10.1038/s41586-024-08473-y3

Lubbock, J. 1865. *Pre-Historic Times as Illustrated by Ancient Remains and the Manners and Customs of Modern Savages.* (London: William & Norgate, 1865).

Lucero, L.J., Fletcher, R. & Coningham, R. From ‘collapse’ to urban diaspora: the transformation of low-density, dispersed agrarian urbanism. *Antiquity* **89**, 1139–1154 (2015).

Lucretius Carus T.. *Titus Lucretius Carus, his six books of Epicurean philosophy*, (trad. T. Creech). Fourth edition. (London: T. Braddyll, [ca. 55 a.C.] 1699).

Meggers, B. J. Prehistoric Population Density in the Amazon Basin, in *Disease and Demography in the Americas* (eds. Verano, J.W & Ubelaker, D.H.) 197-205 (Washington D. C.: Smithsonian Institution Press, 1992).

Mercier, V. Diario de una Expedición del Madre de Dios al Acre. La Paz: Imprenta La Tribuna 1894. *Colección de folletos bolivianos de Hoy* **3**, 1-16, Facsimilar (La Paz, Octubre 1981).

Métraux, A. Ethnography of the Chaco, in *Handbook of South American Indians*, Vol. I, (ed. Steward, J.) 197-370 (Washington D.C.: Smithsonian Institution, 1946).

Morgan, L. H. *Ancient Society*. Second edition (ed. Montagu, A.). Classics of Anthropology (Tucson: University of Arizona Press, [1877-1878] 1985).

Morris, C. The infrastructure of Inka control in the Peruvian central highlands, in *The Inca and Aztec States, 1400–1800: Anthropology and History* (eds. Collier, G.A., Rosaldo, R.I. & Wirth, J. D.) 153-171 (New York: Academic Press, 1982).

Neves, E. G. & Petersen, J.B., Political Economy and Pre-Columbian Landscape, Transformations in Central Amazonia, in *Time and Complexity in Historical Ecology*, (eds. Balée, W. & Erickson, C.L.) 279-309 (New York: Columbia University Press, 2006).

Pärssinen, M. *Tawantinsuyu. The Inca State and Its Political Organization*. (Helsinki: Societas Historica Finlandiae, 1992).

Pärssinen, M. Collasuyu of the Inka State, in *The Inka Empire: A multidisciplinary Approach* (ed. Shimada, I) 265-285 (Austin: University of Texas Press, 2015).

Pärssinen, M. Tequinho Geoglyph Site and Early Polychrome Horizon 300 BC – AD 300/500 in the Brazilian State of Acre, *Amazônica: Revista de Antropologia* **13**, 177-220 (2021a).

Pärssinen, M. Aquiry: um breve retrato de uma civilização amazónica, in *Amazônia: Os Geoglifos e a Civilização Aquiry* (eds. Ranzi, A. & Pärssinen, M.) 19-64 (Florianópolis, Helsinki, Madrid: Officio & Fundação Ibero-Americana da Finlândia/Instituto Ibero-Americano da Finlândia, 2021b).

Pärssinen, M & Ranzi, A. Mobilidade cerimonial e a emergência do poder político com as primeiras estradas conhecidas do oeste amazônico (2000 A.P.), in *(I)Mobilidades na Pré-história: Pessoas, recursos, objetos, sítios e territórios* (eds. Vilaça, R. & R. Simas de Aguiar, R.) 307-349 (Coimbra: Coimbra University Press, 2020).

Pärssinen, M., Kalliola, R. & Ranzi, A. Zanjas circundantes en el extremo norte de Bolivia (Pando y Beni) y sus relaciones con los llamados geoglifos de Acre. *Arqueoantropológicas* **9**, 15-49 (2024).

Pärssinen, M., Kesseli, R.U. & Faldín, J. Paria, the southern Inka capital rediscovered. *Chungara: Revista de Antropología Chilena*, **42**, 235-246 (2010).

Pärssinen, M., Schaan, D. & Ranzi, A. Pre-Columbian geometric earthworks in the upper Purús: a complex society in western Amazonia. *Antiquity* **83**, 1084-1095 (2009).

Pärssinen, M., Ferreira, E., Virtanen, P.K. & Ranzi, A. Domestication in motion: Macrofossils of pre-colonial Brazilian nuts, palms and other Amazonian planted tree species found in the Upper Purus. *Environ. Archaeol.* **26**, 309-322 (2021).

Peripato, V. *et al*. More than 10,000 pre-Columbian earthworks are still hidden throughout Amazonia. *Science* **382**, 103-109 (2023).

Prümers, H., Betancourt, C.J., Iriarte, J., Robinson, M. & Schaich, M. Lidar reveals pre-Hispanic low-density urbanism in the Bolivian Amazon. *Nature* **606**: 325-358 (2022).

Roosevelt, A. C. *Moundbuilders of the Amazon: Geophysical Archaeology on the Marajo Island, Brazil*. (San Diego: Academic Press, 1991).

Roosevelt, A. C. The Amazon and the Anthropocene: 13,000 years of human influence in a tropical rainforest. *Anthropocene* **4**: 69-87 (2013).

Roosevelt, A. C., Housley, R.A., Imazio da Silveira, M, Maranca, R. & Johnson, S. Eighth Millennium Pottery from a Prehistoric Shell Midden in the Brazilian Amazon. *Science* **254**, 1621-1624 (1991).

Rostain, S. *et al.* Two thousand years of garden urbanism in the Upper Amazon, *Science* **383**, 183-189 (2024).

Saunaluoma, S. Pre-Columbian Earthworks in the Riberalta Region of the Bolivian Amazon. *Amazônica, Revista de Antropologia* **2**, 86-111 (2010).

Spengler, O. *The Decline of the West.* (New York & Oxford: Oxford University Press, [1918-1922] 1991).

Spinney, L. How warfare drove the rise of civilizations. *Nature* **645**, 845-847 (2025).

Steward, J. H. *Theory of Culture Change: The Methodology of Multilinear Evolution.* (Urbana: University of Illinois Press, 1955).

Steward, J. H. & Faron, L. C. *Native Peoples of South America*. (New York: MacGraw-Hill Book Company Inc, 1959).

Steward, J. H. & Métraux, A. Tribes of the Peruvian and Ecuadorian Montaña, in *Handbook of South American Indians*, Vol. 3, (ed. Steward, J.H.) 535-656 (Washington D. C.: Smithsonian Institution, 1948).

Thomsen, C. J. *Guide to Northern Archæology by the Royal Society of Northern Antiquaries of Copenhagen*, (ed. Earl of Ellesmere), (London: James Bain, Haymarket, 1848).

Toynbee, A. *A Study of History*, Vols. I-XII. (Oxford: Oxford University Press, 1934-1961).

Tylor, E. B. *Primitive Culture: Researches into the Development of Mythology, Philosophy, Religion, Art, and Custom*, Vols. I-II (London: John Murray, 1871).

Virtanen P.K. Urbanity in Ancient and Present-Day Southwestern Amazonia. Human-Environment Collectives, Cycles of Generosity, and Their Ruptures, in *Urban Imaginaries in Native Amazonia: Tales of Alterity, Power, and Defiance* (eds. Santos-Granero, F. & Fabiano, E.) 205-232 (Tucson: The University of Arizona Press, 2023).

Virtanen, P. K. & Saunaluoma, S. Variable Models for Social Organization of Monumental Earthworks in Upper Purus, Southwestern Amazonia: Archaeological and Ethnographic Perspectives. *Tipiti: Journal of the Society for the Anthropology of Lowland South America* , **13**, 23-43 (2015).

Virtanen, P.K. & Saunaluoma, S. Visualization and movement as configurations of human–nonhuman engagements: precolonial geometric earthwork landscapes of the Upper Purus, Brazil. *Am. Anthropol.* **119**, 614-630 (2017).

Point-by-point discussion, 19.1.2026 (for manuscript 2025-04-08530B)

Code: 2025-04-08530C

We would like to thank our referees for taking the time to review our manuscript **"Over 20,000 pre-colonial earthworks in the Southwest Amazonia."** Below is our point-by-point response to the reviewers' new comments and concerns. The referees' comments are in red and our answers are in black.

Referee #1

The Bayesian modelling presented in this revised manuscript is a useful approach to summarizing the radiocarbon-based estimate of the beginning and end of the Aquiry phase, as well as identifying periods of activity within the Aquiry Phase and for individual sites (KDE models; Extended Data Fig. 5). Several of the sites also contain evidence for previous and subsequent archaeological activity.

We are pleased that the referee #1 understands our methods and considers our Bayesian modelling and KDE models adequate to identify periods of activity within the Aquiry phase in general, as well as for individual sites in particular. This approach provides valid radiocarbon-based evidence for estimating both the period of time when the Aquiry geoglyphs were most actively constructed and the proportion of the geoglyphs that were in use. These estimates were essential in our attempt to provide a justified figure for the number of people living in the area during the peak period.

The usage of a mixed NH-SH calibration curve makes sense.

We agree. Although recalibrating every sample took a lot of time, the result was the most accurate possible.

I'm not so convinced that the KDE activity timings could really be translated into space (Supplementary Fig. 3). Perhaps this Figure could be left out without detriment to the arguments made.

Supplementary Figure 3 has now been included in the manuscript because Referee #5 required us to incorporate it into the chronological and spatial analyses, writing: "*What is needed is a map showing the distribution of the C14-dated sites*."

I agree that there is no need to provide much detail on the pretreatment of the charcoal samples, however please do cite a charcoal pretreatment paper (e.g., one of Bird et al. 2014, Gillespie 1997, Olsson & Kilicci 1964, or Wood 2015).

We thank Referee #1 for the observation, and the suggested citation is now incorporated into the revised manuscript.

Otherwise I'm happy with the responses to my comments.

line 312, "1,31-3,14" should be "1.31-3.14".

We thank Referee #1 for the constructive comment.

Referee #2

REVIEWER #2's NOTES ON THE REVISED PARSSINEN My characterizations of the key results, significance, and originality of the manuscript are as I wrote them in my original review. Briefly, the results are highly significant and robust, as they are based on a large, new database as well as all relevant earlier results by the authors and by others. The methodologies used for gathering the mapping data and the analyses employed are valid and well described. I found the authors' characterization of the uncertainties excellent (and unusually honest). I base my evaluation of the research on my many decades of research in Amazonian archaeology, ethnohistory, and ethnology as well as in tropical ecology.

The authors' answers to the reviewers' questions about radiocarbon dates and the added supplementary information about them and other details seem satisfactory and, in fact, improve the manuscript. Minor errors, such as typographic errors, have been corrected. The authors' term, Aquiry, for the Geoglyph area as now defined, has been clarified, as a reviewer requested. A few sections about which reviewers had questioned the organization of the writing have now had to be removed to limit the length of the paper. Those are all satisfactory outcomes, for me.

Considering that the referee #2 appears to be probably the best-known living specialist of Amazonian archaeology and anthropological history, well-known for her knowledge of Amazonian research traditions and contemporary research, we are glad to read her satisfaction with our revised manuscript and the final results.

The issue raised about "astronomical orientation" has been satisfied by editing the terms used in the article. I would like to mention, however, in support of the authors' interpretation of geometric images as elements of a shared cosmology, that such images used as symbols for astronomical bodies considered by current Indigenous Amazonians as important figures in myths of creation, are present in Amazonian rock art as early as c. 13,000 calendar years ago, in Monte Alegre, Para, Brazil.

Yes, it was somewhat disappointing that we could not expand on this topic due to the limited space. Nevertheless, the article in which some of us present statistical data on the 'astronomical orientation' of Amazonian geometric earthworks has now been accepted for publication. In the future, we are also willing to study more about links for astronomical bodies in ceramics, body paintings, rock art, etc., as well as in Indigenous oral traditions.

The authors are correct to mention, in response to the question by reviewer #3 about the possible effect of a well-forested prehistoric Geoglyph area, that Salati and his colleagues and others have shown that at least 75% of the rain in Amazonian forests derives from moisture stored and respired by the forest itself, not by moisture coming in from oceans, contrary to many researchers' expectations. If the Geoglyph makers followed the widely demonstrated current Indigenous

Amazonian practice of maintaining heavy forest cover through use of agroforestry techniques, then rainfall would have been much more regular and consistent than it is in now deforested or "savanna" parts of Amazonia. Thus, the lesson of the Geophyphs, as presented by the authors, is one very relevant to the current and future of Amazonia as a habitat.

Yes, indeed. We are impressed by the reviewer's understanding of our research findings on the relatively high density of ceremonial earthworks and the sustainability of the livelihood strategies of past Indigenous communities. These findings must certainly be taken into account when planning future land and forest use, as well as climate research.

I agree with the authors that reviewer #3's questions are in some cases based on an inaccurate grasp of the original manuscript and also on speculations that, although interesting, seem beyond the subject of the manuscript and in any case that would not enhance its relevance for Nature. In any case, the authors make several useful clarifications to address most of reviewer #3's more relevant comments and questions. I feel the need to mention that reviewer #3 seems to be influenced by some early assumptions about Amazonia necessarily having a limited carrying capacity because it is a tropical forest. Many of we archaeologists who work in Amazonia have done conclusive empirical research of various kinds that shows that the environmental limitation theory of Amazon prehistory has not been upheld. Furthermore, that reviewer seems acquainted only with Indigenous slash and burn cultivation, whereas numerous Indigenous peoples as well as acculturated Amazonian people (the majority of whose DNA is Indigenous, showing that despite no longer speaking Indigenous languages, they are long-term inhabitants of Amazonia) get most of their calories from tree crops, whether domesticated, cultivated, or wild. As recent researchers, whom the authors refer to in their response, show in various ways, permanent cultivation and harvesting of trees and shade-tolerant annuals and perennials, whether in terra firma or floodplains, was the main subsistence activity, supplemented by fishing and hunting. This type of land use has definitely been shown for sites in the Geoglyph region. Furthermore, reliance on forest utilization for a major part of subsistence would not leave any

particular type of landform such as what the reviewer seems to expect. As an archaeologist an environmental anthropologist, I do not find the population estimates of the authors "shocking" but, as I mention in my review, they seem consistent with those I made based on the sites I have surveyed and excavated in other parts of Greater Amazonia, some of which I mention in my review. I should also mention that my review of studies of pollen results carried out for both prehistoric and modern periods show no indication of wide-areal deforestation in prehistory. The percentages of grass that modern deforested or "savanna" areas have do not occur in prehistory in the places that have been studied (E.g., Roosevelt 2021 and Absy 1979).

All of these issues are highly important and provide valuable additional information for our discussions with Referee #3. Our own research results from diverse disciplinary backgrounds in the Aquiry area fully support these latest findings.

As for reviewer #5, who questions how population estimates can be made when the sites mapped are primarily ceremonial, one can point to the pattern of ceremonial meetings among living Amazonian societies, such as the female initiation rites among the Shipibo of the upper Amazon in Peru or the funerals of the Kayapo and neighboring groups in the middle Xingu. Both groups were very populous during the prehistoric period, as Myers shows for the Shipibo and their neighbors and as Posey has shown for the Kayapo. For these events, as I know from attending some of these ceremonials, people are invited to come from many residential communities in the surrounding area to central places, where they spend several weeks dancing, feasting, wrestling, trading, etc. Thus ceremonial constructions like the Geoglyphs would represent meeting places for a number of residential villages. Thus, making population estimates is a valid exercise, and the authors have made different estimates based on different possible community and family house sizes. The reviewer praises the results of the authors' Lidar mapping program and the statistical analysis, and the authors have satisfactorily addressed his/her suggestions, I think.

Signed

A. C. Roosevelt

As Referee #2 has internationally recognised in-depth knowledge of population estimation methods in the Amazonian context, as well as of Amazonian societies' deep past until the present time, we are grateful for these positive comments.

Referee #3

This manuscript presents two main results: (1) the quantification of the number of geoglyphs, and (2) the implications of this quantification in terms of estimated population density. While the first part represents "hard data," and our previous comments have been partially addressed through clarifications and improved analyses, the second part (see the paragraph entitled Estimation of the population in the revised manuscript) remains purely speculative and unsupported.

It seems the referee #3 considers his/her previous doubts to have been clarified in the first part of our manuscript, which presents the results of our LiDAR survey in terms of the number and size of earthworks. However, Referee #3 still objects to the latter part of the manuscript, regarding it as purely speculative. In our opinion, the referee´s comments on this matter are based on significant misunderstandings and misreading of the Amazonian context, as well as some irrelevant comparisons and statements, which we would like to address.

Their population estimation relies entirely on their own publication (Pärssinen et al., 2009), where they assert—without any supporting evidence—that building a geoglyph with a diameter of 200 m would require 80 individuals working for 100 days. This provides a metric linking the number of earthworks constructed to the amount of labor over time.

We appreciate the reviewer's point, but Pärssinen et al. (2009) clearly stated that their estimation was based on field-measured dimensions of a geoglyph, implying that 8,000 m^3 of soil was moved and that 1 m^3 of soil can be moved per day by one person. The estimate in Pärssinen, Schaan & Ranzi (2009) was based on the fact that, in any construction work, one person can dig approximately 1–2 m^3 per day with an iron spade

and hoe. The calculation presented was also based on late Denise Schaan's practical experiments, which were conducted at various excavation sites over several years and in different months. People in Schaan's experiments used iron hoes and spades instead of wooden tools. Therefore, we believe that our estimation of the number of people required to create ditched enclosures at these sites must be considered an absolute minimum. In addition, we would like to emphasise that our aim was to estimate the size of an average community that built and maintained a geoglyph, not just the number of people that was directly involved in the construction work. In a thriving community that could have both built and continuously maintained a geoglyph, there must have been adults performing daily domestic tasks in agroforestry, gardens, and cultivated fields as well as by fishing, hunting and gathering with those who prepared food for the workers. Additionally, one must consider that a community necessarily comprises elders, young people, children, breastfeeding mothers, and others who cannot contribute as much as healthy adults do. As such, the result was roughly an average of 300 people per geoglyph. Finally, to support the idea that our 2009 estimate was a minimum figure, we cited a 2018 estimate published in *Nature* which suggested that 500–600 inhabitants were required for each earthwork in the Southern Rim of Amazonia (De Souza *et al*. 2018). This second estimation was based on ethnographic evidence and a linear regression model.

As a response to reviewer's criticism that we had based our population estimate entirely on one paper and one line of logic, we would like to point out that we wrote the following on lines 284–286: "*Another [third] way to estimate the number of people in the pre-colonial Aquiry is to consider how many people lived in a typical mound village when these settlements first appeared in the same area in the early 13th century*", and we summarised the results of this comparison on lines 286–316. A more detailed presentation of this comparison can be found in Supplementary Information, lines 175–206. Between these two different archaeological phases, there are no signs of radical population replacement, but rather a clear continuity (see also Iriarte *et al*. 2021). The number of individual mounds (representing multi-family *maloca* dwellings) surrounding a plaza can be easily calculated using satellite imagery. These villages may offer the best indication of the size of the dispersed communities that chose to live together somewhat later in the core area of the ancient Aquiry. Therefore, by comparing population estimates for the mound phase with those for the earlier geoglyph phase, we

concluded that population density during the mound period was approximately 235 people per hectare. However, the average size of the mound villages was 300 people per earthwork, and these occupied a smaller spatial area than the geoglyphs. This provides independent evidence regarding the approximate size of the communities that constructed and maintained the geoglyphs.

A fourth piece of evidence supporting our argument and conclusion is the amount of space Indigenous communities currently require for traditional ceremonies in the same region, for which we have strong first-hand ethnographic evidence (lines 317–326). These ceremonies require an open space of one to two hectares (approximately 1.5 hectares for 300 people) and ceremonial roads. Hence, it can be supposed that the average 3-hectare geoglyph was planned for a maximum of 600 people. If around 300 of these people belonged to their own community, the remaining 300 were probably intended for visitors from other communities, as various historical and ethnographic sources suggest. Anna Roosevelt (Referee #2) also refers to the same point in the Ucayali region, Peru in her assessment. Thus, it is reasonable to assume that inviting more visitors than there were in one community would have posed a considerable threat in the event of a conflict. Finally, we presented a speculative and unlikely theory that if the mounds created by the large *maloca* dwellings were residences for individual families, the figure would be 60 people per earthwork. Thus, it is not adequate to claim that our estimation relies entirely on our own publication of the year 2009 without any supporting evidence.

However, in response to our main comment, the authors now claim that “it is not important how many geoglyphs were built per year.” Moreover, in the manuscript they state: “To calculate the population of the Aquiry, the first obvious question is: how many earthworks were in use at the same time?” (line 252, revised manuscript) and “if an average earthwork required 300 people to build and maintain it, we arrive at a total population of about 1.31–3.14 million” (line 276, revised manuscript).
I strongly disagree with conflating the number of people allegedly required to build the earthworks with those needed to maintain them or with those who might have used them simultaneously. These are three fundamentally different types of

activities. This confusion between metrics is repeated both in their responses to us and to Referee 5 (who raised the same concern), where they write that "the focus of this article is not on the number of earthworks constructed per year, but rather on the peak period during which the largest number of earthworks were used simultaneously." Here again, they shift from discussing the number of people needed for construction (the figure provided in their own paper) to discussing "simultaneous use" and "maintenance." Estimates of the number of people associated with each of these activities should be based on distinct and independent lines of evidence. Yet there is absolutely no supporting evidence for how they estimate the number of people who would have simultaneously used or maintained these geoglyphs. I strongly disagree with conflating the number of people allegedly required to build the earthworks with those needed to maintain them or with those who might have used them simultaneously. These are three fundamentally different types of activities.

In our opinion, Pärssinen et al. (2009) cannot be considered a valid reference for estimating past population densities, as it lacks any robust supporting argument; and even if the estimation in Pärssinen et al. (2009) were reliable, the metric used there concerns construction time per unit of labour. Therefore, it is essential that the authors consider, based on radiocarbon ages and their chronological model, how many geoglyphs were constructed per year...

Of course, the construction, ongoing maintenance, and use of earthworks are all separate activities that the communities of the Aquiry have carried out, even if they are partly overlapping. For example, based on the estimates in the 2009 article, if all the working-age men (one per family) were among the 300 community members, then around 60 men and 20 women (one per three families) would have to dig. They would work a minimum of 100 full days. On average, one geoglyph could be dug in a year. However, according to the oral tradition (collected by PKV) of some Indigenous peoples in the area, the division of labour in local communities was such that some residents were responsible for burning and clearing the forest, while others were

responsible for digging. Applying this information to the 2009 estimate would increase the minimum required excavation time to 200 days, but it would not necessarily change the required community size — only the division of labour would change.

Secondly, it should be noted that maintaining land structures in the Amazonian rainforest requires repetitive, careful cleaning of continuously growing vegetation. It is still a crucial act to prepare and maintain ritual plazas in Amazonia by careful collective cleaning activities. We have robust ethnographic evidence for this in Acre and Amazonas, e.g. diverse people cleaning in rows (field notes by PKV). Rainforest clearings quickly become overgrown if leaves are not cleaned and shoots are not removed at the root. These cleaning operations were generally reported by Europeans who arrived in the 19th century (e.g. Mercier, [1894] 1981), and this practice continues in many places in Amazonia today. For instance, Lathrap (1962) described how ceremonial areas and streets in Peruvian Amazonia were continuously cleaned and kept free of vegetation. Furthermore, our archaeological excavations at over 30 sites in the Aquiry region also prove beyond a doubt that the interior of the geoglyphs was always cleaned after ceremonies. As the places were used temporally, the ceremonial potteries also moved with people. It is often difficult to find diagnostic pieces of pottery or charcoal large enough for dating purposes inside the structures. However, over centuries of use, ash, dust, and tiny pieces of charcoal and pottery normally accumulated on the ground to a depth of between 20 and 40 centimetres, sometimes up to 70 cm. Pottery has also accumulated at the bottom of the ditches. In some cases, more of the cleaned soil drifted into the moats at the end of the geoglyphs' use (field notes by MP and SS). Additionally, it has been found that fire and sandy clay were also used for cleaning in places where food and drink were served, and many dishes were broken (Pärssinen 2021a; Pärssinen *et al*. 2021, and field notes by MP).While the exact organisation of these events remains uncertain, it is evident that these activities, along with the controlled burning of vegetation, were undertaken by the communities themselves. It is important to note that there is no evidence of a centralised state or itinerant construction workers in the Amazon region. If the same workers had designed and dug several geoglyphs, their similarity would certainly attract attention. However, this is not the case. We can therefore assume that the communities themselves did the digging, while the design work required the mathematical skills and expertise of a few individuals.

Finally, according to historical and ethnographic evidence, most people from each community participated in the most important local ceremonies, and important people were also invited from other communities for some specific ceremonies. An independent metric approximation of these people is given on page 8. During the historical period, drinking and dancing activities were extremely popular among the Brazilian Purus and Bolivian Baures (Eder [1772] 1985; Labre 1872), and Eder ([1772] 1985, 85) even argued that the only function of local chiefs, *arama*, was to invite his subjects to drink with "largesse". Nevertheless, drinking was not the only purpose of the gathering. Eder ([1772] 1985, 293–299) also mentions wrestling, archery, running, eating hot corn cobs, and, most importantly, ball games.

In summary, although burning, digging, cleaning, maintaining, organising ceremonies, feasting, and other uses of geometrically patterned earthworks are all different activities, it is quite clear that the same community organised all these activities in each particular ceremonial centre over long periods of time. Thus, two questions arise: how many of these earthworks were constructed per year during the peak periods, and how many of them were in active, simultaneous, yearly use, maintained by their corresponding communities? Archaeological excavations and the chronological layers observed and dated during the last 20 years confirm beyond a doubt that these sites were used for several centuries, sometimes for over a thousand years. Reports of these excavations are filed with the Brazilian Instituto do Patrimônio Histórico e Artístico Nacional, and most of their scientific results can be found in the reference list of this manuscript. A list of all available radiocarbon dates, along with the latest and most accurate calibration corrections as supporting evidence, can be found in the Supplementary Information (Supplementary Tables 1a and 1b). In addition, we used the currently recommended Bayesian approach and modelling to produce a Kernel density estimate of all geoglyph dates (see Supplementary Information, lines 39–74). We also produced a series of maps showing the temporal and geographical development of dated Aquiry sites using OxCal 4.4.4 (see Supplementary Figure 3). Furthermore, we have presented and analysed the overlapping KDE summaries obtained through calibration and Bayesian modelling (Extended Data Figure 5 and lines 251–261), which show that most of the radiocarbon dates obtained from chronological levels associated with human activity inside geoglyphs (e.g. shards, burnt clay, charcoal and burnt macrofossils) are clearly contemporaneous between 100 and 300 AD. These overlapping summaries include the

earthworks built during the period in question, as well as those built between c. 600 BC and 100 AD that were still in active use. In sum, this modelling suggests that up to 10,460 earthworks in total were used at least some moments during the period 100 - 300 AD. This kind of modelling is probably the most common way in which archaeological contemporaneity is indicated in current journals such as *Radiocarbon*, and the other analyses presented before this serve as supporting evidence. Thus, the argument that "*there is absolutely no supporting evidence for how they estimate the number of people who would have simultaneously used or maintained these geoglyphs"* is not true.

As said, when estimating the total population of the Aquiry, the most relevant question for us is when most of the earthworks were used over a short period of time. However, this does not mean that modelling techniques such as Kernel density estimation (KDE) are not useful for determining how many structures were constructed in a given period or year, even though these techniques do not take into account earthworks constructed before the calculated period that were still in use at that time.

Contrary to what the reviewer says, we did indeed estimate the number of geoglyphs constructed per year "*based on radiocarbon ages and their chronological model*". Between lines 252 and 255, we state that "*the densest overlap of calibration dates occurs at around 100–300 AD, both chronologically and spatially, corresponding to a 25% probability of the Aquiry earthworks being constructed, according to Kernel density estimates (see Supplementary Figure 2).*" In addition, between the lines 65 and 66 of the Supplementary Information, we write: "*The KDE model function estimates that 25% of construction activities date to the period 100–300 AD*." We clearly state that this 'independent' chronological activity model yields a figure of 4,360 earthworks per 200 years, equating to an average of 22 geoglyphs constructed per year between 100 and 300 AD. According to our various estimates, this would require an average community of 300 people, multiplied by 4,360, equating to 1.3 million people. Taking the previously mentioned multi-family versus single-family dwellings and visibility issues into account, the absolute minimum required would be between 370,000 and 800,000 people, but this is extremely unlikely.

Furthermore, we would like to point out that the figure for an average community of 300 people includes the calculation of the average labour force required to construct a geoglyph, which is at least 80 people digging 1 m^3 per day. Thus, if all 60 adult men of

each community of 300 people (accepting the nearly universal relation of 1 adult man per family of 5 individuals) and 20 women were needed for each construction for 100 days of the year, then the construction of 4,360 earthworks would have required 261,600 adult men in total. This corresponds (x5) to the same population of 1.3 million people. As we have argued, this is the minimum population size (for constructed 4,360 earthworks) required for the earthwork-building Aquiry society, provided the basic assumptions are correct. Furthermore, if all 80 earthmovers were local men, the average community population would have been near 400 people, and hence the minimum population would have been 1.7 million. Moreover, it is highly unlikely that communities five times smaller — in the case, if the later mound village dwellings used for comparison belonged to single families rather than general multifamily units — would have been able to construct 4,360 monumental earthworks over 200 years.

By using the variety of evidence presented above, particularly based on radiocarbon data, we argue that ca. 25–60% of all known geoglyphs were in simultaneous use over a period of 200 years from 100 to 300 AD. This means that, at any given moment over this period, there were ca. 4,360–10,460 geoglyphs in use. In other words, one must try to envision a human population that would be able to allocate enough labour to construct 4,360 geoglyphs and maintain these, along with thousands of other earthworks constructed before, in a sufficiently adequate condition for festivities, ceremonies, and other activities.

Finally, we are surprised by the opinion that one should look separately at the numbers of people required for building, maintenance, and use of the geoglyphs. Why speculate about these kinds of communal activities? In our view, the only reasonable assumption about the geographical pattern of population distribution of the Aquiry is that it basically consisted of thousands of communities, where each one was associated to a single or multiple geoglyphs that served as centres for festivities and ceremonies. If one accepts that, in most cases, a community with a single ceremonial centre was the operative unit in the study area, then it should be obvious what lines of evidence one can use to estimate the average population size of a community. A community must have been able to use, maintain, and perhaps build a geoglyph, unless it was just maintaining one that was built by the previous occupants of the community.

Accordingly, one should use all possible lines of evidence for estimating the population size that could continue these activities without jeopardising the sustainability of the community. This is what we have done in the manuscript, and we cannot understand what the utility of a separate analysis of the time and people needed for different activities would be, other than approximating the possible number of workers needed to excavate ditches. Estimates of the number of people associated with each activity do not tell us much about the total population, as it is highly likely that the same adults participated in various communal activities (including opening areas for earthworks by fire, digging ditches and opening roads, cleaning roads and earthworks, constructing communal storage and other buildings, sowing and harvesting crops, preparing drinks and food for ceremonies, and participating in celebrations and ceremonies). In addition, women, children, and elderly people have played a role in many of these activities. For these reasons, we cannot calculate the population more accurately. As we have already noted, our Indigenous colleagues have agreed with our estimation of how much space a gathering of 200–400 people would need for their most important traditional ceremonies (lines 320–326). Thus, further analysis should be left until more information is available.

Referee #5

The issue with civilisation is that it is being used in two different concurrent ways because it is an inherently vague, poorly defined term. If it were replaced by "cultural tradition" throughout the early part of the paper the implicit "quality" and "magnitude" inherent to the term "civilisation" would be absent. And the jump at the end of the paper to civilisation referring to massive, wealth state organised societies with large settlements (eg Greater Angkor) several orders of magnitude larger than the earthen enclosures would then be glaringly apparent. The authors would need to explain why comparability is claimed across several orders of magnitude to societies which are literate, had major state administrations and built very substantial structures.

Here, Referee #5's statement is unfortunately outdated. There are more than two ways the term civilization can be used, and in fact, the way in which the referee defines civilization was rejected in South American archaeological and anthropological discourse more than 70 years ago.

Over the last 150 years, hundreds of different definitions for civilization have been proposed. Because it seems to us that the referee does not know the vast literature on civilizations in the South American context, we must summarize some of our earlier writings. In anthropological and archaeological discussions, the concept has a long history and has been treated as an evolutionary process. For example, Edward B. Tylor (1871) stated that civilization signified "the most complex of human conditions." Somewhat later, Lewis Henry Morgan ([1877-1878] 1985) presented his more sophisticated evolutionary theory, how societies developed from savagery to barbarism and from barbarism to civilization (that included writing). This was based on Christian J. Thomsen (1848), and John Lubbock (1865) divisions of European pre-history, and a Roman poet Titus Lucretius Carus´s idea (ca. 55 BC: book V) that the development of humankind started from Stone Age to Copper/Bronze Age and developed from Copper/Bronze Age to the Iron Age of the Roman civilization. Thanks to Friedrich Engels (1884), the writings of Morgan became a part of Marxist theory of how societies developed during their journey toward communism.

At the beginning of 20th century, many other evolutionary theories appeared. Among the most influential were the theories of Spengler and Toynbee. Of these, the British historian Arnold Toynbee collected information of more than 20 independent world civilizations and compared their rise and fall in his 12 voluminous mayor work: *A Study of History* (1934-1961). From the Americas, Toynbee accepted four full entities to his list: Andean, Mexican, Yucatec, and Mayan civilizations. He concluded that civilizations rose mainly due to environmental and other challenges that were resolved by creative individuals and minorities forming elite leaders.

Also, it is important to note that North American anthropologist Julian Steward (1955) opposed Morgan´s original ideas. Adopting some ideas of Toynbee, Steward argued that instead of a unilineal and universal track, human cultures had multilinear development possibilities dependent on environmental, technological, and regional factors. Writing, iron technology, or state administration were not considered necessary conditions for civilization. When Steward edited the Handbook of South American Indians (1946-1950/1959), he saw the Andes as the focus of higher civilizations. While volume I of the Handbook, edited by Steward, was dedicated to marginal hunting and gathering tribes, volume II focused on Andean farming civilizations. Volume III dealt with horticultural, hunting and gathering tropical forest tribes, and the fourth volume detailed

Circum-Caribbean tribes. The level of civilization is only specifically mentioned in the Andean context, when referring to Chavín, Moche (Mochica), Tiwanaku, Inca, and Columbian Muisca (Chibcha) cultures. Also, volume I is an exception among the marginal tribes: there, a Swiss writer, Alfred Métraux (1946, 209) mentions the Diaguita culture in Chile and Argentina as a civilization due to the high quality of their geometrically painted polychrome pottery and highly developed social organization.

The Handbook of South American Indians formed a standard model for South American civilizations for a long time, which Indigenous nations themselves and many decolonial researchers have shown to be wrong in many publications. It is crucial to note that at the time when Handbook was edited, Amazonian archaeology was only developing, and Acrean geoglyphs, among others, were unknown. Therefore, it is understandable that Steward believed the tropical forest to be ecologically unsuitable for cultural development toward civilization. Their claim can only be understood if one knows well the current Amazonian research and genealogy of Amazonian archaeology. Amazonian societies were mainly seen as "primitive" egalitarian tribes living in small impermanent hamlets in a hostile environment and without any complex social organizations (Steward & Métraux 1948; Steward & Faron 1959). This idea was maintained until the end of the last century (Meggers 1992). Nevertheless, our understandings of past human development started to change radically around 30 years ago, as researchers started to realize the effects of European diseases on Indigenous populations (e.g. Denevan 1992, 205-234; Dobyns 1966; Roosevelt 1991) and anthropological research was conducted in the Lower and Central Amazon river, in the Xingú, as well as in the Bolivian Mojos and Baures regions (Erickson, 2000, 2006; Heckenberger *et al*. 2003, 2008; Neves & Petersen 2006; Roosevelt *et al*. 1991; Roosevelt 2013). There, highly developed, mainly multicultural civilisations were found that built monumental earthworks, including dwellings and/or ceremonial centres with roads/causeways and canals. These societies also shared religious views and iconographic trends, and they domesticated various edible plants. The earliest examples of Amazonian civilizations are fully comparable to the early civilisations of the Andes, as evidenced, for example, by the widespread Chavín religious cult, whose iconography and construction of monumental ceremonial centres is comparable in size to those of the Aquiry civilization. Chavín is also known to have been significantly influenced by

Amazonian cultures. Consequently, many authoritative publications, such as *Science* and *Antiquity*, now use the term civilization to refer to Amazonia as well.

Today, civilization is used to speak about Western or Christian civilization, Chinese civilization, Islamic civilization, ancient Andean and Mesoamerican civilizations, and the like, referring general religious, ideological, and cultural features (churches, mosques, pyramids, etc.) within greater multicultural geographical areas of complex societies (see also Spinney 2025). We may well add ancient Amazonian civilizations to this list as well, as proposed by Heckenberger et al. (2008), among others. In the case of the Aquiry, the main cultural features were their ceremonial centers, monumental geometrically patterned ditched earthworks (due absence of suitable stone) with wide and narrow roads, and likely some huge wooden buildings that have already disappeared. Finally, it is also important to note that the 19th century meaning of the term civilization is not the one that the Oxford and Cambridge English Dictionaries use today. For example, OED https://www.oed.com/dictionary/civilization_n?tab=meaning_and_use#9266268 gives seven entries for the noun civilization, of which the nearest of our field are the followings:

-The state or condition of being civilized; human cultural, social, and intellectual development when considered to be advanced and progressive in nature. Also extended use.

-With modifying word. The culture, society, and way of life of a particular country, región, epoch, or group. E.g., Islamic civilization, Neolithic civilization, Christian civilization, Egyptian civilization, etc.

According to the Cambridge online-dictionary the concept of civilization means:

(1.) Developed society, in general, and
(2.) human society with its well developed social organizations, or the culture and way of life of a society or country at a particular period in time, or
(3.) a highly developed culture, including its social organization, government, laws, and arts, or the culture of a social group or country at a particular time, in particular (see, https://dictionary.cambridge.org/dictionary/english/civilization)

These definitions are consistent with our understanding of how the term "civilization" should be used in English. However, if we were to revert to the old Eurocentric definition of civilization from the 19th-century Morgan–Engels school, which encompasses the notions of a requisite hierarchical state and Eurasian-style writing systems, we would face significant challenges and likely accusations of neo-

colonialism. We do not want to adopt this Eurocentric definition of "civilization," and we argue that most Great Andean multicultural complex societies, such as Chavín and Tiwanaku, are rightly called civilizations. The earlier Chavín civilization was structured around ceremonial centers such as Chavín de Huantar, the best-known center of Chavín civilization. It was about 7.5 ha of its size during c. 1000 – 500 BC, and only after this older period, the size of the center duplicated to 15 ha, c. 500 – 200 BC (Burger 1992, 161). Thus, it is not extremely big. The largest geoglyph in Tumichucua (Bolivia) covers an area of 50 hectares. When combined with other earthworks, the entire archaeological site encompasses an area of 120 hectares (Arnold & Prettol 1988; Saunaluoma 2010; Pärssinen, Kalliola & Ranzi 2024).

We interpret the referee's citation to 'Greater Angkor' as referring to the following passage from lines 377–381: "*However, unlike many other tropical civilizations in the world*[16,48]*, it appears that large-scale water management projects with raised fields and causeways were not a major focus of their subsistence strategies in extensive interfluve environments, although the presence of water appears to be one of the key elements for the earthwork sites and their roads*[3]."

Here, note 16 refers to Prümers et al.'s research in Llanos de Mojos, Bolivia, and note 48 refers to Lucero, Fletcher & Coningham's article "*From 'collapse' to urban diaspora*," in which the authors compare Angkor (c. 750,000 people) in Cambodia, Anuradhapura (c. 250,000 people) in Sri Lanka, and the Maya capital of Tikal (c.60,000 people?) in Guatemala. In these tropical regions, the management of different kinds of waterworks was crucial for the maintenance of administrative and socio-political systems, and after the breakdown the inhabitants largely abandoned the urban centres. Finally, "*former subjects continued farming, but many did so in different areas within smaller community networks and engaged in a different kind of urbanism on the periphery of the former states*" (Lucero *et al.* 2015).

The question is therefore merely a general statement that the Aquiry is different from many other tropical civilizations that had large-scale water management projects, including raised fields and causeways. Although some canals are known to exist in the Aquiry region (e.g. Tumichucua), raised fields and causeways are absent. We have discussed omitting this statement at the time, but it was kept in because Referee #5 had specifically asked us to note the comparison with Mesoamerica. As we could not fit in

any new references, we left it in. However, if the referee is bothered by the fact that the article compares Greater Angkor, which had a population of nearly one million, to Tikal, which was more than ten times smaller, then the entire reference can be omitted.

Urban is used because it is term which possesses cachet and has rapidly gained a vague and plastic identity as an academic category. The authors have many companions worldwide in this practice of overextending the term urban.

Since there is no such thing as standard urban density, it is sometimes difficult to assess when the term is being used too broadly. However, this issue has been addressed in previous articles (e.g. Pärssinen, 2021b; Virtanen, 2023), which show a different idea of Amazonian urbanity, and we refer to these articles and other supporting articles in our manuscript. In particular, we highlight how the latest research addresses this issue in relation to the Amazonia. Unfortunately, the reviewer appears to be unfamiliar with this discussion and has not read the referenced sources from the manuscript carefully enough.

For instance, Pärssinen (2021b) compared the Aquiry civilization with the Greek city-states, referencing the work of researchers at the University of Copenhagen in Denmark who catalogued 1,035 Greek *poleis* stretching from Spain and France to Libya and Asia Minor via current Greece. The Greek civilization lasted about 1250 years, from 750 BC to AD 500. Hansen (2006) estimates that some 500 *poleis* are still missing from the Danish inventory, but in general some 850 – 1000 city-states made up Greek civilization during the Archaic and Classical periods (c. 700 – 323 BC). Most Greek city-states were fortified by walls, and they had a plaza for markets and public assemblies. The superficies of smallest city-states had less than 8 ha and their entire defensible territory reached up to c. 10 km² while the most typical *poleis* had c. 27 ha behind walls and their defense circuit varied between 25 and 100 km². Only some 10% of city-states, including Athens, consisted of around 182 ha or more.

Today, we estimate hundreds of the Aquiry sites that contain two, three, or more geoglyphs and cover more than 20 ha. It is also important to consider that it was calculated that the public space of medium size Greek city was c. one quarter of the fortified area, and the rest of the walled city was dedicated for housing and private use. Thus, it was estimated that some 6 – 7 ha was dedicated for ceremonial and social purposes in a typical Greek city. If we now compare this to the ceremonial centers of

the Aquiry, the difference is not very big. The biggest known geoglyph has 50 ha in Tumichucua that covers, as an archaeological site, 120 ha in total, and it is likely that more than a thousand of the Aquiry individual geoglyphs reach 6 – 7 ha. These facts, as well as many other common aspects, indicate that we may speak about civilization and some kind of urbanism, such as low density or forest urbanism, in the connection of the Aquiry.

Furthermore, we have keenly followed the discussion of different kinds of urbanism, especially in the Amazonian context and even in other areas. Among others, we have noted that Lucero, Fletcher & Coningham (2015) seemingly refer to Postclassic Maya culture as smaller communities "*engaged in a different kind of urbanism on the periphery of the former states*." In our area, we do not claim that villages that appeared after the collapse of the Aquiry would have shown any further marks of urbanism. In every case in our manuscript, we refer to articles by leading researchers that deal with pre-colonial Amazonian urbanity. The list of citations is as follows:

1. Roosevelt, A.C. The Amazon and the Anthropocene: 13,000 years of human influence in a tropical rainforest. *Anthropocene* **4**, 69-87 (2013).
2. Heckenberger, M.J., Russell, J.C., Fausto, C., Toney, J.R., Schmidt, M.J., Pereira, E., Franchetto, B. & Kuikuro, A. Pre-Columbian Urbanism, Anthropogenic Landscapes, and the Future of the Amazon. *Science* **321**, 1214-1217 (2008).
3. Neves, E.G. Was Agriculture a Key Productive Activity in Pre-Colonial Amazonia? The Stable Productive Basis for Social Equality in the Central Amazon, in *Human-Environment Interactions, Current and Future Directions* (eds. Brondízio, E.S. & Moran, E.F.) 371-388 (Dordrecht: Springer, 2013).
4. Lombardo, U., Hilbert, L., Bentley, M.K., Bronk Ramsey, C., Dudgeon, K., Gaitan-Roca, A., Iriarte, J., Mejía Ramón, A.G., Quezada, S., Raczka, M., Watling, J.G., Neves, E.G. & Mayle, F. Maize monoculture supported pre-Columbian urbanism in southwestern Amazonia. *Nature*, online 29.1.2025. https://doi.org/10.1038/s41586-024-08473-y3
5. Prümers, H., Betancourt, C.J., Iriarte, J., Robinson, M. & Schaich, M. Lidar reveals pre-Hispanic low-density urbanism in the Bolivian Amazon. *Nature* **606**: 325-358 (2022).
6. Rostain, S., Dorison, A., de Saulieu, G., Prümers, H., Le Pennec, J-L., Mejía Mejía, F., Freire, A-M., Pagán-Jiménez, J. R. & Descola, P. Two thousand years of garden urbanism in the Upper Amazon, *Science* **383**, 183-189 (2024).
7. Virtanen P.K. Urbanity in Ancient and Present-Day Southwestern Amazonia. Human-Environment Collectives, Cycles of Generosity, and Their Ruptures, in *Urban Imaginaries in Native Amazonia: Tales of Alterity, Power, and Defiance*

(eds. Santos-Granero, F. & Fabiano, E.) 205-232 (Tucson: The University of Arizona Press, 2023).

After comparing these (and many other) cases, we are convinced it is correct to speak about different kinds of tropical, low-density urbanism in all these contexts, including the Aquiry. Unfortunately, due the fact that some of us have analysed this topic already elsewhere, it does not make sense to give a new comprehensive analysis in this article. On the administrative aspects of geoglyph building society, please see also Virtanen & Saunaluoma (2015, 2017) and Pärssinen & Ranzi (2020). Finally, we would like to cite forthcoming comments of Kirrily White: "*dense populations? Again it's problematic – but it is frequently used in the literature without clarification of what dense means and the discussion in Amazon is on types of urbanism, so it isn't an outlier in terms of literature from other regions*."

And dense is readily used by the authors when it is not an appropriate descriptor. A region of 183,000 sq km with an average density between 0.07 persons per ha and 0.2 persons per ha, at maximum, is not densely occupied. Extensive dense rural provinces would have overall densities in the 2-5 p/ha range.

This comment is interesting. While Referee #3 has argued that it is impossible for the area to have been so dense that the population reached up to 3 million people, Referee #5 states that 3 million is not dense at all. If we understand correctly, the population of the Aquiry should have been between 36.5 and 91.5 million people (two to five people per hectare) before it could be considered dense. These amounts correspond to the general population estimates of all pre-colonial double continents of the Americas, and all of them should have lived in the Aquiry before we could call the area an "*extensive dense rural province*."

For clarity, we consider an area of 85,000 km² to be relatively dense, not 183,000 km². Secondly, the concept of density is relative, with fuzzy logic, and depends on what we are comparing it to. Referee #5 does not mention where he obtained his universal density values, but he may have his own Interaction-Communication model in mind (Fletcher, White & Penny, 2024), which was developed to study the internal residential density of settlements. We do not know. Nevertheless, we are not talking about the

internal density of settlements when we talk about the Aquiry population density, because the settlements themselves have yet to be discovered.

In every case, we prefer to use these concepts relationally according to the context in which they are employed, as most researchers currently do, until researchers have debated and agreed on the use of our referee's universal values of low, medium, and high population density. Meanwhile, we will ensure that this relationality is clearly expressed throughout our manuscript (e.g. relatively densely populated region).

Comparison to other societies produces further issues - the Neolithic enclosures and megalithic monuments of Europe are considered to have had public, ceremonial and political functions in relation to visits by mobile regional populations. The monuments display mathematical and geometric competence and involved cosmological understandings. They required organised labour. Are they, on the analogy proposed by the authors therefore urban? -a term which specialists who work on Stonehenge reject. And has repeatedly been pointed out large structures do not require large amounts of labour. The population estimates made on the basis of maintenance costs are problematic because the evidence is not provided that earthen embankments stabilised by vegetation need substantial maintenace.

Firstly, as mentioned earlier, it should be noted that maintaining structures in the Amazonian rainforest requires careful repetitive cleaning. It is still a crucial act to prepare and maintain ritual plazas in Amazonia by careful collective cleaning activities. Rainforest clearings quickly become overgrown if leaves are not cleaned and shoots are not removed at the root. We do not know about Stonehenge. However, as we have described earlier: "*These cleaning operations were generally reported by Europeans who arrived in the 19th century (e.g. Mercier [1894]1981), and this practice continues today in many places in Amazonia. For example, Lathrap (1962) described how ceremonial areas and streets in Peruvian Amazonia were continuously cleaned and kept free of vegetation. Furthermore, our archaeological excavations at over 30 sites in the Aquiry region prove beyond doubt that the interior of the geoglyphs has always been cleared before and after ceremonies. As the places were used temporally, the ceremonial potteries also moved with people. It is often difficult to find diagnostic*

pieces of pottery or charcoal large enough to date inside the structures. However, over centuries of use, ash, dust, and tiny pieces of charcoal and pottery have accumulated on the ground. Pottery has also accumulated at the bottom of the ditches, and in some cases, more of the cleaned soil has drifted into the moats at the end of the geoglyphs' use (field notes by MP and SS). Additionally, it has been found that fire and sandy clay were also used for cleaning in places where food and drink were served and many dishes were broken (Pärssinen 2021a; Pärssinen et al. 2021; and field notes by MP)."

To our knowledge, the size of the original circular ditch of the Stonehenge ceremonial centre is approximately 0.785 hectares. In comparison, the Tumichucua circular ditch covers an area of around 50 hectares and has a total area of around 120 hectares, making Stonehenge a relatively small site. The average size of the Aquiry sites is 3 hectares, and there are also hundreds of sites with earthwork concentrations covering an area of 20–40 hectares. However, it is unclear whether the Stonehenge area could potentially comprise thousands, or even up to 20,000, similar sites, or sites that are three times larger on average, within an area of approximately 85,000 km². This is a central question. If this can be proven, then we could consider whether we could also talk about Neolithic forest urbanism in Europe using an analogy.

A specific set of data which is required in this paper is the size distribution of the earthen enclosures. Does it have a characteristic form of many small features, some medium sized features circa 10-14 ha perhaps, a a few rare larger ones at circa 50 ha? Readers need to know, because reference to average sizes is problematic when a massive skew to a few large cases is occurring.

This is a bit odd, given that the sizes of all dated earthworks are explained in the text, and the sizes of the 406 earthworks discovered by our LiDAR project are listed in the Supplementary Table 2. As this article deals with the results of the LiDAR project, our focus is on this material. Looking at this list, it can be seen that the size of earthworks "tends to vary between 0.35 and 14 hectares" (lines 79–80). This means, as everyone can easily see, that there are not any significant jumps between the sizes that gives an average 3 ha. We also note that some known earthworks are large, covering up to 50 hectares, and clusters of various earthworks in the same site exist. As these issues have already been dealt with in previous studies, we have only provided references to some of those articles.

Furthermore, the coordinates of 1,279 earthworks, which were previously known mainly through satellite imagery, have been published as a supplement to *Acta Amazonica* (54/4, 2024):

https://doi.org/10.23729/31c411bf-5d94-4cfc-be06-006c16a5c309 (Dataset>You can open the new version via this link> Data> Files> Ancient earthworks)

We have used this data to calculate the average size of the Aquiry type earthworks in the Blocks 1, 2, and 5. Nevertheless, this data and its copyrights belong to another project coordinated by the University of Turku. The referee may request unpublished information on the size of the earthworks listed in the supplementary material of *Acta Amazonica* via the following email address: riskall@utu.fi.

The occurrence of the earthen enclosures over time also needs to be reconsidered. Even with a claimed peak period of only 200 years (note - in a 1500 year tradition) in the 85,000 sq km denser area 22,400 enclosures generates 4 per sq km ie 1 on average in a 25 ha area ie "half" an enclosure in a century at peak activity. This surely is not substantial or rapid construction. Is the total population actually an aggregate of all the people who have lived in a region over a span of time rather than a maximum population present at any one time.

What can be considered a "substantial amount of construction" is also a fuzzy and relative expression. If one expected that the entire population of North and South America (or between 36.5 and 91.5 million) had to have lived in this particular area before it could be considered substantial or dense, then constructions made by 1.3 million would be a low amount. However, we can return here to one of our main points that the Amazonas covers 6.5 million km², and it is predicted that there are between 10,272 and 23,648 undiscovered pre-Columbian structures in the area "*with 95% probability giving an average of 16,187 sites*" (Peripato et al. 2023). From this point, 22,400 enclosures in an area of 85,000 sq km could indeed be considered relatively substantial. This is because the cited area represents only 1.3% of the entire Amazonia (which is 76.5 times bigger than our 85,000 km²).

We used the OxCal 4.4.4 KDE Model function to estimate the relative intensity of human activity. This identified a local maximum of the Aquiry period in the calendar-year probability distribution between 250 BC and 400 AD (see Supplementary Fig. 2). This suggests that the construction of dated structures belonging to the Aquiry cultural

phase peaked during this period. Judging by the ratio of the KDE distribution sums during the peak and full periods, as well as the associated uncertainties in the KDE plot, the peak corresponds to 61% ± 20% of the full KDE distribution. Since the dates were derived directly from the structures, it can be estimated that approximately 60% of them were constructed during the peak period.

As explained in the manuscript, Supplementary Figure 2 illustrates the relative intensity of the entire Aquiry phase. However, it does not fully account for the fact that many earlier earthworks were still in use during the construction peak around 200 AD (see Extended Data Figure 5 and Supplementary Figure 3). The KDE model function estimates that 25% of construction activities date to the period 100–300 AD. This equates to 22 per year. Therefore, it is also important to examine how many earthworks were actually in use, and how many were cleaned and maintained, during this period according to 14C dates. Examining the overlap of the radiocarbon dates of all sites using KDE-modelled summaries (see Extended Data Figure 5) reveals that, around 200 ± 100 AD, approximately 60% of the dated centres were in use simultaneously, with a considerably high probability. All the radiocarbon dates relating to ceremonial activities in the Amazonas region (n = 4) and Bolivia (n = 3) fall within this period, as do around 60% of Acrean geoglyphs. Taking into account all uncertainties, we estimate that there is a 25–60% probability that all earthworks were in use simultaneously around 100–300 AD, with a strong preference towards the upper limit. It is estimated that during this period, approximately 4,360–10,460 Aquiry earthworks were in use within an area of 183,000 km², and around 4,043–9,702 earthworks were in use within an area of 85,000 km². This equates to 0.048–0.114 earthworks per sq km, which, when multiplied by an average population density of 300, equates to 14.3–34.2 people per sq km in the 85,000 sq km core area. This aligns with the concept of low-density urbanism presented in the Amazonian context. For example, after we submitted this manuscript for *Nature*, Hirst and others published an article where they wrote: "*Our results indicate that the Casarabe Culture [in the Bolivian Mojos] likely grew to a maximum population of between 10,000 and 100,000 people within a 5020 km² quadrant of their former territory, representing a density of between 2 and 20 people km^{-2} . These values…support previous interpretations that this culture practiced a form of low-density urbanism*" (Hirst *et al.* 2025, 1/29).

As with most estimations of this kind, researchers specify the time period during which the population maximum is achieved at sites that were used during various centuries. For instance, De Souza et al. (2018) assert that the maximum extent of the earthworks was reached at some point between 1250 and 1500 AD in the Southern Rim. Similarly, Dyer (2000, 508) writes that, in later medieval Britain, there were likely 861 small towns, and that, in any one year between 1270 and 1540 AD, around 700 of these were functioning as urban communities, and so on. We would argue that the maximum was probably reached in the Aquiry area at some point between 100 and 300 AD.

Note that a Britannica reference - 39 - is not sufficient nor is the use of Curet 1998 as a source for population estimates that is ove 25 years old.

We cited *Encyclopaedia Britannica* because it summarised the size and number of German towns in the 15th century well, which we know to be true after reading dozens of articles on the topic. However, if *Nature* does not allow articles on population history published in the *Encyclopaedia Britannica* to be cited ("*German society, economy and culture in the 14th and 15th centuries: Urban Life*"), we can adjust our general comparison to focus on later medieval British towns, as discussed in the *Cambridge Urban History of Britain* (2000). This can be done with two references. However, we do not understand why we cannot mention Curet's (1998) article published in *Antiquity*, in which a linear regression model was created specifically for estimating prehistoric populations in Lowland South America. This is the same model used by De Souza et al. in their *Nature Communications* article in 2018, which is discussed in our manuscript (lines 43–45, 235–241). Furthermore, we agree with Curet that direct comparison of the spatial size of settlements and ceremonial centres is not recommended due to differences in their functions (lines 292–293).

Kirrily White has also added some additional specific comments:

p. 25 – are they suggesting that state hierarchy was required to construct the ceremonial etc. earthworks in Acre?

Page 25 ? We are not suggesting that in any place of the manuscript. On the contrary, in various earlier articles we have demonstrated that it is unlikely that any hierarchical

state would have been involved when Acrean earthworks were constructed. As ethnographical and ethnohistorical examples from Ceylon, Philippines, the Peruvian coast, or Bolivian Baures, for example, demonstrate that impressive earthworks can be built and maintained without demanding state organization (e.g. Pärssinen 2021b).

p. 26 – addressing of mobility seems adequate, there is insufficient information available to date appearance of domestic flora in the area, or to understand the dating and structure of mounds which have not been previously identified as later mound villages.

???

p. 27 – they have provided more information on the radiocarbon dates, locations, and areas – see file 535106_0_supp_4929181_t4ll10, (supplementary data) lines 45-174. Dated sites are limited but are distributed over a largish area of the Acre earthworks and a few sites in Bolivia and Amazonas. There is tabulated information in the supplementary data.

Yes

p. 28 – they have addressed the density of occurrence of geoglyphs in the dating ranges. Most of the dates are for Acre. There is more information about this in the supplementary material. There is a series of maps in the Supplementary material on p. 4 which shows distribution of dated sites by blocks of time. There are also some sites with multiple dates showing phases of use. See also lines 117-174 on pp. 6-7 in the supplementary material. I think that without identification of occupation areas, they have to go on the size of the earthworks or maybe conjoined earthworks, so that the sizes are all small.

OK. However, it should be recognised that 'small' is also a fuzzy and relative concept. In the Amazonian context, Acrean earthworks, as public ceremonial spaces of their communities, have comparable sizes to many other cultural areas considered to be

examples of low-density urbanism (e.g. Heckenberger *et al*. 2008; Prümers *et al*. 2022; Rostain *et al*. 2024; Lombardo *et al*. 2025).

As in this article, the size and density of Indigenous peoples' ceremonial centres are used to estimate population size. Similarly, the size and density of churches in medieval Christian cities have been used for this purpose in Europe. Sometimes it has been the only possibility, and other times those have been compared to other available historical or archaeological information (e.g. Gauthier *et.al.* 1986–2014; Buringh 2021). Furthermore, while the question of different forms of urbanism and settlement size in different areas and periods is interesting, it is not the main focus of this article. Here, we examine the size of the Aquiry area and the number of ceremonial centres, attempting to make a preliminary population estimate based on currently available information.

p. 29 – see note below for supplementary data on population size, they don't have much to go on, and some discussion of population seems to be needed.

What is the note the reviewer is referring to? Considerable discussion on population is provided especially in lines 242-326 of the main text, and in many parts of the Supplementary Information, especially in the lines 175-206.

Supplementary Data

pp. 2-3, lines 47-74. I am concerned that they are not interpreting the KDE data appropriately by using percentages, would like to discuss it with you, but might need to consult a mathematician.

A mathematician and specialist of a radiocarbon chronology is a member of our research group -- his laboratory has realised the KDE modelling, and he has also given percentages. Percentages were also confirmed by an archaeologist and some other specialists before being added to the manuscript.

pp. 7-8, lines 175-206 discussion of population size. Importantly, they are talking about very small villages and I am aware from Roosevelt (1991. Moundbuilders of the Amazon: Geophysical Archaeology on Marajo Island, Brazil. Academic Press, San

Diego, p.342.) that multifamily longhouses could have 35-60 occupants. In her thesis (pp.171-72), Denise Schaan uses this estimate to propose 12-60 people per building for Camutins mound complex on Marajo Island – the range accounts for the different sizes of houses and mounds.

As one can see, we have used a number situated near the lower limit (25) to avoid accusations of exaggeration.

The way the population estimates are presented in this paper for the geometric earthworks does not seem to be outside the norm for literature in the Amazon regions even though there is not yet occupation evidence, besides perhaps size of earthworks, to base them on and all they can do is use what researchers have said about other regions.

OK.

pp. 8-14 is discussion of lidar methodology. I can't really assess this, but explanations and data appear to be thorough.

OK.

pp. 20-28 is a list of site sizes measured from the satellite and lidar imagery.
OK.

Revised.

p. 2, lines 40-46 – dense populations? Again it's problematic – but it is frequently used in the literature without clarification of what dense means and the discussion in Amazon is on types of urbanism, so it isn't an outlier in terms of literature from other regions.

OK. This is an important point that we have emphasised. We will ensure that this relationality is clearly expressed throughout our manuscript, for example by using the term 'relatively densely populated region'.

Lines 51-52 – again the issue of justification of population estimates. Does the provision of the areas of earthworks help? They have no occupation data and are using comparison with other regions, the size of mounds used at a later time period and ethnographic analogy – is it sufficient?

Ethnographic analogy has been among the most used method in Amazonian population estimations, as well as the size and amount of the habitational mounds. As said, in Europe the size and amounts of cathedrals, churches and basilicas have also been used in population estimations as a line of research. These are our current possibilities, and we are convinced these allow us to give best possible estimation at the moment.

p.4, line 97 – Comparison with Inka, it is comparing apples and oranges.

What is wrong with referring to similar administrative principles in the Inca state? It seems that the referee knows little about the Inca State and has not read the texts to which we are referring, which she should have done. We also advise reading comparative books like *Rethinking the Andes–Amazonia Divide. A cross-disciplinary exploration* (eds. Pearse, A.J., Beresford-Jones, D.G. & Heggarty, P., 2020) and *Interpretando Huellas. Arqueología, Etnohistoria y Etnografía de los Andes y sus Tierras Bajas* (ed. Muñoz, M.A., 2018). Firstly, comparisons between Andean and Amazonian cultures have revealed close ties spanning over 5,000 years. Secondly, the Incas occupied part of the former Aquiry territory. This area was part of Tawantinsuyu, in a quarter known as Antisuyu. It is a well-known fact that the Incas did not introduce radical changes to the local administrative principles in conquered territories. The nearest Inca fortification, Las Piedras (Pärssinen, 2015), is located less than 20 kilometres from the Aquiry site of Tumichucua, and some of us have participated in various international conferences where comparisons have been made between Andean and Amazonian cultural traditions and practices.

As our referee does not appear to have read the texts to which we are referring, we would just like to point out that when discussing different types of South American urbanism, it is important to remember that many provincial capitals of the Inca state were also used for feasting and religious ceremonies. Inca provincial capitals, such as Huánaco, Pumpu, Jauja and Paria, were centres where Inca governors, representing the

ruling Inca, invited local people — who lived in small villages and periodically performed work for the Inca state and church — to demonstrate the generosity of the ruling Inca and redistribute gifts stored in warehouses (Morris 1982, 162–166; Pärssinen 1992, 275). The only permanent inhabitants of these urban-like centres, which covered an area of around 100–200 hectares (Pärssinen *et al*. 2010), were a few hundred women and their guards who prepared food and beverages for the invited guests. When the Inca Empire collapsed in the 16th century, these centres were abandoned.

p.5 lines 110-11 – it's not wrong to say that researchers do use these criteria, just wrong that they do use it – all kinds of hunter gatherers built earthworks and practiced arts etc…

Of course, all human communities practised some form of art, and small mobile groups also built significant monuments. Here, we understand that the referee is criticising earlier researchers, such as V. Gordon Childe, who are discussed in lines 110–11. However, does the referee argue that all kind of hunter gatherers built thousands of geometrically planned monumental earthworks in forested areas with five meter deep, over ten meter wide, and up to 2.5 km long ditches together with hundreds of kilometres of wide roads? We would like to know where all kinds of hunter gatherers may have built thousands of similar monumental earthworks and roads.

Most of the paper is description of the data and discussion of different scenarios of interpreting population sizes. It might be helped by a discussion of population mobility, but they are not claiming large cities, just piling on the discussion of urbanism elsewhere in the Amazon literature. I am confused about whether or not they are trying to claim the Aquiry are a state as well as a civilization…

As said earlier, we are not claiming that the Aquiry was a state, but we agree with Heckenberger et al. (2008) and many others that in this Amazonian context we may speak about ancient civilization.

Cited references:

Arnold, D. & Prettol, K. Aboriginal earthworks near the mouth of the Beni, Bolivia, *J. Field Archaeol.* **15**, 457-465 (1988).

Burger, R. L. *Chavin and the Origins of Andean Civilization.* (London: Thames and Hudson, 1992).

Buringh, E. The Population of European Cities from 700 to 2000. *Res. Data J. Humanit. Soc. Sci*. **6**, 1-18 (2021).

Curet, L.A. New formulae for estimating prehistoric populations for lowland South America and the Caribbean. *Antiquity* **72**, 359-375 (1998).

De Souza, J.G. *et al*. Pre-Columbian earth-builders settled along the entire southern rim of the Amazon. *Nature Communications* **9**, 1125 (2018).

Denevan, W. M. The Pristine Myth: The Landscape of the Americas in 1492. *Annals of the Association of American Geographers* **82**, 369-385, 1992.

Dobyns, H. F. Estimating Aboriginal American Population: An Appraisal of Techniques with a New Hemispheric Estimate. *Current Anthropology* **7**, 395-416, 1966.

Dyer, C. Small towns 1270-1540, in *The Cambridge Urban History of Britain* (ed. Palliser, D.M.) 505-538 (Cambridge: Cambridge University Press, 2000)

Eder, F. J. *Breve descripción de las reducciones de Mojos ca. 1772* (ed. Barnadas, J.M.). (Cochabamba: Historia Boliviana,1985).

Engels, F. *Der Ursprung der Familie, des Privateigenthums und des Staates, im Anschluss an Lewis H. Morgan´s Forschungen*. (Hottingen-Zürich*:* Verlag der Schweizerischen Volksbuchhandlung, 1884).

Erickson, C. L. Lomas de ocupación en los Llanos de Moxos, in *Arqueología de las tierras bajas*, (eds. Durán Coirolo, A. & Bracco Boksar, R.) 207-226 (Montevideo, Uruguay: Comisión Nacional de Arqueología, 2000).

Erickson, C. L. The Domesticated Landscape of the Bolivian Amazon, in *Time and Complexity in Historical Ecology*, (ed. Balée, W. & Erickson, C.L.) 235-278 (New York: Columbia University Press, 2006).

Fletcher, R., White, K. & Penny, D. Risk and low-density dispersed urbanism. *Front. Hum. Dyn.* **6**:1347157 (2024).

Gauthier, N., Picard, J.C., Duval, N. *et al.* (eds) *Topographie Chrétienne des cités de la Gaule, des origines au milieu du VIIIe siècle.* 16 vols. (Paris: Impr. de Boccard, 1986-2014).

Hansen, M. H. *Polis: An Introduction to the Ancient Greek City-State*. (Oxford: Oxford University Press, 2006).

Heckenberger, M. J. *et al.* Amazonia 1492: Pristine Forest or Cultural Parkland? *Science* **301**: 1710-1713 (2003).

Heckenberger, M.J. *et al*. Pre-Columbian Urbanism, Anthropogenic Landscapes, and the Future of the Amazon. *Science* **321**, 1214-1217 (2008).

Hirst J., Singarayer J., Lombardo U. & Mayle F. Constraining the population size estimates of the pre-Columbian Casarabe Culture of Amazonian Bolivia. *PLoS One* **20**(5): e0325104 (2025). https://doi.org/10.1371/journal.pone.0325104

Iriarte, J., de Souza, J.G., Robinson, M.E., Barbosa, A.D. & da Silva, F. Refining the Chronology and Occupation Dynamics of the Mound Villages of South-eastern Acre, Brazil. *Amazônica, Revista de Antropologia* **13**, 153-176 (2021).

Labre, A. R. P., *Rio Purús: Noticia*. (Maranhão: Imp. M. F. V. Pires, 1872).

Lathrap, D.W., *Yarinacocha: Stratigraphic Excavations in the Peruvian Montana*. Doctoral dissertation, Harvard Graduate School of Arts and Sciences, 1962.

Lombardo, U. *et.al.* Maize monoculture supported pre-Columbian urbanism in southwestern Amazonia. *Nature*, online 29.1.2025. https://doi.org/10.1038/s41586-024-08473-y3

Lubbock, J. 1865. *Pre-Historic Times as Illustrated by Ancient Remains and the Manners and Customs of Modern Savages.* (London: William & Norgate, 1865).

Lucero, L.J., Fletcher, R. & Coningham, R. From 'collapse' to urban diaspora: the transformation of low-density, dispersed agrarian urbanism. *Antiquity* **89**, 1139–1154 (2015).

Lucretius Carus T.. *Titus Lucretius Carus, his six books of Epicurean philosophy*, (trad. T. Creech). Fourth edition. (London: T. Braddyll, [ca. 55 a.C.] 1699).

Meggers, B. J. Prehistoric Population Density in the Amazon Basin, in *Disease and Demography in the Americas* (eds. Verano, J.W & Ubelaker, D.H.) 197-205 (Washington D. C.: Smithsonian Institution Press, 1992).

Mercier, V. Diario de una Expedición del Madre de Dios al Acre. La Paz: Imprenta La Tribuna 1894. *Colección de folletos bolivianos de Hoy* **3**, 1-16, Facsimilar (La Paz, Octubre 1981).

Métraux, A. Ethnography of the Chaco, in *Handbook of South American Indians*, Vol. I, (ed. Steward, J.) 197-370 (Washington D.C.: Smithsonian Institution, 1946).

Morgan, L. H. *Ancient Society*. Second edition (ed. Montagu, A.). Classics of Anthropology (Tucson: University of Arizona Press, [1877-1878] 1985).

Morris, C. The infrastructure of Inka control in the Peruvian central highlands, in *The Inca and Aztec States, 1400–1800: Anthropology and History* (eds. Collier, G.A., Rosaldo, R.I. & Wirth, J. D.) 153-171 (New York: Academic Press, 1982).

Neves, E. G. & Petersen, J.B., Political Economy and Pre-Columbian Landscape, Transformations in Central Amazonia, in *Time and Complexity in Historical Ecology*, (eds. Balée, W. & Erickson, C.L.) 279-309 (New York: Columbia University Press, 2006).

Pärssinen, M. *Tawantinsuyu. The Inca State and Its Political Organization*. (Helsinki: Societas Historica Finlandiae, 1992).

Pärssinen, M. Collasuyu of the Inka State, in *The Inka Empire: A multidisciplinary Approach* (ed. Shimada, I) 265-285 (Austin: University of Texas Press, 2015).

Pärssinen, M. Tequinho Geoglyph Site and Early Polychrome Horizon 300 BC – AD 300/500 in the Brazilian State of Acre, *Amazônica: Revista de Antropologia* **13**, 177-220 (2021a).

Pärssinen, M. Aquiry: um breve retrato de uma civilização amazónica, in *Amazônia: Os Geoglifos e a Civilização Aquiry* (eds. Ranzi, A. & Pärssinen, M.) 19-64 (Florianópolis, Helsinki, Madrid: Officio & Fundação Ibero-Americana da Finlândia/Instituto Ibero-Americano da Finlândia, 2021b).

Pärssinen, M & Ranzi, A. Mobilidade cerimonial e a emergência do poder político com as primeiras estradas conhecidas do oeste amazônico (2000 A.P.), in *(I)Mobilidades na Pré-história: Pessoas, recursos, objetos, sítios e territórios* (eds. Vilaça, R. & R. Simas de Aguiar, R.) 307-349 (Coimbra: Coimbra University Press, 2020).

Pärssinen, M., Kalliola, R. & Ranzi, A. Zanjas circundantes en el extremo norte de Bolivia (Pando y Beni) y sus relaciones con los llamados geoglifos de Acre. *Arqueoantropológicas* **9**, 15-49 (2024).

Pärssinen, M., Kesseli, R.U. & Faldín, J. Paria, the southern Inka capital rediscovered. *Chungara: Revista de Antropología Chilena*, **42**, 235-246 (2010).

Pärssinen, M., Schaan, D. & Ranzi, A. Pre-Columbian geometric earthworks in the upper Purús: a complex society in western Amazonia. *Antiquity* **83**, 1084-1095 (2009).

Pärssinen, M., Ferreira, E., Virtanen, P.K. & Ranzi, A. Domestication in motion: Macrofossils of pre-colonial Brazilian nuts, palms and other Amazonian planted tree species found in the Upper Purus. *Environ. Archaeol.* **26**, 309-322 (2021).

Peripato, V. *et al*. More than 10,000 pre-Columbian earthworks are still hidden throughout Amazonia. *Science* **382**, 103-109 (2023).

Prümers, H., Betancourt, C.J., Iriarte, J., Robinson, M. & Schaich, M. Lidar reveals pre-Hispanic low-density urbanism in the Bolivian Amazon. *Nature* **606**: 325-358 (2022).

Roosevelt, A. C. *Moundbuilders of the Amazon: Geophysical Archaeology on the Marajo Island, Brazil*. (San Diego: Academic Press, 1991).

Roosevelt, A. C. The Amazon and the Anthropocene: 13,000 years of human influence in a tropical rainforest. *Anthropocene* **4**: 69-87 (2013).

Roosevelt, A. C., Housley, R.A., Imazio da Silveira, M, Maranca, R. & Johnson, S. Eighth Millennium Pottery from a Prehistoric Shell Midden in the Brazilian Amazon. *Science* **254**, 1621-1624 (1991).

Rostain, S. *et al.* Two thousand years of garden urbanism in the Upper Amazon, *Science* **383**, 183-189 (2024).

Saunaluoma, S. Pre-Columbian Earthworks in the Riberalta Region of the Bolivian Amazon. *Amazônica, Revista de Antropologia* **2**, 86-111 (2010).

Spengler, O. *The Decline of the West.* (New York & Oxford: Oxford University Press, [1918-1922] 1991).

Spinney, L. How warfare drove the rise of civilizations. *Nature* **645**, 845-847 (2025).

Steward, J. H. *Theory of Culture Change: The Methodology of Multilinear Evolution.* (Urbana: University of Illinois Press, 1955).

Steward, J. H. & Faron, L. C. *Native Peoples of South America*. (New York: MacGraw-Hill Book Company Inc, 1959).

Steward, J. H. & Métraux, A. Tribes of the Peruvian and Ecuadorian Montaña, in *Handbook of South American Indians*, Vol. 3, (ed. Steward, J.H.) 535-656 (Washington D. C.: Smithsonian Institution, 1948).

Thomsen, C. J. *Guide to Northern Archæology by the Royal Society of Northern Antiquaries of Copenhagen*, (ed. Earl of Ellesmere), (London: James Bain, Haymarket, 1848).

Toynbee, A. *A Study of History,* Vols. I-XII. (Oxford: Oxford University Press, 1934-1961).

Tylor, E. B. *Primitive Culture: Researches into the Development of Mythology, Philosophy, Religion, Art, and Custom*, Vols. I-II (London: John Murray, 1871).

Virtanen P.K. Urbanity in Ancient and Present-Day Southwestern Amazonia. Human-Environment Collectives, Cycles of Generosity, and Their Ruptures, in *Urban Imaginaries in Native Amazonia: Tales of Alterity, Power, and Defiance* (eds. Santos-Granero, F. & Fabiano, E.) 205-232 (Tucson: The University of Arizona Press, 2023).

Virtanen, P. K. & Saunaluoma, S. Variable Models for Social Organization of Monumental Earthworks in Upper Purus, Southwestern Amazonia: Archaeological and Ethnographic Perspectives. *Tipiti: Journal of the Society for the Anthropology of Lowland South America* **13**, 23-43 (2015).

Virtanen, P.K. & Saunaluoma, S. Visualization and movement as configurations of human–nonhuman engagements: precolonial geometric earthwork landscapes of the Upper Purus, Brazil. *Am. Anthropol.* **119**, 614-630 (2017).

Point-by-point discussion 19.4.2026 (for manuscript 2025-04-08530C)

Code: 2025-04-08530D

We would like to thank our referees again for taking the time to review our manuscript **"Over 20,000 pre-colonial earthworks in the Southwest Amazonia."** Our point-by-point response to the reviewers' new comments and concerns is below. The referees' comments are in red and our responses are in black.

Referee #2

The authors have been responsive to most of the reviewers' criticisms, and I don't see any compelling additional criticisms, in the current reviews, relevant to their imaging and measurement methods and consequent conclusions about the numbers of prehistoric earthworks and the implications. So, their findings from that research don't seem controversial in essence, and they are novel, showing that many more mounds were built and more densely placed during the particular prehistoric period than was expected. That even the terra firma of Amazonia incurred dense human occupations during the last few thousand years of prehistory does fit with much of the recent archaeological data on earthworks and habitation mounds elsewhere in Amazonia and it also fits with the ethnohistoric evidence for existence of larger population sizes in contact-period settlements and ceremonial centers than there are in current indigenous settlements.

…

As for reviewer 3's criticism that the authors use too many different ways to assess what the earthworks might represent in settlement population sizes and densities, that criticism seems to conflict with the first criticism. In any case, to me, it's useful to compare different ways of estimating population for a prehistoric culture. Amazonian cultures, the shape and size of their settlements, and their propensity or not of building earthworks make them extraordinarily varied. Thus, no one choice of ancient, historic, or modern culture could necessarily be the perfect model for any ancient one. But Iriarte's maps and estimates of later mounds and settlements in the area, combined with the ethnohistoric and ethnographic testimonies he references, do, as the authors point out, make clear that by then houses were malocas, which are extended family houses, not nuclear ones. Malocas, as I mention in my reviewer comments on earlier versions of the manuscript, are the most common type of family residence documented in Amazonia before intensive missionization. (MIssionaries did not like extended family living, especially not when the family was matrilineal.) And, as the authors show, the different ways of reconstructing population for the Aquiry tend to produce comparable results. In any case, the authors give a wide range of population in their estimates, not a

single estimate. (By the way, no nuclear family house I've observed in Amazonia was as long as 25 meters; that size would be an extended family house, a maloca.)

Reviewer 3's questions about the chronology of the building and use of the Geoplyph earthworks seem unrealistically persnickety, given the authors' very detailed discussion, analysis, and illustration of the radiocarbon dating and the mapping project. Furthermore, I found the current manuscript's descriptions of methodology in the supplements very clear and detailed.

…

This reviewer 5 also takes issue with the authors' use of the term "civilization" for the prehistoric Anquiry culture, and suggests that they should use the term cultural tradition instead. But nowadays in Amazonian studies most researchers use the term civilization for cultures that have significant cultural complexity in some form, whether monumental constructions, whether they are ceremonial, residential, or agricultural, archaeoastronomical and/or monumental rock art, monumental sculptures and paintings, elaborate crafts and fine art, large, organized settlements with specialized centers, an extensive geographic sphere, or some combination of these characteristics. Thus, the elaborate, monumental, carefully planned, standardized, and geometrically precise earthwork constructions of the widespread Aquiry Geoglyph culture would qualify it to be called a civilization.

In any case, in Greater Amazonia several expansive mound-building cultures developed that are uncontroversially classified by anthropologists as highly complex societies and often referred to as civilizations: the Marajoara culture, the Santarem culture, the Upano society, the Llanos de Mojos mound culture, and the Geoglyph culture. All but Santarem were long-lasting regional cultures, and the Marajoara and Santarem cultures were also "Horizon Styles", called thus because their art styles and settlement patterns rapidly spread through much of Amazonia from their regions of origin. The settlement patterns and earthwork constructions, food choices, agricultural methods, and hunting and fishing methods vary a lot from culture to culture, but all had earthworks, diverse, highly decorated, elaborate, and abundant art and craft production. And all are considered "urban" in settlement pattern either because they had a large nucleated ceremonial-residential center with dispersed towns and villages, like Santarem (at least 4 square km in area, or because they had non-residential ceremonial centers with dispersed residential settlements like the Geoglyph cultures. Or in the case of Marajoara, because they had large and sometimes highly clustered multi-maloca mound residential-ceremonial settlements as well as numerous multi-maloca residential-ceremonia settlements on small mounds.

We are grateful to Referee #2 for these and many other detailed points in her review, with which we are in complete agreement.

Referee #3

They state that 300 people can build a geoglyph of 8,000 cubic meters in 100 days. They also write: “We clearly state that this 'independent' chronological activity model yields a figure of 4,360 earthworks per 200 years, equating to an average of 22 geoglyphs constructed per year between 100 and 300 AD. According to our various estimates, this would require an average community of 300 people, multiplied by 4,360, equating to 1.3 million people.”

This final sentence is where the main disagreement arises. If 300 people build one geoglyph in 100 days, they can build 3.65 geoglyphs per year, or 730 geoglyphs in 200 years. If 4,360 geoglyphs were built in that period, the implied population would be 300 × (4,360 / 730) = 1,792 people, not 1.3 million.

We respectfully note that Referee 3 may not have been fully familiar with the key and relevant Amazonian historical and archaeological literature, as he/she seems to have missed several foundational points addressed in the manuscript and our point-by-point response.

Firstly, as we noted in the previous point-by-point discussion, no evidence has been found in the Amazon to suggest that groups of a few hundred people travelled across a vast area for around 1,400 years to construct geoglyphs. There is also no evidence of state or other administrative structures that would have dispatched such groups. As mentioned previously (see, Point-by-point discussion 23.1.2026, pp. 10), the wide range of structural features that we analysed in the study area (such as sizes, shapes, precision, placement and roads) does not refer to uniform technical plans or the role of common construction designers or teams moving around the area over time (Kalliola et al. 2024, 2026). At least some evidence is required to substantiate such a claim before serious speculation on the matter can begin. Instead, there is abundant historical and ethnographic evidence — some of which we have referred to in the 50 permitted references — that, alongside other duties, regional communities specifically built and maintained such ceremonial and administratively significant centres, to which people from neighbouring areas were also invited. These communities constructed the geoglyphs, used, and maintained them for various centuries, as demonstrated by

multiple excavations and over a hundred test pits. We cannot accept the complete rejection of positive or negative evidence in our analysis.

Secondly, it has been demonstrated that the geoglyphs were constructed in relatively small forest clearings, each measuring normally between half a km² and a few square kilometres (Watling et al., 2015; Pärssinen et al., 2020). Therefore, the area of the Aquiry was not an open savannah. Instead, the forest area first had to be cleared of trees, stumps, and roots to create an open space. Thus, researchers studying the area seem to agree that constructing a geoglyph at a given site is physically possible only once every 27–28 years, when the bamboo flowers and dies, allowing it to be eradicated by fire. This is supported by the fact that hard stone material is unavailable in the area and stone axes found in archaeological discoveries are relatively rare imported items. While the bamboo is alive, it is practically impossible to clear the forest because cutting the stems is dangerous. Furthermore, digging through thick clay soil filled with bamboo rhizomes using stone and/or wooden tools would be extremely difficult. Considering that mass die-offs of bamboo-dominated forests typically cover areas of several hundred, and sometimes thousands, of square kilometres (Carvalho et al., 2013) the same community will not have the opportunity to build a new geoglyph or maize and cassava fields for 27–28 years (see Point-by-Point Discussion, Nature Manuscript 2025-04-08530, p. 22). Over a period of two hundred years, the theoretical possibility arose eight times, but radiocarbon dating suggests that geoglyphs were relatively rarely built close to one another. Even then they were often connected by a road, suggesting simultaneous use, growth in community significance, and population size. As we explained in our earlier point-by-point discussion (21.1.2026, pp. 9–10), a community of 300 people can construct an average geoglyph during the year (following the bamboo die-off), even when accounting for the time taken for forest clearing (100 + 100 days) and other necessary tasks. Furthermore, if we assume that a person can dig 0.5 m³ of earth per day, as Hirst et al. (2025, p. 10) calculated, rather than 1 m³ as we calculated, then a somewhat larger population would have been required, while the task would probably have been impossible with fewer people.

Thirdly, the dry season in the region lasts from May to October. In practice, forest burning can be carried out for three months, from mid-July to mid-October. Clearing the forest and removing stumps and roots is there as labour-intensive and energy-consuming as digging the geoglyphs and cannot be done at any time. If a community of

300 people wanted to build two geoglyphs alongside all the other necessary work in the same year, it would be extremely challenging. If two geoglyphs were sometimes constructed, it is likely that the community's population was twice as large at that time. The idea that the same community of 300 people would start building three or four average-sized geoglyphs in one year, and maintaining these paces year after year in the context of the Western Amazonia, is extremely unlikely.

This number could be increased based on other lines of evidence, such as habitation sites, labor required for maintenance, demographic modeling, and estimates of carrying capacity. However, even accounting for all of these factors, it is not possible to reach a population estimate of 1.3 million.

As Referee 3 is well aware, the Aquiry's *habitation sites* have not yet been identified, partly due to restrictions on excavation permits. Why refer to these kinds of impossibilities? Nevertheless, we have already studied mound villages that appeared in the same locations after the geoglyphs were abandoned. These confirm our calculations that the average community size was around 300 people (lines 287–316). As there are no signs of radical population replacement, this evidence is important.

With regard to demographic modelling, we have already referenced the ethnographic evidence and the linear regression modelling of De Souza et al. (2018). They calculated that there were between 500 and 600 people per earthwork. They consequently calculated that 1,000–1,500 earthworks corresponded to 0.5–1 million inhabitants in the Southern Rim of the Amazon region. This calculation can be considered as one possibility. However, we have suggested that the number of people per earthwork was 45% lower, based on multiple pieces of evidence in the Aquiry area. If De Souza et al. (2018) had argued that the earthworks were constructed by only 600 people, corresponding to the entire population size, we are quite sure the article would never have been allowed to be published.

Furthermore, earlier we demonstrated that mixed agricultural and agroforestry practices in the Aquiry area, containing both *terra firme* and fluvial *varzea* zones, have the potential to support a population up to three million or even more (see Point-by-point discussion, Nature manuscript 2025-04-08530, p. 20-21). In the main text, we mention that the Aquiry area is nutrient-rich, based on its cation concentration. Additionally, a substantial part of the area has eutrophic haplic cambisol and luvisol (Carvalho et al. 2013, Fig. 6), which are generally considered good for agriculture

(https://www.fao.org/4/y1899e/y1899e08.htm). We would also like to refer to an earlier comment by Referee 2 in this respect: “*As an archaeologist and environmental anthropologist, I do not find the authors' population estimates "shocking", but as I mention in my review, they seem consistent with those I made based on the sites I have surveyed and excavated in other parts of Greater Amazonia*.” We can also make a reference here to the most recent population estimate of the Classic Maya in Mesoamerica (https://doi.org/10.1016/j.jasrep.2025.105288). These findings calculated 9.5-16 million people in an area of 95,000 square km, which is up to 10 times the population in a comparable area to the Aquiry. In both areas, maize and other agricultural and agroforestry products were used, and there was arguably no obvious difference in soil fertility.

They cite Hirst et al. (2025). In that paper, Hirst et al. estimate the average population of the Casarabe Culture, based on the energetics of constructing all known earthworks, to be between 9,000 and 60,000 inhabitants, accounting for the movement of 50 million cubic meters of earth over 1,000 years. In contrast, the Aquiry earthworks involved the movement of 8,000 × 4,360 = 35 million cubic meters in 200 years. Taking the upper limit of the Hirst et al. estimate, the maximum population of the Aquiry would be 60,000 × 5 × (35 / 50) = 210,000, which is still far below 1.3 million.

We cite Hirst et al. (2025) (lines 377–378) only to refer to the concept of 'low-density urbanism', not to conduct a systematic comparison. There are various reasons for this in such a short paper. Most of these relate to Galton's problem, i.e. whether the units in question are comparable. Nevertheless, when Referee 3 makes his/her own comparison between our results and those of Hirst et al., he/she disregards the basic rules of comparison, revealing his/her intention to manipulate the results of our debate.

Firstly, Referee 3 fails to consider that Casarabe is located in a natural savannah, whereas Aquiry is located in a dense tropical forest. The undertaking of a mechanical 1:1 comparison between these two different environments is, as mentioned earlier by Referee 2 in other circumstances, "*rather unfair*."

Secondly, when discussing 'the energetics of constructing all known earthworks', Referee 3 fails to consider that half of the energy in the Aquiry area is spent clearing the forest before earthworks can be constructed.

Thirdly, he/she does not take into account the 27–28-year cycle of the bamboo-dominated forest in the Aquiry area mentioned in our first point-by-point discussion, as well as various articles, starting with Carvalho et al. (2013) and McMichael et al. (2014).

Fourthly, Hirst et al. mostly estimated residential mounds, whereas we estimated ceremonial centres. As we noted earlier (lines 296–297), comparisons of this kind are problematic due to the different functions of the sites, so substantial differences may be expected for that reason alone.

Fifthly, Hirst et al. included causeways and the numerous canals associated with agricultural fields in their analysis. However, Referee 3 ignores the hundreds of kilometres of roads in the Aquiry area, along with their embankments and the clearing of trees and other vegetation in the agricultural fields. Therefore, his/her units of comparison are not directly comparable 1:1.

Taking into account the double workload resulting from forest clearing for geoglyphs, roads, and fields, as well as the multiple workloads required to keep these areas clean, changes the formula 60,000 x 5 x (35/50) to about 60,000 x 5 x (120/50), which equals 720,000 people. However, considering that Hirst et al. effectively raise the upper population limit to 100,000 gives a result of 100,000 x 5 x (120/50), which equals 1.2 million. This is almost equal to the lower population limit of 1.3 million obtained from the KDE model, despite the difference in units of comparison. Furthermore, Hirst et al.'s calculation appears to represent a 1,000-year average. In our article, however, we demonstrate that population density has varied significantly over time, so our focus is on the maximum peak period. The long-term average is not directly comparable: in the case of Aquiry, the mean value would actually be around 40% lower, at approximately 747,000 inhabitants, if low-activity periods during around 1,400 years of the entire Aquiry period were also included. Therefore, if we unify our units to make them more comparable, the results would actually be quite similar – as such, Referee 3´s concerns are unfounded.

If we instead apply Pärssinen et al.’s calculation to the Casarabe culture, the resulting population would be 50,000,000 / 8,000 × 300 = 1,875,000 people within an area of

5,000 km², corresponding to a population density of 375 people per km². This is between 20 and 200 times higher than the estimates reported by Hirst et al.

Regarding this second “comparative” exercise, which gives a population of 1,875,000 for Casarabe, we would like to point out the following:

Firstly, since this calculation only considers the volume of earth moved for a single ceremonial centre, it is advisable to exclude the roads and fields from the Hirst et al. (2025: 10) study as well. They estimate that 36,000,000 m³ of earth was moved in constructing the mounds. In turn, we calculated that 8,000 m³ of earth was moved to construct an average geoglyph. In addition, the same amount of labour was used for forest clearing beforehand, resulting in a proportional figure of 16,000 cubic metres of earth and wood being moved. This gives us the formula: 36,000,000 ÷ 16,000 × 300 = 675,000 inhabitants. However, Referee 3 has 'forgotten' that this is a cumulative population figure spanning 1,000 years. Therefore, if the 27–28-year bamboo die-off effect were not counted, the same technical factor of 5, at least, should be applied to this calculation, as was applied earlier when it was compared to Aquiry's 200-year period. When this adjustment is made to the formula, we obtain 675,000 ÷ 5 = 135,000 inhabitants, rather than the 1,875,000 people claimed for the Casarabe. Therefore, the claim that our calculation method would lead to estimates 20–200 times higher than those reported by Hirst et al. (2025) is also unfounded.

Referee #5

The current logical issue in archaeology is that there is no agreed or correct definition of either civilisation or urbanism. As is emphasised in Fletcher (RJF) 2020 in the Journal of Urban Archaeology there are numerous regional definitions of urbanism and no group of regional specialists will concede to any other NOR is there an agreed global definition. Nor does RJF argue for one. RJF argues for the designation of a universal “frame of reference” which would be quantifiable to allow global cross-comparison and has proposed such a frame of reference on which White (KW) has been working.

OK. We agree that there are many definitions of civilization, urbanism, and even culture, and that there is no universally agreed meaning of these concepts. Even when used in a regional context, probably no definition is perfect.

Our specific emphasis is that currently there is a serious logical problem created by regional specialists claiming their own definitions AND concurrently wishing to refer to usage elsewhere to claim a global legitimacy derived from the cachet associated with the terms “urban”.

In the South American context, different types of urban appearance have been recognised for a long time. For example, as Referee 2 writes,”*As John Rowe [1963] pointed out early in the history of anthropological research in South America, ceremonial centres in the Andes can exist separately from residential settlements. Ancient complex cultures there vary in terms of whether people live in such centres, or if the centres are empty when not being used for ceremonies. The resident populations are dispersed around them*.” Furthermore, the term 'low-density urbanism' may have first used in the Amazonian context in the Xingu region by Heckenberger et al. (2008), who referenced E. Howard's *'Garden Cities of Tomorrow'* (1902). Since then, the concept has been widely adopted, as we have previously mentioned. Based on these research traditions, we are referring to ”*discussion about several types of urbanism*” (pp. 47-8) – not any hypothetical universal form of urbanism. As previously mentioned in our earlier point-by-point discussion, Lucero, Fletcher & Coningham (2015) also refer to the smaller communities of the Postclassic Maya culture as ”*a different kind of urbanism*.” In the case of the Aquiry, we prefer to use the concept of *'forest urbanism'* (pp. 377–8), but we also make one a reference for the concept of 'low-density urbanism' as used by Heckenberger et al. (2008) and Hirst et al. (2025). However, to clarify the multiple meanings of the final concept, we will now also add Fletcher (2020) to the list.

The same applies to “civilisation” – there is no agreed definition or usage of the term. The French refer to Palaeolithic cultures as “civilisation” but that is not the conventional English usage. The term civilisation has also rather gone out of fashion in scholarly commentary and is primarily used only as a standard label eg Roman of Chinese civilisation. We do not argue that there is a proper definition of civilisation because there isn’t one. The usage in the paper of a regionally specific term and then claiming association to world-wide examples which purports reference to a global term that actually has no agreed definition is logically inconsistent.

Comparisons can also be used to clarify the narrative by referring to similar or different phenomena in other cultures or civilizations. However, it is important to remember that the purpose of our article is not to undertake a systematic comparison of world civilizations, but rather to describe Aquiry as a multicultural entity, particularly within

the South American context. This entity shared a common cosmovision that was reflected in the construction of monumental, ditched, geometric ceremonial centres, in the same way that mosques reflect the material heritage of Islamic civilization and churches reflect that of Christian civilization. For clarity, we will add a reference to a work that has addressed the topic in greater depth in the Supplementary section. It is also worth noting that the concept of civilizations in the Amazonian context is not new. As early as the 18th century, communities that constructed earthen structures with ditches in the Amazon region were referred to as civilization (Eder, 1772, 1985, 84; see also Ranzi & Pärssinen, 2021, 25). Furthermore, as Referee 2 writes, "*In Greater Amazonia, several expansive mound-building cultures developed that are uncontroversially classified by anthropologists as highly complex societies and often referred to as civilizations*."

Sentences 110-111 are problematic and illustrate the issue. Among the list of words only "administration" is a term among Western criteria used to distinguish towns from villages. Universality has been invoked but all the other labels "sociocultural and cosmological motivations. Including diverse forms of art, dance, music and sport" are characteristics of immensely varied societies including the Australian Aborigines (The First Nations of Australia), the North American Plains Indians and numerous other societies. If all such societies are to be labelled as possessing towns then the significance of the category disappears. We are not advocating for a specific meaning of the label town, only emphasising that the effort to use a generality ascribed to another regional case from elsewhere to validate a particular regional designation - which the authors advocate - while concurrently claiming the uniqueness of their regional designation is not logical. To illustrate further – do the bora rings or the huge, hectare-sized eel traps build by First Nations Australians, or the inukshuk stone trap-lines several kilometres long constructed by the Inuit, or the construction of the massive earthen cursus structures near Stonehenge display the presence of "administration"? Or has "administration" been stretched to mean human collective action, without any trace of the management accoutrements familiarly associated with administration? The paragraph is problematic.

It seems there has been some kind of misunderstanding here. Originally, we were referring to the classic text on urban studies by V. Gordon Childe (1950) and Virtanen's further (2023) analysis of it in relation to Amazonian socio-cosmologies. Specifically, we were referring to the sections on monumental architecture, administration, division of labour, mathematics, geometry and astronomy. When we had to limit the number of

citations, Childe was omitted, but Virtanen was kept because she was also cited elsewhere. However, it seems the reviewer did not read it. As the reference to *early* Western criteria (lines 111-113) is not essential and could lead to misinterpretation, it can be removed. Certain forms of art, dance, music and sport are prevalent in many societies.

In reference to the population estimates the procedures remain problematic. First, the reference to the later mound sites is irrelevant to the calculation derived from the reasonable logistical estimate of the number of people who would be needed to construct a geoglyph.

We disagree. The reference to the later mound sites provides strong additional evidence. As we explained earlier, there is clear continuity between these two different archaeological phases, including in agroforestry and agriculture, and no signs of radical population replacement (see also Iriarte et al., 2021).The number of individual mounds surrounding a plaza, representing multi-family *maloca* dwellings, can be easily calculated using satellite imagery. These villages may offer the best indication of the size of the dispersed communities that chose to live together somewhat later in the core area of the ancient Aquiry.

Secondly, the issue remains that no actual demonstration has been provided of the contiguity of the use of the sites except over many decades and that removes the one-to-one singular connection between a builder population and one site. As the authors have shown elsewhere in their work the charcoal in the sites relates to episodic burning of the local vegetation. The sites are traps for that material. The Bayesian analyses do indeed show the increasing and then the decreasing amount of that burning over time.

The fact is that over a hundred test pits dug within the geoglyphs indisputably show that pieces of charcoal, which were deposited before construction began, are more densely concentrated and generally larger than the tiny, random fragments of charcoal found in slightly ash-rich soil dating from the time of construction and use of the earthworks. The layer associated with the use of the geoglyphs is typically 20–45 cm thick (Schaan et al., 2012; Pärssinen et al., 2024). In exceptional cases, the layer can be up to 70 cm thick. It usually shows signs of consistent cleaning and typically contains less gravel than the other layers. Burnt branches of bigger than 0.5 cm are extremely rare, and

typical fire debris are absent. Pottery shards are also rare in this layer. They are found in slightly greater numbers in the ditches. A distinct fire layer has only been found in the upper level of the geoglyph at the Fazenda Colorado site (Schaan et al., 2012). At the Tequinho site, however, several fire layers have been found in the mounds at the junction of the main road and the geoglyph. One of these contained tens of thousands of fragments of high-quality serving vessels and ceremonial ceramics (Pärssinen 2021; Pärssinen et al., 2021). Where excavations have revealed two distinct layers relating to the use of a geoglyph, the period of use is typically over 500 years (e.g. at the Tequinho, Fazenda Colorada and Severino Calazans sites). This suggests that the geoglyph sites were used intensively and cleaned multiple times a year. Additionally, radiocarbon dating and stratigraphic thicknesses suggest that the sites were normally used for at least two hundred years. Clear burning episodes are only observed in the deeper pre-geoglyph phases and the superficial layer of the post-geoglyph phase.

Furthermore, several excavations have shown that in some geoglyphs the ditches gradually filled in, but in other cases the ditches were also cleared over a period of several hundred years. For example, a piece of charcoal (Ua-72293) was found at a depth of approximately 3.5 metres at the bottom of the Tequinho geoglyph ditch. This charcoal dates to the geoglyph's early phase, around 200 BC–AD 5, but excavations revealed that the ditch was not filled in rapidly until the years AD 420–650. After this period, the site was apparently abandoned, as no older dates were obtained (Pärssinen et al., 2021; Supplementary Table 1a). As there is very little charcoal at these sites, it is unclear what Referee 5 means by "the charcoal in the sites relates to episodic burning of the local vegetation. The sites are traps for that material". In contrast, when we excavated test pits outside the Severino Calazans site in an area that we interpreted as an ancient field, we found moderate quantities of charcoal fragments in three different stratigraphic layers. There were no signs of cleaning (see Supplementary Table 1a).

The ceramics in the sites do indeed show that people are periodically passing through the sites. But neither shows that the population at any one time equalled the total population required to provide different labour populations to build each site that occur within a C14 error range of about 30 years. Because, if one group of people is constructing a new site every few years - say every 6 years - then the population required to construct a set of sites within 30 years is 1/5 of the estimate presumed from

claiming that the labour required is unique to each site and is cumulative and contemporaneous.

With all due respect to Referee 5, as was explained to Reviewer 3, researchers in the field agree that "*that constructing a geoglyph at a given site is physically possible only once every 27–28 years, when the bamboo flowers and dies, allowing it to be eradicated by fire. This is supported by the fact that hard stone material is unavailable in the area and stone axes found in archaeological discoveries are relatively rare imported items*."

Furthermore, there is no archaeological, historical or ethnographic evidence to suggest that the builders of the monumental geoglyphs travelled across a wide area to construct multiple geoglyphs. There is also no discernible technical consistency or precision in the earthworks, nor any evidence of repeated interruptions to cleaning activities or multiple forest fires in the stratigraphy of any site associated with the use of geoglyphs. We would also like to highlight the significant effort required for clearing and constructing even a single monumental site, not to mention the continuous removal of vegetation under the conditions of the Amazon's rapidly growing rainforest. Thus, constructing a geoglyph every six or even every 20 years was not an option.

What is assumed but not empirically demonstrated is that each site remains continuously in use all through the relevant period and that the ceramic debris represents the continual rather than the episodic presence of people. If, by contrast, this is the signature of a seasonal and annual throughput population that is episodically using many sites over a given period of time then the total population was much smaller than the claimed aggregate in the paper. The issue is not how many sites are contemporaneous, ie exist, across a given period of time but the degree to which they are each being used in a given time span, eg a year, and how many sites are being used periodically, either by one group of builders or by episodic aggregations of otherwise very small groups of people to build one. It is a fallacious assumption inherent to property-owning societies that everyone who builds a structure then stays nearby thereafter and is associated with a particular structure. Numerous societies world-wide have not done so.

There are numerous historical and ethnographic accounts describing the types of ceremonies held at monumental ceremonial centres, such as geoglyphs, and how the communities that built them maintained them. We and others have discussed these in numerous articles. The communities living in the vicinity of these sites maintained them continuously; otherwise, they would quickly have become unusable. Participants from

elsewhere were also invited to important events, such as competitions. The host appears to have almost always been the community leader. Mobility and reciprocity were natural for these communities. However, as emphasised previously, it is highly improbable that the same group would have constructed geoglyphs in various locations every six years. With all due respect, reference to ”*numerous societies world-wide*” is not proof. Instead, the well-documented cycle of bamboo-dominated forests in the area and the more than hundred archaeological test pits are. Even if part of the population had moved during the 27–28-year bamboo cycle, new people would have arrived from communities with which they interacted. Furthermore, it is likely that the leaders of these communities had an interest in keeping their communities tied to the centres they led within their territories.

In our view, we have demonstrated that very small groups would not have been able to clear space for open areas spanning several hectares and to construct and maintain relatively large, monumental geoglyphs over the course of centuries, as archaeological excavations have shown. They simply would not have had enough time to burn the forest, clear the roots, and construct deep geoglyphs in two or more locations during this brief period either. If two geoglyphs were constructed, then twice the population would have been needed (relative to the area to be built). Furthermore, according to oral tradition, the division of labor among many communities was such that some were responsible for burning and clearing, while others were responsible for digging (Point-by-point 23.1.2026). Similarly, the necessity of road maintenance, crop rotation, and intensive agroforestry tied communities to the area where each ceremonial centre and its fields were located. However, we still know little about the settlements, partly due to the limited availability of research permits. Based on all the above, it seems more likely to us that the population lived primarily in large *maloca* dwellings shared by several families. These dwellings were surrounded by well-maintained areas, and waste was spread out over the fields. Only future research can resolve this question. Finally, what about our (?) assumption inherent to property-owning societies that ”everyone who builds a structure then stays nearby”? This seems to be a bit of a petty claim among colleagues, when the article's authors include an Indigenous peoples' representative and several scholars who have lived among various Indigenous ethnic groups for years. The issue is certainly not one of a Western, property-centric assumption, but rather the evidential value of archaeological excavations, as well as oral and written traditional

knowledge. Our group has long-standing experience with the archaeology of both sedentary communities and highly mobile ones (e.g., Tupi-Guaraní) as well as communities that were intentionally relocated (e.g., the Inca State) (see, e.g., Pärssinen & Korpisaari 2003).

Depending on the degree to which groups are moving through multiple sites and the frequency with which they are building new sites, which they then use episodically over decades, the population estimates for any one period of time would differ substantially and would be much lower than estimates correlating a sum total, cumulative numbers of all builders for all sites within a ballpark period such as 30 years. The paper needs to incorporate how large those differences in estimates of total populations might be, because radically different estimates of food consumption and firing impact on vegetation among others, then follow.

We have addressed these issues in our earlier articles and in this point-by-point discussion. The ballpark period of 30 years corresponds quite well with the 27–28-year life span of the bamboo-dominated forests of the area. This was when the construction of new earthworks was not an option. Furthermore, there are no known archaeological indications of the rapid abandonment of earthworks. However, upon this request, we will provide more information on the above points, particularly in the 'Supplementary' section of our article.

Finally, we wish to emphasise that the recent data on constructed sites coming out of several different regions of the eastern lowlands of South America is profoundly important, including the data on the sites in this paper. The new information is consistent with the characteristics of numerous “anomalous “ sites worldwide in very diverse environments, which demonstrate that conventional terms such as “urban” do not adequately encapsulate the diversity and different trajectories of what are now many hundreds of sites. In addition, the story of the low-density settlement pattern phenomenon over the previous seven millennia is of serious significance for our efforts to understand the prospects of our present day, burgeoning, low-density, conventionally labelled industrial urban landscapes.
Just as recognising a previously unrecognised class of lifeforms is considered to be a profound intellectual contribution so likewise with these cultural phenomena which are being shoehorned into centuries old categories that no longer have a global, coherent definition. As we and our colleagues have pointed out in 2024 in Frontiers in Human Dynamics, there have been at least two earlier, different operational scales of low-density settlements each scale represented by numerous sites. The sites in both of these different spatial scales ceased to operate and neither scale of site developed into an

order of magnitude larger form. The implications for our industrial low-density giant urban settlements are ominous. Note that our industrial low-density cities do not derive from agrarian low-density cities like Angkor, they only derive from smaller compact industrial cities. And the Classic Maya - Angkor scale low density settlements did not derive from sites resembling the oppida, the Trypillia sites and the recently reported Amazonian sites.

If differing types of settlement can all be blandly designated as a generalised category of low-density urbanism, then not recognising profound differences across differing scales of settlement magnitude has major implications is a worrying problem of scholarship.

We fully understand this perspective. If the concept of 'low-density urbanism' is applied to almost everything other than large, traditional, dense population centres, then existing concepts need to be refined and appropriate terms created for these new phenomena. We only mention this term once in passing because it is currently used very commonly in the Amazonian context, and in that context it also fits Aquiry's case to some extent. We also prefer to refer to the concept of 'forest urbanism' in the case of Aquiry, and only time will tell which term will eventually be used to describe it. Terminology, however, is not the main focus of our article.

References

Carvalho A.L. de *et al*. Bamboo-Dominated Forests of the Southwest Amazon: Detection, Spatial Extent, Life Cycle Length and Flowering Waves. *PLoS ONE* **8**(1): e54852 (2013).

Childe, G.V. 1950. The Urban Revolution. *Town Planning Review* **21**, 3-17.

De Souza, J.G. *et al*. Pre-Columbian earth-builders settled along the entire southern rim of the Amazon. *Nature Communications* **9**, 1125 (2018).

Eder, F. J. *Breve descripción de las reducciones de Mojos ca. 1772* (ed. Barnadas, J.M.). (Cochabamba: Historia Boliviana, 1985).

Heckenberger, M.J. *et al*. Pre-Columbian Urbanism, Anthropogenic Landscapes, and the Future of the Amazon. *Science* **321**, 1214-1217 (2008).

Hirst J., Singarayer J., Lombardo U. & Mayle F. Constraining the population size estimates of the pre-Columbian Casarabe Culture of Amazonian Bolivia. *PLoS One* **20**(5): e0325104 (2025). https://doi.org/10.1371/journal.pone.0325104.

Iriarte, J., de Souza, J.G., Robinson, M.E., Barbosa, A.D. & da Silva, F. Refining the Chronology and Occupation Dynamics of the Mound Villages of South-eastern Acre, Brazil. *Amazônica, Revista de Antropologia* **13**, 153-176 (2021).

Kalliola, R., Pärssinen, M., Ranzi, A. & Barbosa, A.D. Ancient Amazonian Earthwork Roads: Unveiling Ceremonial, Livelihood, and Networking Significances. *Lat. Am. Antiq.* (2026). https://doi.org/10.1017/laq.2025.10168.

Kalliola, R., Pärssinen, M., Ranzi, A., Seppä, I. & Barbosa, A.D. Geographies of ancient geometric earthworks and their builders in Western Amazonia. *Acta Amazonica* **54**, e54hu22351 (2024).

Lucero, L.J., Fletcher, R. & Coningham, R. From 'collapse' to urban diaspora: the transformation of low-density, dispersed agrarian urbanism. *Antiquity* **89**, 1139–1154 (2015).

McMichael, C.H., Palace, M.W. & Golightly, M. Bamboo-dominated forests and pre-Columbian earthwork formations in south-western Amazonia. *J. Biogeogr.* **41**, 1733-1745 (2014).

Pärssinen, M. Tequinho Geoglyph Site and Early Polychrome Horizon 300 BC – AD 300/500 in the Brazilian State of Acre, *Amazônica: Revista de Antropologia* **13**, 177-220 (2021).

Pärssinen, M. & Korpisaari, A. (eds.) *Western Amazonia - Amazônia Ocidental. Multidisciplinary Studies on Ancient Expansionistic Movements, Fortifications and Sedentary Life.* (Helsinki: Renvall Institute Publications, University of Helsinki, 2003).

Pärssinen, M, Balée, W., Ranzi, A. & Barbosa, A.D. The geoglyph sites of Acre, Brazil: 10 000-year-old land-use practices and climate change in Amazonia. *Antiquity* ***94***, 1538-1556 (2020).

Pärssinen, M., Ferreira, E., Virtanen, P.K. & Ranzi, A. Domestication in motion: Macrofossils of pre-colonial Brazilian nuts, palms and other Amazonian planted tree species found in the Upper Purus. *Environ. Archaeol.* **26**, 309-322 (2021).

Pärssinen, M., Kalliola, R. & Ranzi, A. Zanjas circundantes en el extremo norte de Bolivia (Pando y Beni) y sus relaciones con los llamados geoglifos de Acre. *Arqueoantropológicas* **9**, 15-49 (2024).

Ranzi, A. & Pärssinen, M. (eds.) *Amazônia: Os Geoglifos e a Civilização Aquiry*. (Florianópolis, Helsinki, Madrid: Officio & Fundação Ibero-Americana da Finlândia/Instituto Ibero-Americano da Finlândia, 2021).

Rowe, J.H. Urban Settlements in Ancient Peru. *Ñawpa Pacha* **1**, 1-27 (1963).

Schaan, D. *et al*. New radiometric dates for precolumbian (2000–700 BP) earthworks in western Amazonia, Brazil. *J. Field Archaeol.* **37**, 132-142 (2012).

Watling, J., Saunaluoma, S., Pärssinen, M. & Schaan, D.P. Subsistence practices among earthwork builders: Phytolith evidence from archaeological sites in the southwest Amazonian interfluves. *J. Archaeol. Sci. Rep.* **4**, 541-551 (2015).

Virtanen P.K. Urbanity in Ancient and Present-Day Southwestern Amazonia. Human-Environment Collectives, Cycles of Generosity, and Their Ruptures, in *Urban Imaginaries in Native Amazonia: Tales of Alterity, Power, and Defiance* (eds. Santos-Granero, F. & Fabiano, E.) 205-232 (Tucson: The University of Arizona Press, 2023).

Point-by-Point discussion_2025-04-08530E_28.5.2026

We would like to thank our referees again for taking the time to review our manuscript "Over 20,000 pre-colonial earthworks in the Southwest Amazonia." Our point-by-point response to the reviewers' new comments and concerns is below. The referees' comments are in red and our responses are in black.

Referee # 2

While we do not repeat Referee 2's comments here, we would like to express our sincere appreciation for her thorough and detailed discussion, which strongly supports the views we previously presented. At the same time, her review offers several new and important perspectives that will help advance the scientific debate.

Referee # 3

By combining the results of the first LIDAR survey with the already known archaeology of the area, they estimate the Aquiry culture to cover 183,000 km². They describe the Aquiry as a "multicultural, complex society" made up of "different social and linguistic groups" that shared a common socio-cosmology and architectural style. I think this needs some elaboration. The main argument for saying that the Aquiry shared a common socio-cosmology is the construction of highly specific geometric architecture, which is basically what they call architectural style. I don't really see that there is anything else than the shape of the earthworks in defining who the Aquiry people were.

With all due respect to Referee 3, we would like to emphasize that we have included the maximum number of references permitted in our article, and we provide a concise overview of previous research on the subject at the beginning of the manuscript. These references are included precisely to allow readers to explore the broader context of the civilization under study. The cited literature demonstrates that the complex we refer to as Aquiry has previously been described as part of the wider Amazonian geoglyph region, as these sites form a coherent spatial unit and differ both chronologically and typologically from other earthworks in the western Amazon. Elsewhere in the region, ditched earthworks are generally of a later date consist primarily of roughly circular

settlements or defensive enclosures with ditches located outside or immediately adjacent to the embankments. In contrast, current evidence indicates that Aquiry earthworks functioned as ceremonial centers, characterized by embankments regularly situated outside their ditches. These centers also typically feature unusually wide entrance openings. By comparison, the settlements known to date—particularly mound villages and circular embanked centers—represent later developments. Furthermore, several studies have examined how specific geometric forms are internally differentiated in relation to identity distinctions (e.g., Kalliola et al. 2024; Saunaluoma and Virtanen 2015). Geometrically shaped more-than-human figures found in the region (e.g., Schaan et al. 2012; Virtanen and Saunaluoma 2017), as well as the connections between ceramics and the Arawak, Pano, and Guarani language groups (e.g., Pärssinen 2021), have also been widely discussed. Similar practices in food production have likewise been analyzed (Watling et al. 2015; Pärssinen et al. 2021).

They exclude the Baures region because the ages are more recent than those from the Aquiry. However, when comparing the Aquiry to the people who built later mound villages in the same core area around the early 13th century, the authors note that there are no signs of radical population replacement because the people living in these later villages "used similar domestic ceramics" (no reference provided by the way) and shared similar subsistence strategies. So, why excluding Baures on the base of the ages? Is it because of the ceramic style?

The savanna area of Baures, located nearly 500 km to the south, with its causeways, raised fields, and quite different ditched enclosures date to a time when the Aquiry had already disappeared. Its pottery was also very different, which we have been able to ascertain both in the field and in the literature, and thus we do not consider this ecologically and culturally different area relevant for our study.

Also, when the only criterium is the architectural style of the earthworks, in what sense are Aquiry earthworks different from all the other similar earthworks found in the Amazon (Peripato, et al., 2023)? I would expect 1) a clear definition of the architectural features that set the Aquiry apart from other earthwork styles and 2) show that everything considered Aquiry fits the definition. Otherwise, in my opinion, the definition of what constitute the Aquiry is arbitrary.

As we have explained earlier, it is precisely these distinctive features and the chronology of these geoglyphs that distinguish them from other known earthworks. We consider it unnecessary for a short article to include detailed discussion of issues that are not new, but have already been extensively examined in previous studies. Most recently, the characteristic features of Aquiry earthworks and their variability have been discussed by Kalliola et al. (2024), while their relationship to road systems has been addressed by Kalliola et al. (2026, see also Virtanen 2024). The relationship between the Aquiry and the earthworks of Baures and other nearby areas has, in turn, been examined by Pärssinen et al. (2024).

Combining the data from the second group of flights (covering 2,380 km²) with the already known archaeology, they estimated the number and density of earthworks for the whole 183,000 km² area.
The first step of this estimation is based on multiplying all previously detected earthworks by five, because the LIDAR detected five times more earthworks than were detected in deforested areas by looking at optical satellite imagery. This is where my first concern arises. It is possible, and I would expect it, that the undetected earthworks in satellite imagery are smaller and/or of a different type than those detected only by LIDAR. This being the case, they cannot just multiply all the earthworks by five. For example, a rectangular geoglyph with a 4-meter-deep ditch is likely to be identified in both datasets, while a shallow canal or small road could have gone undetected on optical images. Therefore, while the number of earthworks is five times greater, the amount of habitational or ceremonial sites is not. And, most importantly, the amount of work needed for earthwork construction is not five times larger.

Many factors affect the visibility of earthworks. In particular, trenches and embankments in open areas may have eroded, become infilled, and leveled over time, while their visibility may also be reduced by tall grass or shrubbery, regardless of the size of the structures. Therefore, we have included all sites in our analysis and calculated an average accordingly. As we have already noted to Referee 3, canals and roads are not included in any of the calculations presented in this article, contrary to what is repeatedly implied. Including roads (n = 955) would nearly double the number of sites identified through satellite data. Roads are also excluded from the calculations presented by Peripato et al. (2023). The factor of five was specifically calculated relating to ceremonial centers and settlements only. To ensure clarity, we will add a note to this effect in the text accompanying Extended Data Table 1a–c. Furthermore, it should be emphasized that these figures, derived from satellite-based extrapolation,

have not been used in the primary estimates of earthwork numbers or population size, but only as reference material.

The second step consists of estimating the number of earthworks in between the 10-km-spaced LIDAR scans. They basically count how many earthworks are detected in a 1-km swath and multiply them by 10 to account for the whole area. This is where my second big concern arises. The average earthwork is 200 meters in diameter, meaning that a lot of earthworks are cut by the 1-km swath. See a clear example of this in their ED Figure 5, where a large circular ring which is only partially within the scan is counted (red point) as being within the swath. When they multiply the number of earthworks detected in one swath by 10, they are basically counting all the earthworks which are partially outside the swath twice. This results in a further overestimation of the number of habitational or ceremonial sites.

This is indeed an important point. In QGIS, this issue can be addressed by calculating the centroid of the detected structure and assigning it to the tile in which that centroid is located (i.e. the tile covering more than 50% of the total area of the structure in question). This approach avoids double counting, even when a structure spans two tiles. Referee 3's example is therefore correctly recorded based on the centroid (red dot). However, we re-examined all six LiDAR swaths in question—covering nearly 2,500 km—twice more, during which we identified a total of 11 cases for the Aquiry period and 2 cases for the Mound Village period in which features had not been registered, even though their centroids lay outside the tile. This occurred along the longer edges, where the program did not flag the issue, unlike along the shorter edges linking the tiles. In total, this resulted in a small error, which has now been corrected in all calculations. We are grateful for this observation, as it contributes to identifying the issue and improving the accuracy of our results.

The third step consists of extrapolating what has been estimated in the second step to what they consider to be the high-density areas of the Aquiry, which cover 85,000 km². They do this by assuming that 1) the density of earthworks in forested areas is the same as in deforested areas. In supplementary table 2, they take the number of earthworks visually detected in optical satellite images, then they multiply this number by 5 (I already discussed why I don't agree with this approach) and then they multiply this number for the ration between forested over deforested area. First thing I don't understand is why they do this for sectors 1 and 8, for which they have the actual lidar scan. The have a measurement (3200 earthworks detected by lidar in Block 1), but they

don't use it. They use the lidar data only to come up with a 5X factor that they apply to all the deforested areas, concluding that in Block 1 there are 5163 earthworks rather than the 3200 actually detected by LIDAR.

This issue appears to relate to Extended Data Table 2 and the section "*Factor five for satellite imagery*." The referee questions why we do not use LiDAR data for Blocks 1 and 8, even though such data are available. Our response is that we use satellite imagery data, because the analysis concerns satellite imagery. In our view, it would not be methodologically sound to mix datasets obtained using different methods within the same comparison. Only the results of the satellite-based analysis can subsequently be used for correlation with other types of data, as we have done in our study. As previously noted, extrapolation based on satellite imagery yielded, on average, values approximately 18% higher than those obtained using LiDAR data. The most recent corrected extrapolation indicates a difference of 23%. This is remarkably consistent with the observation that approximately 11.5–41.9% of earthworks in the core area likely remain undetected in our LiDAR survey (lines 213–218).

The second thing is that they assume that the density of earthworks in forested areas is the same as in the deforested. But, it can be argued that deforestation follows the existence of roads and modern roads often follow the same path of ancient roads. In fact, McMichael et al. (2017) have shown that modern accessibility to areas of the Amazon correlates with the number of archaeological sites. They have the actual LIDAR for two blocks to test if this is the case or not.

Of course, there is some correlation between roads—especially major ones—and known archaeological sites. Brazilian law has long required archaeological surveys to be conducted prior to the construction of public roads and power lines. Once roads are built, they also provide the easiest means of accessing surrounding terrain and locating new archaeological sites, as movement through dense forest is extremely difficult. It is therefore natural that a correlation exists between roads and surveyed sites. However, it is important to note that tens of thousands of kilometres of small roads have been constructed in the western Amazon in recent decades to clear forests and establish pastures and farmland. These roads typically follow a grid pattern, often spaced approximately 5 km apart, from which logging and forest clearing proceed. Over the past 20 years, we have used satellite imagery to track deforestation, and the number of earthworks identified has increased at a comparable rate. Their density has remained

remarkably consistent, even in newly deforested areas. Importantly, however, we have not observed any clear correlation between these modern, ruler-straight roads and pre-colonial road systems, of which we have identified a total of approximately 350 km (Kalliola et al., 2026).

Their sensitivity analysis demonstrated that regions with denser forest cover experienced about a 10% drop in LiDAR penetration to the ground compared to more open regions, 10% less points shouldn't preclude the detection of large earthworks. However, they also state that "11.5% - 41.9% of the core area was determined to be impenetrable by laser". This second statement is in sharp contrasts with the previous one. Overall, I feel that they are not being clear with what happened with the LIDAR data in the forested area.

It appears that Referee 3 may have overlooked parts of our text and related analysis in the Supplementary Information. Twelve test areas were selected as the training set, including Fuso 19 (Block 1) and Fuso 20 (Block 8), as well as areas characterized by open, mixed, and continuous forest cover. A total of 132 modelled point-cloud images were generated from these data, after which the entire ALS area (4,496.98 km^2) was processed using these models. A more detailed description of the testing procedure and methodology is provided in the Supplementary Information (lines 252–353).

In the LiDAR tiles of the Fuso 19 area (approx. 1 × 2 km), the accuracy required to observe ground patterns was not achieved, with up to 11.3% of the ground remaining undetected even in relatively open areas. In the densest forest areas, this proportion increases to 41.9%. At Fuso 20 (Block 8), forest density is higher, and ground visibility is approximately 10% lower, as noted in our text. The proportion of potentially undetected earthworks reaches ≥17.8% at ground level and up to 54.5% in the densest forest areas.

We do not fully understand where the confusion may have originated, possibly from the following sentence in the Supplementary Information (lines 298–299): "*Furthermore, the results show that the Lábrea region, with denser forest cover than the Rio Branco region, had on average 10% lower LiDAR penetration to the ground.*" In this sentence, we are only comparing overall penetrability between the two areas, referring to observed differences in average ground point density. These differences do not directly

indicate potential geoglyph occlusions, as identifying occlusions requires a more robust evaluation of the spatial distribution of low-penetrability zones, rather than relying solely on average differences. We performed this evaluation in three main steps in our study. This challenge was also noted in the Supplementary Information (lines 307–311): "*The estimation depends on several factors in addition to the ground points resolution. These include the geoglyph area, the earthwork edge height variance, and the ground point density of neighboring raster cells. Thus, while the number of ground points per square meter provides a useful indication, it alone cannot guarantee whether earthworks can be detected or not*"

Step 1 — Threshold identification: We determined that geoglyphs become undetectable when the point density of an open area, assuming this is the highest resolution area where geoglyphs are normally visible, is reduced to 10% of its original resolution. This corresponds to approximately 1.7 ground points/m², below which geoglyphs cannot be detected.

Step 2 — Pixel-level assessment: We then calculated how many 3 × 3 m raster cells fell below this threshold. The results ranged from 46% (Fuso 19) to 54% (Fuso 20), indicating the proportion of small areas that fall below this threshold. As noted in the Supplementary Information (lines 303–306): "*However, this result only indicates the proportion of the raster cells with ground point density below the detection threshold in the study area. The result does not indicate how these raster cells are spatially aggregated, which can affect the analysis regarding the regions where earthworks could be undetectable due to the point cloud density.*"

Step 3 — Identification of aggregated low-penetrability areas: Finally, we searched for large, contiguous areas (≥1 ha) where these low-density pixels were aggregated and met the 1.7 pts/m² criterion. These represent areas where potential geoglyph occlusion could occur. We found that in Fuso 19, at a minimum, 11.3% of the area can be classified as unknown. In contrast, LiDAR penetrability in Fuso 20 is lower, with 17.8% of the area remaining unknown.

In summary, we clearly state in the main text that the Fuso 19 area corresponds to the Rio Branco region and the Fuso 20 area to the Lábrea region (lines 176–177). However, to avoid further misunderstanding, we will also include this information in the relevant sentence in the Supplementary Information: "*Furthermore, the results show that the*

Lábrea region (Fuso 20), which has denser forest cover than the Rio Branco region (Fuso 19), had on average 10% lower LiDAR penetration to the ground." There are no discrepancies in the results.

These data should be shown, they should say how many earthworks they found in the forested areas of block 1 and 8.

Verified earthworks—identified through satellite data or LiDAR—are shown in Fig. 3a and Extended Data Fig. 5. Coordinates of satellite-identified sites are provided in the appendix to Kalliola et al. (2024), and LiDAR-derived site coordinates are listed in the Supplementary Information (Table 2).

Despite claiming that their data come from LIDAR survey, in reality, LIDAR survey is only used (wrongly) to infer the factor 5. All the data used to come up with number of earthworks and population are based on already known number of earthworks in the deforested areas, multiplied by 5 and then extended to the whole area.

With all due respect, this statement is not accurate. Referee 3 appears to have overlooked this point. The figures of 24,700 extrapolated earthworks and 1.3–3.1 million people are derived from our LiDAR data, not from previously known numbers of earthworks in deforested areas (see lines 203–212 and 247–261 of the main text).

The overestimations of these three steps are cumulative, because each step makes a further overestimation of the previous overestimation, making their final number of 24,700 earthworks in the Aquiry region, and their claim that "this cultural area alone contains as many earthworks as previously estimated for the whole of Amazonia," unsupported.

We agree that we overestimated 13 earthworks in our six LiDAR swaths, and this has now been corrected. The revised total is now 23,760 earthworks. Importantly, this figure does not account for LiDAR impenetrability in the core area, which ranges from 11.5% to 41.9% (average 26.7%). Our estimate should therefore be considered conservative and remains well supported.

In the second part of their manuscript, Pärssinen et al. estimate the population density of the Aquiry culture. They do this in two ways: through energetics (by estimating the

amount of work needed to build all the earthworks they estimated to be in the Aquiry area) and ethnographically, by estimating how many people could have lived in what they consider to be habitational mounds.
The first type of estimation is just an arithmetic scaling of what the same authors estimated to be the population needed to build each geoglyph. This work they cite (Pärssinen, et al., 2009) is based on several simplistic assumptions, starting with: "If one agrees that one man or woman could move 1 m³ per day". For example, they do not consider the huge differences in moving 1 m³ when building a causeway vs. excavating a 4-meter-deep ditch or building a mound.

We apologize, but we do not fully understand why this issue should be addressed in the article, as there are no causeways in the Aquiry área.

However, they cite Hirst et al. (Hirst, et al., 2025). Here, Hirst et al. have done the exact same thing, estimating population density for the Casarabe culture by looking at the energetics of earthwork construction. But despite Pärssinen et al. knowing this paper, they don't engage with it. It is very apparent that Hirst et al.'s methodology is far more sophisticated and reliable than what Pärssinen et al. have done in their 2009 paper. I think they should use the methodology in Hirst et al. for two very important reasons. 1) Hirst et al. account for the energy and time involved in not only digging soil, but also transporting it, so that the number of people needed to build 1 m³ of a mound is larger than the amount needed to build 1 m³ of a causeway, for example.

As Referee 3 is well aware, the article by Hirst et al. (2025) was published after we had already submitted our manuscript for peer review. We included it only in the final version to reference the concept of "*low-density urbanism.*" However, the two cases differ in several important respects. As noted earlier, the Aquiry region is located in dense tropical forest, whereas the Casarabe culture developed in an open savannah environment, with well-documented settlements, causeways, and raised fields—all of which remain largely unidentified in the Aquiry cultural context. As also noted by Referee 2:

"Furthermore, reviewer 3 finds fault with Parssinen et al. because their population estimate is higher than that of Hirst et al. (2025) based on Casarabe in the Llanos de Mojos. But Hirst et al.'s estimate is not based counts of habitations in the archaeological habitation mounds, since no one mapped houses and their hearths by ground-penetrating geophysical surveys and stratified-random test excavations at Casarabe as has been done for Marajoara mounds (Bevan and Roosevelt 2003; Roosevelt 1991, 2007). Thus, the site-population estimates for Casarabe don't actually

count people directly and should not be used to rebuke Parssinen et al. for their estimates, also based on mound counts. And as Parssinen et al. point out in their response to the reviewer, the reviewer's comparison treats differently the counts of different types of mounds in the two regions.
In addition, Hirst et al. base their population estimate in part on the notion that the Amazon has a limited carrying capacity. Do some researchers really still assume there is a set carrying capacity for a habitat? I thought we were beyond that, given that Amazonia has shoved in everyone's faces the fact that at various times it supported occupations of dense, sedentary populations for long periods without evidence of significant deforestation. Most of us now understand that different technologies of production permit different carrying capacities and that different regions have different sediments, which affect their ability to support intensive production. In the Llanos, overgrazed Hispanic cattle pastures have replaced prehistoric earthwork field systems built for intensive agriculture in relatively poor soils. But the Aquiry has a range of soils considered to have good agricultural potential."

2) Hirst et al. account for the timespan over which earthworks have been built. It is not the same to assume that the same number of earthworks has been built over 50 years as over 500 years; the first case would imply 10 times the population density of the latter. Pärssinen et al. don't engage with this temporal aspect at all. They estimate that about 25% to 60% of the total estimated Aquiry earthworks were in simultaneous use during a 200 year long window coming up with a figure of 4,360 to 10,460 earthworks that were co-existing/used during that 200-year peak. But use or co-existence doesn't imply any similarity with the amount of work needed for construction.

Directly or indirectly, there is certainly a connection. The larger the earthwork, the larger the community that built and used it. Of course, the amount of labor invested in different daily tasks may have varied from year to year. Nevertheless, there is no evidence of itinerant labor groups constructing earthworks; most likely, this was the responsibility of the local community. This interpretation is supported by the previously mentioned oral history, which clearly indicates that each community divided the work between those who burned the forest and those who dug the earthworks. Approximately every 27–28 years, communities had the opportunity to undertake such construction during the bamboo-dominated forest die-off events, although they also had to maintain many other responsibilities. Communities with fewer than 60 adult men (or approximately 300 people in total) would not have been able to construct and

continuously maintain an average 3 ha earthwork, given their various other obligations, which must also be taken into account.

They do state that the Aquiry built an average of 22 sites per year, but then they calculate the total population simply by multiplying a total of 4,360 to 10,460 earthworks (which represent earthworks used) by their estimate of 300 people (which is the number of people they said are needed for construction), concluding a population of about 1.31–3.14 million, in fact equating the energetics of construction with those of use and/or maintainace. In this way, they add another big overestimation to the previous overestimations.

It appears that Referee 3 has not fully captured the points we explained earlier. The figure of 300 people represents the estimated average population of communities (including non-working individuals), whose adult members constructed earthworks of average size during the years when this was possible, namely during the bamboo-dominated forest die-off events that occurred approximately every 27–28 years. According to strong archaeological evidence, these communities typically maintained their ceremonial centers for 200 years or more, before they were eventually abandoned. During the most active period of the Aquiry (c. AD 100–300), our current estimates suggest that between 4,165 and 10,000 earthworks were in active use, corresponding to a population of approximately 1.25–3 million. This is not an overestimation, as demonstrated by comparisons with earlier estimates of ancient earthworks, later mound village settlements, and the size of ceremonial fields even today.

They also estimated the population size by using ethnographic analogues in their paragraph titled “The Aquiry enclosures vs. later mound villages”. Here, the average village had 11.75 multi-family mounds; each mound housed a minimum of 25 people; 11.75 multiplied by 25 yields approximately 294 (or roughly 300) people per village. But, in ED table 1 they say that the Aquiry earthworks are “Geoglyphs” and “Quad and multilateral embankment” and “mound sites” are classified under “other”, meaning not Aquiry. How did they estimate how many habitational sites where Aquiry? At 300 people per village, we need between 4,360 and 10,466 Villages to come up with a figure comparable to the one based on construction energetics. But, 4360 to 10,466 is the total number of earthworks, not the number of villages, which is likely far smaller (they explicitly state that Aquiry earthworks were ceremonial, non-residential centres). Basically, I couldn’t understand what calculations led them to say: “In every case, when comparing the possible population size of the later villages with communities that maintained earlier ceremonial and political centres, rather than the size of the structures, there is a match with our estimation of c. 300 people per average earthwork and c. 1.31–

3.14 million people or more at the peak of the Aquiry." In my opinion this latest statement is also unsupported.

This statement is somewhat surprising to us. We explain clearly that, despite cultural changes that led dispersed settlements—likely consisting of *maloca*-type individual houses—to organize around round or rectangular plazas, there is no evidence of a radical population replacement in the former Aquiry area. It is therefore likely that these settlements represented communities similar to earlier ones, previously more dispersed but still maintaining shared ceremonial centers. Using a minimum of 25 individuals per communal longhouse (*maloca*), we estimate an average population of approximately 300 people per mound village, despite their smaller spatial extent compared to Aquiry ceremonial centers. This suggests that Aquiry communities and later mound villages had broadly similar average population sizes. Only in the highly unlikely case that c. 25-m-long house foundations belonged to a single family would the population size have been much lower. Hence, the population estimate of approximately 1.25–3 million people is strongly supported.

Referee # 5

Thank you for the work on the paper to bring the focus on to the population estimates. While I consider those estimate to be too high because each mobile group could built and use several features per year without overload given all we know about the readiness with which people can carry out construction logistics, I do not proposed to critique that issue at this time. That can wait for other occasions of debate and discussion.

We would like to thank Referee 5 for his previous comments, which we have addressed in the revised manuscript with additional discussion and analysis in the Supplementary Information. However, we maintain that although hundreds of kilometers of roads were constructed, indicating a high degree of mobility, the vast majority of the population was sedentary, remaining in the vicinity of ceremonial centers. Archaeological and related evidence indicates that ceremonial centers and the roads leading to them were continuously maintained, while surrounding open areas were used persistently for agriculture. There is no evidence for repeated abandonment of these centers. In contrast, at ceremonial centers and settlements associated with the highly mobile groups we have studied elsewhere, such short-term abandonments and burn marks from re-clearing

activities are clearly evident. We also note that sedentary communities based on maize and manioc cultivation became widespread elsewhere in the Amazon during the first millennium BCE. Referee 2, who has extensive expertise in the Amazon region, makes a similar observation: "*Reviewer 5, however, also doubts that in Amazonia settlement was permanent. They suggest that habitation sites might have been lived at episodically, reducing the possible estimated population for the occupation of the Aquiry mounds. However, the excavations by numerous researchers at a wide range Amazonian sites from c. 500 BC to c. 1500 AD shows that residence was indeed often permanent. One has only to look at the stratigraphic profiles published for Amazonian and Orinoco mound sites to see that there is rarely a gap showing abandonment between house floors in occupation layers*." We therefore welcome continued debate on this important topic.

There is, however, a statement in lines 288-91 which is problematic because it continues to claim that a comparison to other 'urban' settlements is meaningful by a reference to German settlements which are labelled 'urban' and have populations in the 100-1000 range. As mentioned before a region may use the label 'urban' that way but others may not so the case could only be made for the validity of a generalisation across continents, timespans and disparate cultures if it could be shown that this was a universal correlation of the label and specific population sizes. As is well known there are numerous different definitions, designations of urbanism and specifications for it.

As we already answered to this question in our previous Point-by-point discussion: "*We agree that there is no such thing as a standard urban density. In fact, European urban cities were not intended to be a basis for comparison, but rather to help the reader to understand more about Western tradition by showing that medieval towns were not large either. Furthermore, European medieval towns were quite autonomous, compact units where agricultural fields and forest resources were located outside the walls, as was the case with ditched and embanked geoglyphs.*" It is common in the social sciences and humanities to refer to better-known cases to help readers understand an argument. In this case, a reference to medieval Germany helps illustrate that not all urban-like centers are large. Just as states such as China and Tuvalu differ greatly in size, they are nevertheless both considered states.

The phrase in lines 360-61 also needs some change of terms. There may be many different concepts in use. The core issue is that there is no agreement about the many meanings attaches to the label 'urban'. That is point being made in the paper which is being referred to.

OK. We modified the phrase accordingly.

I wish the authors well with the publication of this paper. I consider that the new understanding of the diversity of settlement forms from research in Amazonia is profoundly important. I do not consider that its importance is conveyed or enhanced by forcing those diverse, remarkable phenomena into the old category of "urban. That may be awkward, but our job as researchers is to find, recognise and report what is not familiar, rather than seek to normalise it.

OK. Thank you.

References

Hirst J., Singarayer J., Lombardo, U. & Mayle F. Constraining the population size estimates of the pre-Columbian Casarabe Culture of Amazonian Bolivia. *PLoS One* **20**(5): e0325104 (2025). https://doi.org/10.1371/journal.pone.0325104

Kalliola, R., Pärssinen, M., Ranzi, A. & Barbosa, A.D. Ancient Amazonian Earthwork Roads: Unveiling Ceremonial, Livelihood, and Networking Significances. *Lat. Am. Antiq*. (2026). https://doi.org/10.1017/laq.2025.10168.

Kalliola, R., Pärssinen, M., Ranzi, A., Seppä, I. & Barbosa, A.D. Geographies of ancient geometric earthworks and their builders in Western Amazonia. *Acta Amazonica* **54**, e54hu22351 (2024). https://doi.org/10.1590/1809-4392202203511

Pärssinen, M. Tequinho Geoglyph Site and Early Polychrome Horizon 300 BC – AD 300/500 in the Brazilian State of Acre. *Amazônica: Revista de Antropologia* **13**, 177-220 (2021). https://doi.org/10.18542/amazonica.v13i1.9095

Pärssinen, M., Ferreira, E., Virtanen, P.K. & Ranzi, A. Domestication in motion: Macrofossils of pre-colonial Brazilian nuts, palms and other Amazonian planted tree species found in the Upper Purus. *Environ. Archaeol. **26***, 309-322 (2021). https://doi.org/10.1080/14614103.2020.1765295

Pärssinen, M., Kalliola, R. & Ranzi, A. Zanjas circundantes en el extremo norte de Bolivia (Pando y Beni) y sus relaciones con los llamados geoglifos de Acre. *Arqueoantropológicas* **9**(9),15-49 (2024).

Peripato, V. *et al*. More than 10,000 pre-Columbian earthworks are still hidden throughout Amazonia. *Science* **382**, 103-109 (2023). https://doi.org/10.1126/science.ade2541

Saunaluoma, S. & Virtanen, P.K. "Variable models for organization of earthworking communities in Upper Purus, southwestern Amazonia: Archaeological and ethnographic

perspectives. *Tipití: Journal of the Society for the Anthropology of Lowland South America* **13**(1), 23-43 (2015).
https://doi.org/10.70845/2572-3626.1196

Schaan, D. *et al*. New radiometric dates for precolumbian (2000–700 BP) earthworks in western Amazonia, Brazil. *J. Field Archaeol.* **37**, 132-142 (2012).
https://doi.org/10.1179/0093469012Z.00000000012

Virtanen, P.K. Paths and Networks Beyond the Human in Amazonian Social Worlds. in *Lowland South American World*, (ed. High, C. & Costa, L.) 349–366 (Routledge, London, 2025).

Virtanen, P.K. & Saunaluoma, S. Visualization and Movement as Configurations of Human–Nonhuman Engagements: Precolonial Geometric Earthwork Landscapes of the Upper Purus, Brazil. *American Anthropologist*, **119**, 614-630 (2017).
https://doi.org/10.1111/aman.12923

Watling, J., Saunaluoma, S., Pärssinen, M. & Schaan, D.P. Subsistence practices among earthwork builders: Phytolith evidence from archaeological sites in the southwest Amazonian interfluves. *J. Archaeol. Sci. Rep.* **4**, 541-551 (2015).
https://doi.org/10.1016/j.jasrep.2015.10.014
