# Supplementary material for: Over 20,000 precolonial earthworks in the Southwest Amazonia
Source: Nature. 2026 Jul 29;657(8131):420–5. doi: 10.1038/s41586-026-10835-7 (PMC13558069; doi:10.1038/s41586-026-10835-7)
Supplement: Supplementary file 3 [file 41586_2026_10835_MOESM1_ESM.docx]

**Supplementary Information**

**“Over 20,000 pre-colonial earthworks in the Southwest Amazonia” (Martti Pärssinen et al.)**

**Supplementary Note 1**

a. **The name of the Aquiry civilization**

The name Aquiry originally referred to the Acre River, as named and explored by W. Chandless (1866)^57^. It flows from Peru and Bolivia to the Brazilian state of Acre. Passing through the heart of the ancient civilization that built the geoglyphs, it joins the Purus River in the Brazilian state of Amazonas as a major tributary. The Aquiry name probably comes from the Apurinã (Arawakan) word “kaikyry”, meaning “caiman” – hence the “River of Caimans”^18^. We use the term 'civilization' to describe this seemingly multicultural complex society^1^. They shared a socio-cosmology, which is reflected in their geometric monumental enclosures with road systems. This is somewhat similar to the manifestation of multicultural Christian civilization through cathedrals and churches, or of multicultural Islamic civilization through mosques. Both have their own iconography and many shared social practices, as seen also in the Andean Chavín civilization, for example. For more information on the origin of the concept of civilization and its use in the context of South American archaeology and anthropology, see Ranzi & Pärssinen (2021, 19-64)^2^.

b. **Radiocarbon chronology**

So far, 16 geoglyph sites and 19 ditched and embanked structures (i.e. geoglyphs) have been dated using 82 out of a total 162 radiocarbon samples obtained from three sub-regions: eastern Acre, southern Amazonas, and northern Bolivia (Supplementary Tables 1a-b). Furthermore, 16 mound village and roundish embankment sites were dated using 44 samples, all but one of which appear to be from later periods than the geoglyphs. Additionally, three samples not directly associated with earthwork structures but with nearby agricultural fields were dated to the Aquiry period. Finally, 31 samples from seven earthwork sites appear to predate the structures themselves, suggesting earlier human activity at these sites^58^.

Current studies on climate dynamics have revealed a significant influx of air masses and moisture from the Northern Hemisphere over southwestern Amazonia during the South American Summer Monsoon (SASM). Therefore, when calibrating ^14^C raw data, it is recommended^54^ that in SW Amazonia one uses a mixed SHCal20 curve for the Southern Hemisphere^55^ and an IntCal20 curve for the Northern Hemisphere^56^. In this article, we therefore use a 50/50% mixed calibration curve. All dates are presented in Supplementary Tables 1a-b.

Many SW Amazonian earthworks were constructed on sites with a deep human history. On various geoglyph sites, charcoal and ash began to accumulate at the onset of the Holocene era, c. 8,000 BC^58^, and persisted periodically until the emergence of monumental earthworks in the first millennium BC (Supplementary Table 1a). Furthermore, the presence of earlier charcoal in sites such as Los Angeles, Ramal do Capatará, and Severino Calazans has led to supposition that the geometric earthwork tradition started c. 1,200-900 BC^59-60^. However, further analysis and radiocarbon dating in Severino Calazans, Tequinho, Eletronorte I, and Piçarreira indicate that the earliest embankments and ditches were constructed after 750 calBC and that older dates are indicative of earlier activities at the same sites (Supplementary Table 1a). Even at the Los Angeles site, most dates (8/10) seem to fall within the period between 540 BC - 722 AD. However, in this study, the radiocarbon beginning and ending boundaries of the entire Aquiry phase were analysed using Bayesian modelling to address the tendency to overestimate the length of archaeological phases due to uncertainties caused by old wood and calibration probabilities, among others. Furthermore, the Aquiry period and its three sub-regions have been summarised using a Kernel density estimate (KDE) phase modelling.

Bayesian modelling results in mean values of the beginning and end boundaries of the Aquiry phase as 560 ± 40 BC and 810 ± 40 AD, respectively. Considering 40 years of one sigma uncertainty, the entire modelled Aquiry period can therefore be tentatively dated to c. 600 BC–850 AD (Extended Data Figures 2a-b). Furthermore, it appears that most sites were occupied for 200 years or more, with some being occupied for over 1,000 years (Extended Data Figure 7).

In addition, the OxCal 4.4.4 KDE_Model function^54^ was used to estimate the relative intensity of human activity. This identified a local maximum of the Aquiry period in the calendar-year probability distribution of 250 BC – 400 AD (Extended Data Figure 6). This suggests that the construction of dated structures belonging to the Aquiry cultural phase was at its peak during this period, indicating intense human activity. In fact, judging by the ratio of the KDE distribution sums during the peak and full periods, as well as the associated uncertainties in the KDE plot, the peak corresponds to 61% ± 20% of the full KDE distribution. Since the dates were derived directly from the structures, it can be estimated that approximately 60% of them were constructed during the peak period.

This model illustrates the relative intensity of the entire Aquiry phase. However, it does not fully account for the fact that at least two centres from the first peak period (Los Angeles and Severino Calazans) were still in use during the main second peak, nor the fact that several centres in use in c. 200 AD, when the peak was at its highest (Extended Data Figures 7-8), were built one or two centuries earlier. The KDE model function estimates that 25% of construction activities date to the period 100–300 AD. Therefore, it is also important to examine how many earthworks were in use and how many were cleaned and maintained during this period, according to ^14^C dates. It should also be noted that there currently seems to be a consensus that the construction of these earthworks was based on the area's bamboo-dominated forests and their massive die-off every 27–28 years^3,6,45,46^. This allowed people to burn forest openings for few months and create geometric enclosures, wide roads, and agricultural fields in these areas four times every hundred years. Furthermore, our archaeological excavations and more than a hundred test pits have demonstrated that the enclosures were regularly cleared of vegetation and maintained for at least 200 years after construction, with no evidence of burning. Where more than one layer of use was observed with a break, the other layers were also in use for hundreds of years, with no evidence of additional abandonment or forest burning. Typically, a cultural layer associated with the use of a geoglyph on flat ground varies in depth from 20 to 45 cm (e.g., Supplementary Figure 5B). In only a few cases, it has reached a depth of up to 70 cm^9,21^. Instead, if older cultural layers were discovered below those stratums associated with geometric enclosures, signs of periodic burning episodes were regularly observed^58^.

Examining the overlap of the radiocarbon dates of all sites using KDE-modelled summaries (Extended Data Figure 7) reveals that approximately 60% of the dated centres were used simultaneously with considerably high probability around 200 ± 100 AD. All the radiocarbon dates related to ceremonial activities from Amazonas (n = 4) and Bolivia (n = 3) date to this period, as do around 60% of Acrean geoglyphs. Hence, considering all uncertainties, we estimate that the most likely probability of simultaneous use of all earthworks in 100–300 AD is 25% - 60%, with a strong preference towards the upper limit.

In any case, our current radiocarbon dates indicate that ceremonial activity continued after the main peak at the well-dated Tequinho site, but afterwards the inner ditch of the main geoglyph (pit 18) was no longer kept clean and open^8^. Instead, the cleaned earth of the enclosure piled up in the ditch between calAD 420 and 650, and after this the site was eventually abandoned (Supplementary Table 1a). At the Severino Calazans site, the ditched enclosure was abandoned after the main peak, but a strong human activity continued outside the geoglyph in the north around calAD 405-640. However, radiocarbon dates from the Balneário Quinauá site show that new ceremonial centres were also built and used soon after the main peak.

Finally, the Severino Batista, Fazenda Colorada and Jacó Sá sites show that various sites were reused, and new ones were built between AD 600 and 850. After this period, the construction of geometrically planned, ditched ceremonial centres appears to have ceased. However, some of them, such as Fazenda Colorada, Fazenda Iquiri II, Las Palmeras and Três Vertentes, were reused for later mound settlements; and some roundish embanked enclosures (without ditches) also give late post-geoglyph dates in our study area. In summary, our tentative chronology of earthworks for our study area is as follows:

1.	First (I) or Initial Geoglyph Period, c. 600-250 BC, when the first geometrically planned earthworks were constructed and used at sites such as Severino Calazans and Eletronorte I.

2.	Second (II) or Main Geoglyph Period, c. 250 BC - 400 AD, when most of the ditched earthworks were built. In particular, in our list of 16 dated geoglyph sites, up to 60% were in use at the same time in c. 200 AD. The beginning of maize and squash cultivation^37^, as well as the known use of early polychrome pottery^18^, corresponds to this period. Interestingly, the expansion of manioc and peach palm farming is also said to be associated with the expansion of polychrome ceramics^61^.

3.	Third (III) or First Intermediate Period, c. AD 400-600, when several ditched earthworks were abandoned, but some new earthworks, such as the Balneario Quinauá, were constructed.

4.	Fourth (IV) or Final Geoglyph Period, c. AD 600-850, when some earlier ditched enclosures were reused, such as Fazenda Colorada and Severino Batista, and new ones were built, such as Jacó Sá.

5.	Fifth (V) or Second Intermediate Period, c. AD 850-1200, when earlier ditched enclosures were almost completely abandoned without the construction of new ones. Nevertheless, it is possible that some circular embankments and mound settlements were built during this period.

6.	Sixth (VI) or Pre-Colonial Mound Settlement Period, c. AD 1200 - 1500, when new types of mound settlements appeared as a new boom in the core of our study area. Nine out of fourteen dated mound settlements (c. 65%) belong only to this period, and three (c. 22%) belong both to this and the following Colonial period. In particular, in our list of 14 mound settlements, 11 (c. 79%) were in use simultaneously in AD 1425, and a roundish embanked enclosure at Campo Esperança is also dated to this period.

7.	Seventh (VII) or Colonial Mound Settlement Period, c. 1500 - 1808/1825. During this period, two dated mound settlements (c. 15%) were constructed and three (c. 22%) mound settlements constructed in earlier chronological phases continued to be used. In addition, a later roundish embanked enclosure (Tênue) belongs to this period (Supplementary Tables 1a-b).

c. **The size and contemporaneity of the Aquiry earthworks in three sub-regions: a comparison**

As mentioned, around 60% of the dated geoglyph type earthworks appear to have been constructed during the main peak period from 250 BC to 400 AD. Most of the dates in the Acre region originate from the *terra firme* area located between the Acre and Abunã rivers (Fig. 1) to the north-east and south-west of the capital, Rio Branco. Those from Amazonas come from the south-east of Boca do Acre, near the main channel of the Purus River. The Bolivian dates originate from an area near the confluence of the Madre de Dios and the Beni rivers, to the east and south of Riberalta (Extended Data Figure 8). Boca do Acre is approximately 300 km from Riberalta as the crow flies, and Riberalta is 240 km from Los Angeles, the easternmost dated geoglyph in the Rio Branco sub-region. Thus, the core area of the Aquiry civilization is represented.

In this particular comparison, we measured the interior size of the dated geoglyphs to make them comparable with our database of satellite imagery obtained from the same blocks 1, 2 and 5. In this database, all geoglyph sizes refer to interior space, because each ditch of a geoglyph is situated inside the exterior embankment, creating a natural and easily detectable shadow. Later, when we compare our LiDAR data to the mound settlements, we will use exterior embankments and mounds in our size calculation. According to our tests of 24 LiDAR geoglyph images (Block 1), in which both exterior and interior sizes were calculated, the difference between interior and exterior sizes was 24 ±9% in the 0.2–1.75 hectares category, 18 ±12% in the 1.76–6.0 hectares category, and 12 ±6% in the 6.01–16.0 hectares category.

In Acre, the smallest dated geoglyph is a 0.35-hectare interior circle (Balneario Quinauá), connected by a road to a 1.2-hectare (interior) rhombus geoglyph with rounded corners. The second-smallest dated geoglyph, measuring around 0.55 hectares, is a rectangular structure with rounded corners and sides (Severino Batista). The largest and one of the oldest dated geoglyphs in Acre, Eletronorte I:1, has an 8.5-hectare octagonal plaza with five radiating roads (Supplementary Figure 4A). The rest of the Acrean earthworks range in size from 1.15 to 5.2 hectares. In the state of Amazonas, one site has been dated using four samples. This site, known as Cruzeirinho^8^, contains an older quadrilateral structure with a plaza covering around 15.6 hectares. This plaza is partially obscured by a newer square geoglyph covering around 9.4 hectares. This newer structure is connected by a road to a smaller 3.1-hectare square which has been dated. Finally, in Bolivia, two geoglyphs were dated: Las Palmeras, which features a drop-shaped geoglyph with a 6.5-hectare plaza, and Tumichucua, which features a circular geoglyph plaza spanning nearly 50 hectares (the largest known). An embanked, drop-shaped enclosure (Estancia Giese, 3.7 hectares) in Bolivia also appears to belong to the geoglyph period.

The dated geoglyphs appear to be largest in Bolivia and the state of Amazonas, while the smallest were found in the state of Acre. This reflects the fact that the earthworks detected in satellite imagery were also larger in Bolivia and the Amazonas region than in Acre, particularly in the *varzea* of the Madre de Dios, the Beni, and the Purus. According to our database, of the 343 earthworks detected with satellite imagery in Block 1 of Acre (Extended Data Table 2), 276 are geoglyphs (80%) ranging in size from 0.193 to 19.269 hectares (interior), with an average size of 2.363 hectares. In Block 2 of the Amazonas region, the interior size of 169 geoglyphs varies from 0.441 to 15.660 hectares, with an average size of 3.723 hectares. In Bolivia (Block 5), where only around 5% of the land has been deforested, the interior size of 11 geoglyphs ranges from 1.853 to 49.718 hectares, averaging 8.320 hectares. Even when the largest geoglyph, Tumichucua, is excluded, the average size is still 4.180 hectares. Therefore, the average size of geoglyphs in the Amazonas and Bolivian regions seems to be c. 58% and 77% larger than in Acre, respectively.

It is also important to note that while most sites have only one earthwork, some of the more interesting sites have two or more earthworks close together, often connected by a road. This suggests that they were used simultaneously, although one or two of these may be slightly older. For instance, the smaller circular geoglyphs in Fazenda Atlântica and Balneario Quinaua appear to predate the square ones. However, in both instances, these two structures were connected by a road and were utilised concurrently following the construction of the squares^62^. Superposition does occur, too, but this is a rare phenomenon (in less than 2% of our LiDAR sample) and can indicate either renovation (e.g. Cruzeirinho) or the later use of an earlier structure (e.g. Fazenda Colorada).

Finally, three to five Aquiry structures have been established around an embankment at two sites in our LiDAR sample. A later mound settlement may also be located inside or outside the same conglomerate (see Extended Data Figure 4a), but it does not affect the calculations for the Aquiry earthworks. This relatively rare conglomerate type only exists in the vicinity of Rio Branco. Unfortunately, we do not have any radiocarbon dates for this type of earthwork conglomerate. Only one date exists for a later mound settlement inside Dois Circulos: Estrutura IV^63^. In summary, both the largest and smallest geoglyphs seem to belong to the main peak period, and in general, our radiocarbon dates suggest a 60% probability match with the Aquiry earthworks contemporaneity in these sites with multiple structures.

d. **A comparison of the population size of ceremonial centres and mound villages**

In order to estimate how many people lived in an average mound village when these settlements appeared in the former Aquiry core area in the second millennium, we used the recent data by Iriarte et al.^19^ for 20 circular mound villages with information about the diameter of each central plaza and the number of mounds arranged clockwise around it. The average diameter of the plaza in these 20 cases is 90.65 m, and according to satellite images, the mounds, which are between 6 and 10 metres (average 8 m) wide, extend from 15 to 35 metres (average 25 m) outside the plaza. In general, mounds are much bigger than 100 m² and can be classified as multifamily *maloca* longhouses^36,38^. Therefore, the original average exterior diameter of these settlements is approximately 140.65 metres, equivalent to 1.55 hectares, and the average number of longhouses in these villages is 12.6. The same authors also provided information on rectangular mound villages, although they did not provide exact data on their sizes^19^. Nevertheless, we managed to calculate the size of eight of these settlements using satellite imagery. Their average exterior size is 0.6 ha, and they contain an average of 9.65 mounds per village (Supplementary Table 3). Combining this information from circular and rectangular villages and assuming that the average pre-colonial family size in the area was five, as in the pre-colonial Central Andes^23,64^, and that an average longhouse was occupied by at least five families^38^, we arrived at 235 people per hectare and an average village size of 1.28 hectares housing 300 people. However, if we assume that the original houses in all mound villages were single-family homes rather than multifamily *maloca* longhouses and that these homes extended 6–9 metres (on average 7.5 metres) outside the plaza, then the area would have extended outward as families grew or houses were rebuilt. Hence, we would arrive at 47 people per hectare and 60 people (12 families) per average village. Nevertheless, multifamily houses have always been extremely common in the Amazon region. Even the earliest descriptions of northern Bolivian and Acrean villages, following catastrophic demographic declines caused by European diseases, mention villages with between 17 and 62 families surrounded by agricultural plantations^65-66,57^. Considering that these kinds of villages correspond to the archaeological remains of roundish and rectangular mound settlements, with an average of around 12 house foundations per site, the multifamily supposition is much more plausible.

In sum, the sizes of villages and earlier ceremonial centres in hectares seem not to be comparable 1:1, because ceremonial centres were also constructed for visitors from other areas, not just for the people who inhabited the nearby surrounding area. More time and labour were devoted to the construction of geoglyphs and embanked structures, so it is a question of the size of the communities rather than the size of the structures. Therefore, communities with a population of around 60 families and 300 people may have constructed much bigger and more monumental earthworks than was the case with later mound villages.

e. **Methods for processing LiDAR Digital Surface and Terrain Models**

Recently, laser scanner systems have significantly improved in terms of accuracy and spatial resolution, enabling ALS systems to map difficult-to-access areas, such as forests, without causing environmental damage. Laser scanning technology uniquely provides 3D data collection and representation of tree structures, as the laser beam has high penetrability through the canopy, which is not possible with passive satellite images. This capability has been extensively demonstrated in previous studies across various forest types, including the detection of geoglyphs under forests. More specifically, detecting and measuring geoglyphs under tropical forests presents a unique challenge due to the dense canopy that can limit the laser bean penetrability. As a result, careful planning for data acquisition in the research area and further data processing was essential, particularly given the varying forest densities in the area that range from open areas without forest cover to regions with very high tree density. Here, we describe data processing methods used to detect the geoglyph-building Aquiry civilization, their earthworks, and to estimate the pre-colonial population in SW Amazonia.

Full-waveform laser data processing was performed with RiProcess software (RIEGL Gmbh, Austria). The GNSS/IMU navigation data and direct georeferencing were processed using the Applanix/PosPAC MMS 7.0 software. The GNSS/IMU data were integrated in the pre-processing to generate georeferenced laser point cloud (UTM 19 S/ SIRGAS 2000 [ESPG 31979] and UTM 20 S/ SIRGAS 2000 [EPSG:31980]).

The Digital Terrain Model (DTM) and Digital Surface Model (DSM) were generated using LASTools (RapidLasso GmbH, Germany). The digital models processing pipeline included point cloud classification, noise and spike removal, and interpolation to a target resolution of 50 cm for ALS point clouds and 10 cm for UAV-ALS point clouds, which was found to be sufficient for the geoglyph detection. To optimize processing time, the total of 2,254 ALS point cloud tiles of c. 2 km² were processed recursively by dividing each into square subset-tiles (400 m tile size with a 30 m buffer) and utilizing four cores for parallel computing. The buffer size defines an additional area around each subset-tile in all directions, ensuring overlap between adjacent subset-tiles. This overlap prevents data gaps and minimizes preprocessing artifacts. A flag is assigned to the buffer area during tile generation. Similarly, a total of 50 UAV-ALS point cloud tiles were processed with 200 m subset-tile size. The georeferenced point cloud tiles were classified into ground points (Class 2) and non-ground points (Class 0) to enable the generation of both the Digital Terrain Model (DTM) and Digital Surface Model (DSM). During this process, the buffer area was excluded.

After point cloud classification, a Delaunay triangulation can be applied to generate the digital models^67^. More specific regarding DSM processing, the DSM was also created by using the spike-free algorithm^68-69^, which systematically prevents spike formation during TIN construction to accurately represent the uppermost layer of the forest canopy. A spike parameter of 1 m was applied to 99.9% of the point clouds, both for ALS and UAV-ALS point clouds. However, three ALS point cloud tiles (LT03_0056, LT03_0123, and LT09_0041) required customized spike parameters between 1.5 and 2.5, while one ALS point cloud tile (LT03_0093) was removed from the dataset due to its limited coverage in the edge of the research area.

The final DSM and DTM were converted into GeoTIFFs, with interpolated height and hillshade values as pixels, using the BLAST tool in LASTools. The hillshading technique enables a greyscale 3D representation of the terrain surface to be generated considering the relative position of the sun for shading, which highlights the terrain's slope and aspect based on light source direction^67^. The BLAST tool was selected due to its efficient performance when processing billions of LiDAR points from LAS/LAZ files, triangulating them into a seamless TIN, and rasterizing the TIN into a digital raster image. The resulting datasets were then combined into a single raster image using the GDAL library^52^ for further spatial analysis.

f. **Earthwork sensitivity analysis from the Airborne LiDAR point clouds and the upper limit of the detectable earthworks within the acquired LiDAR data**

We evaluated the sensitivity of earthwork detection in our airborne LiDAR point clouds by estimating the required point density for reliable detection within the scanned area. The sensitivity analysis was conducted in four steps:

First, we selected a total of twelve (12) test areas (tiles of approximately 2 km²) with detected geoglyphs both in open (6 areas) and in forest-covered (6 areas) areas. We then randomly downsampled the number of points in each tile, starting from 90% down to 10% of the original spatial resolution in 10% intervals and resulting in nine resampled point clouds for each original point cloud. Additionally, downsampling was performed using smaller intervals, generating two extracted point clouds with 5% and 2.5% of the original resolution. A total of 11 downsampled point cloud versions were created from each original file (132 modelled point cloud images in total) to be used in the sensitivity study.

Second, we computed the Digital Terrain Model (DTM) creation to every downsampled point cloud. The DTMs were generated using LASTools (RapidLasso GmbH, Germany). The digital models processing pipeline included point cloud classification (only ground points are used), noise and spike removal, and interpolation to a target resolution of 50 cm for ALS point clouds, which was found to be sufficient for the earthwork detection. More details are described in the earlier Methods section (Supplementary Note 1e). Hillshaded terrain models were computed similarly for every test tile, aiming to verify each point where the earthworks cannot no longer be visually detected.

Third, we visually assessed the earthwork visibility for every hillshaded terrain model to determine the downsampling level where earthworks were no longer resolvable from the terrain model (Supplementary Figure 1). Based on the visual assessment of the test tiles without forest coverage (on Supplementary Figure 1A), the effective earthwork detection threshold in the Amazonas and Acre region was approximately 0.3 ground points per square metre, corresponding to fewer than 20,000,000 ground points within 2 km². In contrast, Supplementary Figure 1B shows that this threshold is reached at less than 10% of the original resolution when considering ground and non-ground points per square metre acquired at dense forest areas, as the penetration of laser beams is limited. This result is in line with the earlier studies^70-71^, and the experiences obtained in Boreal forests^72-73^.

**Supplementary Figure 1. Comparison of hillshaded digital terrain models (DTMs) of earthworks.** **A.** Open-area earthworks. Hillshaded DTMs derived from the full point cloud (12 ground pts/m²), compared with DTMs generated from progressively downsampled data—10% (1.2 ground pts/m²), 2.5% (0.3 ground pts/m²), and 1% (0.12 ground pts/m²). **B.** Forested-area earthwork. Hillshaded DTMs from the full point cloud (17 ground pts/m²), followed by downsampled versions—50% (8.5 ground pts/m²), 10% (1.7 ground pts/m²), 2.5% (0.4 ground pts/m²), and 1% (0.16 ground pts/m²). Note that under forest cover, the 1% dataset cannot be properly triangulated, resulting in gaps where triangles with edge lengths exceeding 100 m are excluded from DTM computation.

Fourth, based on the previous analysis, the ground point density was systematically checked across all 2,254 tiles using a 3 x 3 m² aggregation window, and all areas with a ground point density lower than the threshold of 0.3 ground points per m² were labelled. The size of the aggregation window was selected based on the typical widths of the edges of the detected earthworks. A uniform 3 × 3 grid was generated, and only the raster grid cells (pixels) within the main area were considered. The main area was delineated using a two-dimensional alpha-shape algorithm applied to each point cloud tile. This procedure was implemented to exclude empty regions that were attributable to the grid configuration itself rather than to limitations in laser scanning penetrability. In total, 46.6% of the 3 x 3 m² raster cells in the scanned area had a ground point density below the effective earthwork detection threshold, corresponding to an area of 2,093.82 km² of the 4,496.98 km² verified. Furthermore, the result shows that the Lábrea region (FUSO 20) with denser forest cover than the Rio Branco region (FUSO 19) had on average 10% less LiDAR penetration to the ground. In the FUSO 20 regions (situated east of the 66W longitude), 54.5% of the 3 x 3 m² raster cells in the scanned area had a ground point density below the effective earthwork detection threshold, while for FUSO 19 (west of the 66W longitude) the percentile was 41.9%.

However, this result only indicates the proportion of the raster cells with ground point density below the detection threshold in the study area. The result does not indicate how these raster cells are spatially aggregated, which can affect the analysis regarding the regions where earthworks could be undetectable due to point cloud density.

Exact estimation of the below-the-threshold raster cells on earthwork detection is challenging. The estimation depends on several factors in addition to the ground points resolution. These factors include the geoglyph area, the earthwork edge height variance, and the ground point density of neighbouring raster cells. Thus, while the number of ground points per square metre provides a useful indication, it alone cannot guarantee whether earthworks can be detected or not in a certain region. For instance, Supplementary Figure 2 illustrates the 'salt-and-pepper' effect that emerges when 3×3 raster cells are classified in binary terms as below threshold (grey) or above threshold (white) for the same tiles as presented on Supplementary Figure 1. Therefore, further analyses were required to understand better the extension and aggregation of regions with less than 0.3 ground points per m². We can clearly see that, even if raster cells with a ground point density under the threshold exist, the distribution of such raster cells and their neighbourhoods can still enable the detection of the earthworks due to their extensive size.

**Supplementary Figure 2**. **Example of earthwork detection in hillshaded DTM images for open (A–B) and forested (C–D) areas:** The corresponding labelled areas shown in panels E–F (open) and G–H (forested). On E to H panels, the white indicates 3 × 3 m^2^ raster cells with the ground point density above the threshold of 0.3 points per square metre, while dark grey represents raster cells that have ground point density below the threshold. The results show that, although low-resolution areas are present within these tiles, the non-uniform point distribution still enables the detection of earthworks.

Therefore, we conducted a subsequent analysis using image processing techniques to aggregate raster cells classified as above or below the threshold. Connected-component labelling was applied with 8-connectivity to group adjacent raster cells into coherent regions, followed by the use of morphological operators to refine region boundaries and remove noise. Supplementary Figure 3 illustrates examples of this aggregation. In this analysis, we considered that only regions larger than 1 hectare (1111 empty raster cells aggregated) composed of raster cells below the threshold would hinder the detection of earthworks. Panel A shows an example of a clear open area that still contains regions where the presence of earthworks remains uncertain at tiles located at Fuso 19. Panel B depicts a mixed open and forested area, in which the uncertain regions are also labelled according to the 1-hectare threshold in the Fuso 20 region.

**Supplementary Figure 3**. **Examples of results from the aggregation of raster cells with the ground point density below the threshold (0.3 pts per square metre) using the labelling connectivity method. A.** Detection of low-resolution regions within a predominantly high-resolution tile in an open area. **B.** Detection of unknown regions within a tile containing both open and forested areas. It is important to note that forested areas are not entirely unknown; rather, these results highlight the specific regions with reduced penetrability that may interfere with earthworks detection.

By estimating only the areas in which the number of aggregated raster cells below the threshold (0.3) exceeds 1 hectare, we conclude that 13.7% of the area can be considered unknown. Penetrability is higher in Fuso 19, with only 11.3% of the area classified as unknown. In contrast, penetrability in Fuso 20 is less effective, with 17.8% of the area remaining unknown, indicating that further studies should place greater focus on this region. The sensitivity analysis result gives an upper limit for the potential areas in the study region that may still have undetected earthworks under the forest cover, based on where the available point density is not sufficient to resolve the underlying earthwork structure. Furthermore, it is clear that geoglyphs measuring one hectare or more, with deep ditches and high embankments, are easier to detect than eroded earthworks with embankments only. Therefore, the impenetrable areas in the Fuso 19 region may fluctuate between 11.3 and 41.9%, and in Fuso 20 between 17.8 and 54.5%, depending on various conditions of the forest and earthwork structures.

**Supplementary Table 1. Radiocarbon dates of earthworks located in the study area. a.** Geoglyph sites. **b.** Mound settlements and roundish embanked enclosures

**a. GEOGLYPH SITES**

**Lab. No.	Pit	Depth (cm)	Material	δ^13^C ‰ VPDB	B.P. date	Calibrated with 2 sigma**

**ACRE**

**Severino Calazans (Acre)**^58^	coordinates **-10.0252 -67.5081**

Ua–59605	19B	134	charcoal	–26.0	8932±37	8250 – 7955 BC

Ua–59604	19A	134	charcoal	–25.9	8849±40	8210 – 7780 BC

Ua–50106	12	150	charcoal	–26.4	8391±59	7585 – 7205 BC

Ua–50102	7F	85	charcoal	–26.5	7050±53	6030 – 5795 BC

Ua–66034	19B	111	charcoal	–25.0	6027±33	5010 – 4800 BC

Ua–66032	19A	120	charcoal	–25.6	5308±32	4250 – 4000 BC

Ua–50105	12	90	charcoal	–27.6	4086±36	2865 – 2490 BC

Ua–37237	3	50	charcoal	–28.2	3990±40	2625 – 2345 BC

Ua–66033	19B	90	charcoal	–26.4	3976±30	2580 – 2350 BC

Ua–58150	13D	232	charcoal	–	3960±109	2870 – 2145 BC

Ua–52903	12	105	charcoal	–27.6	3740±32	2285 – 2030 BC

Ua–59603	19A	85	charcoal	–26.9	3105±31	1440 – 1275 BC

Ua–50103	7D	400–410	charcoal	–28.0	2964±35	1370 – 1045 BC

Ua–37238	5	45	charcoal	–25.3	2915±35	1225 – 1005 BC

ditched enclosure

Ua–59599	20A	120	charcoal	–29.2	2465±31	765 – 415 BC

Ua–59601	20A	145	charcoal	–28.1	2429±31	750 – 400 BC

Ua–59602	20A	156	charcoal	–28.8	2422±31	750 – 400 BC

Ua–59600	20A	133	charcoal	–29.3	2406±32	745 – 395 BC

Ua–37265	6B	50–60	charcoal	–27.8	2275±35	405 – 205 BC

Ua–58153	13C	150	charcoal	–28.3	2147±32	355 – 50 BC

Ua–58151	13D	383	charcoal	–26.6	2078±30	175 BC – AD 10

Ua–66031	19A	23	charcoal	–30.2	2074±30	175 BC – AD 10

Ua–58152	13D	405	charcoal	–27.4	2063±30	165 BC – AD 15

Ua–37264	3	20–30	charcoal	–24.2	2050±35	170 BC – AD 60

Ua–50104	12	60	charcoal	–28.3	1970±35	50 BC – AD 130

Ua–58149	13D	155	charcoal	–27.7	1936±30	AD 15 – 205

Ua–58146	13D	95	charcoal	–26.2	1715±30	AD 250 – 415

**Test pits situated 100–500 meters toward north of the geoglyph of Severino Calazans**^58^

Ua–58155	14	67	charcoal	–26.4	4173±32	2885 – 2630 BC

Ua-58156	14	80	charcoal	–26.1	4035±33	2830 – 2465 BC

Ua–58157	14	97	charcoal	–26.7	3410±31	1875 – 1615 BC

Ua–58159	17	48	charcoal	–27.6	3121±31	1495 –1290 BC

Ua–58160	18	40	charcoal	–28.1	1620±30	AD 405 – 545

Ua–58158	15	60	charcoal	–25.5	1526±30	AD 430 – 605

Ua–58154	14	48	charcoal	–25.4	1512±30	AD 435 – 640

**Severino Batista (Acre)**^58^	**-10.0313 -67.5128**

ditched enclosure

Ua–58147	1	110	charcoal	–27.2	1771±30	AD 215 – 365

Ua–58146	1	95	macrofossil	–26.5	1295±30	AD 655 – 775

**Tequinho (Acre)**^8^	**-9.89774 -67.4235**

Ua–72290*****	16	180–190	charcoal	–25.0	9937±38	9660 – 9290 BC

Ua–48320	9C	90	charcoal	–24.7	4120±42	2875 – 2570 BC

Ua–72287*****	12	90–100	charcoal	–27.2	3710±32	2205 – 1980 BC

Ua–72286*****	12	70–80	charcoal	–28.4	3682±32	2195 – 1955 BC

ditched enclosure

Ua–72289*****	16	130–140	charcoal	–26.8	2328±31	515 – 230 BC

Ua–72293*****	18	320–350	charcoal	–26.1	2089±32	200 BC – AD 5

Ua–72288*****	16	100–110	charcoal	–28.1	2082±31	195 BC – AD 10

Ua–48319	9D	120	charcoal	–27.2	2011±43	150 BC – AD 125

Ua–72284*****	11	80–90	charcoal	–26.8	1995±31	50 BC – AD 120

Ua–48327	9I	210	charcoal	–26.1	1968±33	45 BC – AD 130

Ua–48326	9H	55	charcoal	–27.3	1966±35	50 BC – AD 155

Ua–48321	9A	185	charcoal	–28.0	1964±40	50 BC – AD 205

Ua–48322	9A	130	charcoal	–27.8	1963±39	45 BC – AD 205

Ua–48329	9A	160	charcoal	–26.8	1956±34	45 BC – AD 205

Ua–72285*****	11	120–130	charcoal	–26.8	1956±31	45 BC – AD 205

Ua–48328	9I	150	charcoal	–25.5	1935±33	AD 10 – 210

Ua–50107	9L	70–80	charcoal	–24.4	1910±32	AD 25 – 220

Ua–48323	9A	100	charcoal	–26.5	1879±39	AD 60 – 245

Ua–48325	9H	110	charcoal	–26.1	1874±33	AD 75 – 240

Ua–48324	9H	160	charcoal	–28.3	1841±34	AD 120 – 325

Ua–72291*****	18	40–50	charcoal	–27.5	1569±31	AD 420 – 570

Ua–72283*****	18	210–230	macrofossil	–25.8	1560±31	AD 425 – 575

Ua–72281*****	11	40–50	macrofossil	–25.8	1557±31	AD 425 – 580

Ua–72292*****	18	165	charcoal	–26.8	1525±31	AD 430 – 635

Ua–72282*****	18	90–100	macrofossil	–26.4	1479±31	AD 545 – 645

Ua–50108	18	145	charcoal	–27.2	1476±34	AD 545 – 650

------

*New ^14^C dating

**Eletronorte I:1 (Acre)*	-10.589 -67.9691**

Ua-66022	2	100	charcoal	–25.9	7300±36	6230 – 6075 BC

ditched enclosure

Ua-66021	1	200	charcoal	–27.6	2466±30	765 – 415 BC

Ua-66019	1	140	charcoal	–26.8	2455±30	755 – 410 BC

Ua-66023	2	174	macrofossil	–25.2	2427±30	750 – 400 BC

Ua-66018	1	100	charcoal	–26.5	2419±31	750 – 400 BC

Ua-66020	1	168	charcoal	–25.6	2409±32	745 – 395 BC

Ua-66017	1	70	charcoal	–28.0	2323±30	465 – 230 BC

------

*New ^14^C dating (Supplementary Fig. 4). All samples have been pre-treated according to current recommendations^74^ as a "standard protocol" by the Ångström Laboratory at Uppsala University in Sweden.

**Piçarreira (Acre)*	-10.6565 -68.0945**

Ua-66026	1	180	charcoal	–25.9	8874±41	8235 – 7825 BC

Ua-66025	1	158	charcoal	–26.2	8808±38	8185 – 7725 BC

Ua-66030	4	70	charcoal	–26.0	5619±38	4540 – 4355 BC

ditched enclosure ?

Ua-66028	2	150	charcoal	–26.2	2638±35	900 – 770 BC

ditched enclosure

Ua-66027	2	98	charcoal	–26.0	2095±30	200 BC – AD 5

Ua-66024	1	100	charcoal	–26.3	2069±30	170 BC – AD 10

Ua-66029	2	260	charcoal	–28.6	1859±31	AD 80 – 245

------

*New ^14^C dating (Supplementary Fig. 5). All samples have been pre-treated according to current recommendations^74^ as a "standard protocol" by the Ångström Laboratory at Uppsala University in Sweden.

**Los Angeles (Acre)** ^59^	**-10.7133 -68.1793**

- bottom of wall -	50	-	-	3078**	1495 – 1130 BC
- 12	30	-	-	2825±95	1260 – 805 BC

ditched enclosure?

- exterior	-	-	-	-	2490**	785 – 415 BC
- ditched enclosure
- wall	-	-	-	-	2256**	415 – 150 BC
- ditch	-	180-190	-	-	2200**	395 – 60 BC
- ditch	4	180-190	-	-	2050**	345 BC – AD 120
- -	10-20	-	-	1869**	AD 15 – 335
- -	30-40	-	-	1728**	AD 205 – 535
- 10	25-30	-	-	1410**	AD 540 – 775
- near wall -	10-40/45	-	-	1228**	AD 660 – 960

------

**Estimated values ±60 used for calibration.

**Fazenda Colorada (Acre)**^9^	**-9.87786 -67.5356**

ditched enclosure

Ua-37235	7	150-160	charcoal	–24.1	1865±65	AD 5 – 345

Ua-37256	12	90	charcoal	–25.5	1820±30	AD 130 – 330

Ua-37567	9	218	charcoal	–26.7	1775±35	AD 210 – 380

Ua-37236	10	70–80	charcoal	–23.2	1340±35	AD 640 – 775

Ua-37255	10	67	charcoal	–28.8	1275±30	AD 660 – 825

**Jacó Sá (Acre)**^9^	**-9.96378 -67.4978** (IVB - 8) and **-9.9608 -67.4983** (1)

ditched enclosures (N.=2)

Ua-37259	IVB	10-20	charcoal	–25.4	1485±35	AD 535 – 650

Ua-37258	8	80-90	charcoal	–27.6	1205±30	AD 700 – 945

Ua-37257	1	47	charcoal	–27.8	1195±30	AD 705 – 950

**JK (Acre)**^60^	**-9.73209 -67.0616**

ditched enclosure

Beta-294310	5	170	charcoal	–26.5	1830±30	AD 125 – 320

Beta-294309	5	158	charcoal	–26.6	1710±30	AD 250 – 420

**Ramal do Capatará (Acre)**^60^	**-10.3656 -67.7189**

Beta-288234	ditch	79	charcoal	–25.9	3310±40	1730 – 1500 BC

ditched enclosure

Beta-288233	ditch	170	charcoal	–25.5	1990±30	50 BC – AD 120

Beta-288232	ditch	130-140	charcoal	–27.9	1850±40	AD 80 – 325

**Fazenda Iquiri II (Acre)**^10^	**-10.0713 -67.4103**

ditched enclosure

Ua-49945	13	30	charcoal	–26.5	1741±34	AD 240 – 405

**Fazenda Atlântica (Acre)**^62^	**-10.0777 -67.5754** (7) and **-10.0793 -67.5728** (4-5)

ditched enclosures (N.=2)

Ua-37253	7	55	charcoal	–27.2	2110±35	345 – 1 BC

Ua-37251	4	40	charcoal	–30.6	1905±35	AD 25 – 225

Ua-37252	5	110	charcoal	–25.6	1855±30	AD 85 – 245

**Balneário Quinauá (Acre)**^62^	**-10.0581 -67.6176** (3-6)	and **-10.0549 -67.6167** (11-16)

ditched enclosures (N.=2)

Ua-37261	6	50	charcoal	–27.5	1760±35	AD 230 – 405

Ua-37260	3	160-170	soot on sherd	–13.1	1585±30	AD 415 – 550

Ua-37262	11	42	charcoal	–26.9	1570±35	AD 420 – 570

Ua-37263	16	230	charcoal	–26.9	1565±35	AD 420 – 575

**AMAZONAS**

**Cruzeirinho (Amazonas)**^8^	**-8.84358 -67.259**

ditched enclosure

Ua-48333	U2	20-30	charcoal	–29.1	1846±40	AD 80 – 325

Ua-48331	U2	80	charcoal	–30.0	1813±40	AD 125 – 345

Ua-48330	U2	105	charcoal	–28.3	1781±34	AD 210 – 375

Ua-48332	U2	60	charcoal	–26.1	1705±42	AD 245 – 425

**BOLIVIA**

**Tumichucua (Bolivia)**^75^	**-11.1455 -66.1601**

ditched enclosure

Ua-24932	6	60	charcoal	–27.5	2045±65	345 BC – AD 125

Hela-702	1	40	charcoal	–26.5	1905±40	AD 25 – 235

**Las Palmeras (Bolivia)**^75^	**-10.9883 -66.0119**

ditched enclosure

Ua-24930	3	60	charcoal	–25.8	1850±40	AD 80 – 325

**b. MOUND SETTLEMENTS AND ROUNDISH EMBANKED ENCLOSURES**

**Lab. No.	Pit	Depth (cm)	Material	δ^13^C ‰ VPDB	B.P. date	Calibrated with 2 sigma**

**ACRE**

**Fazenda Colorada (Acre)**^76^	**-9.87786 -67.5356**

Hela-616	mound -	25	charcoal	–26.6	750±35	AD 1220 – 1300

**Fazenda Iquiri II (Acre)**^10^	**-10.0713 -67.4103**

embankment near mounds

Ua-49946	13	50	charcoal	–27.6	706±31	AD 1260 – 1390

mounds

Ua-49944	7	140	charcoal	–27.0	476±30	AD 1405 – 1460

Ua-49943	3	27	charcoal	–26.9	285±31	AD 1495 – 1795

**Coqueiral (Acre)**^10^	**-9.90216 -67.3503**

mounds

Ua-48334	4	135	charcoal	–27.6	534±40	AD 1315 – 1450

Ua-48335	4	100	charcoal	–26.4	512±39	AD 1320 – 1455

Ua-48336	3	60	charcoal	–27.8	404±39	AD 1430 – 1635

**Três Vertentes (Acre)*	-9.74145 -67.0699**

mounds on the south-eastern corner of a ditched enclosure

Ua-50109	1	45	charcoal	–26.7	264±31	AD 1505 – 1800

Ua-50110	1	50-60	charcoal	–27.3	241±32	AD 1520 – …

------

*New ^14^C dating. All samples have been pretreated according to current recommendations^74^ as a "standard protocol" by the Ångström Laboratory at Uppsala University in Sweden.

**Sol de Maio (Acre)**^77^	**-10.0028 -67.4085**

mounds

Ua-44351	2	40-50	soot on sherd	–27.7	644±32	AD 1280 – 1400

Ua-44353	4	90	charcoal	–29.3	644±31	AD 1280 – 1400

Ua-44350	1	71	charcoal	–28.7	612±32	AD 1295 – 1405

Ua-44352	3	63	macrofossil	–26.3	516±31	AD 1325 – 1450

**Sol de Campinas (Acre)**^77-78^	**-10.0579 -67.3091**

mounds?

Beta-408406 11	190-210	charcoal	–27.2	1000±30	AD 990 – 1155

mounds

Beta-408407 11	170-190	charcoal	–25.7	740±30	AD 1225 – 1300

Beta-408409 11	150	charcoal	–24.8	660±30	AD 1275 – 1395

Beta-408410 11	130	charcoa	l	–28.6	590±30	AD 1300 – 1415

Beta-408412 11	50	charcoal	–29.2	510±30	AD 1325 – 1450

**Fonte Boa: Estrutura II (Acre)**^77^	**-10.0752 -67.3193**

mounds

Ua-59659	-	120	charcoal	–26.4	576±27	AD 1305 – 1420

**Caboquinho (Acre)**^63^	**-9.81029 -67.1737**

mounds

Beta-500928	1	145	charcoal	-	770±30	AD 1220 – 1285

Beta-500922	1	78	charcoal	-	740±30	AD 1225 – 1300

Beta-500927	1	135	charcoal	-	660±30	AD 1275 – 1395

Beta-500920	1	48	charcoal	-	640±30	AD 1285 – 1400

Beta-500925	1	ca.115	charcoal	-	580±30	AD 1300 – 1420

Beta-500921	1	58	charcoal	-	540±30	AD 1320 – 1440

Beta-500923	1	98	charcoal	-	510±30	AD 1325 – 1450

Beta-500926	1	125	charcoal	-	490±30	AD 1400 – 1455

Beta-500924	1	ca.108	charcoal	-	470±30	AD 1405 – 1460

**Fazenda Tocantins (Acre)**^63^	**-9.89541 -67.1794**

mounds

Beta-500911	1	ca. 60	charcoal	-	620±30	AD 1295 – 1400

**Boa Esperança (Acre)** ^63^	**-9.84025 -67.1695**

mounds

Beta-500913	2	ca. 65	charcoal	-	540±30	AD 1320 – 1440

**Dois Circulos: Estrutura IV (Acre)**^63^	**-10.2084 -67.6552**

Mounds

Beta-500914	1	ca. 50	charcoal	-	610±30	AD 1295 – 1405

**Dois Circulos: Estrutura V (Acre)**^63^	**-10.205 -67.6507**

mounds

Beta-500919	6	ca. 40	charcoal	-	570±30	AD 1305 – 1425

Beta-500918	5	ca. 30	charcoal	-	520±30	AD 1325 – 1445

Beta-500917	4	ca. 35	charcoal	-	500±30	AD 1395 – 1450

**Campo Esperança (Acre)**^10^	**-10.4384 -67.7062**

roundish embanked enclosure

Ua-49941	11	80	charcoal	–25.2	544±31	AD 1320 – 1440

**Tênue (Acre)**^10^	**-10.4573 -67.6801**

roundish embanked enclosure

Ua-49942	5	90	charcoal	–30.2	204±31	AD 1640 – …

**BOLIVIA**

**Estancia Giese (Bolivia)**^75^	**-10.9761 -66.0177**

Hela-707	1	80	charcoal	-	10355±80	10650 – 9930 BC

embanked roundish enclosure

Hela-708	4	40	charcoal	–24.4	1815±45	AD 125 – 340

Hela-709	6	60	charcoal	–24.4	1695±40	AD 245 – 435

**Las Palmeras (Bolivia)**^75^	**-10.9884 -66.0123**

mounds

Ua-24076	1	96	charcoal	–26.1	285±35	AD 1490 – 1795

**El Circulo (Bolivia)**^75^	**-11.0361 -66.1282**

Hela-570	2	50-60	ceramic temper –23.0	1790±75	AD 75 – 420

circular embanked enclosure with attached mounds

Poz-9426	7	40-50	soot on sherd	-	715±30	AD 1230 – 1390

Poz-9428	10	100-110	macrofossil	-	685±30	AD 1270 – 1390

Poz-9523	7	20	charcoal	-	680±30	AD 1275 – 1390

Poz-9427	8	80	charcoal	-	660±30	AD 1275 – 1395

Poz-9524	6	53	charcoal	-	650±30	AD 1280 – 1395

Poz-9429	10	120-130	soot on sherd	-	645±30	AD 1280 – 1400

Hela-4585	1	85	charcoal	–26.7	600±60	AD 1280 – 1425

**Supplementary Figure 4.** **Archaeological site of Eletronorte I:1. A**. A map of Eletronorte I:1. **B.** The northern profile of Pit 1, made into a small mound associated with the ditch.

**Supplementary Figure 5.** **Archaeological site of Piçarreira. A.** A map of Piçarreira. **B.** Western profile of the Pit 4 made into the interior plaza of the geoglyph.

**Supplementary Table 2. List of earthworks discovered in the ALS data.** Coordinates are presented in decimal degrees, but discoveries in Indigenous territories are not included. Aquiry indicates whether the earthwork meets the definition of an Aquiry structure: yes (y), no (n), and accuracy indicates the clarity of outline recognition in size measurements, classified as good (g) or somewhat good (s)^3^. * refers earthwork that only partially (<50%) intersect the LiDAR swath.

| No | ^o^S | ^o^W | Name in our data | Aquiry | Size (ha) | Accuracy |
| --- | --- | --- | --- | --- | --- | --- |
| 1 | -9.692 | -67.276 | acre40 | y | 1.51 | s |
| 2 | -9.828 | -67.738 | acrq31 | y | 2.26 | g |
| 3 | -9.745 | -67.060 | acrs42 | y | 4.91 | g |
| 4 | -9.849 | -67.901 | Aeroporto - Estructura I | y | 8.75 | g |
| 5 | -9.844 | -67.900 | Aeroporto - Estructura II | n | 3.21 | g |
| *6 | -9.175 | -66.939 | amae16 - Estructura III | y | 1.31 | s |
| 7 | -9.300 | -66.508 | amag4 | y | 0.84 | g |
| *8 | -9.273 | -66.487 | amao9 | y | 0.83 | g |
| 9 | -9.268 | -66.478 | amap9 - Estructura I | y | 5.53 | g |
| 10 | -9.306 | -66.524 | amaq22 | y | 1.42 | g |
| 11 | -9.344 | -66.567 | amaq36 | y | 3.58 | g |
| 12 | -9.160 | -67.496 | amas10 | y | 0.73 | g |
| 13 | -9.582 | -67.282 | amas4 - Estructura II | y | 3.23 | g |
| 14 | -9.427 | -67.246 | amas8 | y | 2.90 | g |
| 15 | -9.987 | -67.499 | Califórnia - Estrutura I | y | 2.89 | g |
| 16 | -9.986 | -67.498 | Califórnia - Estrutura II | y | 2.79 | g |
| 17 | -9.204 | -66.973 | endig1 | y | 1.40 | g |
| 18 | -9.840 | -67.170 | Fazenda Boa Esperança - Estructura I | n | 0.97 | g |
| 19 | -9.840 | -67.169 | Fazenda Boa Esperança - Estructura II | n | 0.51 | g |
| 20 | -9.236 | -67.010 | G.Ch. 4 - 89 | y | 4.22 | g |
| 21 | -8.757 | -67.307 | itaur | y | 1.53 | g |
| 22 | -8.777 | -65.880 | Lid24_L01_101a | y | 1.55 | s |
| 23 | -8.795 | -65.890 | Lid24_L01_102a | n | 4.05 | s |
| 24 | -8.912 | -66.034 | Lid24_L01_112a | y | 2.36 | s |
| 25 | -8.994 | -66.141 | Lid24_L01_119a | y | 3.80 | g |
| 26 | -9.037 | -66.192 | Lid24_L01_123a | y | 4.41 | g |
| 27 | -9.107 | -66.272 | Lid24_L01_129a | n | 0.80 | s |
| 28 | -9.121 | -66.291 | Lid24_L01_130a | y | 2.33 | s |
| 29 | -9.179 | -66.367 | Lid24_L01_135a | y | 1.97 | g |
| 30 | -9.199 | -66.381 | Lid24_L01_137a | y | 1.46 | s |
| 31 | -9.250 | -66.455 | Lid24_L01_142a | n | 3.62 | s |
| 32 | -9.271 | -66.479 | Lid24_L01_143a | n | 12.80 | s |
| 33 | -9.268 | -66.471 | Lid24_L01_143b | y | 3.31 | g |
| *34 | -9.264 | -66.476 | Lid24_L01_143c | y | 2.60 | s |
| 35 | -9.282 | -66.486 | Lid24_L01_144a | n | 0.51 | g |
| 36 | -9.289 | -66.502 | Lid24_L01_145a | n | 5.80 | s |
| 37 | -9.290 | -66.498 | Lid24_L01_145b | n | 2.43 | g |
| 38 | -9.290 | -66.501 | Lid24_L01_145c | y | 2.54 | s |
| 39 | -9.312 | -66.523 | Lid24_L01_147b | y | 0.50 | s |
| 40 | -9.322 | -66.532 | Lid24_L01_147c | y | 0.43 | g |
| 41 | -9.319 | -66.536 | Lid24_L01_147d | n | 5.52 | g |
| 42 | -9.324 | -66.537 | Lid24_L01_148b | y | 0.27 | g |
| 43 | -9.322 | -66.540 | Lid24_L01_148d | n | 6.17 | g |
| 44 | -9.322 | -66.545 | Lid24_L01_148e | y | 6.58 | s |
| 45 | -9.335 | -66.556 | Lid24_L01_149b | n | 0.45 | g |
| 46 | -9.340 | -66.558 | Lid24_L01_149e | n | 1.26 | s |
| *47 | -9.340 | -66.568 | Lid24_L01_150a | y | 7.17 | g |
| 48 | -9.348 | -66.566 | Lid24_L01_150c | y | 2.43 | g |
| 49 | -9.346 | -66.569 | Lid24_L01_150d | y | 3.58 | g |
| 50 | -9.347 | -66.565 | Lid24_L01_150e | n | 0.56 | g |
| 51 | -9.348 | -66.573 | Lid24_L01_150g | n | 0.50 | g |
| 52 | -9.348 | -66.575 | Lid24_L01_150h | y | 1.96 | g |
| 53 | -9.350 | -66.576 | Lid24_L01_150j | n | 0.84 | g |
| 54 | -9.358 | -66.575 | Lid24_L01_151a | n | 4.67 | s |
| 55 | -9.360 | -66.583 | Lid24_L01_151b | n | 0.53 | g |
| 56 | -9.358 | -66.576 | Lid24_L01_151c | n | 0.46 | g |
| 57 | -9.386 | -66.620 | Lid24_L01_153a | n | 4.21 | s |
| 58 | -9.390 | -66.621 | Lid24_L01_154a | n | 0.43 | g |
| 59 | -9.391 | -66.626 | Lid24_L01_154b | n | 0.38 | s |
| 60 | -9.392 | -66.624 | Lid24_L01_154e | y | 2.25 | g |
| 61 | -9.455 | -66.703 | Lid24_L01_159a | n | 1.07 | g |
| 62 | -9.455 | -66.700 | Lid24_L01_159b | n | 0.72 | s |
| 63 | -9.456 | -66.705 | Lid24_L01_159c | y | 1.74 | g |
| 64 | -9.462 | -66.708 | Lid24_L01_160a | n | 1.80 | g |
| 65 | -9.485 | -66.731 | Lid24_L01_162a | y | 6.40 | s |
| 66 | -9.485 | -66.735 | Lid24_L01_162b | y | 1.16 | s |
| 67 | -9.483 | -66.733 | Lid24_L01_162c | n | 0.82 | s |
| 68 | -9.517 | -66.777 | Lid24_L01_165a | y | 3.64 | g |
| 69 | -9.520 | -66.778 | Lid24_L01_165b | y | 2.68 | g |
| 70 | -9.517 | -66.782 | Lid24_L01_165c | n | 0.69 | s |
| 71 | -9.534 | -66.795 | Lid24_L01_166a | y | 0.54 | g |
| 72 | -9.592 | -66.866 | Lid24_L01_171a | y | 3.81 | g |
| 73 | -9.598 | -66.869 | Lid24_L01_171b | y | 2.12 | s |
| 74 | -9.704 | -67.004 | Lid24_L01_181a | n | 4.73 | s |
| 75 | -9.715 | -67.018 | Lid24_L01_182a | y | 2.99 | g |
| 76 | -9.743 | -67.049 | Lid24_L01_184a | y | 3.33 | g |
| 77 | -9.749 | -67.064 | Lid24_L01_185c | y | 3.15 | g |
| 78 | -9.749 | -67.062 | Lid24_L01_185d | y | 3.17 | g |
| 79 | -9.752 | -67.071 | Lid24_L01_185e | y | 7.61 | s |
| 80 | -9.862 | -67.206 | Lid24_L01_195a | y | 0.63 | g |
| 81 | -9.865 | -67.208 | Lid24_L01_195b | y | 0.93 | g |
| 82 | -9.960 | -67.323 | Lid24_L01_203a | y | 2.66 | g |
| 83 | -9.960 | -67.319 | Lid24_L01_203b | n | 0.16 | g |
| 84 | -9.962 | -67.322 | Lid24_L01_203c | n | 1.55 | g |
| 85 | -9.962 | -67.320 | Lid24_L01_203d | y | 1.61 | g |
| 86 | -9.962 | -67.320 | Lid24_L01_203e | y | 3.87 | g |
| 87 | -9.963 | -67.319 | Lid24_L01_203f | y | 0.84 | g |
| 88 | -9.961 | -67.317 | Lid24_L01_203g | n | 1.10 | g |
| 89 | -9.965 | -67.319 | Lid24_L01_203h | y | 5.10 | g |
| 90 | -9.963 | -67.318 | Lid24_L01_203i | y | 1.06 | g |
| 91 | -9.959 | -67.324 | Lid24_L01_203j | n | 0.62 | s |
| 92 | -9.986 | -67.349 | Lid24_L01_205a | n | 2.47 | g |
| 93 | -10.024 | -67.398 | Lid24_L01_209a | y | 0.49 | g |
| 94 | -10.024 | -67.397 | Lid24_L01_209b | n | 0.39 | s |
| 95 | -10.133 | -67.529 | Lid24_L01_218a | y | 0.98 | s |
| 96 | -10.172 | -67.578 | Lid24_L01_222a | y | 0.29 | g |
| 97 | -10.173 | -67.578 | Lid24_L01_222b | y | 0.22 | g |
| 98 | -10.181 | -67.594 | Lid24_L01_223a | n | 1.01 | g |
| 99 | -7.661 | -64.521 | Lid24_L01_3a | n | 3.88 | s |
| 100 | -8.121 | -65.072 | Lid24_L01_43a | y | 3.48 | s |
| 101 | -8.143 | -65.105 | Lid24_L01_45a | n | 4.32 | g |
| 102 | -8.832 | -66.077 | Lid24_L02_114a | y | 2.17 | s |
| 103 | -8.800 | -66.040 | Lid24_L02_116a | n | 10.19 | s |
| 104 | -9.994 | -67.502 | Lid24_L02_12a | y | 0.35 | g |
| 105 | -9.984 | -67.495 | Lid24_L02_13c | y | 5.26 | s |
| 106 | -9.960 | -67.468 | Lid24_L02_15a | n | 0.88 | g |
| 107 | -9.958 | -67.467 | Lid24_L02_15b | n | 2.19 | s |
| 108 | -9.962 | -67.465 | Lid24_L02_15c | n | 1.13 | g |
| 109 | -9.948 | -67.443 | Lid24_L02_16a | n | 1.05 | g |
| 110 | -8.071 | -65.159 | Lid24_L02_179a | y | 0.93 | s |
| 111 | -9.875 | -67.359 | Lid24_L02_23a | y | 0.56 | s |
| 112 | -9.818 | -67.292 | Lid24_L02_27a | y | 0.85 | g |
| 113 | -9.702 | -67.148 | Lid24_L02_38a | y | 3.10 | g |
| 114 | -9.651 | -67.081 | Lid24_L02_42a | y | 2.41 | g |
| 115 | -9.623 | -67.054 | Lid24_L02_44a | y | 1.93 | g |
| 116 | -9.583 | -66.999 | Lid24_L02_48a | y | 5.46 | g |
| 117 | -9.573 | -66.991 | Lid24_L02_49a | y | 3.46 | s |
| 118 | -9.567 | -66.979 | Lid24_L02_49b | n | 5.11 | g |
| 119 | -9.565 | -66.978 | Lid24_L02_50a | y | 0.70 | g |
| 120 | -9.560 | -66.976 | Lid24_L02_50b | y | 1.55 | s |
| *121 | -9.541 | -66.942 | Lid24_L02_52a | y | 2.81 | s |
| 122 | -9.514 | -66.915 | Lid24_L02_54a | n | 5.75 | g |
| 123 | -9.509 | -66.913 | Lid24_L02_54b | n | 5.12 | s |
| 124 | -9.491 | -66.894 | Lid24_L02_56a | y | 5.46 | s |
| 125 | -9.486 | -66.882 | Lid24_L02_56b | n | 3.34 | s |
| 126 | -9.469 | -66.864 | Lid24_L02_58a | y | 4.27 | s |
| 127 | -9.455 | -66.841 | Lid24_L02_59a | y | 8.53 | g |
| 128 | -9.454 | -66.839 | Lid24_L02_59b | y | 1.46 | g |
| 129 | -9.446 | -66.837 | Lid24_L02_60a | y | 1.11 | g |
| 130 | -9.415 | -66.793 | Lid24_L02_63a | y | 3.15 | g |
| 131 | -9.381 | -66.759 | Lid24_L02_65a | n | 6.82 | s |
| 132 | -10.058 | -67.589 | Lid24_L02_6a | n | 4.42 | s |
| 133 | -9.330 | -66.690 | Lid24_L02_70a | y | 7.51 | g |
| 134 | -9.308 | -66.661 | Lid24_L02_72a | n | 7.16 | g |
| 135 | -9.276 | -66.621 | Lid24_L02_75a | y | 9.87 | g |
| 136 | -9.274 | -66.619 | Lid24_L02_75b | y | 8.22 | g |
| 137 | -9.266 | -66.612 | Lid24_L02_76a | n | 4.81 | g |
| 138 | -9.252 | -66.591 | Lid24_L02_77a | n | 5.69 | s |
| 139 | -9.242 | -66.580 | Lid24_L02_78a | n | 12.51 | s |
| 140 | -9.237 | -66.576 | Lid24_L02_78b | y | 8.41 | s |
| 141 | -10.054 | -67.578 | Lid24_L02_7a | y | 0.37 | g |
| 142 | -10.051 | -67.579 | Lid24_L02_7b | n | 4.62 | s |
| 143 | -10.054 | -67.578 | Lid24_L02_7c | y | 0.25 | s |
| 144 | -9.214 | -66.548 | Lid24_L02_80a | n | 6.77 | s |
| 145 | -9.150 | -66.477 | Lid24_L02_85a | y | 5.28 | s |
| 146 | -9.138 | -66.454 | Lid24_L02_87a | y | 1.80 | g |
| 147 | -10.039 | -67.563 | Lid24_L02_8a | n | 1.07 | g |
| 148 | -8.869 | -66.276 | Lid24_L03_104a | y | 2.48 | s |
| 149 | -9.952 | -67.596 | Lid24_L03_10a | y | 1.59 | g |
| 150 | -8.520 | -65.845 | Lid24_L03_134a | y | 0.65 | s |
| 151 | -9.914 | -67.544 | Lid24_L03_13a | y | 0.42 | g |
| 152 | -9.914 | -67.544 | Lid24_L03_13b | y | 0.42 | g |
| 153 | -8.314 | -65.597 | Lid24_L03_152a | y | 2.31 | g |
| 154 | -7.890 | -65.082 | Lid24_L03_189a | n | 1.88 | g |
| 155 | -7.885 | -65.077 | Lid24_L03_189b | n | 2.32 | g |
| 156 | -9.733 | -67.332 | Lid24_L03_29a | y | 3.30 | s |
| 157 | -9.696 | -67.288 | Lid24_L03_32a | y | 1.08 | s |
| 158 | -9.697 | -67.287 | Lid24_L03_32b | y | 0.29 | g |
| 159 | -9.697 | -67.285 | Lid24_L03_32c | n | 2.52 | s |
| 160 | -9.694 | -67.284 | Lid24_L03_32d | y | 2.25 | g |
| 161 | -9.693 | -67.281 | Lid24_L03_32e | y | 0.93 | s |
| 162 | -9.694 | -67.282 | Lid24_L03_32f | n | 0.23 | s |
| *163 | -9.696 | -67.290 | Lid24_L03_32g | y | 2.48 | g |
| 164 | -9.693 | -67.280 | Lid24_L03_32h | n | 0.17 | g |
| 165 | -9.693 | -67.282 | Lid24_L03_32i | n | 0.46 | g |
| 166 | -9.691 | -67.274 | Lid24_L03_33a | y | 0.37 | s |
| 167 | -9.690 | -67.274 | Lid24_L03_33b | n | 0.20 | s |
| 168 | -9.690 | -67.273 | Lid24_L03_33c | y | 3.18 | s |
| 169 | -9.690 | -67.273 | Lid24_L03_33d | n | 0.32 | s |
| 170 | -9.689 | -67.270 | Lid24_L03_33f | n | 0.37 | s |
| 171 | -9.680 | -67.267 | Lid24_L03_33g | n | 1.49 | s |
| 172 | -9.657 | -67.235 | Lid24_L03_35a | n | 5.08 | g |
| 173 | -9.661 | -67.235 | Lid24_L03_35b | y | 1.43 | s |
| 174 | -9.651 | -67.230 | Lid24_L03_36a | y | 4.75 | g |
| 175 | -9.653 | -67.231 | Lid24_L03_36b | y | 3.18 | g |
| 176 | -9.641 | -67.214 | Lid24_L03_37a | n | 1.17 | s |
| 177 | -9.555 | -67.111 | Lid24_L03_44a | y | 6.97 | g |
| 178 | -9.555 | -67.112 | Lid24_L03_44b | y | 1.42 | s |
| 179 | -9.556 | -67.108 | Lid24_L03_44c | y | 2.73 | s |
| 180 | -9.552 | -67.104 | Lid24_L03_45a | y | 3.18 | g |
| 181 | -9.551 | -67.107 | Lid24_L03_45b | n | 0.64 | s |
| 182 | -9.550 | -67.097 | Lid24_L03_45c | y | 4.97 | g |
| 183 | -9.550 | -67.107 | Lid24_L03_45d | n | 0.63 | s |
| 184 | -9.545 | -67.091 | Lid24_L03_45e | y | 2.69 | s |
| 185 | -9.518 | -67.067 | Lid24_L03_47a | n | 3.89 | s |
| 186 | -9.514 | -67.063 | Lid24_L03_48a | y | 1.38 | g |
| 187 | -9.500 | -67.047 | Lid24_L03_49a | y | 0.75 | s |
| 188 | -9.501 | -67.048 | Lid24_L03_49b | n | 2.17 | g |
| 189 | -9.504 | -67.053 | Lid24_L03_49c | n | 1.06 | s |
| 190 | -9.457 | -66.982 | Lid24_L03_53a | y | 1.56 | s |
| 191 | -9.325 | -66.828 | Lid24_L03_64a | y | 1.77 | s |
| 192 | -9.325 | -66.827 | Lid24_L03_64b | y | 0.87 | s |
| 193 | -9.321 | -66.822 | Lid24_L03_65a | y | 0.89 | s |
| 194 | -9.300 | -66.796 | Lid24_L03_67a | y | 1.95 | s |
| 195 | -9.298 | -66.796 | Lid24_L03_67b | y | 4.47 | g |
| 196 | -9.272 | -66.767 | Lid24_L03_69a | y | 3.15 | s |
| 197 | -9.086 | -66.529 | Lid24_L03_85a | y | 2.01 | s |
| 198 | -9.010 | -66.447 | Lid24_L03_92a | y | 2.56 | g |
| 199 | -9.007 | -66.443 | Lid24_L03_92b | y | 2.01 | g |
| 200 | -9.003 | -66.436 | Lid24_L03_92c | y | 1.54 | g |
| 201 | -9.012 | -66.442 | Lid24_L03_92d | y | 2.27 | s |
| 202 | -8.828 | -66.367 | Lid24_L04_101a | y | 6.84 | g |
| 203 | -8.806 | -66.335 | Lid24_L04_103a | y | 3.08 | s |
| 204 | -8.628 | -66.121 | Lid24_L04_119a | y | 0.96 | g |
| 205 | -9.857 | -67.628 | Lid24_L04_12a | y | 2.36 | s |
| 206 | -8.167 | -65.559 | Lid24_L04_159a | y | 0.67 | s |
| 207 | -9.796 | -67.549 | Lid24_L04_17a | n | 0.59 | g |
| 208 | -9.797 | -67.545 | Lid24_L04_17b | n | 0.61 | s |
| 209 | -9.727 | -67.462 | Lid24_L04_23a | y | 0.62 | g |
| 210 | -9.728 | -67.462 | Lid24_L04_23b | n | 4.01 | g |
| 211 | -9.727 | -67.458 | Lid24_L04_23c | n | 4.67 | s |
| *212 | -9.719 | -67.447 | Lid24_L04_24a | y | 2.82 | g |
| 213 | -9.692 | -67.414 | Lid24_L04_27a | y | 5.71 | s |
| 214 | -9.668 | -67.398 | Lid24_L04_28a | n | 14.45 | s |
| 215 | -9.670 | -67.397 | Lid24_L04_28b | n | 1.39 | g |
| 216 | -9.671 | -67.398 | Lid24_L04_28c | y | 0.61 | g |
| 217 | -9.672 | -67.394 | Lid24_L04_28d | y | 0.58 | s |
| 218 | -9.664 | -67.392 | Lid24_L04_28e | y | 0.55 | s |
| 219 | -9.668 | -67.393 | Lid24_L04_28f | y | 0.28 | s |
| 220 | -9.668 | -67.394 | Lid24_L04_28g | n | 0.44 | s |
| 221 | -9.670 | -67.394 | Lid24_L04_28h | n | 3.01 | s |
| 222 | -9.675 | -67.394 | Lid24_L04_28i | n | 1.27 | s |
| 223 | -9.676 | -67.394 | Lid24_L04_28j | n | 1.10 | s |
| 224 | -9.663 | -67.383 | Lid24_L04_29a | y | 1.69 | s |
| 225 | -9.656 | -67.381 | Lid24_L04_29b | y | 0.95 | s |
| 226 | -9.648 | -67.362 | Lid24_L04_30a | y | 9.28 | s |
| 227 | -9.643 | -67.362 | Lid24_L04_30b | y | 1.15 | s |
| 228 | -9.631 | -67.351 | Lid24_L04_31a | y | 1.77 | s |
| 229 | -9.643 | -67.361 | Lid24_L04_31b | y | 3.45 | s |
| 230 | -9.587 | -67.297 | Lid24_L04_35a | y | 0.76 | g |
| 231 | -9.589 | -67.292 | Lid24_L04_35b | y | 0.66 | g |
| 232 | -9.572 | -67.272 | Lid24_L04_37a | y | 0.55 | g |
| 233 | -9.573 | -67.271 | Lid24_L04_37b | y | 3.28 | s |
| 234 | -9.543 | -67.232 | Lid24_L04_39a | y | 5.43 | g |
| 235 | -9.525 | -67.213 | Lid24_L04_41a | y | 1.20 | g |
| 236 | -9.462 | -67.133 | Lid24_L04_46a | y | 0.24 | g |
| 237 | -9.422 | -67.082 | Lid24_L04_50a | y | 2.12 | s |
| 238 | -9.411 | -67.079 | Lid24_L04_51a | y | 1.70 | g |
| 239 | -9.394 | -67.051 | Lid24_L04_52a | y | 0.51 | s |
| 240 | -9.355 | -67.002 | Lid24_L04_56a | y | 11.45 | g |
| 241 | -9.353 | -67.000 | Lid24_L04_56b | y | 7.76 | g |
| 242 | -9.346 | -66.992 | Lid24_L04_57a | y | 4.97 | g |
| 243 | -9.320 | -66.960 | Lid24_L04_59a | y | 7.20 | g |
| 244 | -9.321 | -66.960 | Lid24_L04_59b | y | 2.34 | g |
| 245 | -9.313 | -66.952 | Lid24_L04_59c | y | 4.96 | s |
| 246 | -9.315 | -66.952 | Lid24_L04_59d | y | 2.75 | g |
| 247 | -9.308 | -66.955 | Lid24_L04_59e | y | 6.37 | s |
| 248 | -9.321 | -66.967 | Lid24_L04_59f | y | 0.60 | g |
| 249 | -9.300 | -66.943 | Lid24_L04_60b | y | 1.69 | g |
| 250 | -9.270 | -66.907 | Lid24_L04_63a | y | 6.42 | s |
| 251 | -9.254 | -66.881 | Lid24_L04_65a | y | 6.52 | g |
| 252 | -9.184 | -66.802 | Lid24_L04_70a | y | 4.73 | s |
| 253 | -9.185 | -66.796 | Lid24_L04_71a | y | 1.59 | g |
| 254 | -9.107 | -66.703 | Lid24_L04_77a | y | 2.59 | g |
| 255 | -9.099 | -66.701 | Lid24_L04_78a | y | 0.60 | s |
| 256 | -8.710 | -66.366 | Lid24_L05_106a | y | 7.07 | g |
| 257 | -8.705 | -66.359 | Lid24_L05_106b | y | 1.07 | g |
| 258 | -8.609 | -66.243 | Lid24_L05_114a | y | 1.74 | s |
| 259 | -8.060 | -65.566 | Lid24_L05_162a | n | 15.59 | s |
| 260 | -8.051 | -65.560 | Lid24_L05_163a | y | 1.02 | g |
| 261 | -9.741 | -67.626 | Lid24_L05_16a | y | 1.23 | s |
| 262 | -7.633 | -65.053 | Lid24_L05_199a | n | 4.35 | g |
| 263 | -7.638 | -65.057 | Lid24_L05_199b | n | 9.38 | g |
| 264 | -7.617 | -65.032 | Lid24_L05_201a | n | 4.12 | g |
| 265 | -7.619 | -65.030 | Lid24_L05_201b | n | 3.54 | s |
| 266 | -9.588 | -67.433 | Lid24_L05_29a | y | 0.83 | s |
| 267 | -9.589 | -67.437 | Lid24_L05_29b | y | 0.39 | s |
| 268 | -9.553 | -67.402 | Lid24_L05_32a | y | 0.69 | s |
| 269 | -9.557 | -67.401 | Lid24_L05_32b | n | 3.21 | g |
| *270 | -9.561 | -67.398 | Lid24_L05_32c | n | 0.76 | s |
| 271 | -9.560 | -67.405 | Lid24_L05_32d | n | 1.10 | s |
| 272 | -9.492 | -67.320 | Lid24_L05_38a | y | 1.97 | g |
| 273 | -9.893 | -67.808 | Lid24_L05_3a | n | 3.65 | g |
| 274 | -9.461 | -67.285 | Lid24_L05_40a | y | 0.66 | s |
| 275 | -9.460 | -67.284 | Lid24_L05_40b | y | 7.27 | g |
| 276 | -9.434 | -67.252 | Lid24_L05_42a | y | 8.46 | g |
| 277 | -9.444 | -67.262 | Lid24_L05_42b | y | 0.70 | s |
| 278 | -9.428 | -67.238 | Lid24_L05_43a | y | 5.72 | g |
| 279 | -9.424 | -67.239 | Lid24_L05_43b | y | 8.05 | g |
| 280 | -9.431 | -67.242 | Lid24_L05_43d | n | 2.25 | g |
| 281 | -9.420 | -67.229 | Lid24_L05_44a | y | 8.07 | g |
| 282 | -9.415 | -67.230 | Lid24_L05_44b | y | 0.78 | s |
| 283 | -9.408 | -67.211 | Lid24_L05_45a | y | 1.20 | s |
| 284 | -9.213 | -66.978 | Lid24_L05_62b | y | 3.71 | s |
| 285 | -9.182 | -66.943 | Lid24_L05_64a | y | 4.68 | g |
| 286 | -9.184 | -66.940 | Lid24_L05_64b | y | 3.89 | g |
| 287 | -9.184 | -66.943 | Lid24_L05_64c | n | 1.40 | s |
| 288 | -9.181 | -66.941 | Lid24_L05_65a | y | 6.42 | g |
| 289 | -9.140 | -66.889 | Lid24_L05_68a | n | 0.55 | s |
| 290 | -9.116 | -66.858 | Lid24_L05_70a | y | 2.12 | g |
| 291 | -9.122 | -66.865 | Lid24_L05_70b | y | 2.70 | s |
| 292 | -9.098 | -66.836 | Lid24_L05_72a | n | 7.49 | g |
| 293 | -9.097 | -66.836 | Lid24_L05_72b | y | 8.23 | s |
| 294 | -9.084 | -66.825 | Lid24_L05_73a | y | 2.72 | g |
| 295 | -9.057 | -66.790 | Lid24_L05_75a | y | 1.42 | g |
| 296 | -9.040 | -66.761 | Lid24_L05_77a | y | 7.36 | g |
| 297 | -9.041 | -66.772 | Lid24_L05_77b | y | 5.08 | g |
| 298 | -9.042 | -66.771 | Lid24_L05_77c | y | 1.37 | s |
| 299 | -8.978 | -66.685 | Lid24_L05_83a | y | 1.42 | s |
| 300 | -8.970 | -66.680 | Lid24_L05_83b | y | 5.61 | g |
| 301 | -8.970 | -66.678 | Lid24_L05_83c | y | 3.29 | s |
| 302 | -8.973 | -66.685 | Lid24_L05_83d | y | 1.14 | s |
| 303 | -8.938 | -66.636 | Lid24_L05_86a | y | 4.02 | s |
| 304 | -8.899 | -66.590 | Lid24_L05_89a | y | 1.14 | g |
| 305 | -8.848 | -66.529 | Lid24_L05_94a | y | 1.35 | g |
| 306 | -8.830 | -66.512 | Lid24_L05_95a | y | 1.64 | s |
| 307 | -8.818 | -66.495 | Lid24_L05_96a | y | 0.60 | g |
| 308 | -8.808 | -66.476 | Lid24_L05_97a | y | 0.92 | s |
| 309 | -8.435 | -66.173 | Lid24_L06_124a | y | 6.80 | g |
| 310 | -9.725 | -67.744 | Lid24_L06_12a | y | 0.37 | g |
| 311 | -8.253 | -65.948 | Lid24_L06_139a | y | 4.12 | g |
| 312 | -9.846 | -67.884 | Lid24_L06_1a | y | 1.20 | g |
| 313 | -7.495 | -65.031 | Lid24_L06_205a | n | 12.12 | s |
| 314 | -7.282 | -64.783 | Lid24_L06_223a | y | 1.14 | s |
| 315 | -9.555 | -67.544 | Lid24_L06_26a | n | 13.80 | g |
| 316 | -9.552 | -67.535 | Lid24_L06_26b | y | 0.28 | s |
| 317 | -9.541 | -67.526 | Lid24_L06_27a | y | 0.57 | s |
| 318 | -9.363 | -67.309 | Lid24_L06_43a | y | 0.28 | g |
| 319 | -9.356 | -67.290 | Lid24_L06_44a | y | 4.84 | g |
| 320 | -9.200 | -67.101 | Lid24_L06_57a | y | 1.99 | g |
| 321 | -9.194 | -67.101 | Lid24_L06_57b | n | 2.47 | g |
| 322 | -9.158 | -67.048 | Lid24_L06_61a | y | 6.68 | g |
| 323 | -9.152 | -67.050 | Lid24_L06_61b | y | 4.27 | g |
| 324 | -9.145 | -67.032 | Lid24_L06_62a | y | 2.76 | g |
| *325 | -9.106 | -66.981 | Lid24_L06_66a | n | 9.01 | s |
| 326 | -9.039 | -66.906 | Lid24_L06_71a | y | 2.90 | s |
| 327 | -9.037 | -66.906 | Lid24_L06_71b | y | 4.60 | g |
| 328 | -9.027 | -66.897 | Lid24_L06_72a | y | 5.28 | g |
| 329 | -9.031 | -66.902 | Lid24_L06_72b | y | 6.85 | s |
| 330 | -8.994 | -66.851 | Lid24_L06_75a | n | 0.92 | s |
| 331 | -8.905 | -66.745 | Lid24_L06_83a | y | 11.60 | g |
| 332 | -8.888 | -66.722 | Lid24_L06_84a | y | 0.51 | g |
| 333 | -8.880 | -66.719 | Lid24_L06_85a | y | 0.74 | s |
| 334 | -8.853 | -66.677 | Lid24_L06_87a | y | 1.35 | g |
| 335 | -8.784 | -66.597 | Lid24_L06_93a | y | 4.43 | g |
| 336 | -8.775 | -66.586 | Lid24_L06_94a | y | 11.83 | g |
| 337 | -8.773 | -66.578 | Lid24_L06_94b | y | 3.14 | s |
| 338 | -7.299 | -64.751 | Lid24_L07_179a | y | 2.00 | g |
| 339 | -7.298 | -64.753 | Lid24_L07_179b | y | 2.89 | g |
| 340 | -7.299 | -64.748 | Lid24_L07_179c | y | 0.71 | s |
| 341 | -7.307 | -64.747 | Lid24_L07_180a | y | 1.95 | g |
| 342 | -7.439 | -64.635 | Lid24_L07_185a | y | 1.42 | g |
| 343 | -7.487 | -64.593 | Lid24_L07_189a | y | 1.79 | g |
| 344 | -7.842 | -66.795 | Lid24_L08_147a | y | 9.79 | g |
| 345 | -7.846 | -66.799 | Lid24_L08_147b | y | 3.00 | g |
| 346 | -8.734 | -68.962 | Lid24_L08_18a | y | 0.39 | S |
| 347 | -8.722 | -68.936 | Lid24_L08_18b | y | 0.46 | s |
| 348 | -4.900 | -66.911 | Lid24_L09_1a | y | 1.73 | s |
| 349 | -4.898 | -66.908 | Lid24_L09_1b | y | 5.55 | g |
| 350 | -4.895 | -66.907 | Lid24_L09_1Ba | y | 0.23 | s |
| 351 | -4.896 | -66.907 | Lid24_L09_1Bb | y | 0.20 | s |
| 352 | -4.899 | -66.906 | Lid24_L09_1c | y | 0.26 | s |
| 353 | -4.899 | -66.906 | Lid24_L09_1d | y | 0.23 | s |
| 354 | -8.757 | -67.309 | Lid24_L09_215a | y | 3.94 | g |
| 355 | -9.085 | -67.460 | Lid24_L09_235a | y | 5.23 | g |
| 356 | -9.239 | -67.545 | Lid24_l09_245a | y | 1.15 | g |
| 357 | -9.257 | -67.556 | Lid24_L09_246a | y | 3.78 | s |
| 358 | -9.262 | -67.555 | Lid24_L09_246b | y | 2.46 | g |
| 359 | -9.259 | -67.555 | Lid24_L09_246c | y | 4.97 | s |
| 360 | -9.262 | -67.558 | Lid24_L09_246d | y | 5.14 | g |
| 361 | -9.262 | -67.556 | Lid24_L09_246e | y | 7.45 | s |
| 362 | -9.561 | -67.726 | Lid24_L09_265a | y | 1.20 | g |
| 363 | -9.557 | -67.716 | Lid24_L09_265b | y | 0.92 | g |
| 364 | -9.576 | -67.731 | Lid24_L09_266a | y | 3.84 | g |
| 365 | -9.585 | -67.740 | Lid24_L09_267a | y | 0.99 | s |
| 366 | -9.584 | -67.740 | Lid24_L09_267b | y | 1.57 | g |
| 367 | -9.603 | -67.742 | Lid24_L09_268a | y | 3.55 | s |
| 368 | -9.602 | -67.745 | Lid24_L09_268b | y | 5.92 | s |
| 369 | -9.622 | -67.754 | Lid24_L09_269a | y | 4.06 | g |
| 370 | -9.614 | -67.757 | Lid24_L09_269b | y | 1.18 | g |
| 371 | -9.659 | -67.781 | Lid24_L09_272a | y | 1.38 | g |
| 372 | -9.710 | -67.805 | Lid24_L09_275a | y | 2.68 | g |
| 373 | -8.973 | -69.147 | Lid24_L10_10a | n | 2.57 | s |
| 374 | -10.061 | -67.727 | Lid24_L10_111a | n | 0.68 | g |
| 375 | -10.066 | -67.720 | Lid24_L10_111b | n | 1.98 | s |
| 376 | -10.085 | -67.708 | Lid24_L10_112a | n | 0.51 | s |
| 377 | -10.156 | -67.649 | Lid24_L10_117a | n | 0.26 | s |
| 378 | -10.172 | -67.638 | Lid24_L10_119a | n | 4.01 | g |
| 379 | -10.174 | -67.636 | Lid24_L10_119b | n | 0.61 | s |
| 380 | -10.202 | -67.617 | Lid24_L10_120a | n | 0.38 | g |
| 381 | -10.202 | -67.616 | Lid24_L10_120b | n | 0.53 | g |
| 382 | -10.202 | -67.616 | Lid24_L10_120c | n | 0.77 | g |
| 383 | -10.203 | -67.617 | Lid24_L10_120d | n | 0.25 | g |
| 384 | -10.195 | -67.614 | Lid24_L10_120e | n | 1.14 | g |
| 385 | -10.190 | -67.614 | Lid24_L10_120f | n | 0.77 | s |
| 386 | -9.270 | -68.869 | Lid24_L10_32a | n | 0.86 | s |
| 387 | -9.590 | -68.510 | Lid24_L10_59a | n | 1.51 | g |
| 388 | -9.592 | -68.521 | Lid24_L10_59b | n | 4.66 | g |
| 389 | -9.630 | -68.433 | Lid24_L10_64a | n | 2.28 | g |
| 390 | -9.668 | -68.343 | Lid24_L10_69a | n | 0.99 | g |
| 391 | -9.756 | -68.134 | Lid24_L10_82a | n | 1.56 | g |
| 392 | -9.760 | -68.120 | Lid24_L10_83a | n | 4.48 | s |
| 393 | -9.934 | -67.569 | Lidar.Ac.1 - Estructura VI | y | 1.25 | g |
| 394 | -9.202 | -67.101 | MP.Am.17 - Estructura I | y | 6.57 | g |
| 395 | -9.373 | -67.318 | MP.Am.30 | y | 5.67 | g |
| 396 | -9.574 | -67.273 | MP.Am.36 - Estructura I | y | 2.22 | g |
| *397 | -9.577 | -67.272 | MP.Am.36 - Estructura II | y | 1.82 | s |
| 398 | -9.968 | -67.469 | Nakahara 108 - Estructura I | y | 0.76 | g |
| 399 | -9.967 | -67.469 | Nakahara 108 - Estructura II | y | 0.32 | g |
| *400 | -9.676 | -67.392 | Nakahara 185 | y | 2.14 | s |
| 401 | -9.668 | -67.392 | Nakahara 186 | y | 2.38 | g |
| *402 | -9.663 | -67.250 | Nakahara 188 | y | 6.67 | s |
| 403 | -9.953 | -67.595 | Nakahara 23 - Estrutura II-muretas | y | 3.88 | g |
| 404 | -9.796 | -67.544 | Nakahara AC 209 | n | 1.17 | s |
| *405 | -9.749 | -67.197 | Oco do Mundo | y | 4.67 | g |
| 406 | -9.621 | -67.759 | Vila do V | y | 6.38 | g |

**Supplementary Table 3. Size of rectangular mound settlements in SW Amazonia**. The list is based on Iriarte et al. 2020^19^, and the sizes are measured by us using satellite imagery. The number of mounds in Nakahara 174 Estrutura I is based on our own observations.

**Supplementary References**

57. Chandless, W. Notes on the River Aquiry, the principal Affluent of the River Purȗs. *Journal of the Royal Geographical Society of London* **36**, 119-126 (1866).

58. Pärssinen, M, Balée, W., Ranzi, A. & Barbosa, A.D. The geoglyph sites of Acre, Brazil: 10 000-year-old land-use practices and climate change in Amazonia. Antiquity **94**, 1538-1556 (2020).

59. Dias, O. As Estruturas Arqueológicas de Terra no Estado do Acre - Amazônia Ocidental, Brasil. Um caso de resiliência? in *Estudos Contemporâneos de Arqueologia* (eds. Dias, O, Carvalho, E. & Zimmermann, M.) 59-168 (Palmas: Unitins, IAB, 2006).
60. Saunaluoma, S. & Schaan, D. Monumentality in Western Amazonian formative societies: geometric ditched enclosures in the Brazilian state of Acre. *Antiqua* **2**:e1 (2012).
61. Neves, E.G. Ecology, Ceramic Chronology and Distribution. Long-Term History and Political Change in the Amazonian Floodplain, in *Handbook of South American Archaeology* (eds. Silvermann, H., & Isbell, W.) 359-379 (New York: Springer, 2008).
62. Saunaluoma, S. Geometric Earthworks in the State of Acre, Brazil: Excavations at the Fazenda Atlântica and Quinauá sites. *Lat. Am. Antiq.* **23**, 565-583 (2012).
63. Iriarte, J., de Souza, J.G., Robinson, M.E., Barbosa, A.D. & da Silva, F. Refining the Chronology and Occupation Dynamics of the Mound Villages of South-eastern Acre, Brazil. *Amazônica, Revista de Antropologia* **13**, 153-176 (2021).
64. Cook, N. D. *Demographic Collapse: Indian Peru 1520-1620*. (Cambridge: Cambridge University Press, 1981).
65. Mercier, V, Diario de una Expedición del Madre de Dios al Acre. La Paz: Imprenta La Tribuna 1894. *Colección de folletos bolivianos de Hoy* **3**, 1-16, Facsimilar (La Paz, Octubre 1981).
66. Labre, C.A.R.P. Colonel Labre’s Explorations in the Region between the Beni and Madre de Dios Rivers and the Purus. *Proceedings of the Royal Geographical Society and Monthly Record of Geography* **11**, 496–502 (1889).
67. Kraus, K., & Pfeifer, N. Advanced DTM generation from LIDAR data. *International Archives Of Photogrammetry Remote Sensing and Spatial Information Sciences* **34**(3/W4), 23-30 (2001).
68. Khosravipour, A., Skidmore, A.K., Isenburg, M. & Wang, T.J. Development of an algorithm to generate pit-free Digital Surface Models from LiDAR, *Proceedings of SilviLaser 2015*, 247-249 (2015).
69. Khosravipour, A., Skidmore, A. K., & Isenburg, M. Generating spike-free digital surface models using LiDAR raw point clouds: A new approach for forestry applications. *International Journal of Applied Earth Observation and Geoinformation* **52**, 104-114 (2016).
70. Sithole, G. & Vosselman, G. Experimental comparison of filter algorithms for bare-Earth extraction from airborne laser scanning point clouds. *ISPRS Journal of Photogrammetry and Remote Sensing* **59**, 85-101 (2004). <https://doi.org/10.1016/j.isprsjprs.2004.05.004>
71. Cățeanu, M. & Ciubotaru, A. The Effect of LiDAR Sampling Density on DTM Accuracy for Areas with Heavy Forest Cover. *Forests* **12**, 265 (2021). <https://doi.org/10.3390/f12030265>
72. Hyyppä, J. *et al.* Review of methods of small‐footprint airborne laser scanning for extracting forest inventory data in boreal forests. *International Journal of Remote Sensing* **29**, 1339-1366 (2008). <https://doi.org/10.1080/01431160701736489>
73. Ahokas, E., Hyyppä, J., Yu, X., Holopainen, M. Transmittance of Airborne Laser Scanning Pulses for Boreal Forest Elevation Modeling. *Remote Sensing* **3**, 1365-1379 (2011). <https://doi.org/10.3390/rs3071365>
74. Wood, R. From revolution to convention: the past, present and future of radiocarbon dating. *J. Archaeol. Sci.* **56**, 61-72 (2015).
75. Saunaluoma, S. Pre-Columbian Earthworks in the Riberalta Region of the Bolivian Amazon. *Amazônica, Revista de Antropologia* **2**, 86-111 (2010).
76. Pärssinen, M., Ranzi, A., Saunaluoma, S. & Siiriäinen, A. Geometrically patterned ancient earthworks in the Rio Branco Region of Acre, Brazil: New evidence of ancient chiefdom formations in Amazonian interfluvial *terra firme* environment, in *Western Amazonia - Amazônia Ocidental.* *Multidisciplinary Studies on Ancient Expansionistic Movements, Fortifications* *and Sedentary Life* (eds. Pärssinen, M. & Korpisaari, A.) 97-133 (Helsinki: Renvall Institute Publications, University of Helsinki, 2003).
77. Saunaluoma, S., Moat, J., Pugliese, F. & Neves, E.G. Patterned Villagescapes and Road Networks in Ancient Southwestern Amazonia. *Lat. Am. Antiq.* **32**, 173-187 (2021).
78. Neves, E. G. *et al.* Pesquisa e Formação nos Sítios Arqueológicos Espinhara e Sol de Campinas do Acre – PESC: Relatório Final. Universidade de São Paulo. Manuscript (2016). DOI:[10.13140/RG.2.2.27534.72006](http://dx.doi.org/10.13140/RG.2.2.27534.72006)
